# Heterobimetallic Pd–K carbene complexes *via* one-electron reductions of palladium radical carbenes†

**DOI:** 10.1039/c6sc00948d

**Published:** 2016-03-24

**Authors:** Peng Cui, Melissa R. Hoffbauer, Mariya Vyushkova, Vlad M. Iluc

**Affiliations:** a Department of Chemistry and Biochemistry, University of Notre Dame Notre Dame IN 46556 USA viluc@nd.edu; b Notre Dame Radiation Laboratory, University of Notre Dame Notre Dame IN 46556 USA

## Abstract

Heterobimetallic Pd–K carbenes featuring Pd–C_carbene_–K moieties were synthesized *via* an unprecedented sequential substitution/reduction reaction from a radical precursor, [{PC˙(sp^2^)P}^*t*Bu^PdI] ([PC(sp^2^)P]^*t*Bu^ = bis[2-(di-iso-propylphosphino)-4-*tert*-butylphenyl]methylene). Polymeric structures were observed in the solid state for the heterobimetallic compounds that can be interrupted in the presence of a donor solvent.

## Abstract 2

Unprecedented sequential substitution/reduction synthetic strategy on the Pd radical carbenes afforded heterobimetallic Pd–K carbene complexes, which features novel Pd–C_carbene_–K structural moieties.

## Graphical abstract

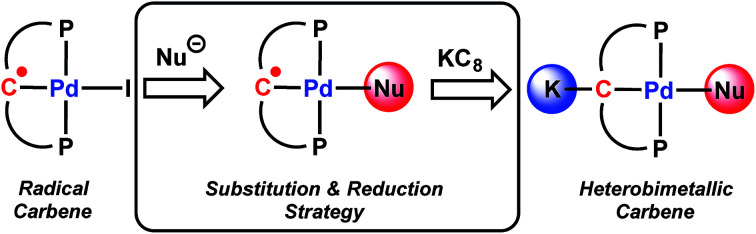

## Introduction

The influence of Lewis acids in catalysis cannot be underestimated. While most efforts have focused on using them as the sole mediators of chemical transformations,^1–5^ few examples have been reported that discuss the role of Lewis acids as co-activators in homogeneous catalysis.^6–9^ These are related to heterogeneous processes,^10,11^ with a singular example discussing the role of potassium ions on the hydrogenation of dinitrogen in the Haber–Bosch process,^12^ and biological systems, with several examples reporting that Lewis acids are necessary cofactors that help modulate the redox properties of the oxygen-evolving complex's manganese cluster and likely its reactivity.^13–17^ In organic synthesis, Shibasaki's rare-earth alkali-metal heterobimetallic complexes^18^ are among the most enantioselective and broadly used catalysts to date,^19–21^ but few studies discuss the role of Lewis acids in applications of late transition metals, in general, and of carbene complexes, in particular.^22–24^

Transition metal carbenes are of vital importance to science and have witnessed a tremendous progress in their applications in the past few decades.^25–32^ Late transition metal complexes with N-heterocyclic,^33,34^ carbocyclic,^35^ and heteroatom stabilized carbene ligands^36–38^ have been extensively studied as a consequence; however, the corresponding non-heteroatom stabilized species, [M=CRR′] (R, R′ = alkyl or H), are less explored.^39–45^ This situation is even more pronounced for group 10 metals, likely because these metals are too electron rich to stabilize the M=C bond. Pioneering work by Hillhouse showed that a Ni(0) carbene containing a CPh_2_ moiety could be isolated by the thermolysis or photolysis of its diphenyldiazoalkane precursor.^46,47^ The isolation of the corresponding Pd and Pt carbenes is, however, more challenging than that of nickel complexes,^48–54^ because of the highly reactive nature of the former.^55,56^ On the other hand, these species are crucial intermediates in a variety of catalytic transformations,^57,58^ such as palladium carbene mediated cyclopropanations, cross-coupling with diazo compounds, and migratory insertion reactions.^59^

**Chart 1:**
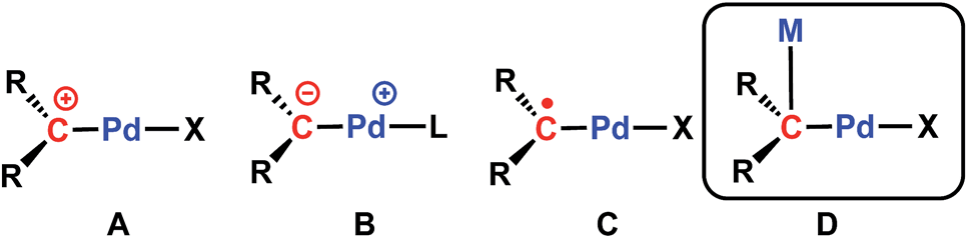
Illustration of known palladium carbene complexes: (A) cationic carbene; (B) ylide-like carbene; (C) radical carbene; (D) heterobimetallic carbene discussed in this work.

It is worth mentioning that only two examples of cationic Pd(ii) carbene complexes are known (Chart 1, type A), synthesized *via* triflate or hydride abstraction, and that their reactivity has not been studied.^48,49^ A salt metathesis strategy was also reported for the synthesis of methanediide-based Pd(ii) carbene complexes.^50,51^ We have recently applied dehydrohalogenation reactions^60^ to synthesize Pd(ii) carbene complexes, [{PC(sp^2^)P}^R^Pd(PMe_3_)] (R = H, [PC(sp^2^)P]^H^ = bis[2-(di-iso-propylphosphino)phenyl]methylene); R = ^*t*^Bu, ([PC(sp^2^)P]^*t*Bu^ = bis[2-(di-iso-propylphosphino)-4-*tert*-butylphenyl]methylene).^61–63^ Interestingly, the Pd–C_carbene_ bonds in these compounds are best described as ylide-like (Chart 1, type B), as demonstrated by their strong nucleophilic reactivity toward polar substrates (MeI, HCl, MeOH, *para*-toluidine),^61^ strong Lewis acids,^62^ C–H^64^ and Si–H^65^ bond activation reactions. Furthermore, our recent study on the redox-induced umpolung of palladium carbenes revealed that the radical carbene [{PC˙(sp^2^)P}^*t*Bu^PdI] (1, Chart 1, type C) bridges cationic and anionic carbenes *via* reversible one-electron transfer processes.^66,67^

We reasoned that the presence of iodide as a leaving group in 1 would facilitate its substitution with various anionic nucleophiles to afford new radical carbene species, which would generate Pd(ii) carbenes with functional groups when subsequent one-electron reductions are applied. Herein, we report a sequential substitution/reduction reaction of the radical carbene 1 to form heterobimetallic Pd–K carbene complexes featuring amides or benzyl ligands that represent rare examples of late transition metal complexes containing Pd–C_carbene_–M units (Chart 1, type D). Such bonding motifs were recently proposed to be instrumental in the hydroarylation of dienes catalysed by rhodium carbodicarbene complexes, but structural characterization was not available.^22^

## Results and discussion

### Synthesis and characterization of metal complexes

**Scheme 1:**
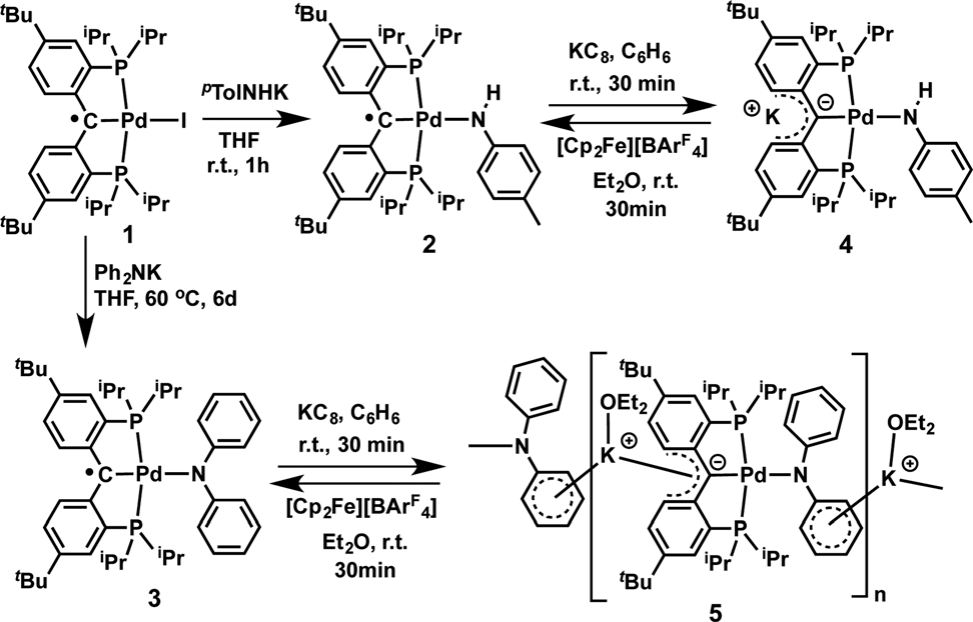
Synthesis of heterobimetallic carbene complexes 4 and 5.

**Fig. 1:**
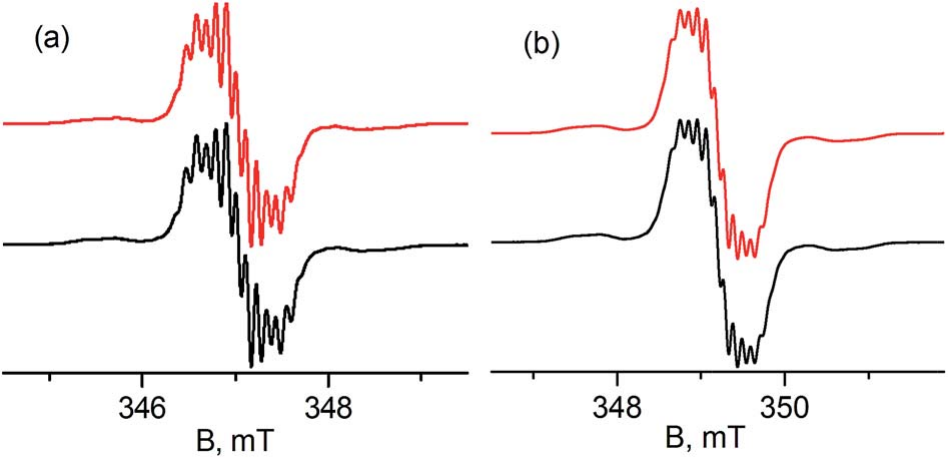
Experimental (black) and simulated (red) X-band EPR spectra of 10^−4^ M toluene solutions of radicals 2 (a) and 3 (b) at 298 K. Simulation parameters for 2: *a*_1_(2H) = 0.32 mT, *a*_2_(2H) = 0.13 mT, *a*_3_(2H) = 0.11 mT, *a*(N) = 0.09 mT, *a*(^105^Pd) = 0.47 mT, *g* = 2.0088; for 3: *a*_1_(2H) = 0.31 mT, *a*_2_(2H) = 0.13 mT, *a*_3_(2H) = 0.10 mT, *a*(N) = 0.09 mT, *a*(^105^Pd) = 0.51 mT, *g* = 2.0079. Contribution from the ^105^Pd species is 22.33% for both radicals.

Treatment of 1 with potassium amides, R^1^R^2^NK, in THF afforded new radical complexes, [{PC˙(sp^2^)P}^*t*Bu^PdNR^1^R^2^] (2: R^1^ = H, R^2^ = ^*p*^Tol; 3: R^1^ = R^2^ = Ph, Scheme 1), which were crystallized from *n*-pentane at −35 °C as dark-green crystals in high yield. As observed for 1,^67^2 and 3 are silent by ^1^H and ^31^P NMR spectroscopy and are thermally robust. The effective magnetic moments *μ*_eff_ of 1.70 *μ*_B_ and 1.54 *μ*_B_ obtained using the Evans method^68^ indicated an *S* = 1/2 ground state for both compounds. EPR spectra of radicals 2 and 3 (Fig. 1) exhibit similar hyperfine patterns arising from an interaction of the unpaired electron with three pairs of magnetically equivalent protons, one nitrogen nucleus, and a ^105^Pd nucleus. As the natural abundance of the magnetic ^105^Pd isotope is 22.33%, each spectrum exhibits contributions from the non-magnetic palladium isotope (strong, well-resolved central signal) and the ^105^Pd isotope (broad wings). Proton hyperfine couplings were assigned to protons of the phenyl rings adjacent to the radical center.

**Fig. 2:**
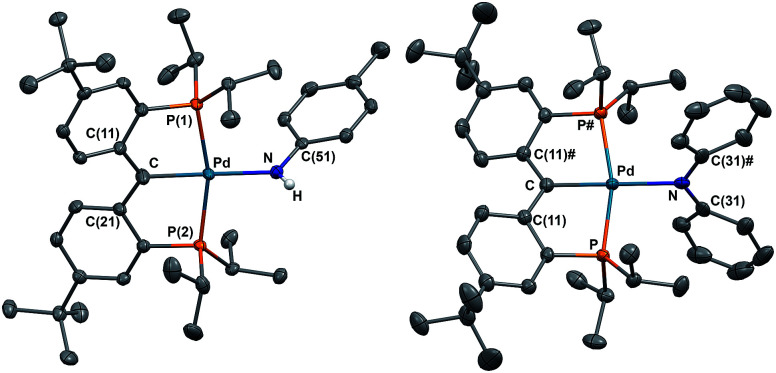
Thermal-ellipsoid (35% probability) representation of 2 (left) and 3 (right); most hydrogen atoms were removed for clarity. Selected distances [Å] and angles [°] for 2: Pd–C 2.019(2), Pd–P(1) 2.2983(5), Pd–P(2) 2.2841(5), Pd–N 2.0787(18), Pd–C–C(11) 118.57(15), Pd–C–C(21) 119.30(15), C(11)–C–(21) 121.98(19); for 3: Pd–C 2.024(2), Pd–P 2.3021(5), Pd–N 2.149(2), Pd–C–C(11) 118.97(11), C(11)–C–(11)# 122.1(2).

The solid state molecular structures of 2 and 3 are consistent with their radical carbene nature (Fig. 2): both contain square-planar palladium centers bound to sp^2^ hybridized backbone carbons (*Σ*_angles_ at C_carbene_ are 359.9° for 2 and 360.0° for 3). The Pd–C_carbene_ distances in 2 (2.019(2) Å) and 3 (2.024(2) Å) are comparable to each other and close to the value of 2.022(3) Å in 1.^67^ Trigonal planar geometries were observed for both amide nitrogen atoms in 2 and 3, with the amide planes roughly perpendicular to the planes defined by C_carbene_, C(11), and C(21) or C(11)^#^ (80.6° for 2 and 85.8° for 3). A longer Pd–N distance of 2.149(2) Å was observed in 3 compared to 2.0787(18) Å for 2, attributed to steric reasons.

**Fig. 3:**
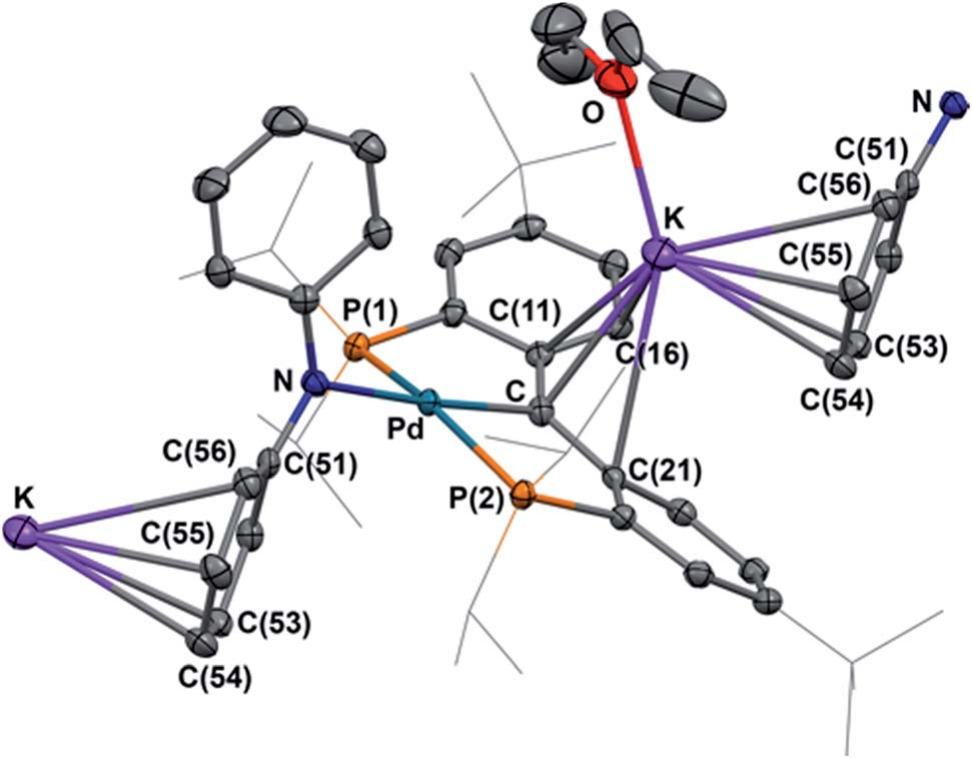
Thermal-ellipsoid (35% probability) representation of 5; most hydrogen atoms were removed for clarity. Selected distances [Å] and angles [°]: Pd–C 2.043(2), Pd–P(1) 2.2827(5), Pd–P(2) 2.3064(5), Pd–N 2.1445(16), K–C 2.939(2), K–C(11) 3.047(2), K–C(21) 3.292(2), K–C(16) 3.069(2), K–C(53) 3.240(2), K–C(54) 3.004(2), K–C(55) 3.062(2), K–C(56) 3.372(2), Pd–C–C(11) 118.45(14), Pd–C–C(21) 118.31(14), C(11)–C–(21) 123.14(18).

Both radical complexes were subsequently treated with an equivalent of KC_8_ in benzene, and the corresponding diamagnetic heterobimetallic complexes [K(OEt_2_)_*n*_][{PC(sp^2^)P}^*t*Bu^PdNR^1^R^2^] (4: R^1^ = H, R^2^ = ^*p*^Tol, *n* = 0; 5: R^1^ = R^2^ = Ph, *n* = 1) were obtained in high yield (Scheme 1). Both compounds are only soluble in ethereal solvents and were recrystallized by diethyl ether/*n*-pentane diffusion at ambient temperature. The solid state molecular structure of 5 was determined by X-ray diffraction studies (Fig. 3). Compound 5 exists as a notable polymer in which the anionic [{PC(sp^2^)P}^*t*Bu^PdNPh_2_] moieties are bridged by potassium ions through the carbene units and one phenyl ring of the amide group. The average K–C distances are slightly shorter when potassium binds the phenyl ring of the amide group rather than the carbene moiety (3.08 Å *vs.* 3.17 Å). The coordination sphere of potassium was further completed by an additional diethyl ether molecule. The C_carbene_ carbon retains its trigonal planar geometry (*Σ*_angles_ at C_carbene_ are 359.9°) with a Pd–C_carbene_ distance of 2.043(2) Å, which is shorter than the corresponding value of 2.076(3) Å in [{PC(sp^2^)P}^*t*Bu^Pd(PMe_3_)].^62^ The trigonal planar geometry at the amide nitrogen was also retained, but the dihedral angle between the amide plane and the plane defined by C_carbene_, C(11) and C(21) was much smaller than that in 3 (59.4° *vs.* 85.8°), probably due to the more congested environment found in the polymer.

Due to its poor solubility in non-coordinating solvents, the solution behaviour of 5 was studied in THF-*d*_8_. The ^1^H NMR spectrum in THF-*d*_8_ recorded at 298 K showed the peaks for the free Et_2_O at *δ* = 3.39 (q) and 1.12 (t) ppm, thus indicating its labile coordination that led to its displacement by the THF-*d*_8_ molecules. Unlike the polymeric structure observed in the solid state, 5 exhibited a *C*_2_ symmetry in the THF-*d*_8_ solution, as evidenced by only a broad peak at *δ* = 48.64 ppm (Δ*ν*^1/2^ ≈ 870 Hz) in the ^31^P{^1^H} NMR spectrum, as well as three broad peaks for the ^i^Pr (*δ* = 1.98 and 1.14 ppm) and ^*t*^Bu (*δ* = 1.06 ppm) groups. Therefore, the polymeric structure of 5 is disrupted in the donor solvent and even in the presence of a small amount of THF-*d*_8_ since the ^1^H NMR spectrum of 5 in C_6_D_6_ with 2 drops of THF-*d*_8_ also showed *C*_2_ symmetry.

Interestingly, the NPh_2_ group on palladium exhibited three well-resolved peaks at *δ* = 7.35 (d), 6.80 (t), and 6.18 (t) ppm in the ^1^H NMR spectrum of 5 in THF-*d*_8_, however, no resonances were observed from the phenyl rings of the supporting ligand in both ^1^H and ^13^C{^1^H} NMR spectra. The variable temperature NMR spectra recorded in the temperature range of 208 to 323 K in THF-*d*_8_ did not show any changes for these resonances. Only broadening of all the other peaks was observed in the ^1^H NMR spectrum at 208 K, while a relatively sharp (Δ*ν*^1/2^ ≈ 320 Hz) peak was found in the ^31^P{^1^H} NMR spectrum (see Fig. S35 and S36†). The phenyl rings of the supporting ligand are NMR silent in this whole temperature range. It is known that larger alkali metals such as potassium prefer anionic units that can delocalize the electron density, thus shifting the coordination of potassium away from the carbanion to the peripheral phenyl rings, leading to an η^6^-coordination mode.^69,70^ Therefore, the observed solution behaviour might be explained by a fast motion of the potassium ions between the two phenyl rings of the supporting ligand that cannot be frozen out at 208 K, causing a *C*_2_ symmetry on the NMR time scale. A comparable system has been reported for arene complexes of yttrium supported by a macrocyclic [P_2_N_2_] ligand ([P_2_N_2_] = [PhP(CH_2_SiMe_2_NSiMe_2_CH_2_)_2_PPh]).^71^ However, since all the signals from the phenyl rings of the supporting ligand could not be observed in the whole temperature range, a non-dynamic process caused by the formation of other polymers or oligomers containing bridging potassium ions cannot be ruled out.^72^

The ^1^H NMR spectrum of 4 in THF-*d*_8_ also exhibited a similar *C*_2_ symmetry as observed for 5. No coordination of diethyl ether was observed, even though 4 was crystallized from a diethyl ether/*n*-pentane mixture, indicating a slightly different bonding mode of potassium in 4, caused by the different amide substituent on palladium. Unlike those in 5, the phenyl groups from the supporting ligand in 4 exhibited three broad singlets at *δ* = 7.23, 6.61 and 6.54 ppm in the ^1^H NMR spectrum. Only one sharp singlet at *δ* = 46.36 ppm was observed in the ^31^P{^1^H} NMR spectrum. Therefore, a similar polymeric chain can be proposed for complex 4 (*vide infra*). Notably, the proton of the NH group appears as a singlet at *δ* = 1.33 ppm, thus the formation of a possible [Pd=N^*p*^Tol] or [Pd–N^*p*^Tol–Pd] imido species is excluded.

Our study of the solution behavior of 4 and 5 shows that not only the polymeric structure can be disrupted by donor solvents such as THF, but that the mobility of the potassium cation increases as well. These findings support the proposal by Meek and coworkers that the interaction of a rhodium catalyst for the hydroarylation of dienes with a Lewis acid has to be reversible in order to observe an increased activation of the substrate.^22^

**Scheme 2:**
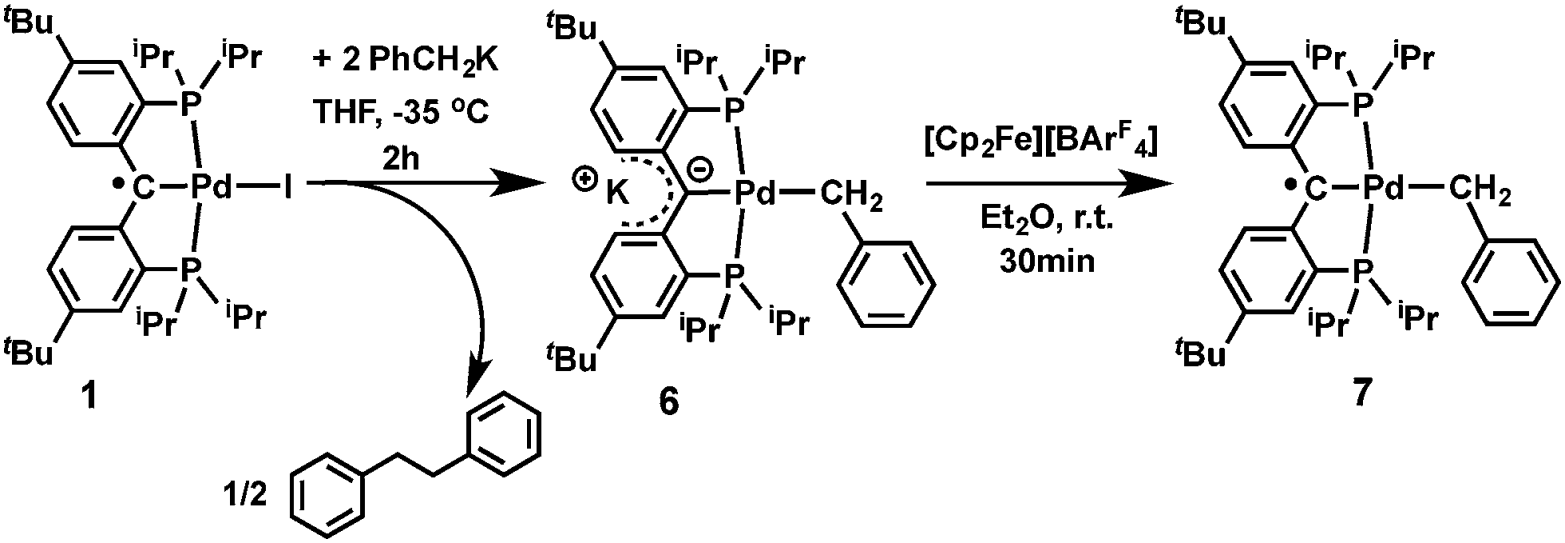
Synthesis of 6 and 7.

Heterobimetallic carbene complexes^73,74^ containing K–C_carbene_–M units, in which the carbene is not stabilized by adjacent heteroatoms, are rare;^23,75^ to the best of our knowledge, complexes 4 and 5 represent the only known characterized heterobimetallic carbene species possessing such binding motifs. The successful synthesis of amide substituted carbene complexes 4 and 5 from the radical precursor 1 prompted us to investigate other nucleophiles, especially those featuring an alkyl group. Interestingly, the reaction of 1 with two equivalents of PhCH_2_K in THF led to a dark-brown diamagnetic complex, K[{PC(sp^2^)P}^*t*Bu^Pd(CH_2_Ph)] (6), in 64% yield, instead of the expected benzyl substituted Pd(ii) radical carbene [{PC˙(sp^2^)P}^*t*Bu^Pd(CH_2_Ph)] (7, Scheme 2). Using one equivalent of PhCH_2_K also led to 6, albeit with a lower conversion.

**Fig. 4:**
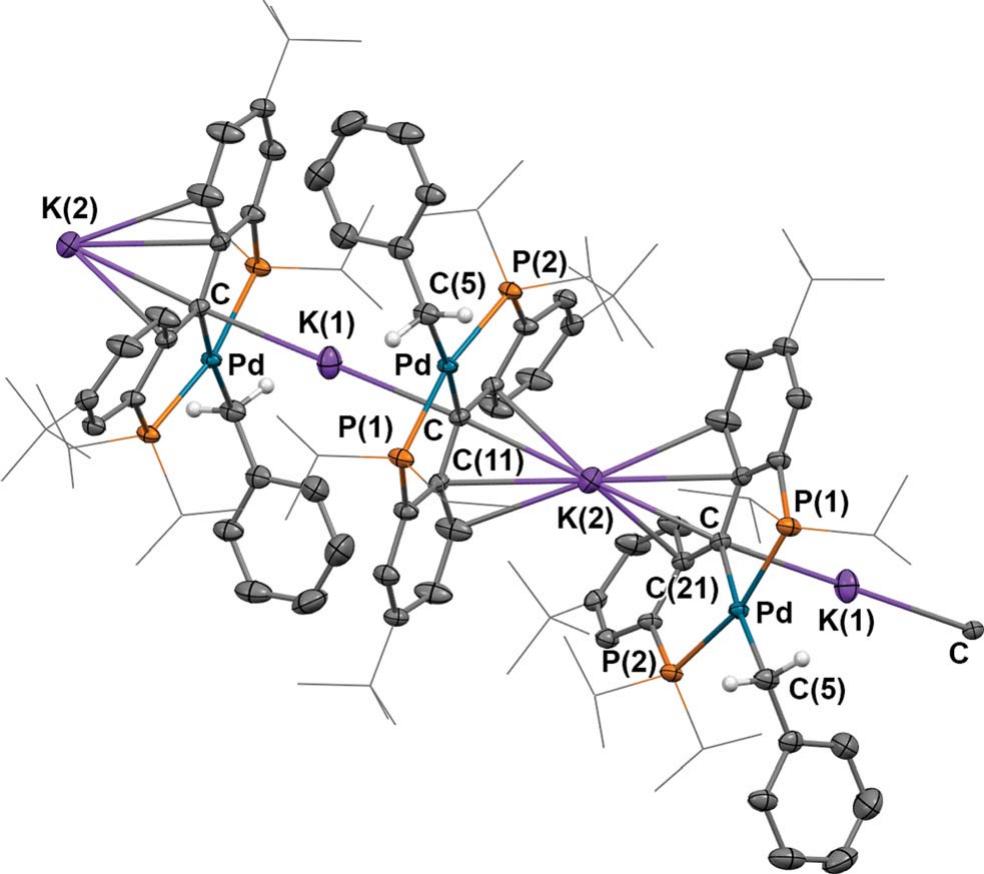
Thermal-ellipsoid (35% probability) representation of 6; most hydrogen atoms were removed for clarity. Selected distances [Å] and angles [°]: Pd–C 2.095(2), Pd–P(1) 2.2695(6), Pd–P(2) 2.2868(6), Pd–C(5) 2.163(2), K(1)–C 3.040(2), K(2)–C 3.028(2), K(2)–C(11) 3.162(2), K(2)–C(16) 3.176(3), K(2)–C(21) 2.998(2), Pd–C–C(11) 118.76(15), Pd–C–C(21) 118.06(15), C(11)–C–(21) 123.02(19).

As observed for 5, X-ray diffraction studies on single crystals of 6 grown from a diethyl ether solution also showed a polymeric structure, where the anionic carbene moieties [{PC(sp^2^)P}^*t*Bu^PdCH_2_Ph] were bridged by potassium ions (Fig. 4). Although the C_carbene_ carbon still retained its trigonal planar geometry (*Σ*_angles_ at C_carbene_ are 359.8°), the Pd–C_carbene_ distance of 2.095(2) Å is slightly longer than that of 2.076(3) Å in [{PC(sp^2^)P}^*t*Bu^Pd(PMe_3_)],^62^ and of 2.043(2) Å in 5. Two different coordination modes were found for potassium ions: one is sandwiched between two [{PC(sp^2^)P}^*t*Bu^PdCH_2_Ph] moieties in an η^4^ fashion, with K–C distances ranging from 2.998(2) to 3.176(3) Å, while the other is only η^1^ coordinated to the carbene carbons of two anionic carbene moieties, with a K–C distance of 3.040(2) Å. The K–C_carbene_–K–C_carbene_ chain is not strictly linear, with the K–C_carbene_–K and C_carbene_–K–C_carbene_ angles being 163.40(8)° and 173.86(9)°, respectively. The different bridging modes observed for potassium in 5 and 6 are largely attributed to the bulkier Ph_2_N amide group in 5 than the benzyl group in 6.

Similarly to 4 and 5, complex 6 is only soluble in ethereal solvents and its ^1^H NMR spectrum recorded in THF-*d*_8_ is consistent with a *C*_2_ symmetric structure in solution. The ^31^P {^1^H} NMR spectrum only shows a sharp singlet at *δ* = 48.52 ppm. A triplet at *δ* = 2.45 ppm (^3^*J*_PH_ = 5.0 Hz) was observed for the benzyl CH_2_ group in the ^1^H NMR spectrum that correlates with a triplet at *δ* = 14.12 ppm (^2^*J*_PC_ = 9.0 Hz) in the corresponding ^13^C{^1^H} NMR spectrum. The peak corresponding to the carbene carbon could not be assigned unambiguously in the ^13^C{^1^H} NMR spectrum, a situation previously observed for [{PC(sp^2^)P}^H^PdPMe_3_],^61^ as well as for other palladium carbene complexes.^50^

As observed for 4 and 5, a discrepancy between the solid state and THF-*d*_8_ solution structures of 6 exists that suggests a disruption of the polymeric chain by the donor solvent. In the ^1^H NMR spectrum, three well-resolved peaks at *δ* = 7.20 (dt), 6.66 (td) and 6.52 (dd) ppm were assigned to the signals from the phenyl groups of the supporting ligand, which are very close to those of 4. Considering that benzyl and NH^*p*^Tol groups have similar steric profiles, complexes 4 and 6 most likely possess similar solution structures. The ^1^H NMR spectrum of 6 in C_6_D_6_ with 2 drops of THF-*d*_8_ also showed a *C*_2_ symmetric structure. Notably, resonances of the phenyl groups of the supporting ligand are significantly shifted upfield in THF-*d*_8_ compared to those in C_6_D_6_ with only a small amount of THF-*d*_8_ (from 7.61, 6.94, and 6.86 ppm to 7.20, 6.66, and 6.52 ppm, respectively), consistent with the generation in THF-*d*_8_ of a monomer, in which the potassium ion interacts with only one carbene moiety and not with two, as observed in the solid state or in a non-donor solvent.^76^

The formation of 6 is a combined substitution and reduction of radical complex 1: the benzyl group on palladium was introduced by substitution of iodide by a benzyl anion. Due to the reductive nature of the benzyl anion, the loss of an electron from it also reduced the radical species to an anionic carbene. This process is accompanied by the formation of the benzyl radical [PhCH_2_˙], which dimerized to form PhCH_2_CH_2_Ph. Analysis of the crude reaction mixture by ^1^H NMR spectroscopy indicated the formation of PhCH_2_CH_2_Ph, which was confirmed by comparison with an authentic sample. A minor palladium containing species was also observed, which showed two sets of peaks for the benzylic CH_2_ groups at *δ* = 3.81 (s) and 2.94 (^3^*J*_PH_ = 5.5 Hz) ppm in the ^1^H NMR spectrum, and a sharp singlet at *δ* = 39.86 ppm in the ^31^P{^1^H} NMR spectrum. We tentatively assigned this species as complex [{PC(sp^3^)(CH_2_Ph)P}^*t*Bu^Pd(CH_2_Ph)], containing another benzyl group on the backbone carbon *via* radical coupling. Attempts to isolate this compound were hampered by its high solubility in aliphatic solvents.

Although the substitution reaction of 1 with PhCH_2_K led directly to the formation of the heterobimetallic carbene complex 6, the subsequent one-electron oxidation of 6 with [Cp_2_Fe][BAr^F^_4_] afforded the radical complex 7 in quantitative yield (Scheme 2). The high solubility of 7 in aliphatic solvents prevented its separation from the byproduct, Cp_2_Fe, but its EPR spectrum (in the presence of Cp_2_Fe) in toluene at 298 K revealed a hyperfine splitting from four pairs of magnetically equivalent protons and a ^105^Pd nucleus (Fig. S5 and S6†). The smallest hyperfine constant was attributed to the CH_2_ group protons (0.08 mT), while the remaining proton hyperfine constants (0.32, 0.19 and 0.12 mT) were assigned to the six phenyl ring protons of the supporting ligand, which are close to the corresponding values for 2 and 3. Accordingly, the one-electron oxidations of the heterobimetallic carbene complexes 4 and 5 with [Cp_2_Fe][BAr^F^_4_] also regenerated the radical complexes 2 and 3, respectively (Scheme 1), as confirmed by EPR spectroscopy (Fig. S1 and S3†).

Preliminary reactivity studies showed that the reaction of 6 with CH_3_CN (5 equivalents) in THF quantitatively afforded [{PC(sp^3^)HP}^*t*Bu^Pd(CH_2_Ph)] (8, eqn (1)), which is the product of C–H activation, similarly to [{PC(sp^2^)P}^H^Pd(PMe_3_)].^61,62^ Given the presence of benzyl and amide ligands that could have been protonated by CH_3_CN, it is interesting to note that the former carbene carbon is more nucleophilic than these ligands, consistent with a localized negative charge on that carbon.1
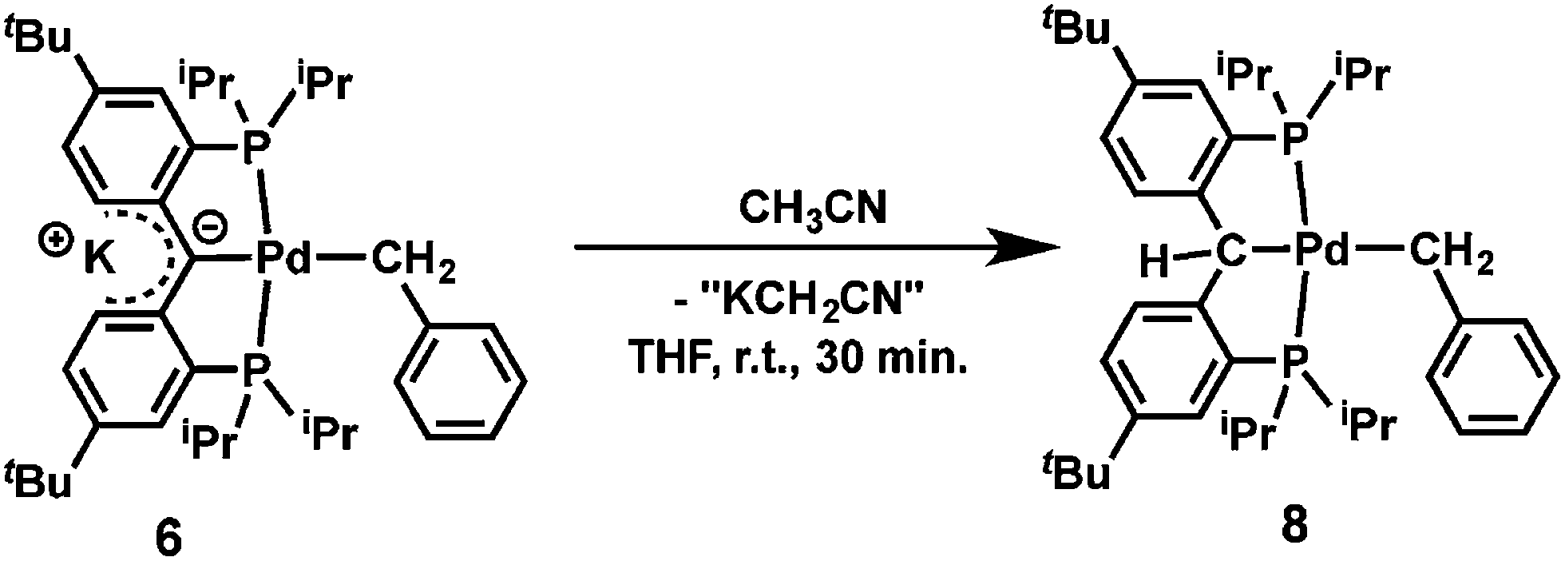


We also tested the direct nucleophilic substitutions of the parent carbene complex [{PC(sp^2^)P}^*t*Bu^Pd(PMe_3_)] with ^*p*^TolNHK, Ph_2_NK and PhCH_2_K. Although the formation of the heterobimetallic complexes 4 and 6 was confirmed (^*p*^TolNHK, 65% and PhCH_2_K, 60% conversion) by ^31^P{^1^H} NMR spectra, some unknown species as well as unreacted starting material were also observed after 24 h. Attempts to isolate pure 4 and 6 from these mixtures met with difficulty and were inefficient. The bulky nucleophile Ph_2_NK does not react with [{PC(sp^2^)P}^*t*Bu^Pd(PMe_3_)] under similar conditions, probably due to its steric hindrance. The sluggish substitution reactions observed for these nucleophiles could also be attributed to the relatively strong coordination of PMe_3_ to palladium, therefore, the substitution/reduction strategy presented herein is preferable because it takes advantage of a facile redox process that provides a relatively easier access to heterobimetallic Pd–K carbene complexes.

### DFT calculations

**Fig. 5:**
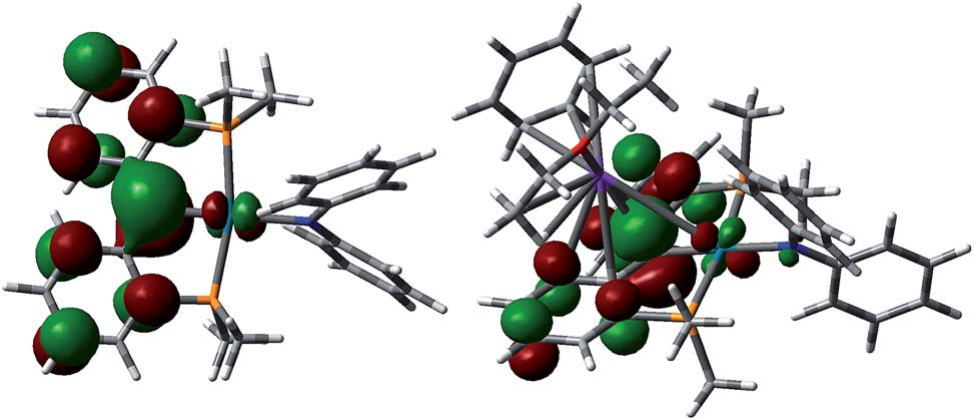
HOMO for [{PC(sp^2^)P}^Me^PdNPh_2_]^−^ (5′, left) and [{PC(sp^2^)-K(OEt_2_)(C_6_H_6_)P}^Me^PdNPh_2_] (5′′, right).

DFT calculations (B3LYP functional, LANL2DZ basis set) were performed on a model of the anion of 5, 5′, with the ^*t*^Bu and ^i^Pr groups replaced by H and methyl groups, respectively (Fig. 5). As was observed for [{PC(sp^2^)P}^R^Pd(PMe_3_)],^61,62^ the HOMO of 5′ shows a π type interaction between the carbene carbon p orbital and the appropriate symmetry d orbital of Pd, while the LUMO shows the corresponding σ interaction, with both molecular orbitals having antibonding character. Thus the carbene moiety in these heterobimetallic carbene complexes is predicted to show some similar reactivity to that observed for [{PC(sp^2^)P}^R^Pd(PMe_3_)], as was shown in eqn (1).^62,64,66^

In order to study the influence of the potassium cation on the Pd–C interaction, another simplified model, 5′′, was considered, in which the interaction between potassium and a NPh_2_ group from a neighboring molecule was replaced with a potassium benzene interaction. The HOMO of 5′′ (Fig. 5) resembles that calculated for 5′, in agreement with an electrostatic interaction between the potassium cation and the anionic carbon, while the LUMO is localized on the benzene ring coordinated to the potassium atom. The interaction between the cation and the carbene ligand observed in both the solid state structure and the computed model involves the delocalized π orbital of the two phenyl rings attached to the carbene atom. That carbon maintains its planar geometry in both 5 and 6, similarly to other delocalized π systems coordinated to potassium.^77–79^

A similar situation was observed for 6. We studied the electronic structure of the free anion (6′) and a model of the heterobimetallic carbene (6′′). In both, the iso-propyl groups and the *tert*-butyl groups were replaced by hydrogen atoms. For 6′′, in order to simulate the coordination environments of both potassium atoms, we reduced the polymer to a dimer (Fig. S24 and S25†). Both HOMO and HOMO−1 for 6′′ are comprised of the lone pair on the carbenic carbon; LUMO and LUMO+1 are localized on the benzene rings coordinated to the terminal potassium cations. The planarity of the carbenic carbons can be attributed to the symmetric donation from the lone pair on these atoms to the two potassium atoms.

## Conclusions

In conclusion, we showed that a substitution/reduction strategy employing the radical carbene [{PC˙(sp^2^)P}^*t*Bu^PdI] (1) can be utilized to synthesize a series of heterobimetallic Pd–K carbene complexes {[{PC(sp^2^)P}^*t*Bu^PdX]K(OEt_2_)_*n*_} (4: X = NH^*p*^Tol, *n* = 0; 5: X = NPh_2_, *n* = 1; 6: X = CH_2_Ph, *n* = 0) bearing functional groups on palladium. Polymeric structures were exhibited by these heterobimetallic Pd–K carbenes in the solid state, featuring unprecedented Pd–C_carbene_–K units that are not easily accessible by other synthetic strategies. The present carbenes complete the palladium carbene series containing a cationic (Chart 1, type A), an ylide-like (type B), a radical (type C), and a heterobimetallic (type D) carbene. The isolation of these novel species not only provides a fundamental understanding of their bonding and structural features, but also sheds some light on the role of alkalis in transition metal catalysis. Notably, the solution behaviour of these complexes showed that the interaction of the potassium ion with the carbene moiety is highly influenced by the donating ability of the solvent, which led to a lower aggregation state of the complex; this may better represent species related to transition metal catalytic systems with alkali metals in polar solvents. More importantly, the flexible interaction of potassium might also be crucial in terms of substrate activation, as proposed recently in the rhodium catalyzed hydroarylation of dienes, that a reversible interaction with Lewis acids enhance the reactivity.^22^ Finally, the facile redox properties exhibited by these palladium carbenes also indicate their potential non-innocent ligand-based reactivity, and, therefore, future studies can lead to new and useful transformations in synthesis.

## Experimental

All experiments are performed under an inert atmosphere of N_2_ using standard glovebox techniques. Solvents hexanes, *n*-pentane, diethyl ether, and CH_2_Cl_2_ were dried by passing through a column of activated alumina and stored in the glovebox. THF and THF-*d*_8_ were dried over LiAlH_4_ followed by vacuum transfer and stored in the glovebox, while C_6_D_6_ was dried over CaH_2_ followed by vacuum transfer, and stored in the glovebox. Complex 1,^67^ [Cp_2_Fe][BAr^F^_4_],^80^ and KC_8_^81^ were prepared according to literature procedures. ^*p*^TolNHLi, ^*p*^TolNHK and Ph_2_NK were prepared by deprotonation of amines with ^*n*^BuLi and KH, respectively. ^1^H, ^13^C{^1^H}, ^31^P{^1^H} NMR spectra were recorded on a Bruker DRX 500 spectrometer. All chemical shifts are reported in *δ* (ppm) with reference to the residual solvent resonance of deuterated solvents for proton and carbon chemical shifts, and to external H_3_PO_4_, for ^31^P chemical shifts, respectively. Magnetic moments were determined by the Evans method^68,82,83^ by using a capillary containing 1,3,5-trimethoxybenzene in C_6_D_6_ as a reference. EPR spectra were recorded on a Bruker EMXplus EPR spectrometer with a standard X-band EMXplus resonator and an EMX premium microwave bridge, at microwave power of 2 mW, modulation frequency 100 kHz and amplitude 0.01 mT. Elemental analyses were performed on a CE-440 Elemental analyzer, or by Midwest Microlab. Gaussian 03 (revision D.02) was used for all reported calculations. The B3LYP (DFT) method was used to carry out the geometry optimizations on model compounds specified in text using the LANL2DZ basis set. The validity of the true minima was checked by the absence of negative frequencies in the energy Hessian.

### Synthesis of [{PC˙(sp^2^)P}^*t*Bu^PdNH^*p*^Tol] (2)

^*p*^TolNHK (10.2 mg, 0.071 mmol) in 1 mL of THF was slowly added to a dark-green solution of 1 (50 mg, 0.067 mmol) in 1 mL of THF at −35 °C. The resulted greenish slurry was allowed to stir at room temperature for 1 h. All volatiles were removed under reduced pressure and the residue was extracted with 6 mL of *n*-pentane, and filtered to give a dark-green solution. The volume of this *n*-pentane solution was reduced to about 0.5 mL under reduced pressure and stored at −35 °C to give compound 2 as green crystals. Yield 42 mg (87%). For 2: [{PC˙(sp^2^)P}^*t*Bu^PdNH^*p*^Tol] (2) is paramagnetic. Magnetic moment (Evans method, 298 K): *μ*_eff_ = 1.70 *μ*_B_; EPR: *g* = 2.0088. Anal. calcd for C_40_H_60_NP_2_Pd (723.28 g mol^−1^): C, 66.42; H, 8.36; N, 1.94. Found: C, 66.52; H, 8.31; N, 1.68.

### Synthesis of [{PC˙(sp^2^)P}^*t*Bu^PdNPh_2_] (3)

Ph_2_NK (19.5 mg, 0.094 mmol) and 1 (70 mg, 0.094 mmol) were mixed in 5 mL of THF and heated at 60 °C for 6 days, during which time a greenish slurry was formed. All volatiles were removed under reduced pressure and the residue was extracted with *n*-pentane (3 × 4 mL), and filtered to give a dark-green solution. The volume of the *n*-pentane solution was reduced to about 1 mL under reduced pressure and stored at −35 °C to give compound 2 as green crystals. Yield 59 mg (80%). For 3: [{PC˙(sp^2^)P}^*t*Bu^PdNPh_2_] (3) is paramagnetic. Magnetic moment (Evans method, 298 K): *μ*_eff_ = 1.54 *μ*_B_; EPR: *g* = 2.0079. Anal. calcd for C_45_H_62_NP_2_Pd (785.35 g mol^−1^): C, 68.82; H, 7.96; N, 1.78. Found: C, 68.91; H, 7.55; N, 1.40.

### Synthesis of [{PC(sp^2^)P}^*t*Bu^PdNH^*p*^Tol]^−^K^+^ (4)

KC_8_ (10.3 mg, 0.076 mmol) and 2 (55 mg, 0.076 mmol) were mixed in 3 mL of C_6_H_6_ at room temperature. The resulted dark-brown reaction mixture was stirred at room temperature for 30 min. All volatiles were removed under reduced pressure and the residue was extracted with 4 mL of diethyl ether and filtered to give a dark-brown solution. The volume of this solution was reduced to about 1.5 mL under reduced pressure and layered with 7 mL of *n*-pentane at room temperature. Compound 4 crystallized from this solution as dark-brown crystals. Yield 48 mg (83%). For 4: ^1^H NMR (500 MHz, 25 °C, THF-*d*_8_): *δ* = 7.23 (br s, 2H, Ar*H*), 6.61 (br s, 2H, Ar*H*), 6.54 (br s, 2H, Ar*H*), 6.35 (d, ^3^*J*_HH_ = 7.5 Hz, 2H, Ar*H*), 6.10 (d, ^3^*J*_HH_ = 8.0 Hz, 2H, ArH), 2.36 (m, 4H, C*H*(CH_3_)_2_), 1.98 (s, 3H, ^*p*^TolC*H*_3_), 1.33 (s, 1H, N*H*), 1.24 (dt, ^3^*J*_HH_ = 7.5 Hz, ^3^*J*_HP_ = 7.0 Hz, 12H, CH(C*H*_3_)_2_), 1.16 (dt, ^3^*J*_HH_ = 7.5 Hz, ^3^*J*_HP_ = 8.0 Hz, 12H, CH(C*H*_3_)_2_), 1.16 (s, 18H, C(C*H*_3_)_3_) ppm; ^13^C{^1^H} NMR (126 MHz, 25 °C, THF-*d*_8_): *δ* = 162.42 (s, Ar*C*), 161.98 (m, Ar*C*), 130.91 (br s, Ar*C*), 129.56 (br s, Ar*C*), 129.40 (s, Ar*C*), 128.50 (br s, Ar*C*), 119.08 (br s, Ar*C*), 115.73 (s, Ar*C*), 115.53 (br s, Ar*C*), 112.95 (s, Ar*C*), 110.35 (br s, Ar*C*), 34.03 (s, *C*(CH_3_)_3_), 32.22 (br s, C(*C*H_3_)_3_), 25.85 (br s, *C*H(CH_3_)_2_), 20.97 (s, ^*p*^Tol*C*H_3_), 19.25 (s, CH(*C*H_3_)_2_), 18.99 (s, CH(*C*H_3_)_2_) ppm. The peak for the carbenic carbon cannot be assigned in the 162–110 ppm region, but the total number of carbon peaks are correct. ^31^P{^1^H} NMR (202 MHz, 25 °C, THF-*d*_8_): *δ* = 46.36 (s) ppm. Anal. calcd for C_40_H_60_KNP_2_Pd (762.38 g mol^−1^): C, 63.02; H, 7.93; N, 1.84. Found: C, 63.72; H, 8.42; N, 1.39.

### Synthesis of [{PC(sp^2^)P}^*t*Bu^PdNPh_2_]^−^[KOEt_2_]^+^ (5)

KC_8_ (8.6 mg, 0.064 mmol) and 3 (50 mg, 0.064 mmol) were mixed in 2 mL of C_6_H_6_ at room temperature. The resulted greenish-brown reaction mixture was stirred at room temperature for 30 min. All volatiles were removed under reduced pressure and the residue was extracted with ether (3 × 5 mL) and filtered to give a greenish-brown solution. The volume of this solution was reduced to about 3 mL under reduced pressure and layered with 9 mL of *n*-pentane at room temperature. Compound 5 crystallized from this solution as greenish brown crystals. Yield 41 mg (71%). For 5: ^1^H NMR (500 MHz, 25 °C, THF-*d*_8_): *δ* = 7.35 (d, ^3^*J*_HH_ = 7.5 Hz, 4H, Ar*H*), 6.80 (t, ^3^*J*_HH_ = 7.3 Hz, 4H, Ar*H*), 6.18 (t, ^3^*J*_HH_ = 7.0 Hz, 2H, Ar*H*), 3.39 (q, ^3^*J*_HH_ = 7.0 Hz, 4H, OC*H*_2_CH_3_), 1.98 (br s, 4H, C*H*(CH_3_)_2_), 1.14 (br s, 24H, CH(C*H*_3_)_2_), 1.12 (t, ^3^*J*_HH_ = 7.0 Hz, 4H, OCH_2_C*H*_3_), 1.06 (br s, 18H, C(C*H*_3_)_3_) ppm; ^13^C{^1^H} NMR (126 MHz, 25 °C, THF-*d*_8_): *δ* = 158.82 (s, Ar*C*), 128.14 (s, Ar*C*), 121.73 (s, Ar*C*), 113.63 (s, Ar*C*), 66.47 (s, O*C*H_2_CH_3_), 31.85 (s, *C*(CH_3_)_3_), 19.04 (br s, CH(*C*H_3_)_2_), 18.19 (br s, CH(*C*H_3_)_2_), 15.85 (s, OCH_2_*C*H_3_) ppm. The peaks for the phenyl of the (PC(sp^2^)P) ligand backbone were not observed in the ^1^H and ^13^C{^1^H} NMR spectra (*vide supra*). ^31^P{^1^H} NMR (202 MHz, 25 °C, THF-*d*_8_): *δ* = 48.64 (s) ppm. Anal. calcd for C_49_H_72_KNOP_2_Pd (898.57 g mol^−1^): C, 65.50; H, 8.08; N, 1.56. Found: C, 65.61; H, 8.12; N, 1.44.

### Synthesis of [{PC(sp^2^)P}^*t*Bu^PdCH_2_Ph]^−^K^+^ (6)

PhCH_2_K (17.5 mg, 0134 mmol) in 1 mL of THF was slowly added to 1 (50 mg, 0.067 mmol) in 1 mL of THF at −35 °C. The dark-red reaction mixture was stirred at room temperature for 2 hours. Volatiles were removed under reduced pressure and the residue was extracted with 8 mL of diethyl ether and filtered to give a dark-brown solution. The volume of this solution was reduced to about 1.5 mL under reduced pressure and layered with 7 mL of *n*-pentane. Compound 6 crystallized from this solution at −35 °C as dark-brown solid. Yield 32 mg (64%). For 6: ^1^H NMR (500 MHz, 25 °C, THF-*d*_8_): *δ* = 7.20 (dt, ^3^*J*_HH_ = 9.0 Hz, ^3^*J*_HP_ = 4.5 Hz, 2H, Ar*H*), 6.98 (d, ^3^*J*_HH_ = 7.0 Hz, 2H, Ar*H*), 6.81 (t, ^3^*J*_HH_ = 7.5 Hz, 2H, Ar*H*); 6.66 (td, ^4^*J*_HH_ = 2.3 Hz, ^3^*J*_HP_ = 4.8 Hz, 2H, Ar*H*), 6.52 (dd, ^3^*J*_HH_ = 9.0 Hz, ^4^*J*_HH_ = 2.0 Hz, 2H, Ar*H*), 6.46 (t, ^3^*J*_HH_ = 7.3 Hz, 1H, Ar*H*), 2.45 (t, ^3^*J*_HP_ = 5.0 Hz, 2H, C*H*_2_Ph), 2.26 (m, 4H, C*H*(CH_3_)_2_), 1.16 (dt, ^3^*J*_HH_ = 7.0 Hz, ^3^*J*_HP_ = 6.5 Hz, 12H, CH(C*H*_3_)_2_), 1.15 (s, 18H, C(C*H*_3_)_3_), 1.14 (^3^*J*_HH_ = 6.0 Hz, ^3^*J*_HP_ = 7.5 Hz, 12H, CH(C*H*_3_)_2_) ppm; ^13^C{^1^H} NMR (126 MHz, 25 °C, THF-*d*_8_): *δ* = 161.56 (t, *J*_CP_ = 18.6 Hz, Ar*C*), 159.81 (t, *J*_CP_ = 1.3 Hz, Ar*C*), 130.35 (t, *J*_CP_ = 3.0 Hz, Ar*C*), 129.94 (s, Ar*C*), 128.43 (s, Ar*C*), 128.27 (s, Ar*C*), 127.46 (s, Ar*C*), 121.85 (t, *J*_CP_ = 19.0 Hz, Ar*C*), 118.62 (s, Ar*C*), 115.38 (t, *J*_CP_ = 10.3 Hz, Ar*C*), 34.00 (s, *C*(CH_3_)_3_), 32.27 (s, C(*C*H_3_)_3_), 26.07 (t, ^1^*J*_CP_ = 10.5 Hz, *C*H(CH_3_)_2_), 20.26 (t, ^2^*J*_CP_ = 3.2 Hz, CH(*C*H_3_)_2_), 19.04 (s, CH(*C*H_3_)_2_), 14.12 (t, ^2^*J*_CP_ = 9.0 Hz, *C*H_2_Ph) ppm; ^31^P{^1^H} NMR (202 MHz, 25 °C, THF-*d*_8_): *δ* = 48.52 (s) ppm. Anal. calcd for C_40_H_59_KP_2_Pd (747.36 g mol^−1^): C, 64.28; H, 7.96. Found: C, 64.81; H, 8.53.

### Synthesis of [{PC˙(sp^2^)P}^*t*Bu^PdCH_2_Ph] (7)

[Cp_2_Fe][BAr^F^_4_] (32 mg, 0.031 mmol) in 1 mL of THF was slowly added to 6 (23 mg, 0.031 mmol) in 1 mL of THF at −35 °C. The dark-red reaction mixture was stirred at room temperature for 30 min. All volatiles were removed under reduced pressure and the residue was extracted with 4 mL of *n*-pentane and filtered to give a red solution. The volatiles were removed under reduced pressure to give a red oily mixture of 7 and Cp_2_Fe, which are not separable due to similar solubilities in common solvents. For 7: [{PC˙(sp^2^)P}^*t*Bu^PdCH_2_Ph] (7) is paramagnetic. EPR: *g* = 2.0086.

### Oxidation of [{PC(sp^2^)P}^*t*Bu^PdNH^*p*^Tol]^−^K^+^ (4)

[Cp_2_Fe][BAr^F^_4_] (6.9 mg, 0.007 mmol) in 1 mL of diethyl ether was added to [{PC(sp^2^)P}^*t*Bu^PdNH^*p*^Tol]^−^K^+^ (4) (5 mg, 0.007 mmol) in 1 mL of diethyl ether at −35 °C. The resulted brownish-green solution was allowed to stir at room temperature for 30 min. The volatiles were removed under reduced pressure and the residue was extracted with *n*-pentane. This solution was filtered and the volatiles were removed under reduced pressure to give a green solid. The EPR spectrum measured in toluene at 298 K is identical to that of a pure sample of 2 isolated by a different method (*vide supra*).

### Oxidation of [{PC(sp^2^)P}^*t*Bu^PdNPh_2_]^−^[KOEt_2_]^+^ (5)

[Cp_2_Fe][BAr^F^_4_] (5.8 mg, 0.006 mmol) in 1 mL of diethyl ether was added to 5 (5 mg, 0.006 mmol) in 1 mL of diethyl ether at −35 °C. The resulted green solution was allowed to stir at room temperature for 30 min. The volatiles were removed under reduced pressure and the residue was extracted with *n*-pentane and filtered. The volatiles were removed under reduced pressure to give a green solid. The EPR spectrum measured in toluene at 298 K is identical to that of a pure sample of 3 isolated by a different method (*vide supra*).

### Synthesis of [{PCHP}^*t*Bu^PdCH_2_Ph] (8)

CH_3_CN (4 mg, 0.1 mmol) in 0.5 mL of THF was added to a solution of 6 (15 mg, 0.02 mmol) in 1 mL of THF at room temperature. The dark-red solution immediately turned to orange, which was stirred for another 30 min. Volatiles were removed under vacuum to give an yellow oil, which was extracted into 3 mL of pentane, filtered and removal of all volatiles under reduced pressure afforded 8 as an yellow foam. Yield 14 mg (quantitative). For 8: ^1^H NMR (500 MHz, 25 °C, C_6_D_6_): *δ* = 7.51 (d, ^3^*J*_HH_ = 7.0 Hz, 2H, Ar*H*); 7.46–7.44 (m, 4H, Ar*H*); 7.26 (t, ^3^*J*_HH_ = 7.8 Hz, 2H, Ar*H*); 7.21 (dd, ^3^*J*_HH_ = 8.5 Hz, ^4^*J*_HH_ = 2.0 Hz, 2H, Ar*H*); 6.96 (t, ^3^*J*_HH_ = 7.3 Hz, 1H, Ar*H*), 5.65 (s, 1H, C*H*_backbone_), 3.00 (t, ^3^*J*_HP_ = 5.3 Hz, 2H, C*H*_2_Ph), 2.41 (m, 2H, C*H*(CH_3_)_2_), 2.27 (m, 2H, C*H*(CH_3_)_2_), 1.28 (dt, 6H, ^3^*J*_HH_ = 8.0 Hz, ^3^*J*_HP_ = 7.5 Hz, CH(C*H*_3_)_2_), 1.27 (s, 18H, C(C*H*_3_)_3_), 1.17 (dt, ^3^*J*_HH_ = 6.5 Hz, ^3^*J*_HP_ = 6.5 Hz, 6H, CH(C*H*_3_)_2_), 1.06 (m, 12H, CH(C*H*_3_)_2_) ppm; ^13^C{^1^H} NMR (126 MHz, 25 °C, C_6_D_6_): *δ* = 157.67 (t, *J*_CP_ = 15.0 Hz, Ar*C*), 155.51 (s, Ar*C*), 146.47 (s, Ar*C*), 136.23 (t, *J*_CP_ = 17.3 Hz, Ar*C*), 128.70 (s, Ar*C*), 128.34 (s, Ar*C*), 127.90 (s, Ar*C*), 127.72 (t, *J*_CP_ = 8.4 Hz, Ar*C*), 127.00 (s, Ar*C*), 120.48 (s, Ar*C*), 56.26 (s, C*H*_backbone_), 34.36 (s, *C*(CH_3_)_3_), 31.65 (s, C(*C*H_3_)_3_), 25.62 (t, ^1^*J*_PC_ = 11.2 Hz, *C*H(CH_3_)_2_), 25.52 (t, *J*_PC_ = 9.3 Hz, *C*H(CH_3_)_2_), 19.87 (t, ^2^*J*_PC_ = 3.6 Hz, CH(*C*H_3_)_2_), 19.32 (t, ^2^*J*_PC_ = 2.5 Hz, CH(*C*H_3_)_2_), 19.17 (t, ^2^*J*_PC_ = 2.1 Hz, CH(*C*H_3_)_2_), 18.00 (s, CH(*C*H_3_)_2_), 13.22 (t, ^2^*J*_PC_ = 7.8 Hz, *C*H_2_Ph); ^31^P{^1^H} NMR (202 MHz, 25 °C, C_6_D_6_): *δ* = 45.76 (s) ppm. Anal. calcd for C_40_H_60_P_2_Pd (709.27 g mol^−1^): C, 67.74; H, 8.53. Found: C, 67.88; H, 8.62.

### Substitution reactions of carbene [{PC(sp^2^)P}^*t*Bu^Pd(PMe_3_)] with PhCH_2_K, ^*p*^TolNHK and Ph_2_NK nucleophiles

PhCH_2_K (2.8 mg, 0.022 mmol) in 1 mL of THF was added to carbene (15 mg, 0.022 mol) in 0.5 mL of THF at −35 °C. The dark-red solution was then stirred at room temperature for 24 h. Volatiles were removed under reduced pressure and the residues were dissolved in C_6_D_6_ (with 2 drops of THF-*d*_8_) and monitored by ^1^H and ^31^P{^1^H} NMR spectra. Same procedures were applied for ^*p*^TolNHK and Ph_2_NK. The reactions with PhCH_2_K and ^*p*^TolNHK showed conversion to the heterobimetallic carbene 6 and 4 in 60% and 65% conversion, respectively, based on the ^31^P{^1^H} NMR spectra. No conversion was observed for Ph_2_NK.

## Supplementary Material

SC-007-C6SC00948D-s001

SC-007-C6SC00948D-s002

SC-007-C6SC00948D-s003
