# Supplementary material for: Heterobimetallic Pd–K carbene complexes via one-electron reductions of palladium radical carbenes
Source: Chem Sci. 2016 Mar 24;7(7):4444–52. doi: 10.1039/c6sc00948d (PMC6014298; doi:10.1039/c6sc00948d)
Supplement: SC-007-C6SC00948D-s002 [file SC-007-C6SC00948D-s002.pdf]

Electronic Supplementary Material (ESI) for Chemical Science.
This journal is © The Royal Society of Chemistry 2016

Electronic Supporting Information for:

Heterobimetallic Pd-K Carbene Complexes via One-Electron Reductions of Palladium Radical Carbenes

Peng Cui,[†] *Melissa R. Hoffbauer,*[†] *Mariya Vyushkova,*[‡] *and Vlad M. Iluc*[†]*

[†]Department of Chemistry and Biochemistry, University of Notre Dame, Notre Dame, IN 46556

[‡]Notre Dame Radiation Laboratory, Notre Dame, Indiana 46556

*Corresponding author: viluc@nd.edu

1 X-ray data for compounds 2, 3, 5 and 6 **S5**

2 EPR Spectra **S6**

Figure S1. EPR spectrum of [{PC$^•$(sp^2)P}^{tBu}PdNH^{p}Tol] (**2**) ... S6
Figure S2. Experimental and simulated EPR spectrum of **2**... S7
Figure S3. EPR spectrum of [{PC$^•$(sp^2)P}^{tBu}PdNPh$_2$] (**3**) ... S8
Figure S4. Experimental and simulated EPR spectrum of **3**... S9
Figure S5. EPR spectrum of [{PC$^•$(sp^2)P}^{tBu}PdCH$_2$Ph] (**7**) ... S10
Figure S6. Experimental and simulated EPR spectrum of **7**... S11

3 DFT Results **S12**

Figure S7. Computed molecules ... S12
3.1 [{PC$^•$(sp^2)P}^{tBu}PdNH^{p}Tol] (**2**) ... S12
Table S1. Optimized coordinates for [{PC$^•$(sp^2)P}^{tBu}PdNH^{p}Tol] (**2**) ... S12
Figure S8. Overlaid structures for [{PC$^•$(sp^2)P}^{tBu}PdNH^{p}Tol] (**2**) (red: X-ray, blue: optimized) ... S15
Table S2. Selected distances (Å) and angles (°) for the optimized geometry and the crystal structure of [{PC$^•$(sp^2)P}^{tBu}PdNH^{p}Tol] (**2**) ... S16
Figure S9. Optimized geometry for [{PC$^•$(sp^2)P}^{tBu}PdNH^{p}Tol] (**2**) ... S16
3.2 [{PC$^•$(sp^2)P}^{tBu}PdNPh$_2$] (**3**) ... S17
Table S3. Optimized coordinates for [{PC$^•$(sp^2)P}^{tBu}PdNPh$_2$] (**3**) ... S17
Table S4. Selected distances (Å) and angles (°) for the optimized geometry and the crystal structure of [{PC$^•$(sp^2)P}^{tBu}PdNPh$_2$] (**3**) ... S20
Figure S10. Optimized geometry for [{PC$^•$(sp^2)P}^{tBu}PdNPh$_2$] (**3**)... S20
Figure S11. Overlaid structures for [{PC$^•$(sp^2)P}^{tBu}PdNPh$_2$] (**3**) (red: X-ray, blue: optimized) ... S21
3.3 [{PC$^•$(sp^2)P}^{Me}PdNHPh] (**2'**) ... S22
Table S5. Optimized coordinates for [{PC$^•$(sp^2)P}^{Me}PdNHPh] (**2'**)... S22
Figure S12. Optimized geometry for [{PC$^•$(sp^2)P}^{Me}PdNHPh] (**2'**) ... S23
Table S6. Selected distances (Å) and angles (°) for the optimized geometry and the crystal structure of [{PC$^•$(sp^2)P}^{Me}PdNHPh] (**2'**) ... S24
Figure S13. SOMO and spin density for [{PC$^•$(sp^2)P}^{Me}PdNHPh] (**2'**) ... S24

3.4 [{PC$^{\bullet}$(sp^2)P}^{Me}PdNPh$_2$] (**3'**) S25
Table S7. Optimized coordinates for [{PC$^{\bullet}$(sp^2)P}^{Me}PdNPh$_2$] (**3'**) S25
Table S8. Selected distances (Å) and angles (°) for the optimized geometry and the crystal structure of [{PC$^{\bullet}$(sp^2)P}^{Me}PdNPh$_2$] (**3'**) S26
Figure S14. Optimized geometry for [{PC$^{\bullet}$(sp^2)P}^{Me}PdNPh$_2$] (**3'**) S27
Figure S15. SOMO and spin density for [{PC$^{\bullet}$(sp^2)P}^{Me}PdNPh$_2$] (**3'**) S27
3.5 [{PC(sp^2)P}^{Me}PdNHPh]$^-$ (**4'**) S28
Table S9. Optimized coordinates for [{PC(sp^2)P}^{Me}PdNHPh]$^-$ (**4'**) S28
Figure S16. Optimized geometry for [{PC(sp^2)P}^{Me}PdNHPh]$^-$ (**4'**) S29
Table S10. Selected distances (Å) and angles (°) for the optimized geometry of [{PC(sp^2)P}^{Me}PdNHPh]$^-$ (**4'**) S30
Figure S17. Frontier molecular orbitals for [{PC(sp^2)P}^{Me}PdNHPh]$^-$ (**4'**) S30
3.6 [{PC(sp^2)P}^{Me}PdNPh$_2$]$^-$ (**5'**) S31
Table S11. Optimized coordinates for [{PC(sp^2)P}^{Me}PdNPh$_2$]$^-$ (**5'**) S31
Table S12. Selected distances (Å) and angles (°) for the optimized geometry of [{PC(sp^2)P}^{Me}PdNPh$_2$]$^-$ (**5'**) S32
Figure S18. Optimized geometry for [{PC(sp^2)P}^{Me}PdNPh$_2$]$^-$ (**5'**) S33
Figure S19. Frontier molecular orbitals for [{PC(sp^2)P}^{Me}PdNPh$_2$]$^-$ (**5'**) S33
3.7 [{PC(sp^2)-K(OEt$_2$)(C$_6$H$_6$)P}^{Me}PdNPh$_2$] (**5"**) S34
Table S13. Optimized coordinates for [{PC(sp^2)-K(OEt$_2$)(C$_6$H$_6$)P}^{Me}PdNPh$_2$] (**5"**) S34
Figure S20. Optimized geometry for [{PC(sp^2)-K(OEt$_2$)(C$_6$H$_6$)P}^{Me}PdNPh$_2$] (**5"**) S36
Table S14. Selected distances (Å) and angles (°) for the optimized geometry of [{PC(sp^2)-K(OEt$_2$)(C$_6$H$_6$)P}^{Me}PdNPh$_2$] (**5"**) S36
Figure S21. Frontier molecular orbitals for [{PC(sp^2)-K(OEt$_2$)(C$_6$H$_6$)P}^{Me}PdNPh$_2$] (**5"**) S37
3.8 [{PC(sp^2)P}^{Me}PdCH$_2$Ph]$^-$ (**6'**) S38
Table S15. Optimized coordinates for [{PC(sp^2)P}^{Me}PdCH$_2$Ph]$^-$ (**6'**) S38
Figure S22. Optimized geometry for [{PC(sp^2)P}^{Me}PdCH$_2$Ph]$^-$ (**6'**) S39
Table S16. Selected distances (Å) and angles (°) for the optimized geometry of [{PC(sp^2)P}^{Me}PdCH$_2$Ph]$^-$ (**6'**) S39
Figure S23. Frontier molecular orbitals for [{PC(sp^2)P}^{Me}PdCH$_2$Ph]$^-$ (**6'**) S40
3.9 [{PC(sp^2)-K(C$_6$H$_6$)P}^{Me}PdCH$_2$Ph]$_2$K$^+$ (**6"**) S41
Table S17. Optimized coordinates for [{PC(sp^2)-K(C$_6$H$_6$)P}^{Me}PdCH$_2$Ph]$_2$K$^+$ (**6"**) S41
Figure S24. Optimized geometry for [{PC(sp^2)-K(C$_6$H$_6$)P}^{Me}PdCH$_2$Ph]$_2$K$^+$ (**6"**) S44
Table S18. Selected distances (Å) and angles (°) for the optimized geometry of [{PC(sp^2)-K(C$_6$H$_6$)P}^{Me}PdCH$_2$Ph]$_2$K$^+$ (**6"**) S44
Figure S25. Frontier molecular orbitals for [{PC(sp^2)-K(C$_6$H$_6$)P}^{Me}PdCH$_2$Ph]$_2$K$^+$ (**6"**) S45
3.10 [{PC$^{\bullet}$(sp^2)P}^{Me}PdCH$_2$Ph] (**7'**) S46
Table S19. Optimized coordinates for [{PC$^{\bullet}$(sp^2)P}^{Me}PdCH$_2$Ph] (**7'**) S46
Figure S26. Optimized geometry for [{PC$^{\bullet}$(sp^2)P}^{Me}PdCH$_2$Ph] (**7'**) S47
Table S20. Selected distances (Å) and angles (°) for the optimized geometry of [{PC(sp^2)P}^{Me}PdCH$_2$Ph]$^-$ (**6'**) S48
Figure S27. SOMO and spin density for [{PC$^{\bullet}$(sp^2)P}^{Me}PdCH$_2$Ph] (**7'**) S48

4 NMR Spectra S49

4.1 NMR Spectra for $[\{PC(sp^2)P\}^{tBu}PdNH^{p}Tol]^{-}K^{+}$ (**4**) S49

Figure S28. ^1H NMR spectrum for $[\{PC(sp^2)P\}^{tBu}PdNH^{p}Tol]^{-}K^{+}$ (**4**)........ S49

Figure S29. $^{31}P\{^1H\}$ NMR spectrum for $[\{PC(sp^2)P\}^{tBu}PdNH^{p}Tol]^{-}K^{+}$ (**4**) S50

Figure S30. $^{13}C\{^1H\}$ NMR spectrum for $[\{PC(sp^2)P\}^{tBu}PdNH^{p}Tol]^{-}K^{+}$ (**4**) S51

Figure S31. ^1H-^{13}C HSQC NMR spectrum for $[\{PC(sp^2)P\}^{tBu}PdNH^{p}Tol]^{-}K^{+}$ (**4**).... S52

4.2 NMR Spectra for $[\{PC(sp^2)P\}^{tBu}PdNPh_2]^{-}[KOEt_2]^{+}$ (**5**)........ S53

Figure S32. ^1H NMR spectrum for $[\{PC(sp^2)P\}^{tBu}PdNPh_2]^{-}[KOEt_2]^{+}$ (**5**) S53

Figure S33. $^{31}P\{^1H\}$ NMR spectrum for $[\{PC(sp^2)P\}^{tBu}PdNPh_2]^{-}[KOEt_2]^{+}$ (**5**)........ S54

Figure S34. $^{13}C\{^1H\}$ NMR spectrum for $[\{PC(sp^2)P\}^{tBu}PdNPh_2]^{-}[KOEt_2]^{+}$ (**5**) S55

Figure S35. VT ^1H NMR spectra for $[\{PC(sp^2)P\}^{tBu}PdNPh_2]^{-}[KOEt_2]^{+}$ (**5**)........ S56

Figure S36. VT $^{31}P\{^1H\}$ NMR spectra for $[\{PC(sp^2)P\}^{tBu}PdNPh_2]^{-}[KOEt_2]^{+}$ (**5**)..... S57

4.3 NMR Spectra for $[\{PC(sp^2)P\}^{tBu}PdCH_2Ph]^{-}K^{+}$ (**6**) S58

Figure S37. ^1H NMR spectrum for $[\{PC(sp^2)P\}^{tBu}PdCH_2Ph]^{-}K^{+}$ (**6**) S58

Figure S38. $^{31}P\{^1H\}$ NMR spectrum for $[\{PC(sp^2)P\}^{tBu}PdCH_2Ph]^{-}K^{+}$ (**6**) S59

Figure S39. $^{13}C\{^1H\}$ NMR spectrum for $[\{PC(sp^2)P\}^{tBu}PdCH_2Ph]^{-}K^{+}$ (**6**)........ S60

Figure S40. ^1H-^{13}C HSQC NMR spectrum for $[\{PC(sp^2)P\}^{tBu}PdCH_2Ph]^{-}K^{+}$ (**6**)..... S61

4.4 NMR Spectra for $[\{PC(sp^3)HP\}^{tBu}PdCH_2Ph]$ (**8**)........ S62

Figure S41. ^1H NMR spectrum for $[\{PC(sp^3)HP\}^{tBu}PdCH_2Ph]$ (**8**) S62

Figure S42. $^{31}P\{^1H\}$ NMR spectrum for $[\{PC(sp^3)HP\}^{tBu}PdCH_2Ph]$ (**8**)........ S63

Figure S43. $^{13}C\{^1H\}$ NMR spectrum for $[\{PC(sp^3)HP\}^{tBu}PdCH_2Ph]$ (**8**) S64

5 Crystallographic tables S65

5.1 Crystal data for $[\{PC^{\bullet}(sp^2)P\}^{tBu}PdNH^{p}Tol]$ (**2**)........ S65

Figure S44. Thermal-ellipsoid representation of **2** S65

Table S21. Crystal data and structure refinement for **2** S66

Table S22. Atomic coordinates and equivalent isotropic displacement parameters (Å^2) for **2** S67

Table S23. Anisotropic displacement parameters (Å^2) for **2** S70

Table S24. Distances [Å] for **2**........ S72

Table S25. Angles [°] for **2**........ S74

5.2 Crystal data for $[\{PC^{\bullet}(sp^2)P\}^{tBu}PdNPh_2]$ (**3**) S77

Figure S45. Thermal-ellipsoid representation of **3** S77

Table S26. Crystal data and structure refinement for **3** S78

Table S27. Atomic coordinates and equivalent isotropic displacement parameters (Å^2) for **3** S79

Table S28. Anisotropic displacement parameters (Å^2) for **3** S81

Table S29. Distances [Å] for **3**........ S82

Table S30. Angles [°] for **3**........ S83

5.3 Crystal data for $[\{PC(sp^2)P\}^{tBu}PdNPh_2]^{-}[KOEt_2]^{+}$ (**5**)........ S85

Figure S46. Thermal-ellipsoid representation of **5** S85

Figure S47. Polymeric structure of **5** S85

Table S31. Crystal data and structure refinement for **5** S86

Table S32. Atomic coordinates and equivalent isotropic displacement parameters (Å^2) for **5** S87

Table S33. Anisotropic displacement parameters (Å^2) for **5** S91
Table S34. Distances [Å] for **5** S93
Table S35. Angles [°] for **5** S95
5.4 Crystal data for [{PC(sp^2)P}^{tBu}PdCH$_2$Ph]$^-$K$^+$ (**6**) S98
Figure S48. Thermal-ellipsoid representation of **6** S98
Figure S49. Polymeric structure of **6** S99
Table S36. Crystal data and structure refinement for **6** S99
Table S37. Atomic coordinates and equivalent isotropic displacement parameters (Å^2) for **6** S100
Table S38. Anisotropic displacement parameters (Å^2) for **6** S103
Table S39. Distances [Å] for **6** S105
Table S40. Angles [°] for **6** S107

1 X-ray data for compounds 2, 3, 5 and 6

X−Ray crystal structure of [{PC•(sp²)P}^{tBu}PdNH^{p}Tol] (2). Single crystals were obtained as dark-green blocks from a concentrated *n*-pentane at at −35 °C in the glovebox. Crystal and refinement data for **2**: $C_{40}H_{60}NP_2Pd$; M_r = 723.23; Triclinic; space group $P\bar{1}$; *a* = 9.9475(7) Å; *b* = 13.7704(10) Å; *c* = 14.6585(10) Å; α = 98.9950(19)°; β = 100.8508(18)°; γ = 97.3666(18)°; V = 1921.9(2) $Å^3$; Z = 2; T = 120(2) K; λ = 0.71073 Å; μ = 0.593 mm^{-1}; d_{calc} = 1.250 $g{\cdot}cm^{-3}$; 47002 reflections collected; 6761 unique (R_{int} = 0.0350); giving R_1 = 0.0253, wR_2 = 0.0606 for 6096 data with [I>2σ(I)] and R_1 = 0.0302, wR_2 = 0.0624 for all 6761 data. Residual electron density ($e^{-}{\cdot}Å^{-3}$) max/min: 0.818/−0.527.

X−Ray crystal structure of [{PC•(sp²)P}^{tBu}PdNPh$_2$] (3). Single crystals were obtained as dark-green blocks from a concentrated *n*-pentane at at −35 °C in the glovebox. Crystal and refinement data for **3**: $C_{45}H_{62}NP_2Pd$; M_r = 785.30; Monoclinic; space group C2/c; *a* = 20.2994(14) Å; *b* = 17.5268(14) Å; *c* = 15.9561(16) Å; α = 90°; β = 123.775(3)°; γ = 90°; V = 4718.8(7) $Å^3$; Z = 4; T = 120(2) K; λ = 0.71073 Å; μ = 0.488 mm^{-1}; d_{calc} = 1.105 $g{\cdot}cm^{-3}$; 50505 reflections collected; 4154 unique (R_{int} = 0.0474); giving R_1 = 0.0246, wR_2 = 0.0587 for 3778 data with [I>2σ(I)] and R_1 = 0.0300, wR_2 = 0.0604 for all 4154 data. Residual electron density ($e^{-}{\cdot}Å^{-3}$) max/min: 0.481/−0.418.

X−Ray crystal structure of [{PC(sp²)P}^{tBu}PdNPh$_2$]$^-$[KOEt$_2$]$^+$ (5). Single crystals were obtained as greenish-brown blocks from a concentrated solution of diethyl ether layered with *n*-pentane at −35 °C in the glovebox. Crystal and refinement data for **5**: $C_{49}H_{72}KNOP_2Pd$; M_r = 898.52; Monoclinic; space group $P2_1/n$; *a* = 10.2409(9) Å; *b* = 18.1690(15) Å; *c* = 25.743(2) Å; α = 90°; β = 90.553(3)°; γ = 90°; V = 4789.7(7) $Å^3$; Z = 4; T = 120(2) K; λ = 0.71073 Å; μ = 0.575 mm^{-1}; d_{calc} = 1.246 $g{\cdot}cm^{-3}$; 75438 reflections collected; 8436 unique (R_{int} = 0.0384); giving R_1 = 0.0252, wR_2 = 0.0563 for 7432 data with [I>2σ(I)] and R_1 = 0.0318, wR_2 = 0.0582 for all 8436 data. Residual electron density ($e^{-}{\cdot}Å^{-3}$) max/min: 0.420/−0.279.

X−Ray crystal structure of [{PC(sp²)P}^{tBu}PdCH$_2$Ph]$^-$K$^+$ (6). Single crystals were obtained as dark-brown blocks by slow evaporation of diethyl ether solution at room temperature in the glovebox. Crystal and refinement data for **6**: $C_{40}H_{59}KP_2Pd$; M_r = 747.31; Monoclinic; space group C2/c; *a* = 26.989(3) Å; *b* = 12.0603(10) Å; *c* = 23.568(2) Å; α = 90°; β = 96.879(4)°; γ = 90°; V = 7616.1(12) $Å^3$; Z = 8; T = 120(2) K; λ = 0.71073 Å; μ = 0.707 mm^{-1}; d_{calc} = 1.303 $g{\cdot}cm^{-3}$; 90845 reflections collected; 6712 unique (R_{int} = 0.0540); giving R_1 = 0.0267, wR_2 = 0.0661 for 5799 data with [I>2σ(I)] and R_1 = 0.0349, wR_2 = 0.0692 for all 6712 data. Residual electron density ($e^{-}{\cdot}Å^{-3}$) max/min: 0.917/−0.478.

2 EPR Spectra

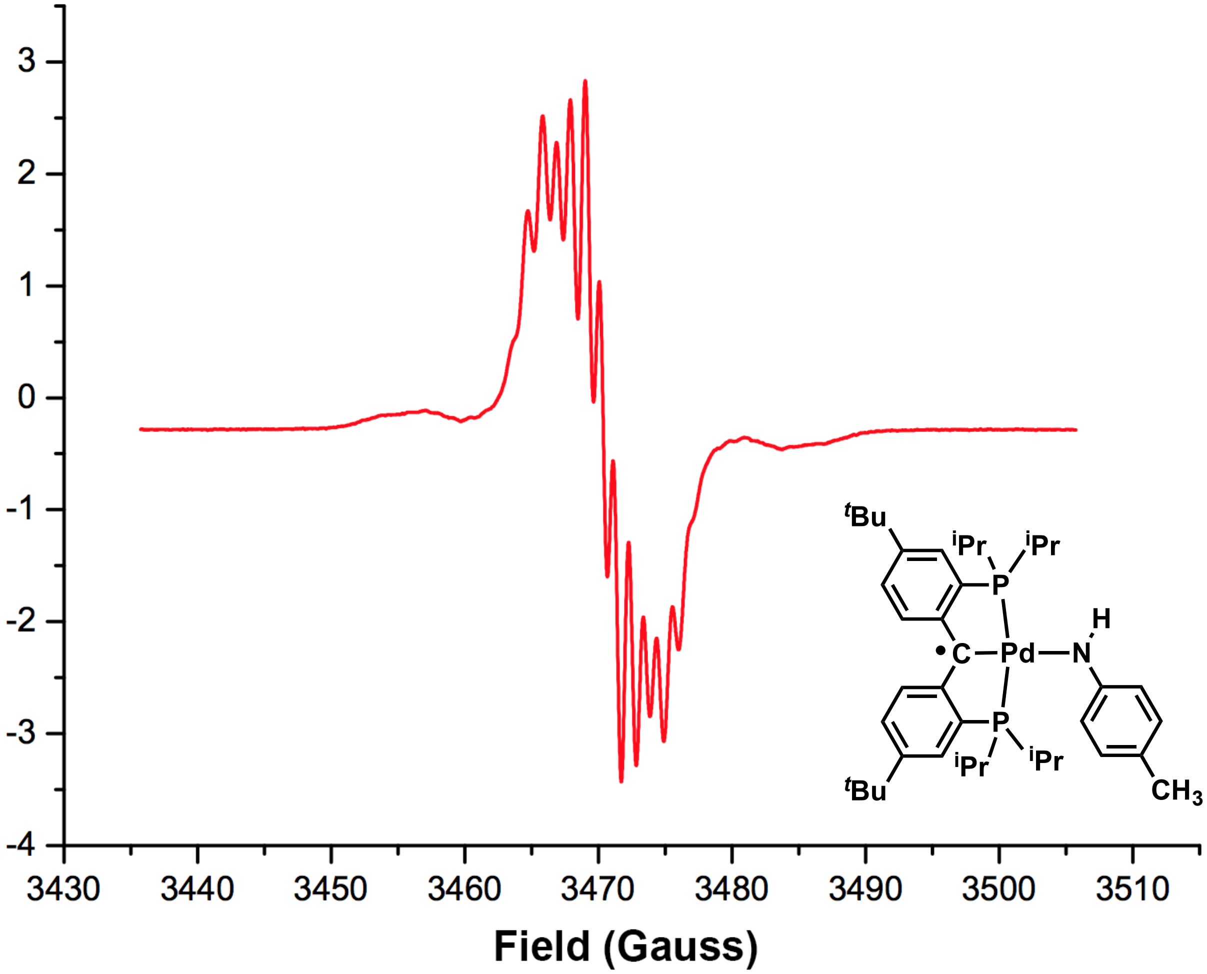

Figure S1. EPR spectrum of [{PC•(sp^2)P}^{tBu}PdNH^{p}Tol] (**2**) (1 mM solution in toluene, 298 K).

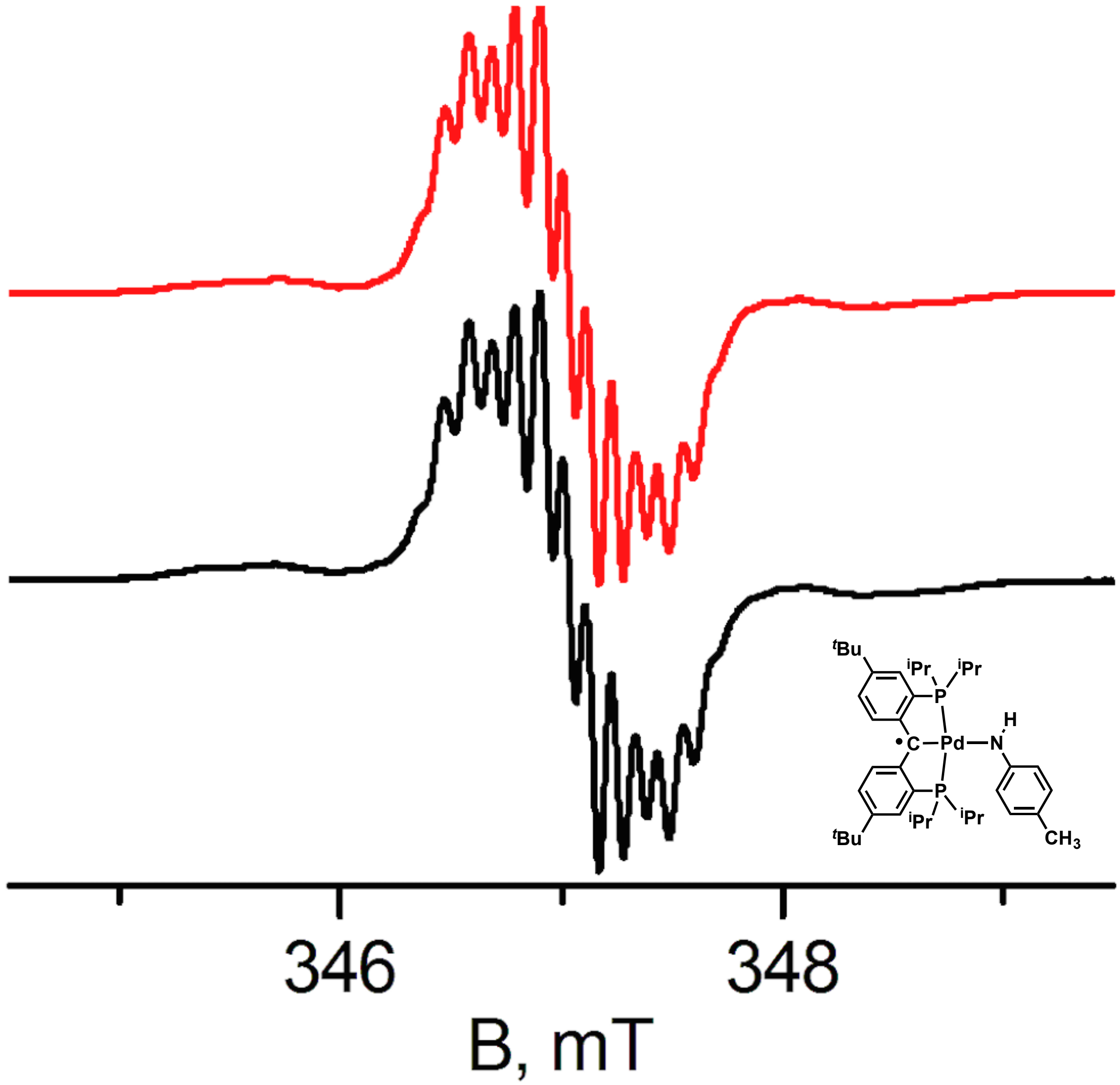

Figure S2. Experimental (black) and simulated (red) X-band EPR spectra of 10^{-4}M toluene solution of [{PC•(sp^2)P}^{tBu}PdNH^{p}Tol] (**2**). Simulation parameters: a_1(2H) = 0.32 mT, a_2(2H) = 0.13 mT, a_3(2H) = 0.11 mT, a(N) = 0.09 mT, a(^{105}Pd) = 0.47 mT, g = 2.0088, 22.33% contribution from the ^{105}Pd species.

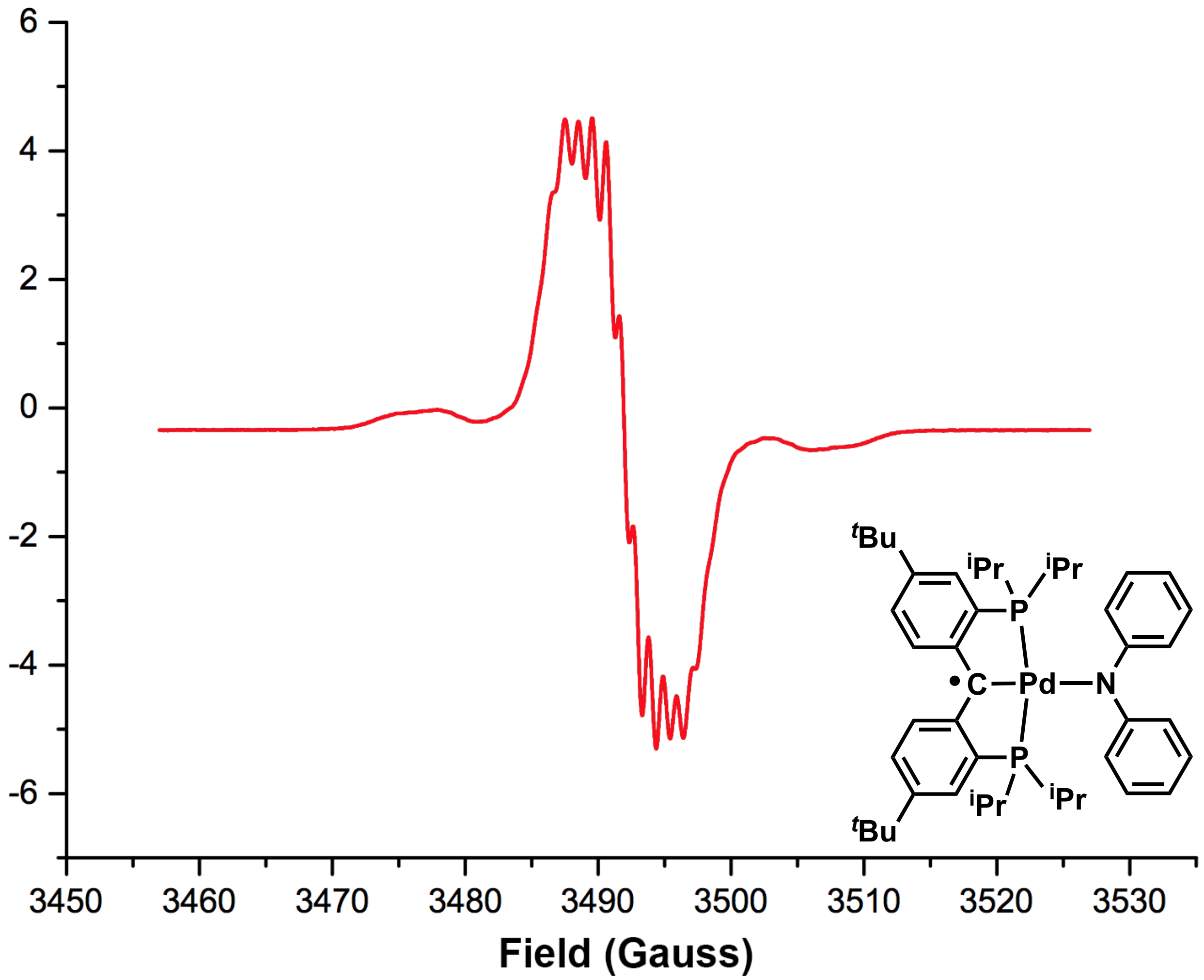

Figure S3. EPR spectrum of [{PC•(sp^2)P}^{tBu}PdNPh$_2$] (**3**) (1 mM solution in toluene, 298 K).

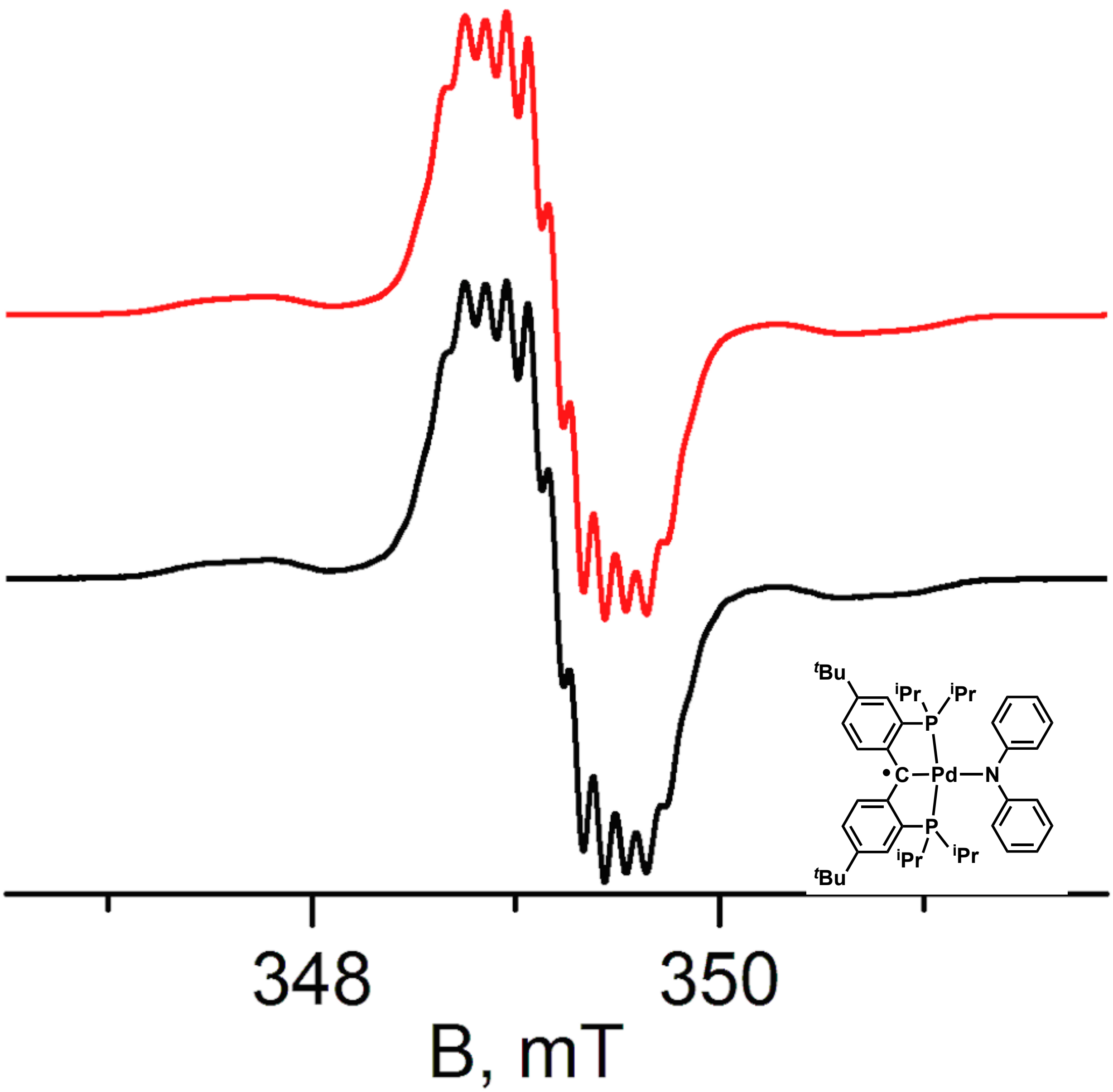

Figure S4. Experimental (black) and simulated (red) X-band EPR spectra of 10^{-4}M toluene solution of [{PC$^{\bullet}$(sp^2)P}^{tBu}PdNPh$_2$] (**3**). Simulation parameters: a_1(2H) = 0.31 mT, a_2(2H) = 0.13 mT, a_3(2H) = 0.10 mT, a(N) = 0.09 mT, a(^{105}Pd) = 0.51 mT, g = 2.0079, 22.33% contribution from the ^{105}Pd species.

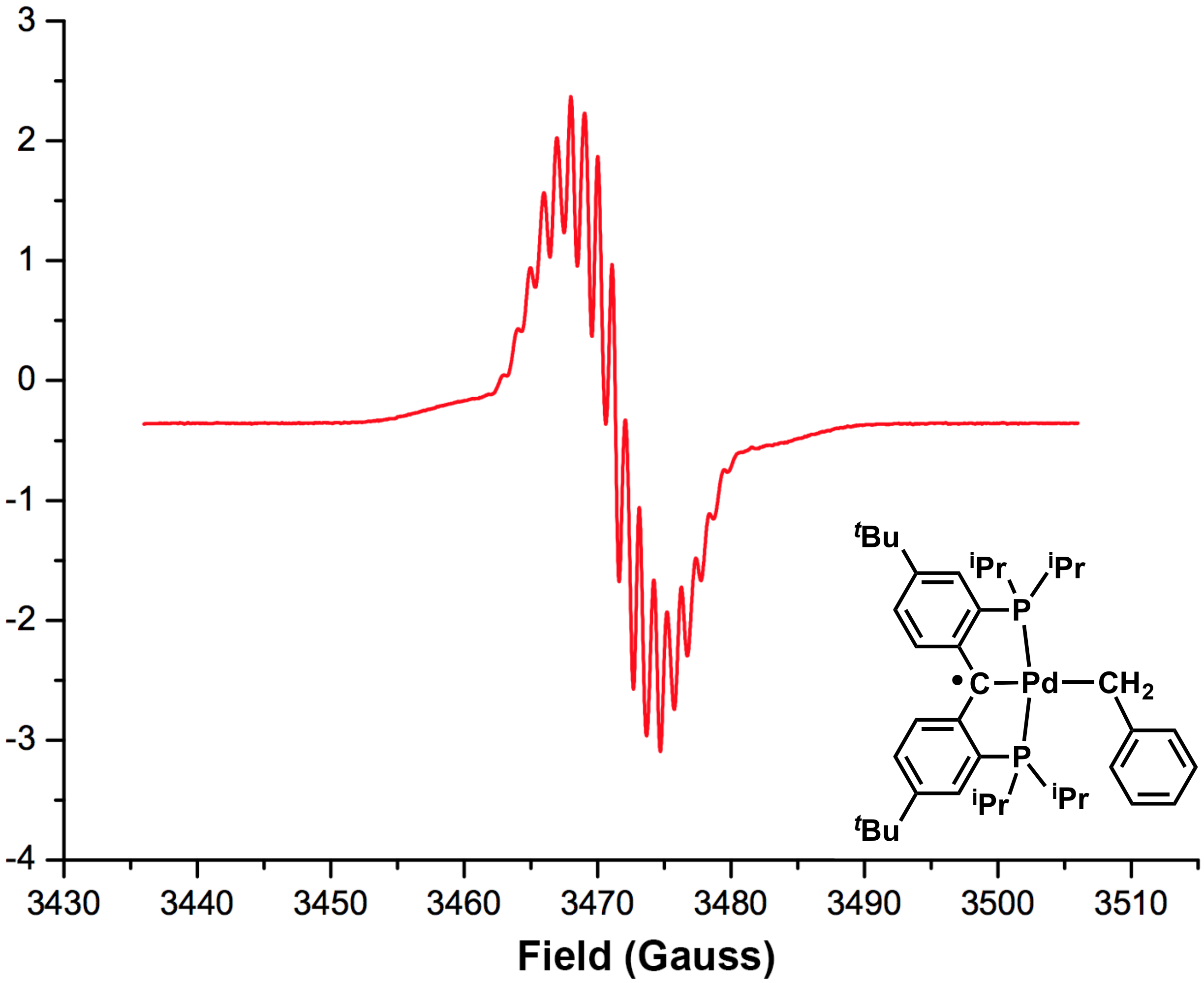

Figure S5. EPR spectrum of [{PC$^{\bullet}$(sp^2)P}^{tBu}PdCH$_2$Ph] (**7**) (1 mM solution in toluene, 298 K).

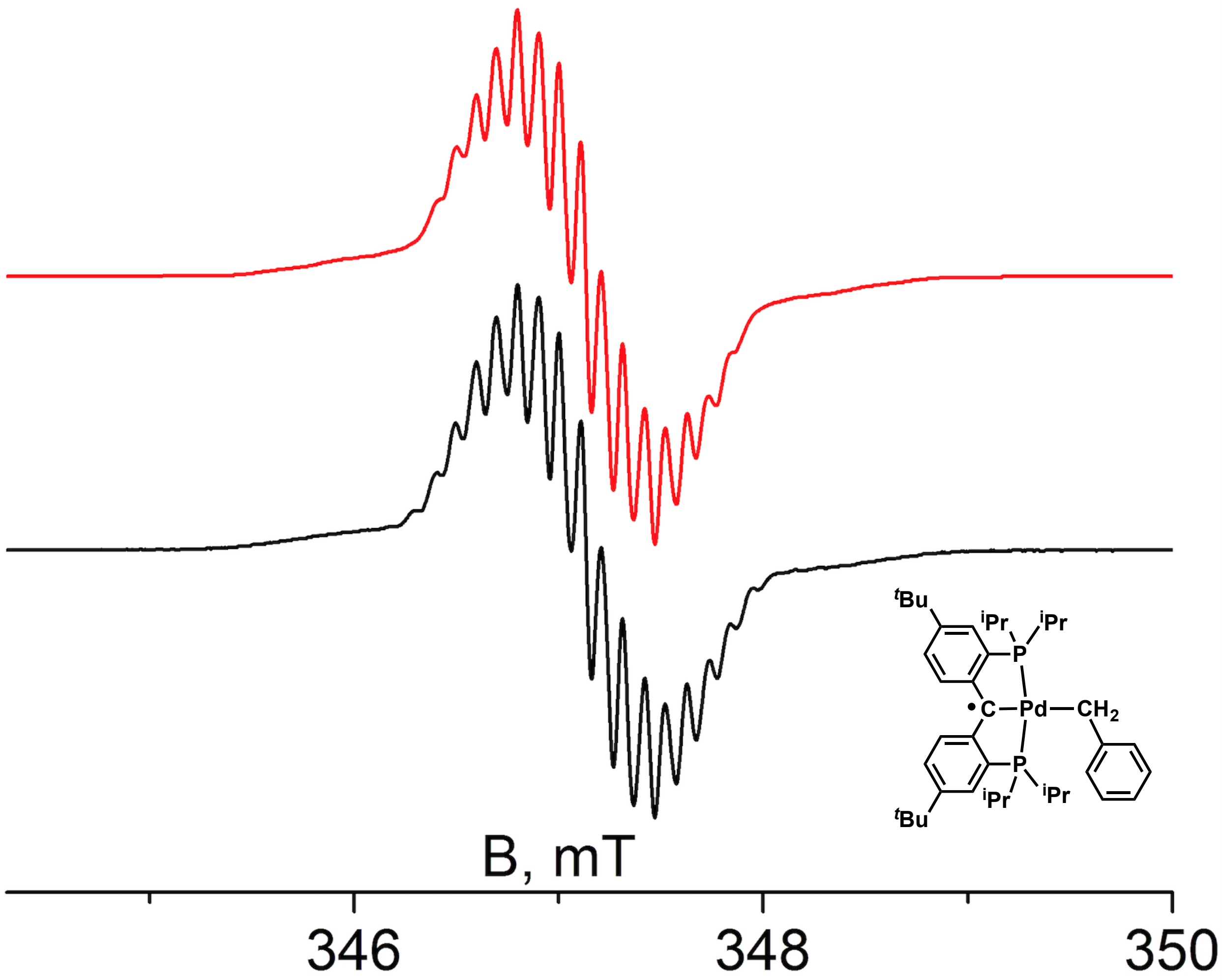

Figure S6. Experimental (black) and simulated (red) X-band EPR spectra of 10^{-4}M toluene solution of [{PC$^{\bullet}$(sp^2)P}^{tBu}PdCH$_2$Ph] (**7**). Simulation parameters: a_1(2H) = 0.32 mT, a_2(2H) = 0.19 mT, a_3(2H) = 0.12 mT, a_4(2H) = 0.08 mT, a(^{105}Pd) = 0.33 mT, g = 2.0086, 22.33% contribution from the ^{105}Pd species.

3 DFT Results

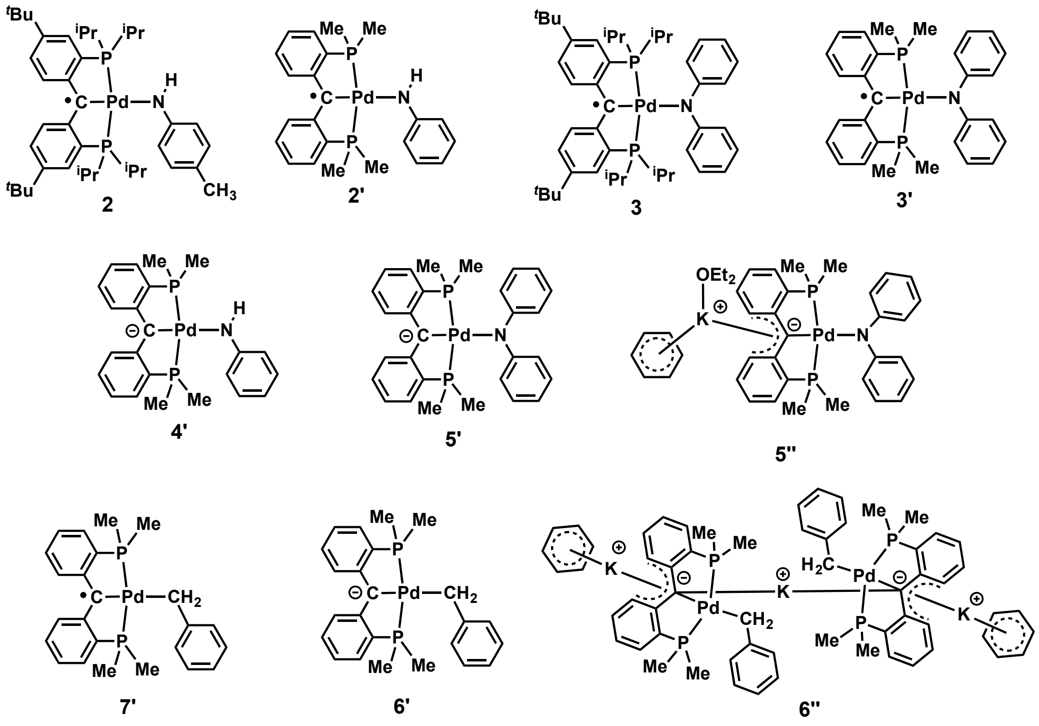

Figure S7. Computed molecules.

3.1 [{PC$^\bullet$(sp^2)P}^{tBu}PdNH^{p}Tol] (2)

Table S1. Optimized coordinates for [{PC$^\bullet$(sp^2)P}^{tBu}PdNH^{p}Tol] (**2**).

atom	x	y	z
C	−1.535225	5.049549	0.877464
C	−1.077807	3.810869	0.314745
C	−1.109118	3.720263	−1.111923
C	−1.574046	4.783029	−1.904850
C	−2.027553	5.997024	−1.343648
C	−1.991721	6.098823	0.068320
N	−0.627874	2.765001	1.096325
Pd	−0.152331	0.808087	0.510697
C	0.348286	−1.123105	0.003227
C	1.743297	−1.423738	−0.326165
C	2.785835	−0.539408	0.114803
C	4.130005	−0.773671	−0.196115
C	4.539767	−1.877970	−0.986373

Continued on next page

Table S1. – continued from previous page

atom	x	y	x
C	3.515221	−2.729857	−1.460982
C	2.165868	−2.516989	−1.144322
P	2.163552	0.956563	1.050297
C	2.673042	0.734456	2.892311
C	4.199642	0.665985	3.114801
C	6.035767	−2.084045	−1.311763
C	6.578724	−0.845062	−2.084982
C	−2.512759	7.149255	−2.208016
P	−2.342399	0.103165	−0.152331
C	−3.811275	0.455477	1.023987
C	−4.285940	1.920316	0.893443
C	−2.078444	−1.750860	−0.078395
C	−0.699328	−2.144771	0.004792
C	−0.464342	−3.545098	0.171536
C	−1.512853	−4.474668	0.213802
C	−2.866186	−4.082964	0.089824
C	−3.113197	−2.693006	−0.050932
C	−4.044748	−5.081117	0.126676
C	−3.568480	−6.545329	0.298902
C	−2.975778	0.513615	−1.921610
C	−4.339891	−0.124311	−2.262762
C	3.174733	2.446947	0.404491
C	2.856824	2.687170	−1.088160
C	−4.987409	−4.735238	1.317906
C	−4.846907	−4.985114	−1.205531
C	6.278856	−3.340961	−2.184484
C	6.837997	−2.251282	0.013206
C	−1.901928	0.125468	−2.961314
C	−3.403718	0.122594	2.476253
C	2.882063	3.710118	1.246207
C	1.959798	−0.501637	3.482767
H	−4.142172	−2.347144	−0.117547
H	−1.263149	−5.520845	0.360904
H	0.550998	−3.898299	0.319000
H	−5.393224	−3.719625	1.234241
H	−4.450564	−4.805162	2.272767
H	−5.835525	−5.433063	1.348531
H	−0.611736	2.975361	2.095065
H	−5.245145	−3.975801	−1.367452
H	−5.695841	−5.682332	−1.191327
H	−4.209721	−5.236906	−2.063105
H	−4.436743	−7.216616	0.311547

Continued on next page

Table S1. – continued from previous page			
**atom**	**x**	**y**	**x**
H	−3.024962	−6.687713	1.242008
H	−2.916211	−6.860466	−0.525945
H	4.883027	−0.074113	0.159425
H	3.763698	−3.569485	−2.102739
H	1.418846	−3.175434	−1.575503
H	5.770263	−3.267340	−3.154520
H	5.936346	−4.255934	−1.683828
H	7.353105	−3.451160	−2.381100
H	6.734778	−1.374249	0.664352
H	7.906817	−2.383890	−0.203276
H	6.489014	−3.128699	0.572875
H	6.035980	−0.705281	−3.028643
H	7.644185	−0.978279	−2.317447
H	6.476793	0.076871	−1.499354
H	−2.580655	0.771172	2.800336
H	−3.083946	−0.920845	2.584549
H	−4.257201	0.290174	3.147675
H	−3.076335	1.606819	−1.909615
H	−4.626482	−0.218453	0.721577
H	−2.219681	0.451303	−3.961188
H	−1.754492	−0.961834	−2.989312
H	−0.935102	0.591588	−2.739885
H	−4.662256	2.153222	−0.110260
H	−3.471368	2.616902	1.116909
H	−5.102458	2.104089	1.605457
H	−5.125872	0.156490	−1.551172
H	−4.269260	−1.218696	−2.292918
H	−4.660834	0.213097	−3.258200
H	4.236413	2.176412	0.505130
H	0.873357	−0.441092	3.350414
H	2.176320	−0.579192	4.557144
H	2.306816	−1.423777	2.999336
H	4.719660	1.552373	2.731084
H	4.627449	−0.223756	2.636735
H	4.411311	0.597507	4.190927
H	3.437964	4.562362	0.832603
H	3.183917	3.595505	2.295621
H	1.813089	3.949570	1.211816
H	1.813455	2.997561	−1.214998
H	3.032422	1.790458	−1.694978
H	3.497739	3.491308	−1.475131
H	−0.742127	2.809153	−1.580926

Continued on next page

Table S1. – continued from previous page

atom	x	y	x
H	−1.573893	4.667213	−2.989306
H	−2.330600	7.020210	0.544033
H	−1.528345	5.167742	1.961924
H	−2.572425	6.854120	−3.263342
H	−1.840189	8.018657	−2.148883
H	−3.511830	7.495392	−1.904919
H	2.279189	1.635352	3.384223

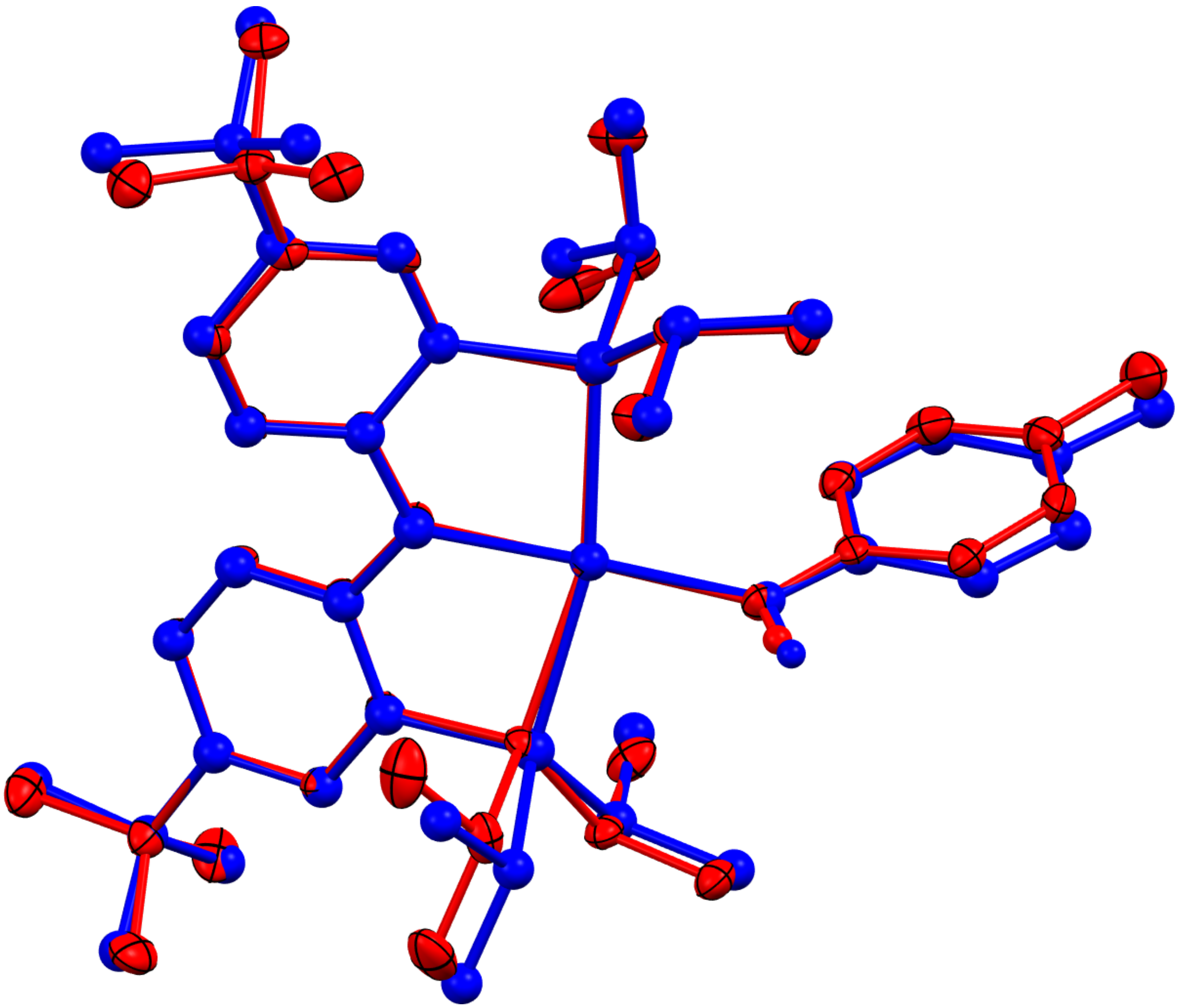

Figure S8. Overlaid structures for [{PC$^{\bullet}$(sp^2)P}^{tBu}PdNH^{p}Tol] (**2**) (red: X-ray, blue: optimized).

Table S2. Selected distances (Å) and angles (°) for the optimized geometry and the crystal structure of [{PC$^{\bullet}$(sp^2)P}^{tBu}PdNH^{p}Tol] (**2**).

**Distance**	**Calcd.**	**X-Ray**	**Angle**	**Calcd.**	**X-Ray**
Pd – N	2.097	2.0787(18)	P(1) – Pd – P(2)	165.97	163.689(19)
Pd – C	2.059	2.019(2)	C – Pd – N	177.88	178.97(9)
Pd – P(1)	2.394	2.2983(5)	C – Pd – P(1)	82.99	82.15(6)
Pd – P(2)	2.383	2.2841(5)	C – Pd – P(2)	82.99	81.80(6)
N – C(51)	1.381	1.354(3)	N – Pd – P(1)	98.32	98.20(5)
C – C(11)	1.463	1.451(3)	N – Pd – P(2)	95.68	97.79(5)
C – C(21)	1.465	1.457(3)	Pd – N – C(51)	128.50	126.94(15)

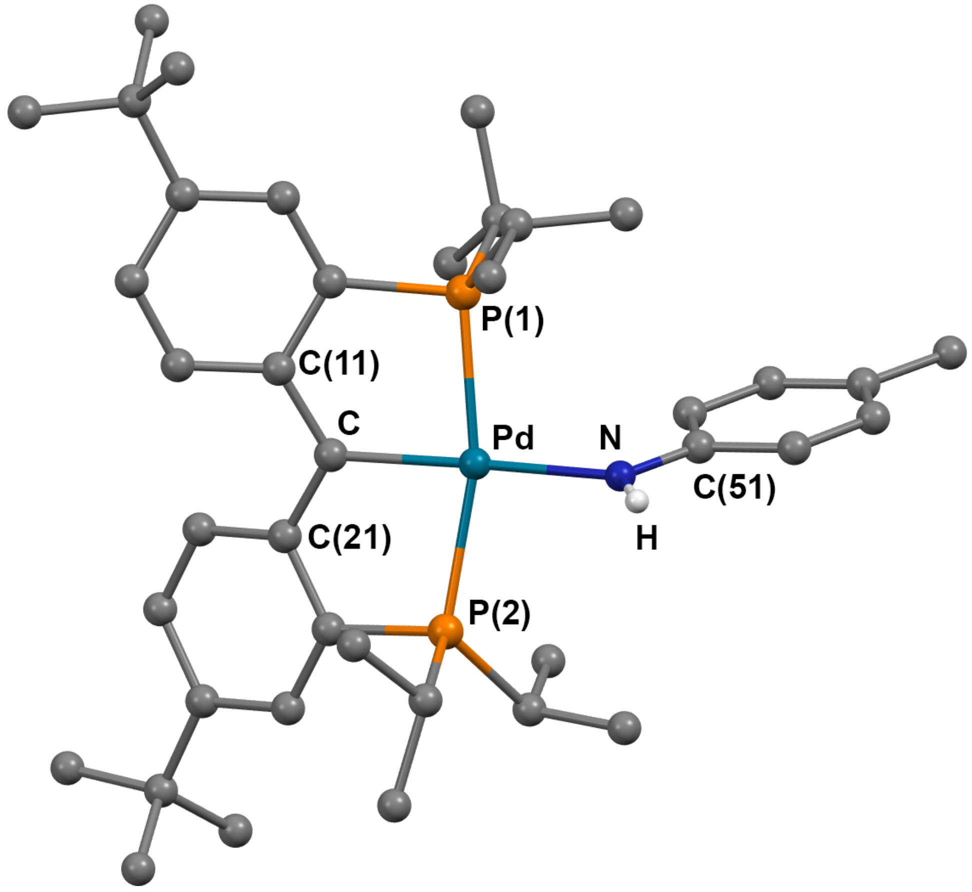

Figure S9. Optimized geometry for [{PC$^{\bullet}$(sp^2)P}^{tBu}PdNH^{p}Tol] (**2**).

3.2 [{PC$^{\bullet}$(sp^2)P}^{tBu}PdNPh$_2$] (3)

Table S3. Optimized coordinates for [{PC$^{\bullet}$(sp^2)P}^{tBu}PdNPh$_2$] (**3**).

atom	x	y	z
C	0.006383	−2.885191	2.442283
C	0.249803	−3.557902	1.207922
C	0.828532	−4.863536	1.297817
C	1.121071	−5.448114	2.539367
C	0.860531	−4.769425	3.748525
C	0.304563	−3.476196	3.679741
N	0.001932	−2.887208	0.000046
C	−0.244723	−3.558408	−1.207800
C	−0.821324	−4.864990	−1.297667
C	−1.112616	−5.450201	−2.539210
C	−0.852829	−4.771272	−3.748395
C	−0.298868	−3.477179	−3.679647
C	−0.001956	−2.885526	−2.442194
Pd	0.000398	−0.733781	−0.000051
P	−2.320152	−0.414234	−0.565819
C	−3.726187	−1.365386	0.325857
C	−3.804024	−2.826724	−0.167499
P	2.320480	−0.411129	0.565835
C	3.727755	−1.360493	−0.325781
C	3.807542	−2.821674	0.167739
C	2.503971	1.368132	0.018355
C	1.260229	2.033063	−0.221988
C	1.356737	3.363316	−0.744956
C	2.591267	3.980517	−0.961298
C	3.820621	3.332050	−0.672223
C	3.737308	2.008086	−0.185481
C	−0.000970	1.325591	−0.000045
C	−1.263101	2.031393	0.221894
C	−2.505970	1.364796	−0.018372
C	−3.740150	2.003120	0.185482
C	−3.825196	3.326992	0.672179
C	−2.596693	3.977115	0.961153
C	−1.361354	3.361545	0.744794
C	5.158774	4.063875	−0.915018
C	6.385937	3.194148	−0.543485
C	−5.164302	4.057100	0.914879
C	−6.390321	3.185776	0.543322
C	2.777081	−0.464165	2.436751
C	4.257074	−0.136085	2.731147

Continued on next page

Table S3. – continued from previous page

atom	x	y	x
C	5.201672	5.359192	−0.050715
C	5.275837	4.448582	−2.420342
C	1.834440	0.468283	3.228408
C	3.554087	−1.293221	−1.858030
C	−2.776813	−0.467865	−2.436704
C	−4.257336	−0.142046	−2.730932
C	−5.208801	5.352311	0.050502
C	−5.281946	4.441742	2.420174
C	−1.835705	0.466122	−3.228369
C	−3.552719	−1.297697	1.858105
H	4.652281	1.460682	0.016781
H	2.598448	4.985741	−1.377688
H	0.450248	3.890918	−1.023316
H	4.370492	6.033235	−0.290323
H	6.139003	5.904324	−0.227136
H	5.140590	5.117530	1.018379
H	5.263329	3.552540	−3.054273
H	6.215889	4.986703	−2.603635
H	4.450636	5.096534	−2.739219
H	6.435016	2.278881	−1.147986
H	6.377606	2.909215	0.516922
H	7.307808	3.760842	−0.726761
H	2.566162	−1.501822	2.726860
H	4.655890	−0.836985	−0.053994
H	2.008597	1.520301	2.968237
H	2.018243	0.349128	4.304890
H	0.781090	0.240663	3.032404
H	4.949943	−0.826053	2.235060
H	4.435434	−0.212694	3.812775
H	4.505733	0.888690	2.427929
H	2.664609	−1.849152	−2.172990
H	4.425398	−1.752176	−2.345434
H	3.468283	−0.261348	−2.219357
H	2.882529	−3.361318	−0.057856
H	3.982093	−2.892811	1.248171
H	4.635518	−3.334505	−0.340640
H	−1.064550	−5.407503	−0.389449
H	−1.556643	−6.444438	−2.561265
H	−1.075566	−5.233803	−4.706583
H	−0.088336	−2.925952	−4.595143
H	0.423431	−1.884954	−2.406164
H	−4.654406	1.454502	−0.016735

Continued on next page

Table S3. – continued from previous page

atom	x	y	x
H	−2.605185	4.982368	1.377451
H	−0.455555	3.890374	1.023070
H	−4.378469	6.027404	0.290099
H	−6.146820	5.896277	0.226868
H	−5.147383	5.110667	−1.018578
H	−5.268293	3.545760	3.054165
H	−6.222713	4.978642	2.603383
H	−4.457618	5.090803	2.739054
H	−6.438238	2.270469	1.147853
H	−6.381566	2.900812	−0.517075
H	−7.312933	3.751285	0.726530
H	−2.564331	−1.505180	−2.726899
H	−4.655004	−0.843139	0.053965
H	−2.011521	1.517846	−2.968125
H	−2.019390	0.346736	−4.304845
H	−0.781982	0.240170	−3.032445
H	−4.949084	−0.833138	−2.234845
H	−4.435679	−0.218826	−3.812551
H	−4.507551	0.882312	−2.427591
H	−2.662594	−1.852507	2.173212
H	−4.423509	−1.757648	2.345502
H	−3.468201	−0.265671	2.219296
H	−2.878316	−3.365119	0.058214
H	−3.978420	−2.898203	−1.247934
H	−4.631355	−3.340584	0.340893
H	1.072400	−5.405776	0.389607
H	1.566676	−6.441643	2.561444
H	1.084261	−5.231465	4.706718
H	0.093463	−2.925147	4.595213
H	−0.420476	−1.885248	2.406214

Table S4. Selected distances (Å) and angles (°) for the optimized geometry and the crystal structure of [{PC•(sp^2)P}^{tBu}PdNPh$_2$] (**3**).

Distance	Calcd.	X-Ray	Angle	Calcd.	X-Ray
Pd – N	2.153	2.149(2)	P – Pd – P#1	164.69	163.80(2)
Pd – C	2.059	2.024(2)	C – Pd – N	180.00	180.0
Pd – P	2.410	2.3021(5)	C – Pd – P	82.34	81.900(12)
Pd – P#1	2.410	2.3021(5)	C – Pd – P#1	82.34	81.900(12)
N – C(31)	1.404	1.357(2)	N – Pd – P	97.66	98.100(12)
N – C(31)#1	1.404	1.357(2)	N – Pd – P#1	97.65	98.100(12)
C – C(11)	1.463	1.444(2)	Pd – N – C(31)	118.56	117.94(12)
C – C(11)#1	1.463	1.444(2)	Pd – N – C(31)#1	118.55	117.94(12)

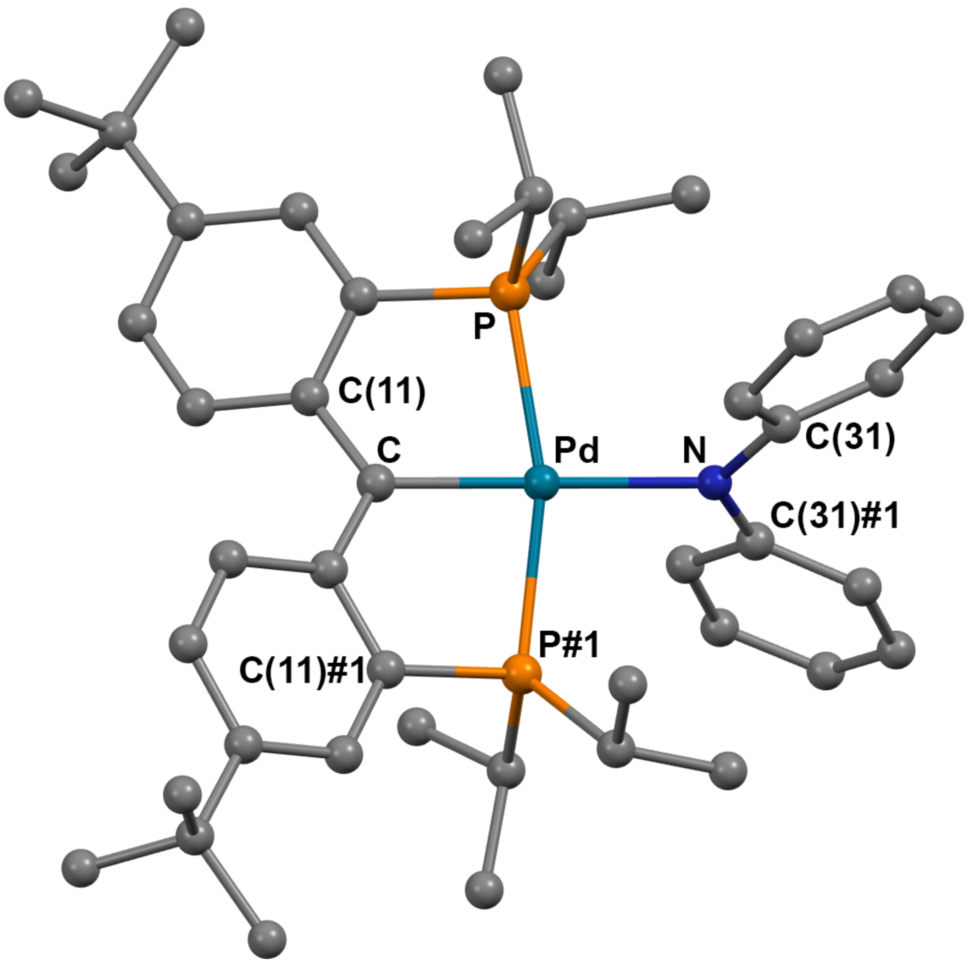

Figure S10. Optimized geometry for [{PC•(sp^2)P}^{tBu}PdNPh$_2$] (**3**).

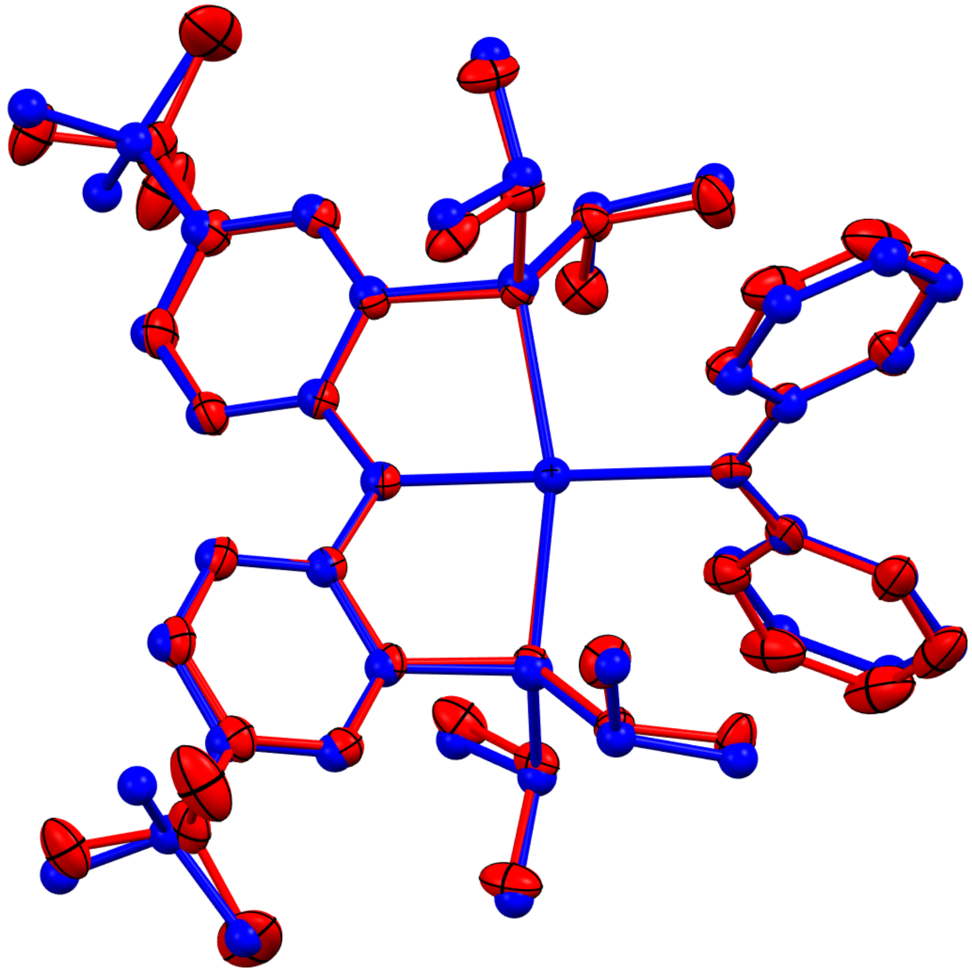

Figure S11. Overlaid structures for [{PC$^{\bullet}$(sp^2)P}^{tBu}PdNPh$_2$] (**3**) (red: X-ray, blue: optimized).

3.3 [{PC•(sp^2)P}^{Me}PdNHPh] (2')

Table S5. Optimized coordinates for [{PC•(sp^2)P}^{Me}PdNHPh] (**2'**).

atom	x	y	z
C	−4.652272	−0.863966	−0.857068
C	−3.372795	−0.799864	−0.210450
C	−3.378490	−0.549231	1.199792
C	−4.578858	−0.366195	1.904055
C	−5.828158	−0.425331	1.249759
C	−5.843522	−0.682758	−0.138846
N	−2.186990	−0.983904	−0.896133
Pd	−0.287925	−0.299570	−0.361140
C	1.618161	0.363918	0.055455
C	2.631644	−0.612466	0.465453
C	2.426267	−2.005643	0.179662
C	3.360790	−2.983358	0.547841
C	4.527453	−2.626618	1.254623
C	4.729635	−1.272820	1.599019
C	3.809841	−0.287900	1.214510
P	0.796411	−2.382630	−0.652267
C	1.213413	−3.066740	−2.359020
H	1.853539	−3.952548	−2.274645
H	5.246395	−3.386164	1.550333
P	−0.866929	1.961545	0.086571
C	−2.008676	2.842756	−1.117560
H	−2.993250	2.367810	−1.067501
C	0.824860	2.733778	−0.112079
C	1.908666	1.790143	−0.114946
C	3.214060	2.325279	−0.368378
C	3.420504	3.698772	−0.555798
C	2.342140	4.607107	−0.495562
C	1.040914	4.108216	−0.279123
H	2.507293	5.672159	−0.636169
C	−1.489441	2.530524	1.771573
H	−1.481979	3.625212	1.828713
C	0.063691	−3.860434	0.246925
H	−0.013890	−3.622355	1.311471
H	−0.837114	2.121890	2.548640
H	−1.612674	2.733310	−2.131149
H	−0.943261	−4.037006	−0.142995
H	1.734696	−2.295989	−2.933616
H	0.200915	4.800658	−0.261561
H	4.423889	4.065209	−0.762724

Continued on next page

Table S5. – continued from previous page

atom	x	y	x
H	4.056394	1.648626	−0.466041
H	−2.319464	−1.202101	−1.885156
H	3.182050	−4.030194	0.307554
H	5.603939	−0.989145	2.181256
H	3.971218	0.736705	1.532685
H	−2.505444	2.153543	1.919589
H	−2.102658	3.906989	−0.873087
H	0.678940	−4.757793	0.114852
H	−2.425267	−0.544449	1.725713
H	−4.540755	−0.190881	2.979058
H	−6.794705	−0.740407	−0.667178
H	−4.689448	−1.058790	−1.929483
H	0.287458	−3.335438	−2.878290
H	−6.754538	−0.288028	1.801521

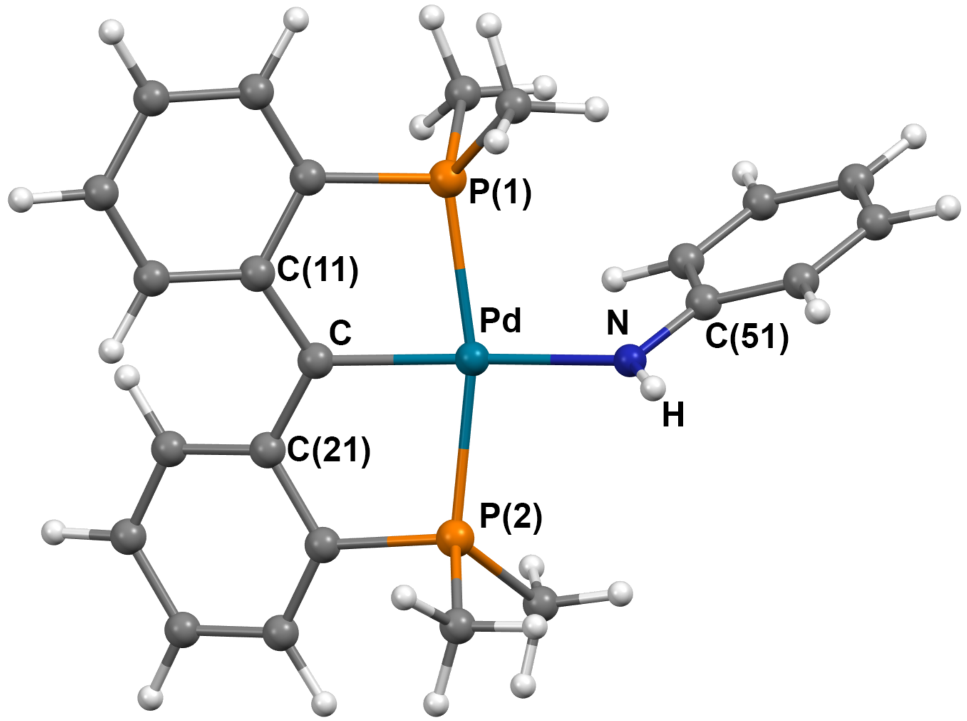

Figure S12. Optimized geometry for [{PC•(sp^2)P}^{Me}PdNHPh] (**2'**).

Table S6. Selected distances (Å) and angles (°) for the optimized geometry and the crystal structure of [{PC$^{\bullet}$(sp^2)P}^{Me}PdNHPh] (**2'**).

**Distance**	**Calcd.**	**X-Ray**	**Angle**	**Calcd.**	**X-Ray**
Pd – N	2.088	2.0787(18)	P(1) – Pd – P(2)	166.48	163.689(19)
Pd – C	2.061	2.019(2)	C – Pd – N	176.76	178.97(9)
Pd – P(1)	2.377	2.2983(5)	C – Pd – P(1)	83.16	82.15(6)
Pd – P(2)	2.366	2.2841(5)	C – Pd – P(2)	83.36	81.80(6)
N – C(51)	1.382	1.354(3)	N – Pd – P(1)	97.96	98.20(5)
C – C(11)	1.465	1.451(3)	N – Pd – P(2)	95.55	97.79(5)
C – C(21)	1.466	1.457(3)	Pd – N – C(51)	127.55	126.94(15)

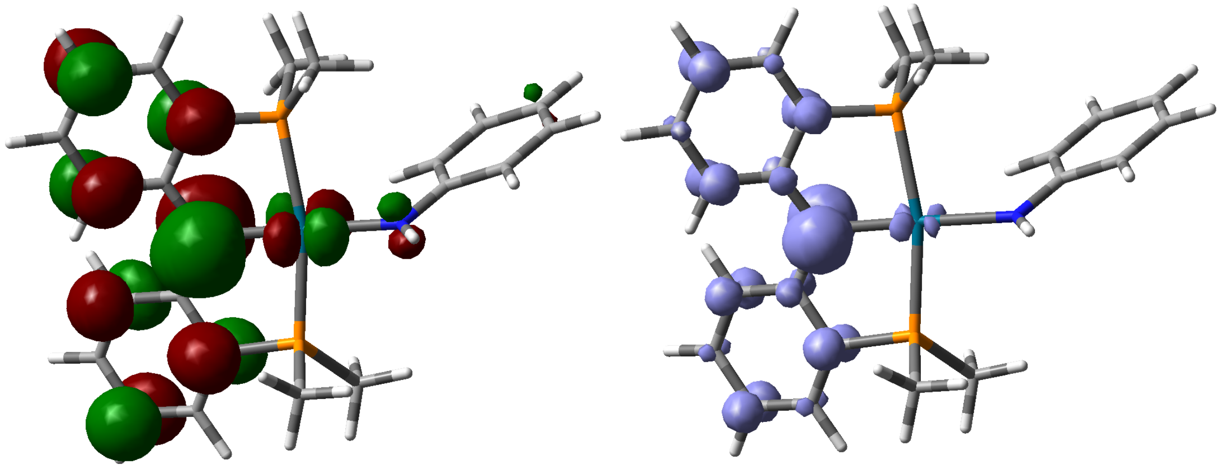

Figure S13. SOMO (left) and spin density (right) for [{PC$^{\bullet}$(sp^2)P}^{Me}PdNHPh] (**2'**).

3.4 [{PC$^\bullet$(sp^2)P}^{Me}PdNPh$_2$] (3')

Table S7. Optimized coordinates for [{PC$^\bullet$(sp^2)P}^{Me}PdNPh$_2$] (**3'**).

atom	x	y	z
C	−2.131918	0.814338	−2.304877
C	−2.768275	0.664222	−1.037100
C	−4.043837	1.290399	−0.876910
C	−4.636240	2.010385	−1.925808
C	−3.992094	2.145800	−3.174284
C	−2.730850	1.540407	−3.347082
N	−2.095689	0.000003	0.000006
C	−2.768259	−0.664224	1.037118
C	−4.043816	−1.290416	0.876938
C	−4.636201	−2.010408	1.925841
C	−3.992045	−2.145816	3.174312
C	−2.730806	−1.540408	3.347100
C	−2.131891	−0.814332	2.304890
Pd	0.032860	0.000003	−0.000007
P	0.320678	−2.351202	−0.191606
C	−0.692098	−3.273676	−1.477359
H	−1.746484	−3.220539	−1.189290
P	0.320684	2.351209	0.191589
C	−0.692093	3.273692	1.477334
H	−1.746477	3.220563	1.189259
C	2.096310	2.384220	0.771410
C	2.789220	1.136901	0.605319
C	4.123885	1.077918	1.123468
C	4.729135	2.193475	1.718024
C	4.040272	3.420733	1.821474
C	2.714355	3.502029	1.347954
C	2.090328	0.000000	−0.000002
C	2.789220	−1.136902	−0.605320
C	2.096307	−2.384218	−0.771418
C	2.714351	−3.502028	−1.347961
C	4.040271	−3.420736	−1.821473
C	4.729137	−2.193480	−1.718016
C	4.123888	−1.077923	−1.123462
H	4.515197	4.284721	2.278907
H	4.515196	−4.284725	−2.278904
C	0.240935	3.471109	−1.320434
H	0.516940	4.497438	−1.051656
H	0.934692	3.093345	−2.076912
H	−0.568473	2.787682	2.449070

Continued on next page

Table S7. – continued from previous page

atom	x	y	x
C	0.240918	−3.471104	1.320414
H	0.516921	−4.497434	1.051636
H	0.934672	−3.093344	2.076896
H	−0.568468	−2.787665	−2.449093
H	2.168594	4.438415	1.450538
H	5.738283	2.105794	2.115091
H	4.662807	0.136632	1.098636
H	−0.773589	3.448713	−1.729008
H	−0.384976	4.323233	1.549295
H	−4.554330	−1.222976	−0.079469
H	−5.606072	−2.478472	1.763687
H	−4.458927	−2.701461	3.983657
H	−2.214588	−1.623540	4.302360
H	−1.164354	−0.338386	2.454731
H	2.168587	−4.438412	−1.450549
H	5.738289	−2.105803	−2.115077
H	4.662814	−0.136639	−1.098624
H	−0.773608	−3.448707	1.728983
H	−0.384989	−4.323219	−1.549319
H	−4.554344	1.222954	0.079501
H	−5.606114	2.478438	−1.763646
H	−4.458989	2.701441	−3.983625
H	−2.214640	1.623546	−4.302346
H	−1.164377	0.338403	−2.454726

Table S8. Selected distances (Å) and angles (°) for the optimized geometry and the crystal structure of [{PC$^{\bullet}$(sp^2)P}^{Me}PdNPh$_2$] (**3'**).

Distance	Calcd.	X-Ray	Angle	Calcd.	X-Ray
Pd – N	2.129	2.149(2)	P – Pd – P#1	166.09	163.80(2)
Pd – C	2.057	2.024(2)	C – Pd – N	180.00	180.0
Pd – P	2.376	2.3021(5)	C – Pd – P	83.04	81.900(12)
Pd – P#1	2.376	2.3021(5)	C – Pd – P#1	83.04	81.900(12)
N – C(31)	1.403	1.357(2)	N – Pd – P	96.96	98.100(12)
N – C(31)#1	1.403	1.357(2)	N – Pd – P#1	96.96	98.100(12)
C – C(11)	1.465	1.444(2)	Pd – N – C(31)	118.64	117.94(12)
C – C(11)#1	1.465	1.444(2)	Pd – N – C(31)#1	118.64	117.94(12)

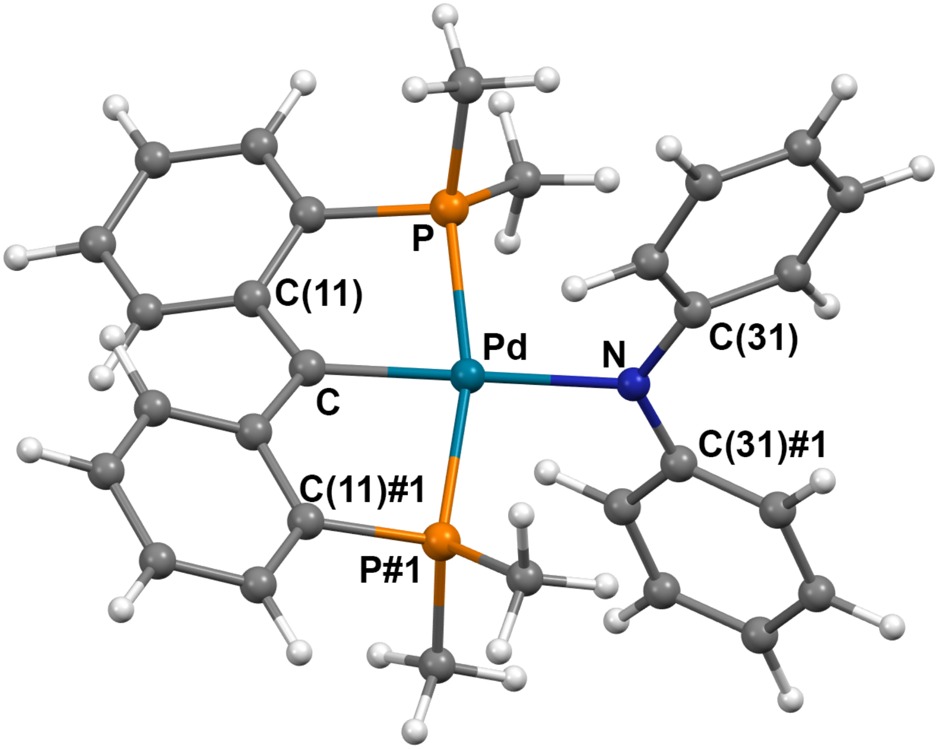

Figure S14. Optimized geometry for [{PC$^{\bullet}$(sp^2)P}^{Me}PdNPh$_2$] (**3'**).

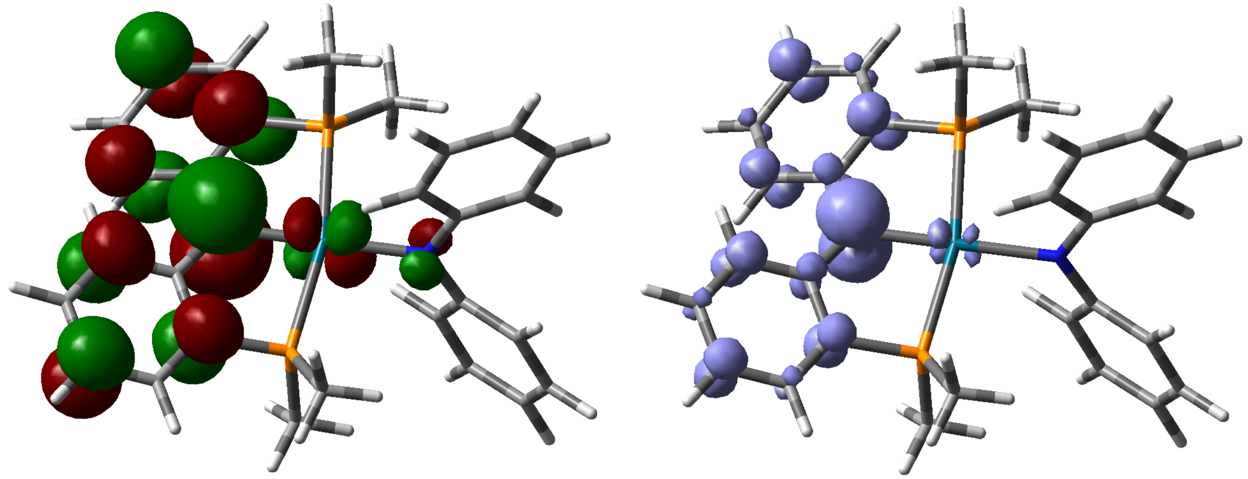

Figure S15. SOMO (left) and spin density (right) for [{PC$^{\bullet}$(sp^2)P}^{Me}PdNPh$_2$] (**3'**).

3.5 [{PC(sp^2)P}^{Me}PdNHPh]$^-$ (4’)

Table S9. Optimized coordinates for [{PC(sp^2)P}^{Me}PdNHPh]$^-$ (**4’**).

atom	x	y	z
C	−4.836109	−0.170071	−0.817446
C	−3.510597	−0.191309	−0.247653
C	−3.443169	−0.177755	1.190731
C	−4.601098	−0.144457	1.978093
C	−5.892375	−0.122065	1.398701
C	−5.983468	−0.136937	−0.011817
N	−2.371845	−0.225168	−1.006148
Pd	−0.319688	−0.068505	−0.424108
C	1.718517	0.096384	0.044445
C	2.441705	−1.111792	0.373797
C	1.889010	−2.426417	0.094030
C	2.568639	−3.623696	0.364533
C	3.837376	−3.622173	0.976984
C	4.380231	−2.362447	1.342929
C	3.721186	−1.164283	1.062508
P	0.180791	−2.363008	−0.610817
C	0.232058	−3.235646	−2.288051
H	0.612302	−4.258861	−2.179413
H	4.362297	−4.550021	1.192477
P	−0.316437	2.252493	−0.009231
C	−1.114340	3.387705	−1.286262
H	−2.186843	3.170378	−1.327589
C	1.497733	2.614127	−0.006962
C	2.315771	1.413771	−0.003154
C	3.737354	1.688697	−0.127930
C	4.255289	2.984698	−0.173879
C	3.420568	4.130097	−0.101848
C	2.030004	3.911530	−0.033947
H	3.833848	5.135765	−0.129016
C	−1.029299	2.921705	1.607940
H	−0.863471	4.003585	1.684533
C	−0.884518	−3.539661	0.407857
H	−0.836364	−3.226756	1.454927
H	−0.524498	2.418132	2.437776
H	−0.667967	3.179884	−2.263443
H	−1.922278	−3.466660	0.066712
H	0.895373	−2.671872	−2.950809
H	1.354901	4.769421	−0.018462
H	5.333032	3.113547	−0.286894

Continued on next page

Table S9. – continued from previous page

atom	x	y	x
H	4.421536	0.856393	−0.250941
H	−2.553697	−0.231673	−2.012540
H	2.098580	−4.576394	0.112181
H	5.331142	−2.323863	1.876939
H	4.157491	−0.239924	1.424669
H	−2.100607	2.698808	1.651305
H	−0.958721	4.443588	−1.032860
H	−0.529694	−4.574292	0.321098
H	−2.456912	−0.207178	1.650913
H	−4.498098	−0.138769	3.064189
H	−6.964514	−0.121665	−0.489803
H	−4.935182	−0.179948	−1.904552
H	−0.775256	−3.259754	−2.719191
H	−6.785936	−0.096779	2.018921

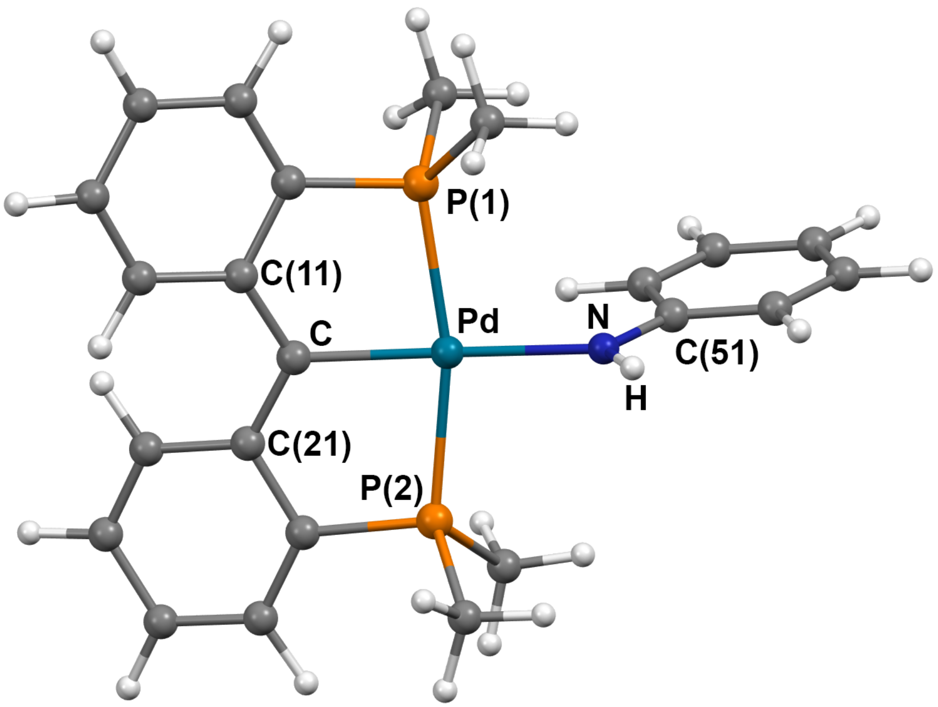

Figure S16. Optimized geometry for [{PC(sp^2)P}^{Me}PdNHPh]$^-$ (**4’**).

Table S10. Selected distances (Å) and angles (°) for the optimized geometry of [{PC(sp^2)P}^{Me}PdNHPh]$^-$ (**4'**).

Distance	Calcd.	Angle	Calcd.
Pd – N	2.139	P(1) – Pd – P(2)	166.51
Pd – C	2.098	C – Pd – N	177.10
Pd – P(1)	2.358	C – Pd – P(1)	83.22
Pd – P(2)	2.357	C – Pd – P(2)	83.56
N – C(51)	1.369	N – Pd – P(1)	96.97
C – C(11)	1.447	N – Pd – P(2)	96.37
C – C(21)	1.446	Pd – N – C(51)	130.22

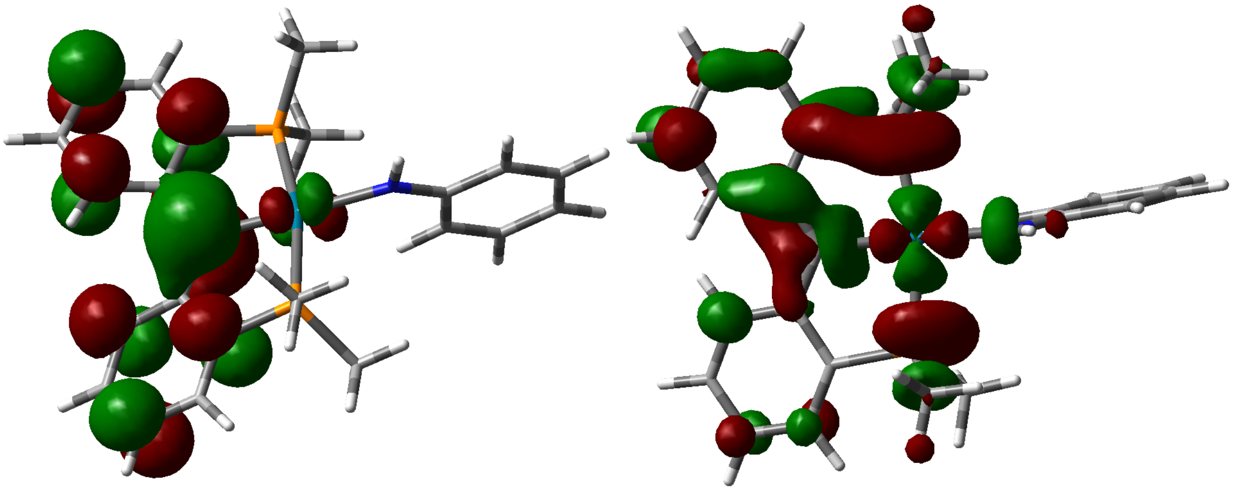

Figure S17. Frontier molecular orbitals for [{PC(sp^2)P}^{Me}PdNHPh]$^-$ (**4'**) (left: HOMO, right: LUMO).

3.6 [{PC(sp^2)P}^{Me}PdNPh$_2$]$^-$ (5')

Table S11. Optimized coordinates for [{PC(sp^2)P}^{Me}PdNPh$_2$]$^-$ (**5'**).

atom	x	y	z
C	−2.145481	0.691039	−2.337995
C	−2.819230	0.580309	−1.080935
C	−4.123319	1.172432	−1.005822
C	−4.705179	1.805790	−2.115326
C	−4.029154	1.886586	−3.351465
C	−2.737049	1.324152	−3.440316
N	−2.150681	−0.000025	0.000009
C	−2.819194	−0.580361	1.080976
C	−4.123283	−1.172493	1.005904
C	−4.705107	−1.805847	2.115428
C	−4.029049	−1.886625	3.351549
C	−2.736949	−1.324173	3.440364
C	−2.145415	−0.691065	2.338020
Pd	0.049024	−0.000004	−0.000017
P	0.339739	−2.341170	−0.196792
C	−0.658549	−3.247601	−1.514298
H	−1.720921	−3.192111	−1.254737
P	0.339704	2.341169	0.196718
C	−0.658642	3.247651	1.514145
H	−1.721001	3.192165	1.254531
C	2.120233	2.430021	0.688958
C	2.816934	1.168705	0.519457
C	4.193725	1.201002	0.980096
C	4.792093	2.358358	1.482411
C	4.088828	3.587538	1.574322
C	2.736711	3.591249	1.177098
C	2.143225	0.000011	0.000013
C	2.816966	−1.168668	−0.519422
C	2.120287	−2.429992	−0.688962
C	2.736799	−3.591207	−1.177089
C	4.088930	−3.587473	−1.574269
C	4.792171	−2.358281	−1.482331
C	4.193770	−1.200939	−0.980026
H	4.565566	4.484919	1.961896
H	4.565694	−4.484844	−1.961832
C	0.135806	3.471549	−1.303256
H	0.398548	4.504297	−1.042110
H	0.806520	3.114743	−2.090522
H	−0.502473	2.750739	2.476051

Continued on next page

Table S11. – continued from previous page

atom	x	y	x
C	0.135780	−3.471609	1.303128
H	0.398538	−4.504346	1.041954
H	0.806456	−3.114831	2.090439
H	−0.502325	−2.750661	−2.476180
H	2.156948	4.511985	1.264725
H	5.825057	2.305084	1.829860
H	4.763166	0.277844	0.988680
H	−0.896173	3.422289	−1.664727
H	−0.350301	4.297457	1.592329
H	−4.660837	−1.156295	0.062393
H	−5.694903	−2.250682	2.009190
H	−4.487402	−2.374341	4.209332
H	−2.184373	−1.377558	4.378100
H	−1.144362	−0.271724	2.414482
H	2.157053	−4.511951	−1.264743
H	5.825145	−2.304989	−1.829749
H	4.763195	−0.277771	−0.988588
H	−0.896216	−3.422368	1.664554
H	−0.350214	−4.297408	−1.592498
H	−4.660848	1.156222	−0.062297
H	−5.694976	2.250613	−2.009058
H	−4.487535	2.374306	−4.209231
H	−2.184497	1.377553	−4.378066
H	−1.144424	0.271712	−2.414485

Table S12. Selected distances (Å) and angles (°) for the optimized geometry of [{PC(sp^2)P}^{Me}PdNPh$_2$]$^-$ (**5'**).

Distance	Calcd.	Angle	Calcd.
Pd – N	2.200	P(1) – Pd – P(2)	165.89
Pd – C	2.094	C – Pd – N	180.00
Pd – P(1)	2.367	C – Pd – P(1)	82.95
Pd – P(2)	2.367	C – Pd – P(2)	82.95
N – C(3)	1.397	N – Pd – P(1)	97.05
N – C(4)	1.397	N – Pd – P(2)	97.05
C – C(1)	1.446	Pd – N – C(3)	118.59
C – C(2)	1.446	Pd – N – C(4)	118.59

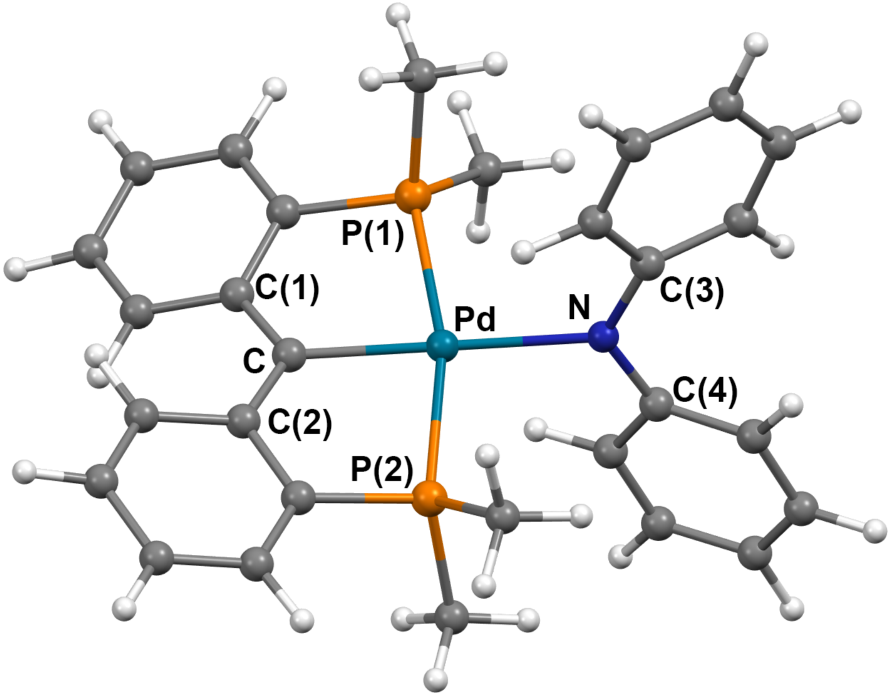

Figure S18. Optimized geometry for [{PC(sp^2)P}^{Me}PdNPh$_2$]$^-$ (**5'**).

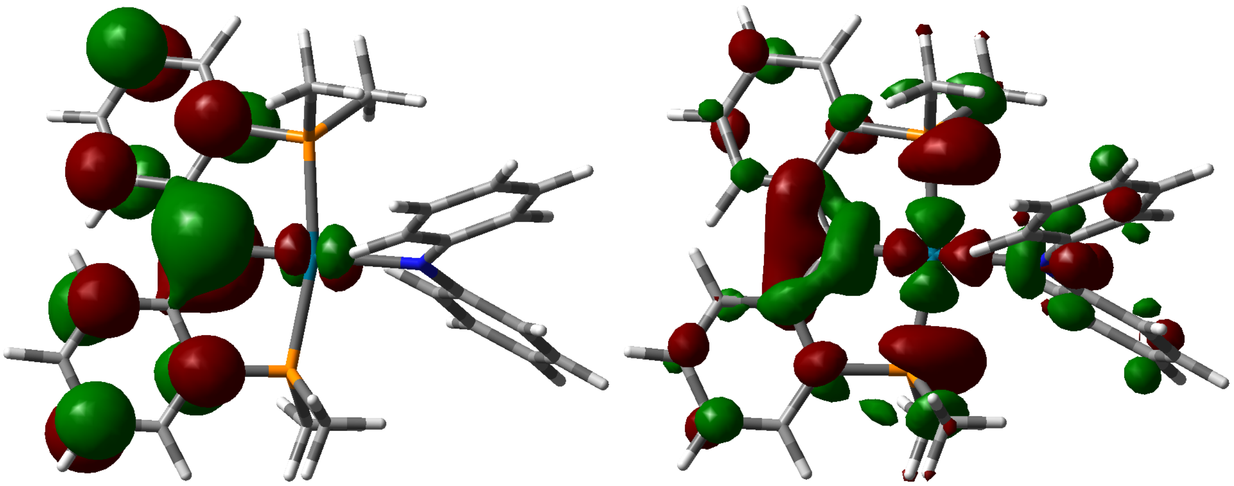

Figure S19. Frontier molecular orbitals for [{PC(sp^2)P}^{Me}PdNPh$_2$]$^-$ (**5'**) (left: HOMO, right: LUMO).

3.7 [{PC(sp^2)-K(OEt_2)(C_6H_6)P}^{Me}PdNPh$_2$] (5”)

Table S13. Optimized coordinates for [{PC(sp^2)-K(OEt_2)(C_6H_6)P}^{Me}PdNPh$_2$] (**5”**).

atom	x	y	z
Pd	1.219841	−0.385802	0.387587
N	3.045871	0.414415	−0.459697
C	4.211994	−0.364326	−0.380016
C	4.338498	−1.338516	0.653933
C	5.449069	−2.193924	0.727114
H	5.507273	−2.920872	1.536053
H	3.542794	−1.412421	1.391950
C	6.484640	−2.118612	−0.226381
H	7.346412	−2.778744	−0.166513
C	6.372924	−1.168723	−1.263974
H	7.151517	−1.101401	−2.022718
C	5.264720	−0.311109	−1.346422
H	5.195071	0.392681	−2.170993
C	3.035153	1.700170	−1.001603
C	1.811800	2.240617	−1.505782
C	1.728821	3.553360	−1.994836
C	2.857381	4.397746	−2.005957
C	4.074367	3.887802	−1.503675
C	4.169120	2.577296	−1.013231
H	5.116063	2.225985	−0.614413
H	4.959647	4.522196	−1.485896
H	2.794033	5.412974	−2.389812
H	0.778841	3.920783	−2.382655
H	0.938742	1.589356	−1.505585
P	1.244661	0.962530	2.320290
C	1.577359	2.812679	2.241559
C	2.396033	0.393963	3.703271
H	3.435414	0.468011	3.367371
H	2.249850	1.008654	4.599103
H	2.167328	−0.649332	3.938711
H	1.496559	3.268793	3.235407
H	2.583288	2.975098	1.843020
H	0.861864	3.281463	1.560635
P	0.874535	−2.071096	−1.248774
C	0.353035	−1.663540	−3.020971
C	2.269050	−3.302341	−1.538036
H	3.135119	−2.780776	−1.956245
H	2.557288	−3.736833	−0.577971
H	1.944916	−4.098974	−2.218098

Continued on next page

Table S13. – continued from previous page

atom	x	y	x
H	1.159427	−1.110494	−3.513695
H	−0.547618	−1.040605	−3.008752
H	0.144430	−2.582741	−3.581470
C	−0.530410	−1.159108	1.248893
C	−1.163312	−0.406785	2.319552
C	−0.484803	0.718529	2.941983
C	−1.071522	1.524274	3.927067
C	−2.395929	1.306038	4.361301
C	−3.110106	0.243681	3.760621
C	−2.531412	−0.580029	2.786518
C	−0.987782	−2.460246	0.784914
C	−0.512396	−3.022840	−0.465763
C	−0.991411	−4.234876	−0.988349
C	−1.942295	−5.006377	−0.292044
C	−2.349673	−4.545990	0.984422
C	−1.893080	−3.332142	1.506907
H	−0.495540	2.339691	4.365095
H	−2.846112	1.927182	5.130434
H	−4.140918	0.052790	4.060052
H	−3.143281	−1.369701	2.364337
H	−2.175718	−3.072266	2.520817
H	−3.013182	−5.162512	1.590171
H	−2.298392	−5.952403	−0.690309
H	−0.598922	−4.604931	−1.936253
K	−2.432296	0.561117	−0.279970
O	−2.794109	3.158047	−0.947951
C	−2.386857	3.752849	−2.229157
C	−3.262834	4.163464	0.020508
C	−3.729439	3.458464	1.291717
C	−1.926714	2.641904	−3.170449
H	−3.245426	4.296296	−2.655308
H	−1.574608	4.473165	−2.045965
H	−2.437149	4.859646	0.235979
H	−4.088287	4.732096	−0.437457
H	−2.913813	2.921457	1.789815
H	−4.543235	2.754763	1.073288
H	−4.113399	4.197635	2.006000
H	−2.729666	1.911939	−3.333317
H	−1.040234	2.130627	−2.776545
H	−1.652448	3.064992	−4.144648
C	−4.812989	−0.424760	−3.057974
C	−4.046211	−1.548695	−2.683078

Continued on next page

Table S13. – continued from previous page

atom	x	y	x
C	−4.202322	−2.119680	−1.401851
C	−5.132060	−1.562703	−0.497615
C	−5.898782	−0.438156	−0.870313
C	−5.738511	0.132308	−2.150796
H	−6.623429	−0.019709	−0.175522
H	−5.260561	−2.012204	0.483644
H	−3.614979	−2.987246	−1.113452
H	−3.343163	−1.989110	−3.386089
H	−4.703541	0.001063	−4.053058
H	−6.336189	0.992811	−2.442020

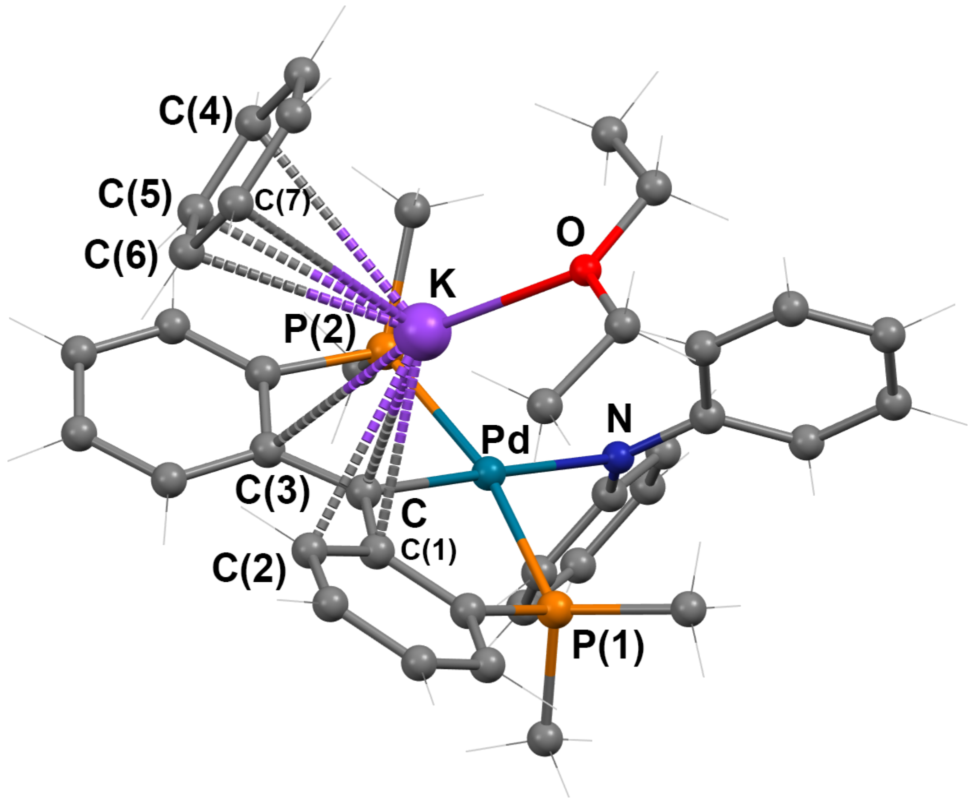

Figure S20. Optimized geometry for [{PC(sp^2)-K(OEt$_2$)(C$_6$H$_6$)P}^{Me}PdNPh$_2$] (**5’’**).

Table S14. Selected distances (Å) and angles (°) for the optimized geometry of [{PC(sp^2)-K(OEt_2)(C_6H_6)P}^{Me}PdNPh_2] (**5''**).

Distance	Calcd.	Angle	Calcd.
Pd – N	2.166	P(1) – Pd – P(2)	166.62
Pd – C	2.098	C – Pd – N	178.78
Pd – P(1)	2.357	C – Pd – P(1)	83.28
Pd – P(2)	2.374	C – Pd – P(2)	84.26
K – O	2.706	N – Pd – P(1)	95.76
K – C	2.989	N – Pd – P(2)	96.62
K – C(1)	3.050	O – K – C	139.87
K – C(2)	3.273	K – C – Pd	96.25
K – C(3)	3.514		
K – C(4)	3.582		
K – C(5)	3.403		
K – C(6)	3.442		
K – C(7)	3.656		

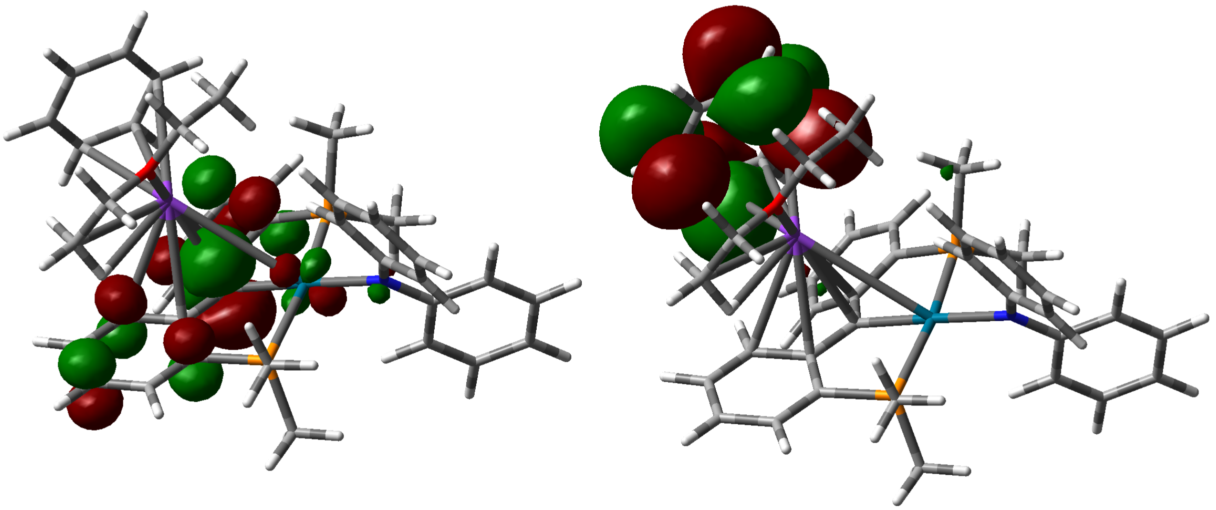

Figure S21. Frontier molecular orbitals for [{PC(sp^2)-K(OEt_2)(C_6H_6)P}^{Me}PdNPh_2] (**5''**) (left: HOMO, right: LUMO).

3.8 [{PC(sp^2)P}^{Me}PdCH$_2$Ph]$^-$ (6')

Table S15. Optimized coordinates for [{PC(sp^2)P}^{Me}PdCH$_2$Ph]$^-$ (**6'**).

atom	x	y	z
C	−3.964969	−1.335521	−1.199055
C	−3.283109	−1.458283	0.050811
C	−4.016540	−1.055368	1.209562
C	−5.327010	−0.555925	1.123095
C	−5.975358	−0.437459	−0.125857
C	−5.274696	−0.836998	−1.285622
C	−1.907229	−1.981722	0.136732
Pd	−0.184353	−0.558610	0.024603
C	1.590353	0.641580	−0.019718
C	2.857369	−0.022396	0.165257
C	2.979979	−1.466209	0.037217
C	4.191013	−2.158403	0.185690
C	5.382872	−1.484131	0.518503
C	5.297301	−0.081346	0.728143
C	4.102490	0.620174	0.562931
P	1.367690	−2.299397	−0.311331
C	1.516177	−3.085350	−2.027318
H	2.365828	−3.778604	−2.062907
H	6.321264	−2.020311	0.640755
P	−1.222665	1.542827	0.344760
C	−2.678739	2.097237	−0.719314
H	−3.543752	1.458017	−0.523667
C	0.159158	2.716649	−0.050733
C	1.445341	2.066075	−0.215818
C	2.495845	2.969088	−0.655958
C	2.292172	4.340572	−0.824183
C	1.033336	4.946210	−0.576116
C	−0.029486	4.097912	−0.203063
H	0.884238	6.016516	−0.700631
C	−1.819851	2.003152	2.080063
H	−2.102770	3.062401	2.119089
C	1.298934	−3.827958	0.798070
H	1.376444	−3.499094	1.838853
H	−1.001887	1.819713	2.783309
H	−2.394274	2.000898	−1.771568
H	0.341020	−4.340761	0.655467
H	1.672616	−2.282065	−2.753405
H	−1.021640	4.525544	−0.047128
H	3.123686	4.955324	−1.173477

Continued on next page

Table S15. – continued from previous page

atom	x	y	x
H	3.463282	2.554734	−0.918639
H	−1.687059	−2.682398	−0.681301
H	4.208901	−3.242813	0.057490
H	6.186071	0.466855	1.046046
H	4.089711	1.679118	0.797280
H	−2.679787	1.378929	2.344685
H	−2.934201	3.143209	−0.509399
H	2.122107	−4.517960	0.575444
H	−3.537981	−1.146664	2.184640
H	−5.849798	−0.262747	2.033784
H	−5.754556	−0.759118	−2.261277
H	−3.439795	−1.631224	−2.106768
H	0.590742	−3.618494	−2.274968
H	−6.990601	−0.051285	−0.192738
H	−1.695098	−2.452561	1.107942

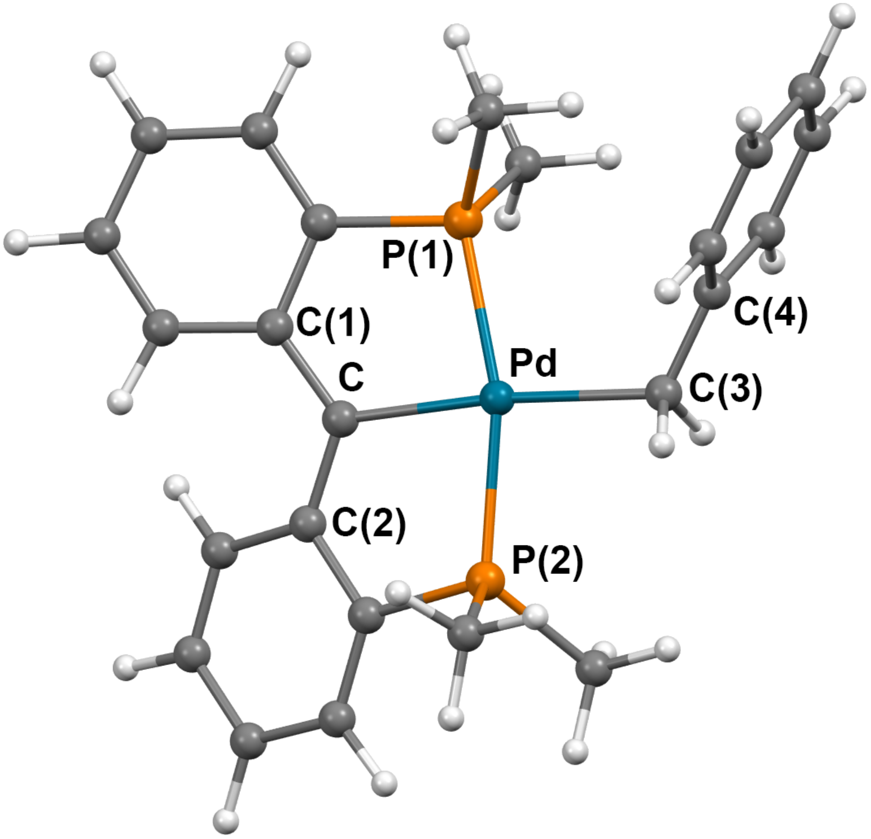

Figure S22. Optimized geometry for [{PC(sp^2)P}^{Me}PdCH$_2$Ph]$^-$ (**6’**).

Table S16. Selected distances (Å) and angles (°) for the optimized geometry of $[\{PC(sp^2)P\}^{Me}PdCH_2Ph]^-$ (**6'**).

**Distance**	**Calcd.**	**Angle**	**Calcd.**
Pd – C(3)	2.237	P(1) – Pd – P(2)	164.72
Pd – C	2.143	C – Pd – C(3)	174.26
Pd – P(1)	2.366	C – Pd – P(1)	82.46
Pd – P(2)	2.356	C – Pd – P(2)	82.26
C(3) – C(4)	1.475	C(3) – Pd – P(1)	102.72
C – C(1)	1.445	C(3) – Pd – P(2)	92.55
C – C(2)	1.442	Pd – C(3) – C(4)	119.33

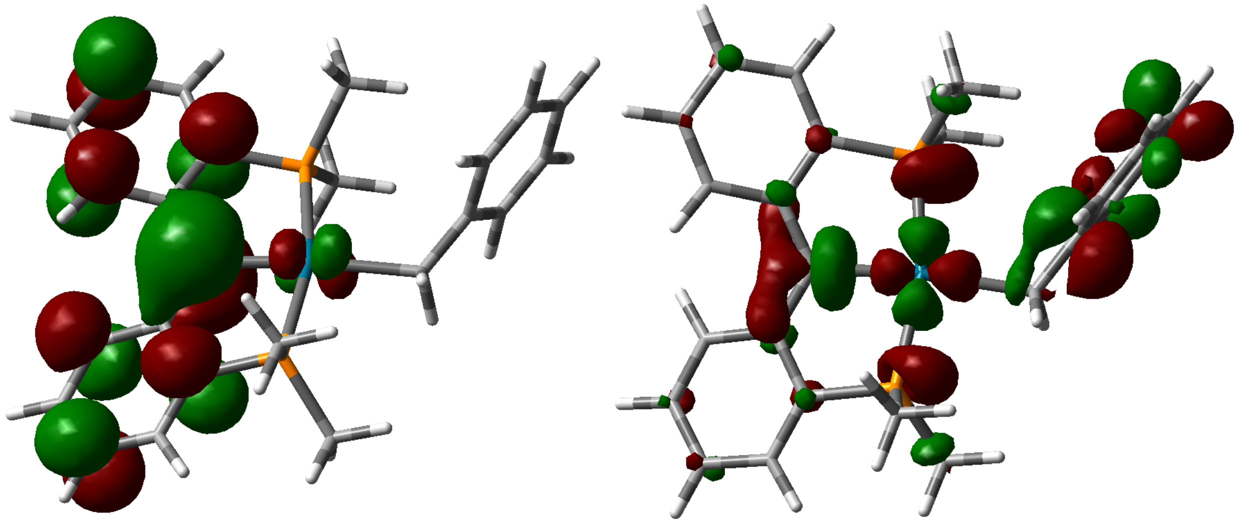

Figure S23. Frontier molecular orbitals for $[\{PC(sp^2)P\}^{Me}PdCH_2Ph]^-$ (**6'**) (left: HOMO, right: LUMO).

3.9 [{PC(sp^2)-K(C_6H_6)P}^{Me}PdCH_2Ph]$_2K^+$ (6”)

Table S17. Optimized coordinates for [{PC(sp^2)-K(C_6H_6)P}^{Me}PdCH_2Ph]$_2K^+$ (**6”**).

atom	x	y	z
C	−2.393398	−5.261673	0.406852
C	−3.630152	−4.619908	0.692934
C	−4.777920	−5.097098	0.002042
C	−4.693117	−6.142791	−0.935436
C	−3.453628	−6.753730	−1.214782
C	−2.304899	−6.306795	−0.528543
C	−3.718026	−3.515003	1.689709
Pd	−3.287318	−1.475904	1.023537
P	−3.694889	−1.678123	−1.302469
C	−5.431928	−2.018650	−1.963879
H	−6.147864	−1.314578	−1.527155
C	−2.891118	0.595678	0.542270
C	−2.821696	0.971657	−0.866228
C	−3.239898	0.033071	−1.901596
C	−3.252284	0.362279	−3.266170
C	−2.811632	1.619492	−3.729050
C	−2.294315	2.520452	−2.771431
C	−2.284886	2.213977	−1.403710
K	−5.660247	1.489097	0.680373
C	−2.862890	2.994140	1.566414
C	−2.779907	1.544093	1.654285
C	−2.695247	1.062484	3.026115
C	−2.595235	1.916252	4.135411
C	−2.621551	3.317385	3.990812
C	−2.782509	3.833613	2.687882
P	−2.803955	−0.784719	3.219437
C	−4.114157	−1.101193	4.539723
H	−5.065032	−0.659600	4.226078
H	−2.541074	3.972668	4.852671
H	−2.824082	1.864111	−4.787060
K	0.001563	0.007908	0.004872
C	2.889179	−0.595524	−0.535368
C	2.824029	−0.964868	0.875180
C	3.248102	−0.022371	1.904632
C	3.265233	−0.345388	3.270637
C	2.823953	−1.599614	3.740927
C	2.300895	−2.503789	2.789486
C	2.286702	−2.203549	1.420409
K	5.654430	−1.502218	−0.677034

Continued on next page

Table S17. – continued from previous page

atom	x	y	x
C	2.850693	−2.998534	−1.548382
C	2.771378	−1.548653	−1.642615
C	2.682915	−1.073184	−3.016386
C	2.576134	−1.931819	−4.121264
C	2.598811	−3.332349	−3.970269
C	2.763422	−3.842983	−2.665597
P	2.797246	0.772640	−3.219082
C	4.107953	1.077285	−4.541747
H	5.057738	0.635326	−4.225323
H	2.513013	−3.991383	−4.828746
P	3.703370	1.685278	1.295925
C	5.443822	2.026656	1.947623
H	6.155938	1.316833	1.513998
H	2.840053	−1.839414	4.799991
C	8.507339	−3.228185	0.425452
C	7.462680	−4.169466	0.556900
C	8.512830	−2.065165	1.226701
C	7.470763	−1.841471	2.153454
Pd	3.287104	1.473170	−1.027485
C	3.718274	3.508745	−1.704230
C	3.631670	4.618353	−0.712554
C	4.780479	5.099628	−0.026251
C	4.696765	6.149533	0.906619
C	3.457363	6.760721	1.185755
C	2.307577	6.309763	0.503905
C	2.394986	5.260456	−0.426877
C	1.234299	1.343210	−4.107605
H	1.124482	0.809568	−5.058539
C	2.678695	2.870485	2.345195
H	2.878266	2.704326	3.409821
H	0.350958	1.155925	−3.491606
H	4.247060	2.155962	−4.667671
H	1.613671	2.708803	2.153056
H	5.730815	3.042740	1.663318
C	−8.502469	3.232935	−0.415585
C	−7.453846	4.170442	−0.542408
C	−8.512856	2.073907	−1.222554
C	−7.471726	1.850394	−2.150428
C	−1.239229	−1.354631	4.105239
H	−1.131677	−0.826191	5.059319
C	−2.664211	−2.857233	−2.352771
H	−2.861145	−2.687979	−3.417409

Continued on next page

Table S17. – continued from previous page

atom	x	y	x
H	−0.356571	−1.160172	3.490474
H	−4.250460	−2.181037	4.658405
H	−1.600158	−2.693610	−2.156985
H	−5.717465	−3.037919	−1.689679
H	2.491849	−1.510769	−5.123188
H	2.822169	−4.920080	−2.513223
H	2.988664	−3.469956	−0.582230
H	4.734243	3.407210	−2.115989
H	3.013855	3.676173	−2.531182
H	3.608654	0.395346	3.993228
H	1.822828	−2.922894	0.755254
H	1.883689	−3.453901	3.121532
H	1.303107	2.418928	−4.302129
H	3.809951	0.635899	−5.499372
H	5.752560	4.663516	−0.256775
H	5.598841	6.504250	1.401869
H	3.392448	7.578073	1.899418
H	1.346264	6.784084	0.692711
H	1.498311	4.932101	−0.951817
H	2.928283	3.900221	2.078383
H	5.467335	1.935432	3.039565
H	−2.513430	1.490879	5.135715
H	−2.843825	4.911233	2.540280
H	−2.998727	3.469666	0.601967
H	−4.734400	−3.415994	2.101090
H	−3.014226	−3.686017	2.516474
H	−3.591417	−0.375866	−3.993423
H	−1.825503	2.931451	−0.733433
H	−1.877845	3.472961	−3.097481
H	−1.303900	−2.431756	4.293288
H	−3.817516	−0.665556	5.500377
H	−5.750081	−4.661045	0.232347
H	−5.594447	−6.494515	−1.434174
H	−3.387899	−7.567883	−1.932021
H	−1.343555	−6.780967	−0.717545
H	−1.497512	−4.936439	0.935070
H	−2.912071	−3.888487	−2.090145
H	−5.451161	−1.918013	−3.055072
C	−6.418542	2.783249	−2.272623
C	−6.413540	3.945018	−1.470358
H	−5.614734	4.673594	−1.580647
H	−5.614806	2.610119	−2.983543

Continued on next page

Table S17. – continued from previous page

atom	x	y	x
H	−7.491987	0.971057	−2.789287
H	−9.337898	1.369339	−1.150119
H	−9.317376	3.419108	0.279565
H	−7.460880	5.077541	0.056846
C	6.421407	−3.943862	1.483741
C	6.421476	−2.778110	2.280237
H	9.323053	−3.414365	−0.268745
H	7.473622	−5.079538	−0.037777
H	9.334898	−1.357499	1.150766
H	5.625819	−4.675404	1.597738
H	7.487204	−0.958911	2.787948
H	5.617054	−2.604660	2.990310

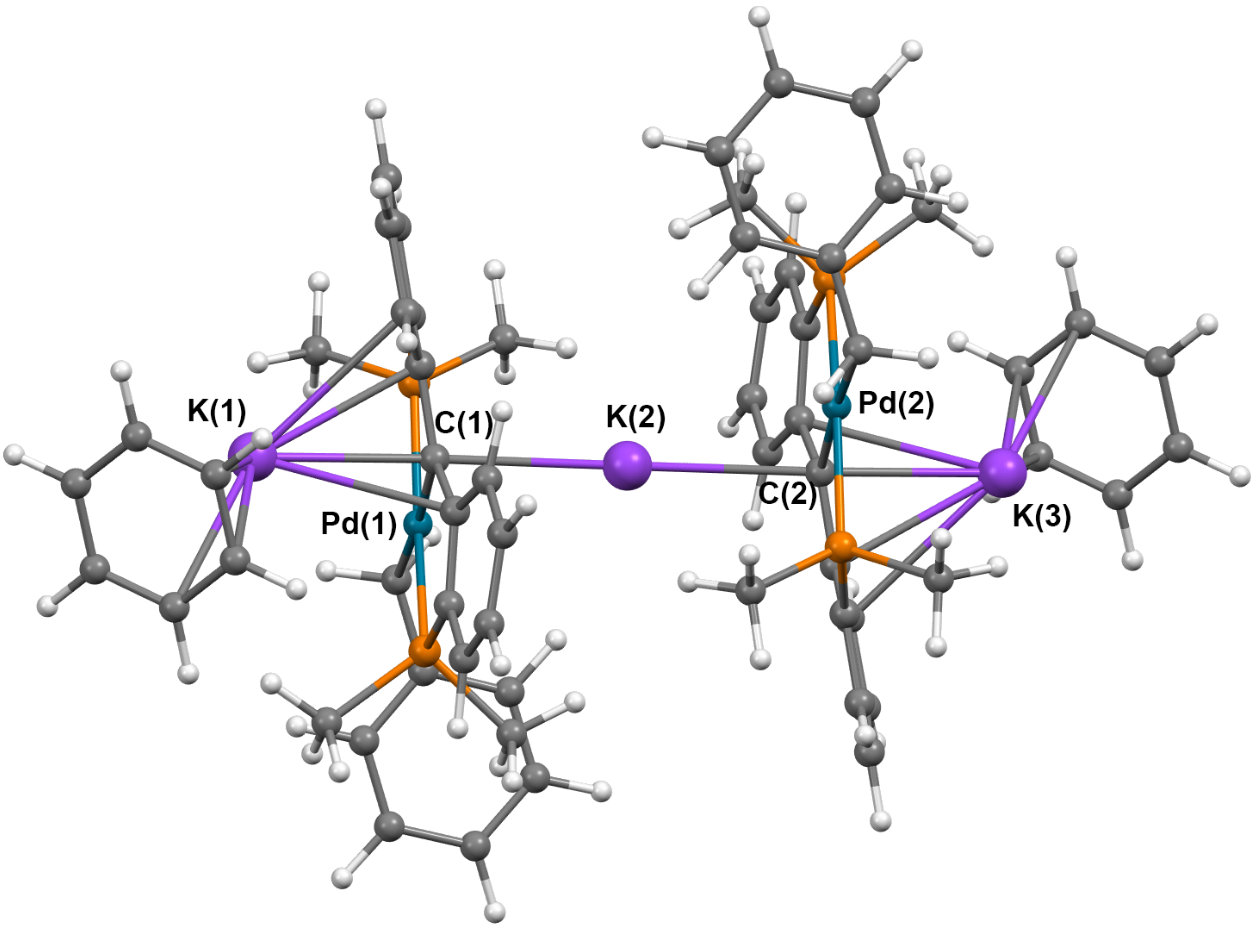

Figure S24. Optimized geometry for [{PC(sp^2)-K(C_6H_6)P}^{Me}PdCH_2Ph]$_2K^+$ (**6’’**).

Table S18. Selected distances (Å) and angles (°) for the optimized geometry of [{PC(sp^2)-K(C_6H_6)P}^{Me}PdCH_2Ph]$_2$K$^+$ (**6’’**).

**Distance**	**Calcd.**	**Angle**	**Calcd.**
Pd(1) – C(1)	2.163	K(1) – C(1) – K(2)	170.13
Pd(2) – C(2)	2.163	C(1) – K(2) – C(2)	179.68
K(1) – C(1)	2.914	K(2) – C(2) – K(3)	170.10
K(2) – C(1)	2.999	K(1) – C(1) – Pd(1)	96.43
K(2) – C(2)	3.000	K(3) – C(2) – Pd(2)	96.26
K(3) – C(2)	2.913	Pd(1) – C(1) – K(2)	91.47
		Pd(2) – C(2) – K(2)	91.65

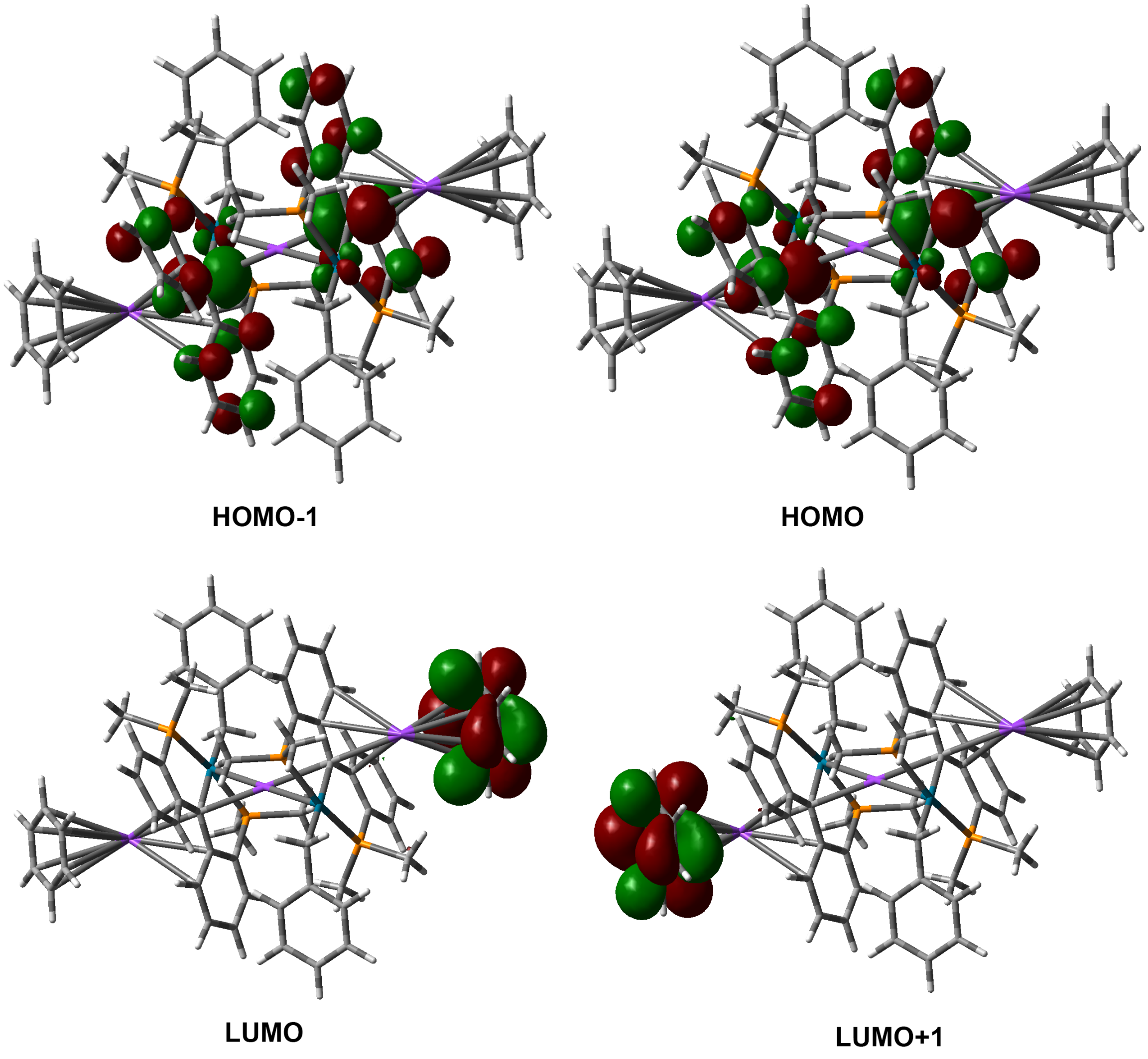

Figure S25. Frontier molecular orbitals for [{PC(sp^2)-K(C_6H_6)P}^{Me}PdCH_2Ph]$_2$K$^+$ (**6’’**) (top left: HOMO-1, top right: HOMO, bottom left: LUMO, bottom right: LUMO+1).

3.10 [{PC$^{\bullet}$(sp^2)P}^{Me}PdCH$_2$Ph] (7’)

Table S19. Optimized coordinates for [{PC$^{\bullet}$(sp^2)P}^{Me}PdCH$_2$Ph] (**7’**).

atom	x	y	z
C	−4.347685	−0.536723	−0.580230
C	−3.216833	−1.281455	−0.145991
C	−3.244359	−1.772121	1.189902
C	−4.330930	−1.521964	2.044120
C	−5.437288	−0.768306	1.596635
C	−5.436904	−0.281315	0.274509
C	−2.054142	−1.546242	−1.045002
Pd	−0.257509	−0.445055	−0.501144
C	1.507628	0.570449	0.063499
C	2.638404	−0.220821	0.550299
C	2.710192	−1.618182	0.216965
C	3.765741	−2.431369	0.651993
C	4.780746	−1.905749	1.478198
C	4.707087	−0.552168	1.871754
C	3.667921	0.271995	1.419637
P	1.258663	−2.246565	−0.782120
C	1.935664	−2.595246	−2.509130
H	2.764582	−3.311131	−2.464255
H	5.592314	−2.540222	1.825004
P	−1.234768	1.687879	−0.127769
C	−2.344170	2.502562	−1.413186
H	−3.268783	1.925301	−1.496009
C	0.304269	2.757303	−0.087784
C	1.542754	2.030395	−0.022796
C	2.741301	2.812286	−0.124327
C	2.703022	4.209465	−0.223171
C	1.471777	4.899385	−0.221074
C	0.274142	4.156975	−0.162170
H	1.444444	5.983969	−0.288527
C	−2.138886	2.015437	1.492291
H	−2.374577	3.081245	1.593561
C	0.898014	−3.973305	−0.120713
H	0.667493	−3.903953	0.946406
H	−1.496879	1.703962	2.321080
H	−1.828533	2.502818	−2.377939
H	0.028526	−4.384159	−0.644227
H	2.290337	−1.655492	−2.941602
H	−0.677856	4.684175	−0.195032
H	3.633958	4.765905	−0.312567

Continued on next page

Table S19. – continued from previous page

atom	x	y	x
H	3.700626	2.307316	−0.170337
H	−2.265733	−1.276680	−2.089489
H	3.800472	−3.482864	0.371719
H	5.459840	−0.144135	2.543430
H	3.616064	1.296167	1.774048
H	−3.060132	1.425494	1.514909
H	−2.587263	3.532889	−1.129821
H	1.752515	−4.643786	−0.265242
H	−2.394354	−2.347364	1.554004
H	−4.318646	−1.914353	3.059550
H	−6.291098	0.283180	−0.096038
H	−4.382370	−0.185962	−1.611413
H	1.136126	−2.997026	−3.140649
H	−6.279878	−0.577259	2.257055
H	−1.759006	−2.602898	−1.001993

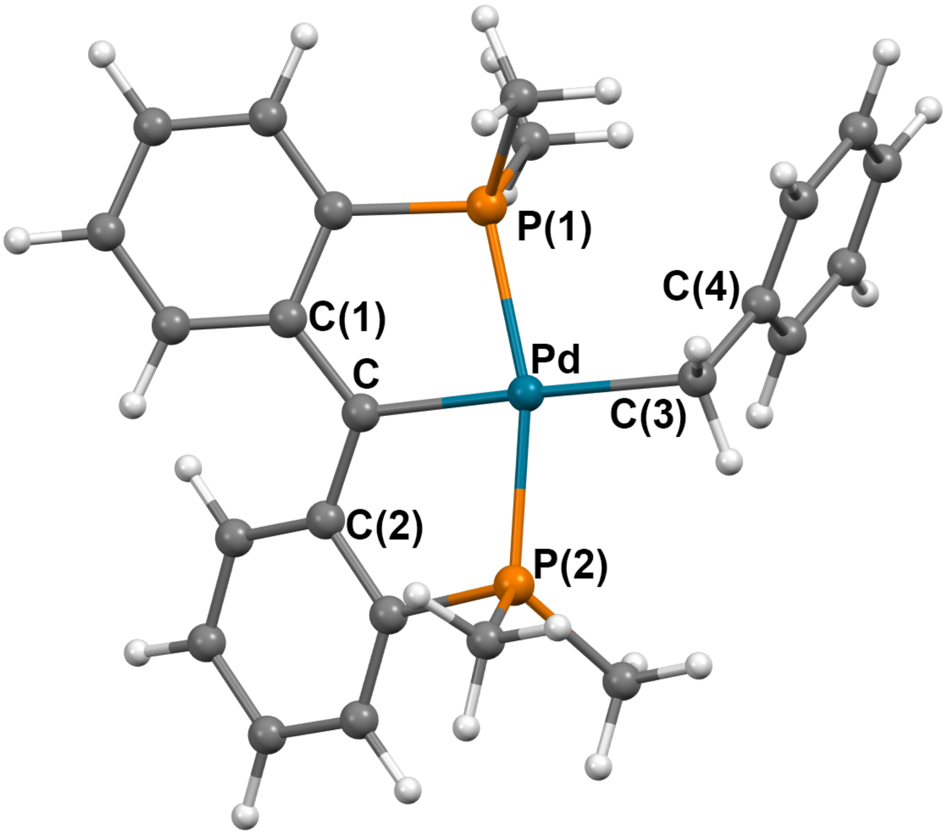

Figure S26. Optimized geometry for [{PC$^{\bullet}$(sp^2)P}^{Me}PdCH$_2$Ph] (**7’**).

Table S20. Selected distances (Å) and angles (°) for the optimized geometry of [{PC(sp^2)P}^{Me}PdCH$_2$Ph]$^-$ (**6'**).

Distance	Calcd.	Angle	Calcd.
Pd – C(3)	2.176	P(1) – Pd – P(2)	164.52
Pd – C	2.113	C – Pd – C(3)	178.15
Pd – P(1)	2.376	C – Pd – P(1)	82.54
Pd – P(2)	2.371	C – Pd – P(2)	82.11
C(3) – C(4)	1.493	C(3) – Pd – P(1)	98.86
C – C(1)	1.463	C(3) – Pd – P(2)	96.54
C – C(2)	1.463	Pd – C(3) – C(4)	113.74

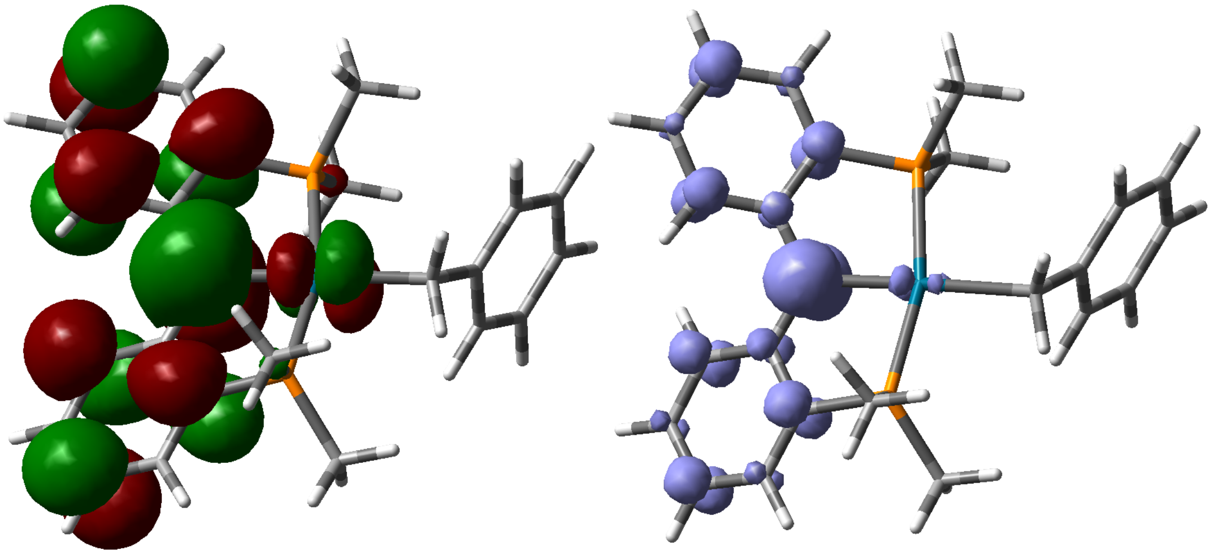

Figure S27. SOMO (left) and spin density (right) for [{PC$^•$(sp^2)P}^{Me}PdCH$_2$Ph] (**7'**).

4 NMR Spectra

4.1 NMR Spectra for [{PC(sp^2)P}^{tBu}PdNH^{p}Tol]$^-$K$^+$ (4)

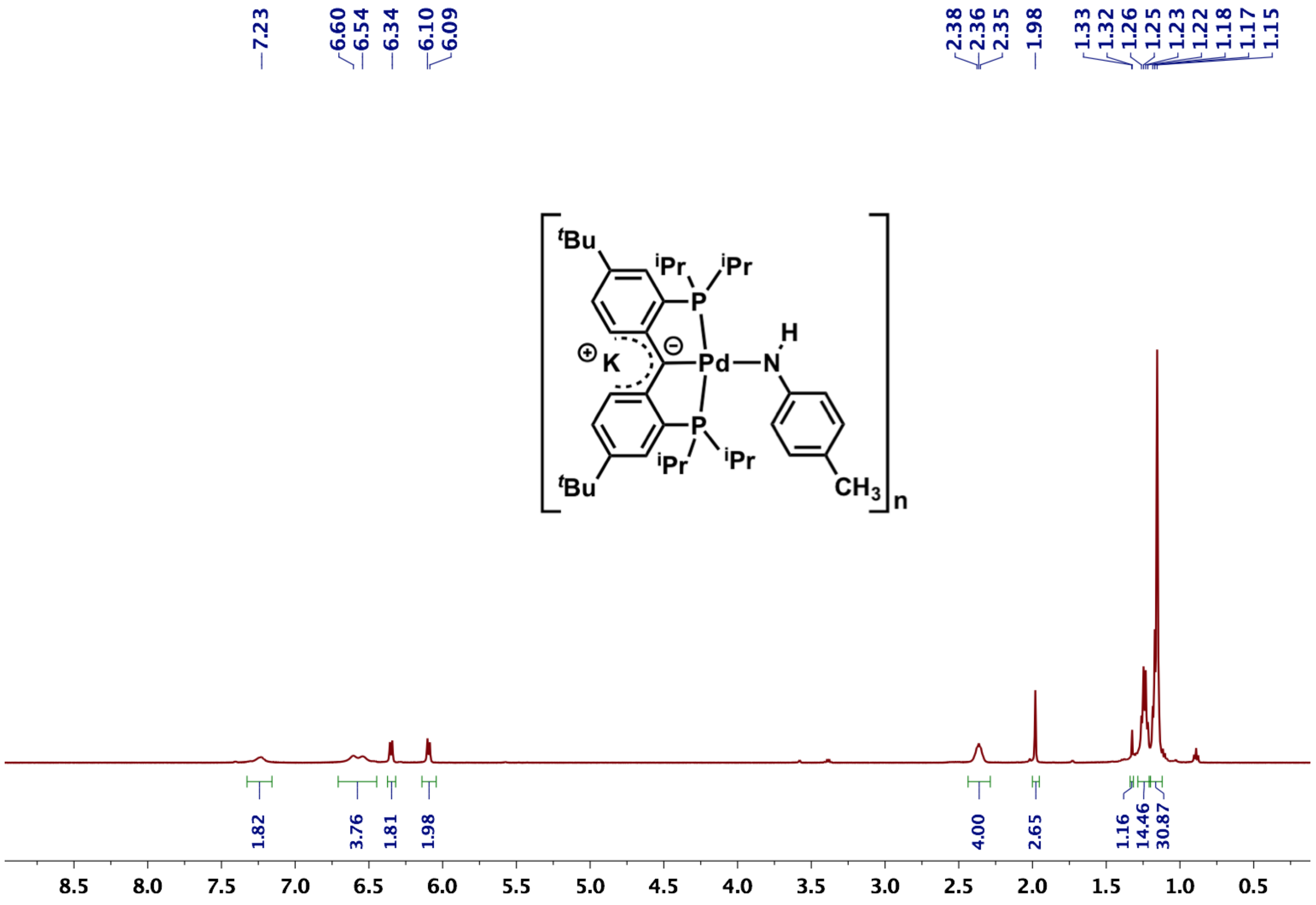

Figure S28. ^{1}H NMR spectrum for [{PC(sp^2)P}^{tBu}PdNH^{p}Tol]$^-$K$^+$ (**4**).

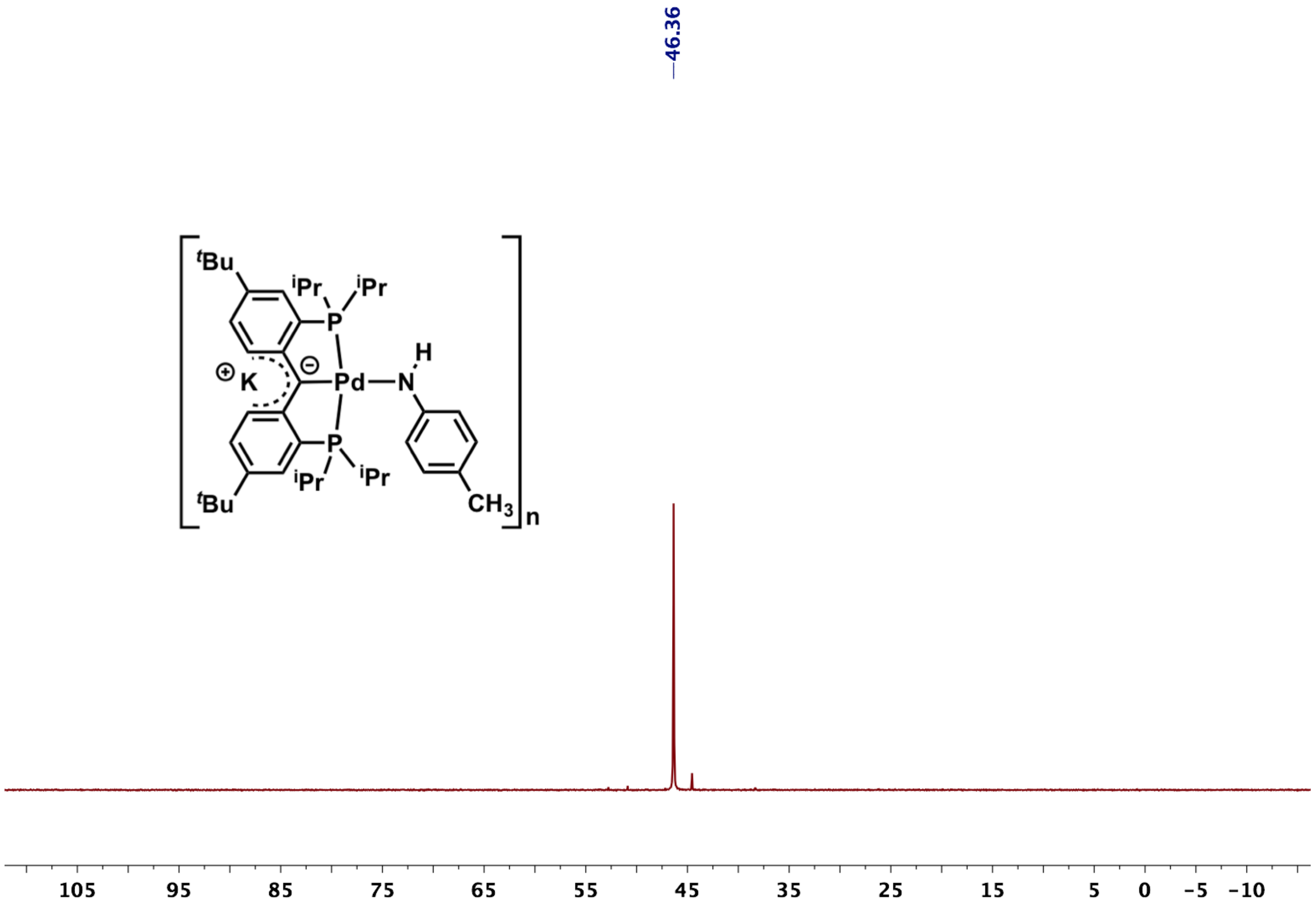

Figure S29. $^{31}P\{^{1}H\}$ NMR spectrum for [{PC(sp^2)P}^{tBu}PdNH^{p}Tol]$^{-}$K^{+} (**4**).

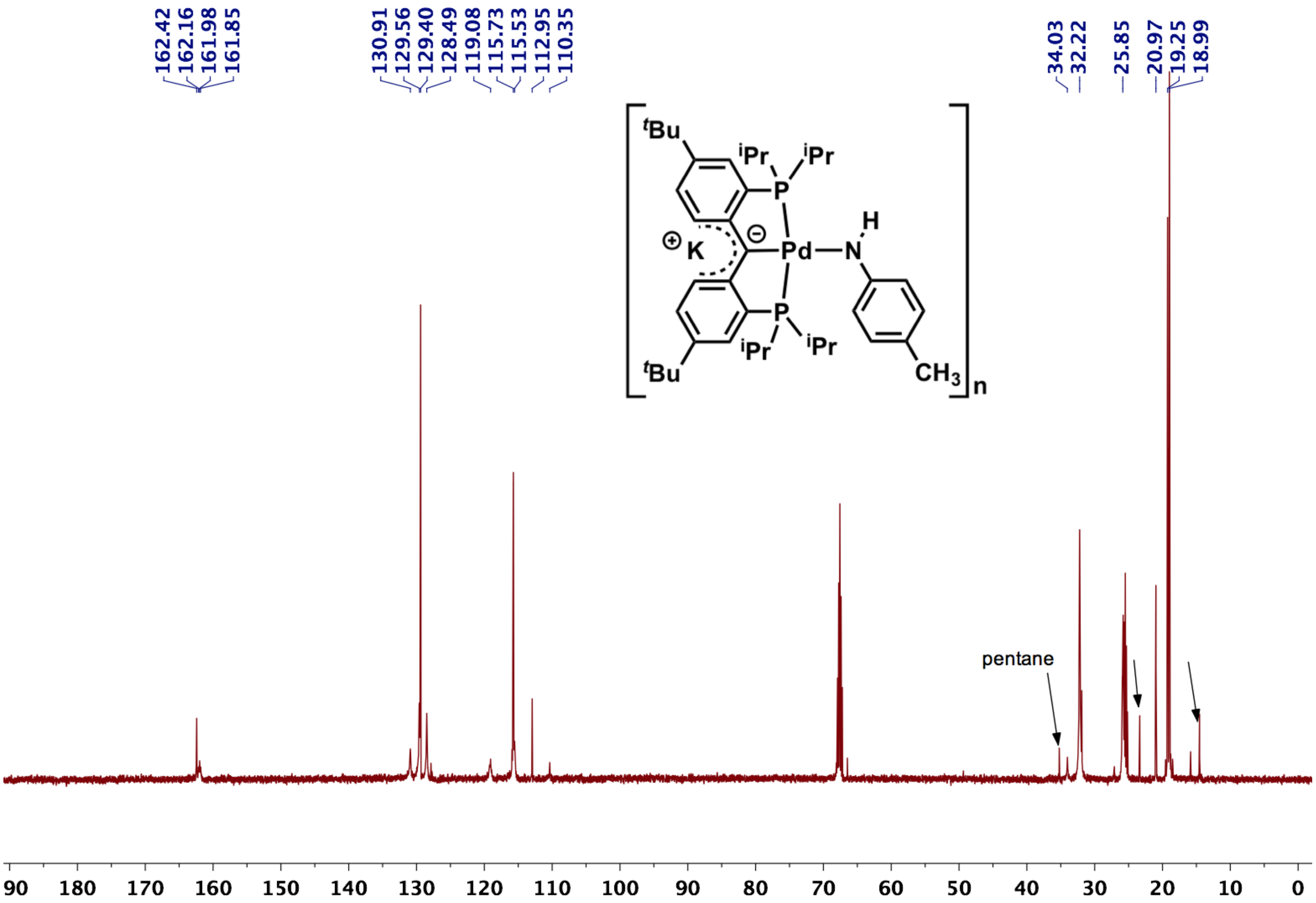

Figure S30. $^{13}C\{^{1}H\}$ NMR spectrum for [{PC(sp^2)P}^{tBu}PdNH^{p}Tol]$^-$K$^+$ (**4**).

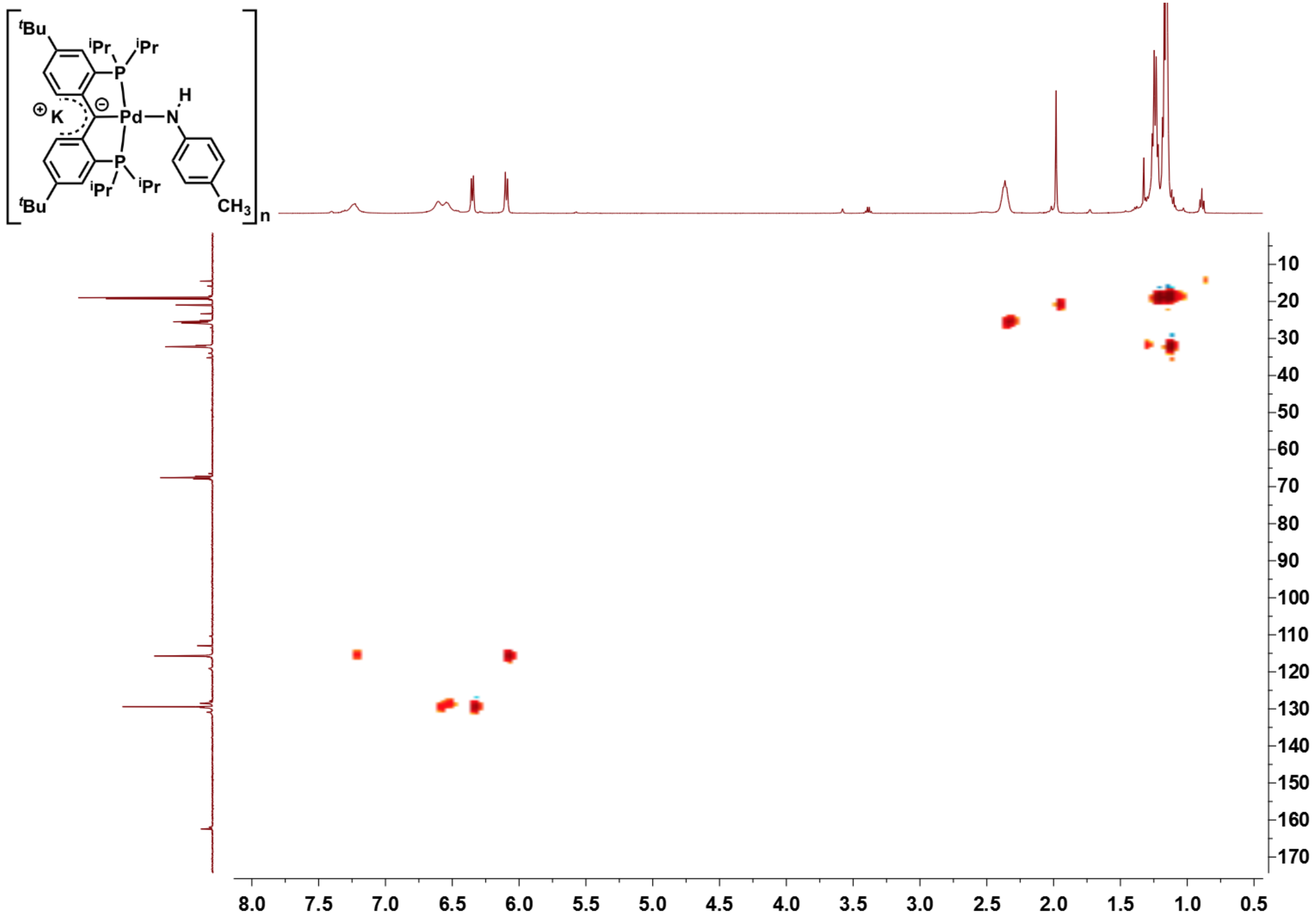

Figure S31. ^{1}H-^{13}C HSQC NMR spectrum for [{PC(sp^{2})P}^{tBu}PdNH^{p}Tol]$^{-}$K^{+} (**4**).

4.2 NMR Spectra for [{PC(sp^2)P}^{tBu}PdNPh$_2$]$^-$[KOEt$_2$]$^+$ (5)

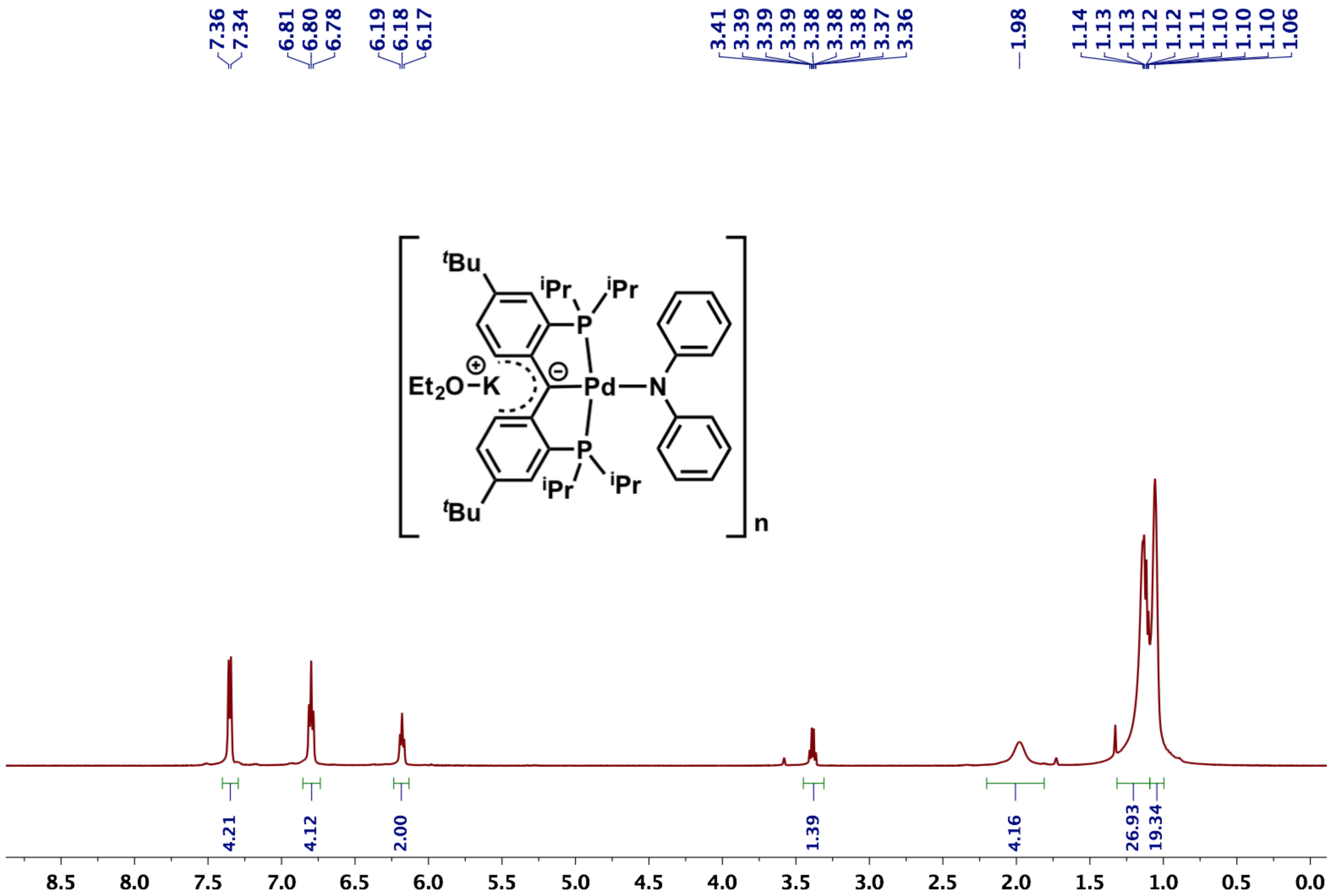

Figure S32. ^{1}H NMR spectrum for [{PC(sp^2)P}^{tBu}PdNPh$_2$]$^-$[KOEt$_2$]$^+$ (**5**).

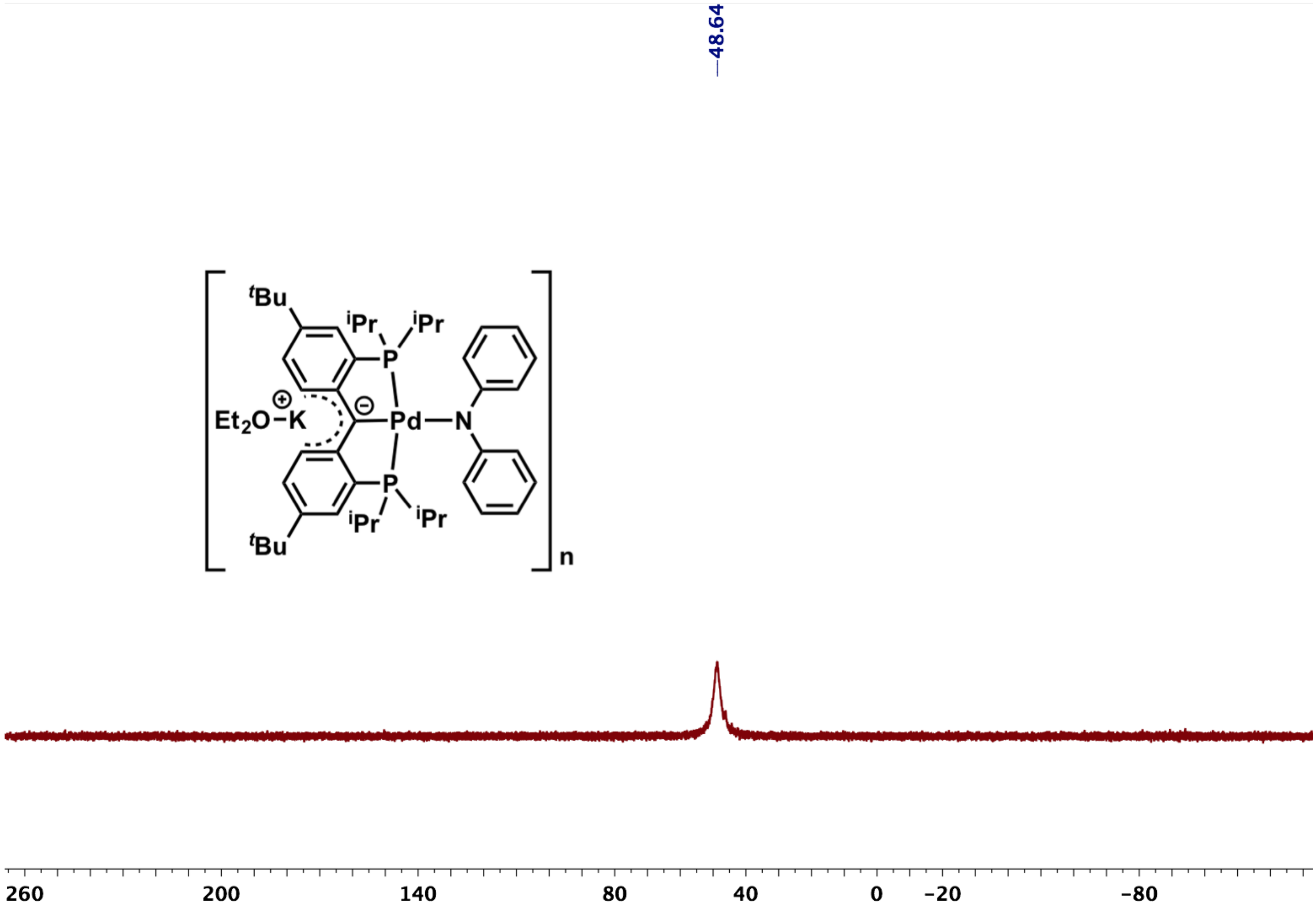

Figure S33. $^{31}P\{^{1}H\}$ NMR spectrum for $[\{PC(sp^2)P\}^{tBu}PdNPh_2]^{-}[KOEt_2]^{+}$ (**5**).

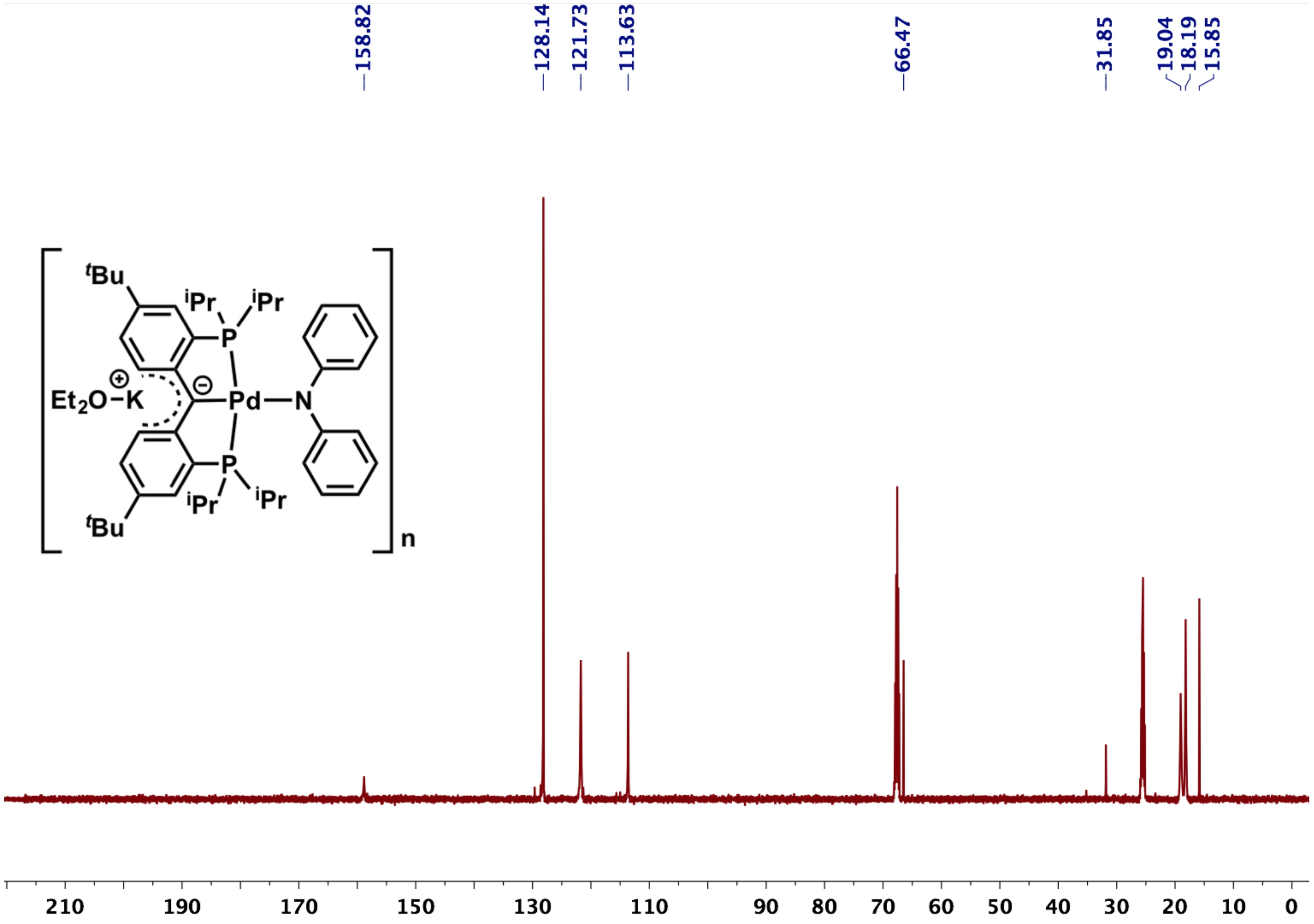

Figure S34. $^{13}C\{^1H\}$ NMR spectrum for $[\{PC(sp^2)P\}^{tBu}PdNPh_2]^-[KOEt_2]^+$ (**5**).

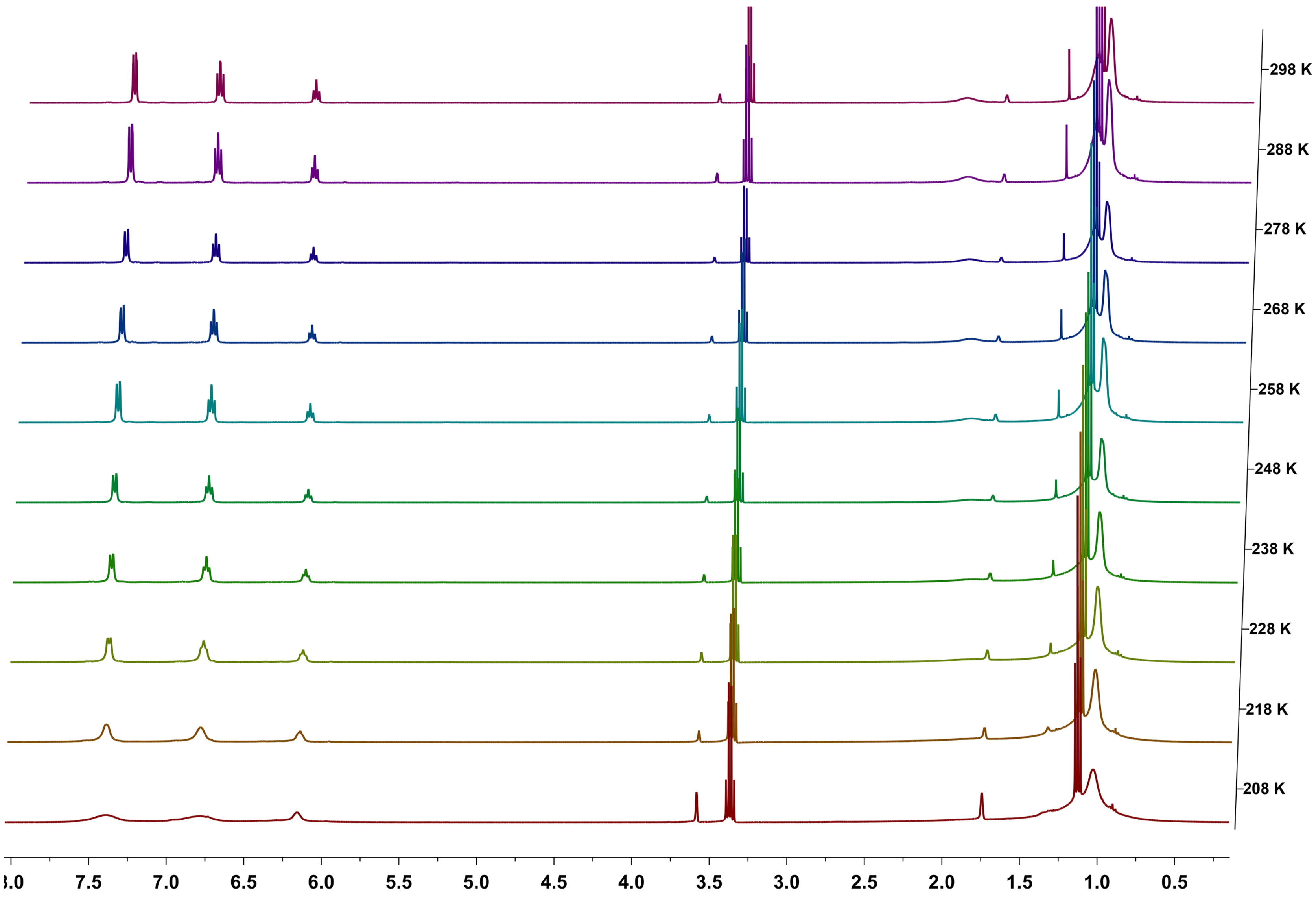

Figure S35. ^{1}H NMR spectra for $[\{PC(sp^2)P\}^{tBu}PdNPh_2]^-[KOEt_2]^+$ (**5**) at different temperatures.

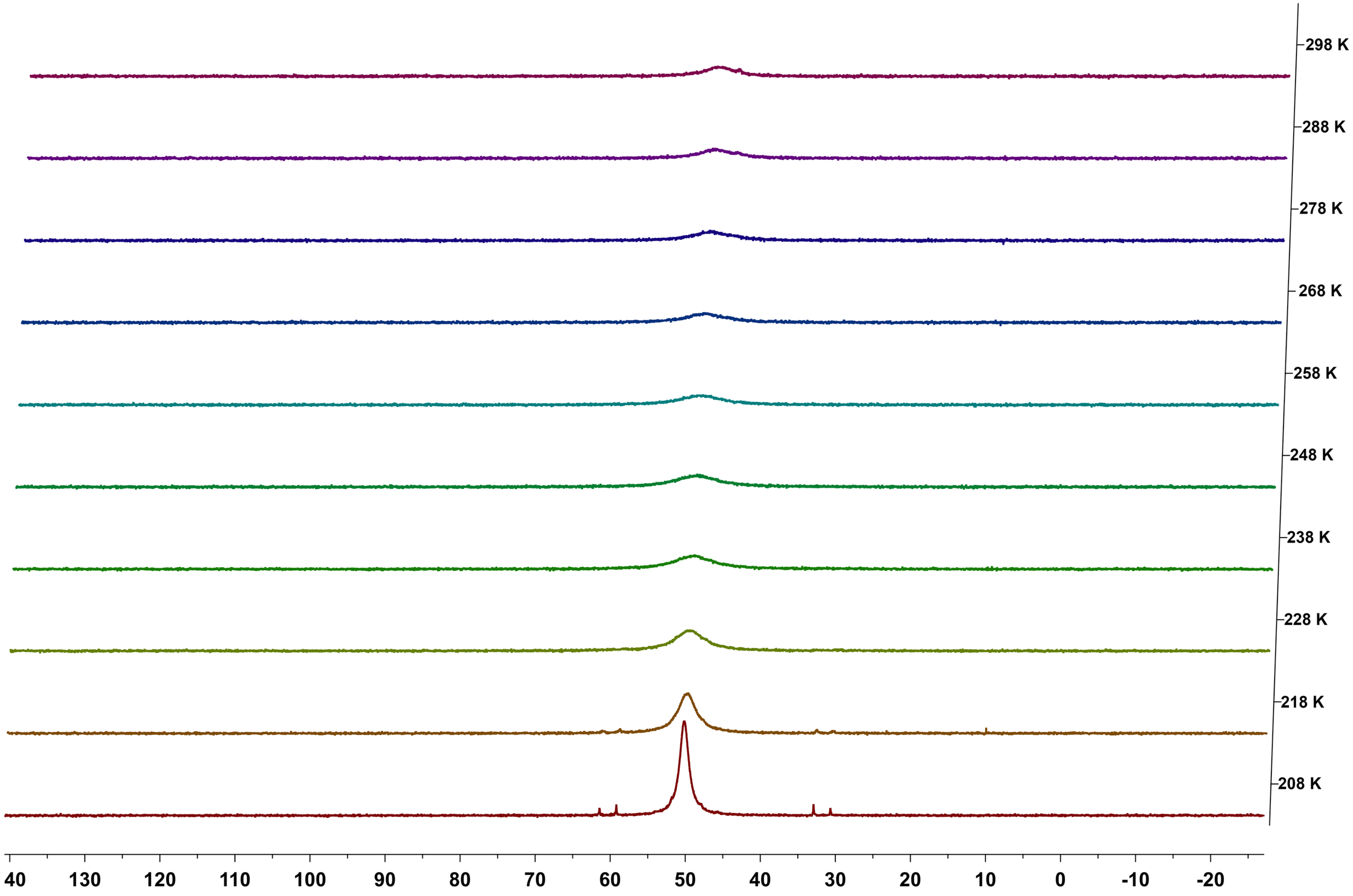

Figure S36. $^{31}P\{^{1}H\}$ NMR spectra for $[\{PC(sp^2)P\}^{tBu}PdNPh_2]^{-}[KOEt_2]^{+}$ (**5**) at different temperatures.

4.3 NMR Spectra for [{PC(sp^2)P}^{tBu}PdCH$_2$Ph]$^-$K$^+$ (**6**)

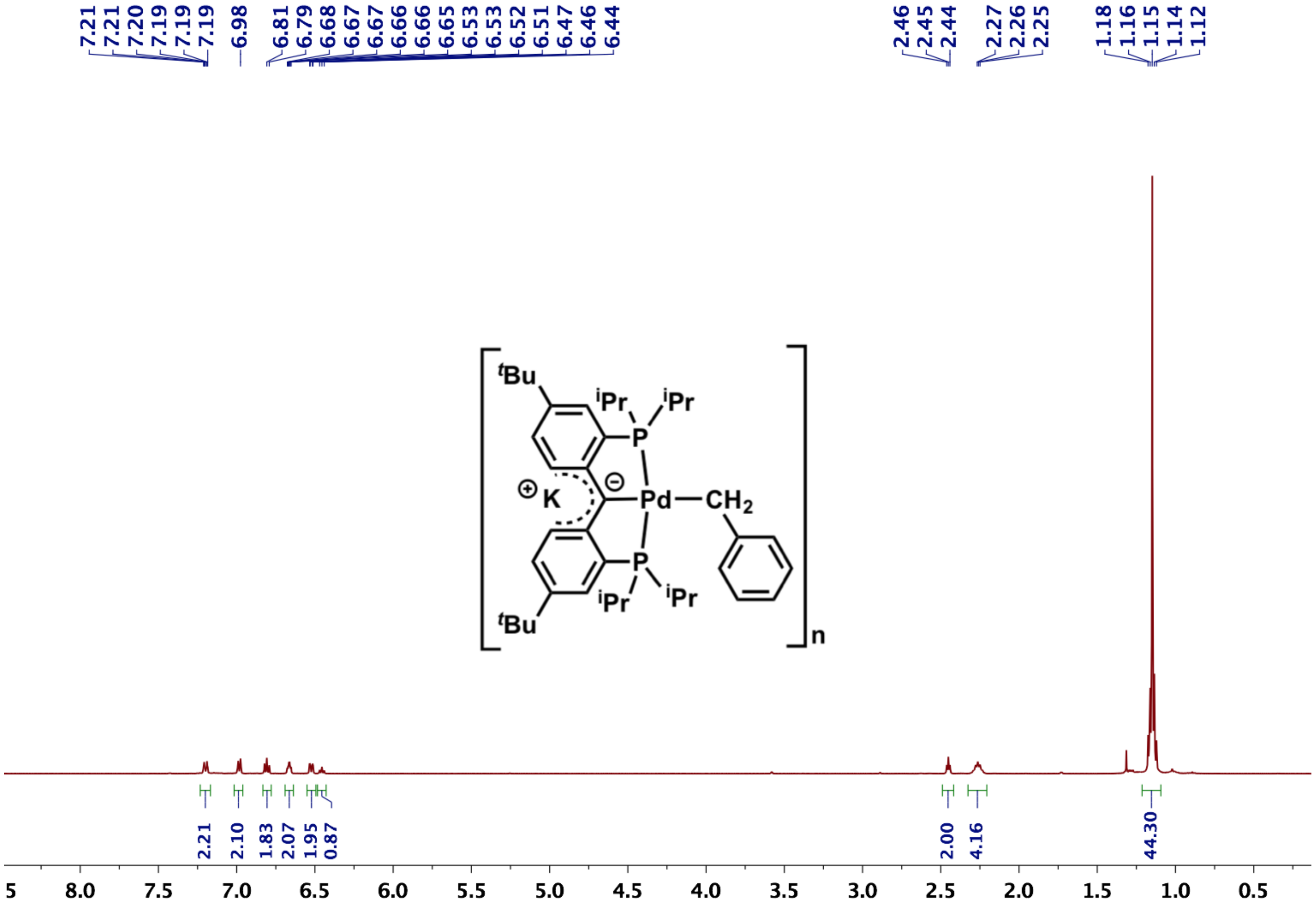

Figure S37. ^{1}H NMR spectrum for [{PC(sp^2)P}^{tBu}PdCH$_2$Ph]$^-$K$^+$ (**6**).

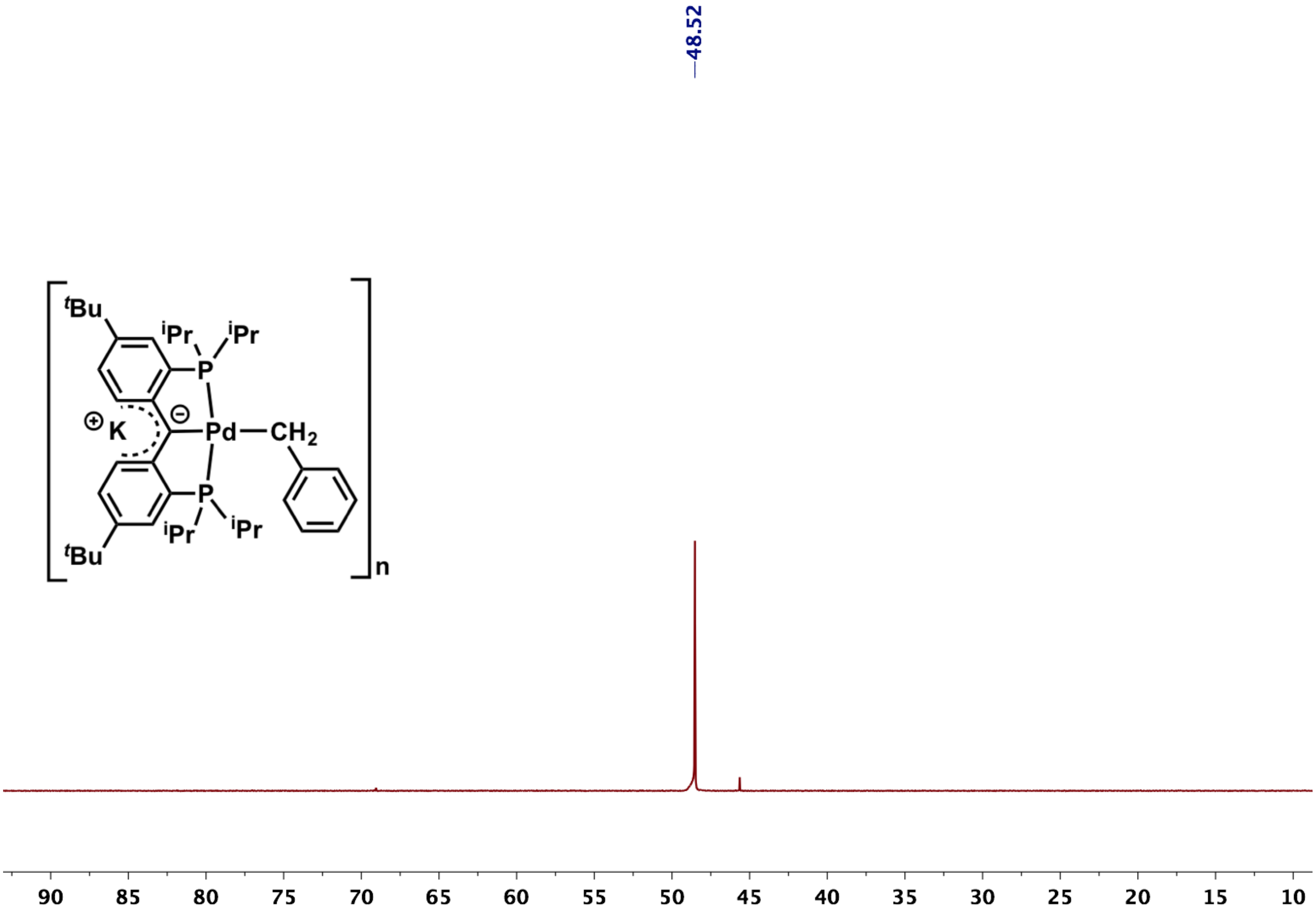

Figure S38. $^{31}P\{^1H\}$ NMR spectrum for $[\{PC(sp^2)P\}^{tBu}PdCH_2Ph]^-K^+$ (**6**).

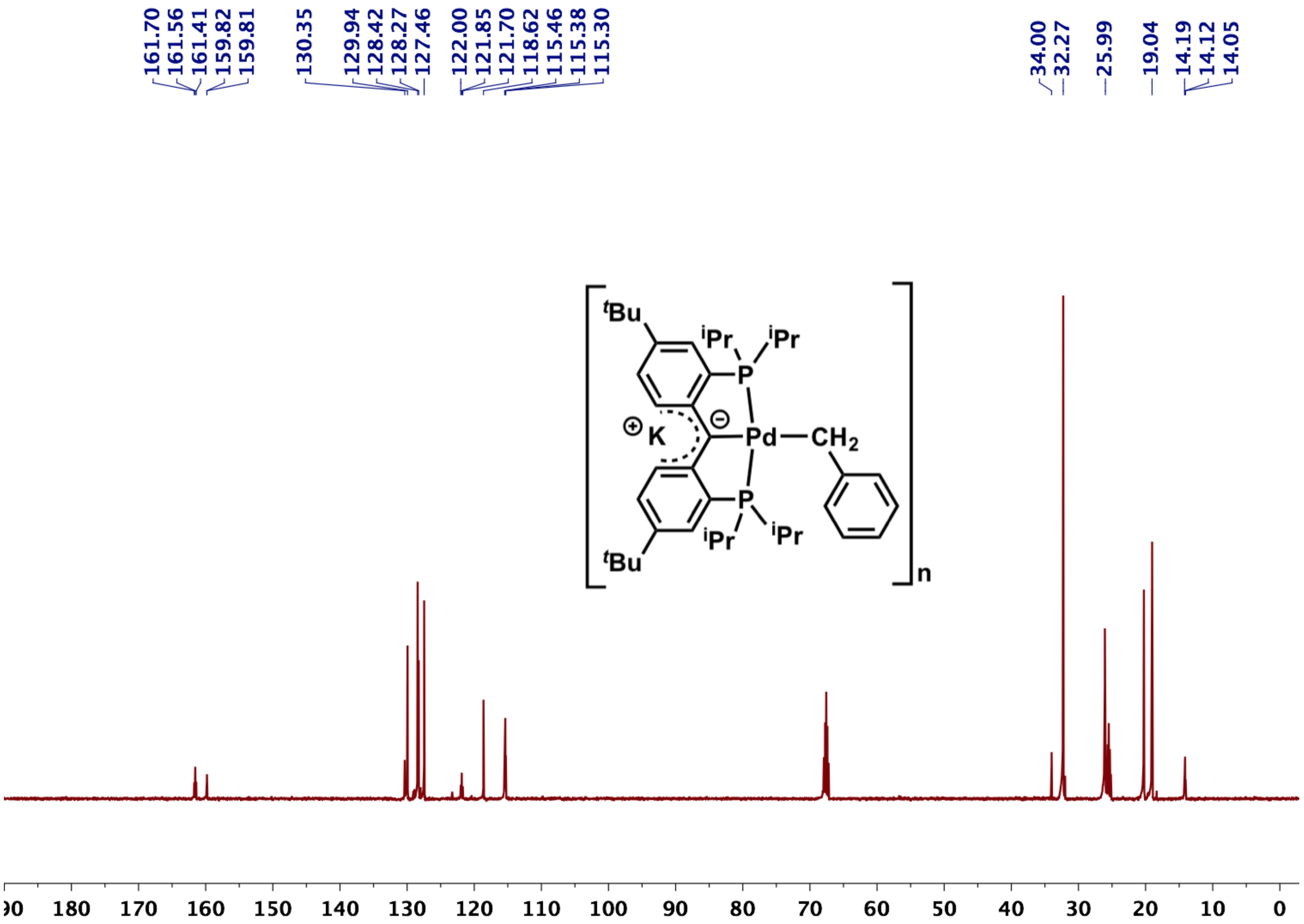

Figure S39. $^{13}C\{^1H\}$ NMR spectrum for $[\{PC(sp^2)P\}^{tBu}PdCH_2Ph]^{-}K^{+}$ (**6**).

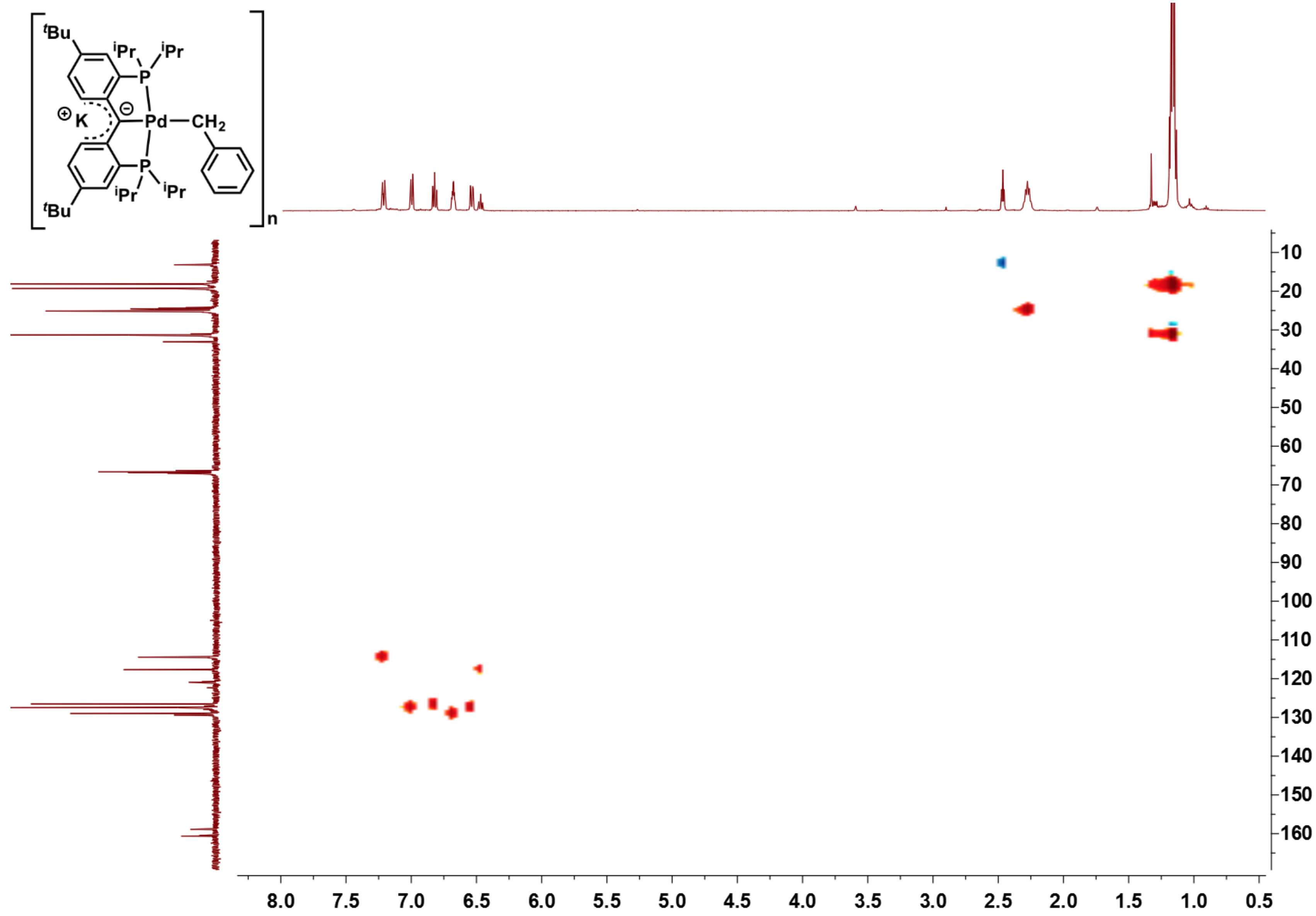

Figure S40. ^1H-^{13}C HSQC NMR spectrum for $[\{PC(sp^2)P\}^{tBu}PdCH_2Ph]^-K^+$ (**6**).

4.4 NMR Spectra for [{PC(sp^3)HP}^{tBu}PdCH$_2$Ph] (8)

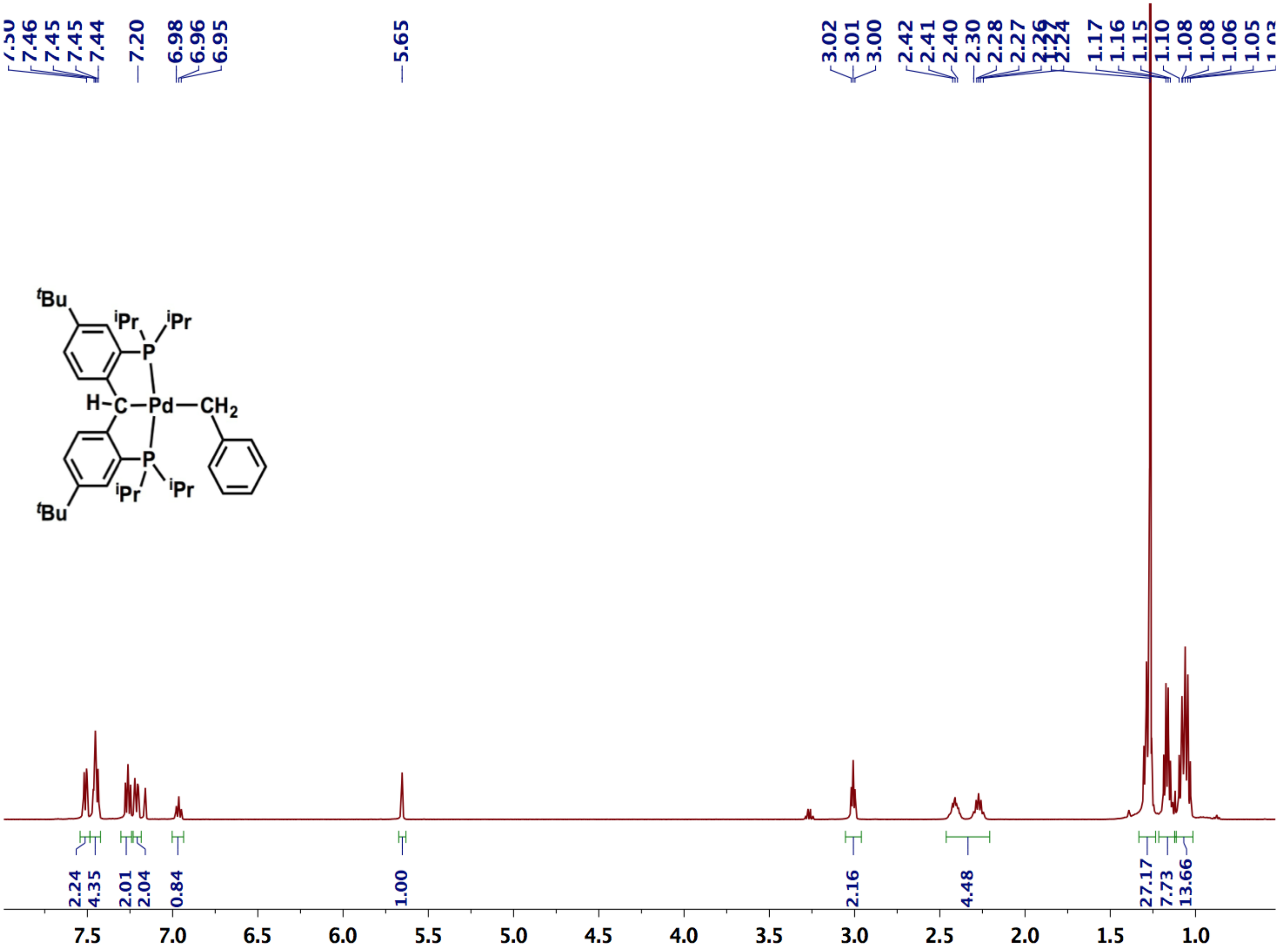

Figure S41. ^{1}H NMR spectrum for [{PC(sp^3)HP}^{tBu}PdCH$_2$Ph] (**8**).

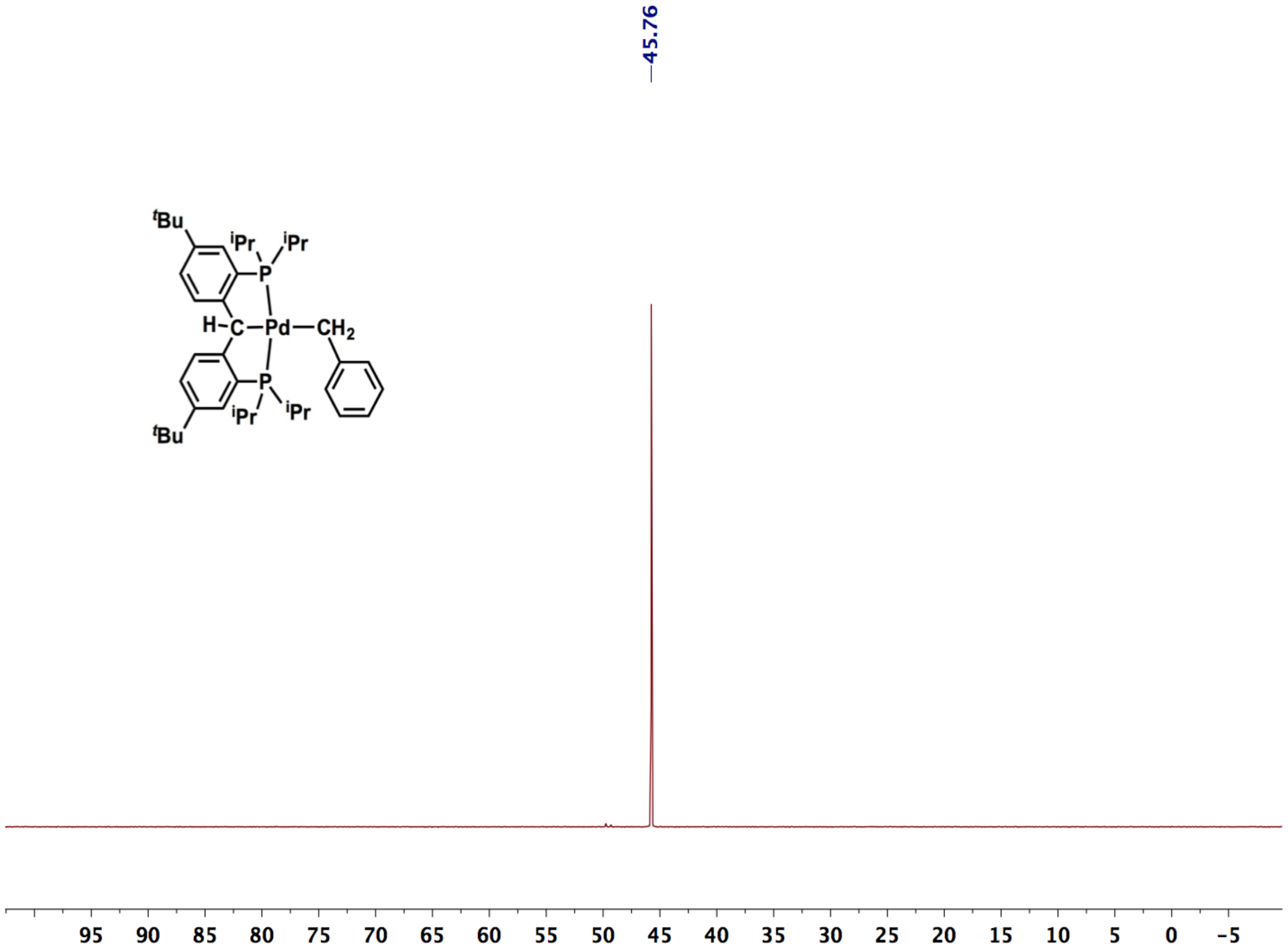

Figure S42. $^{31}P\{^1H\}$ NMR spectrum for $[\{PC(sp^3)HP\}^{tBu}PdCH_2Ph]$ (**8**).

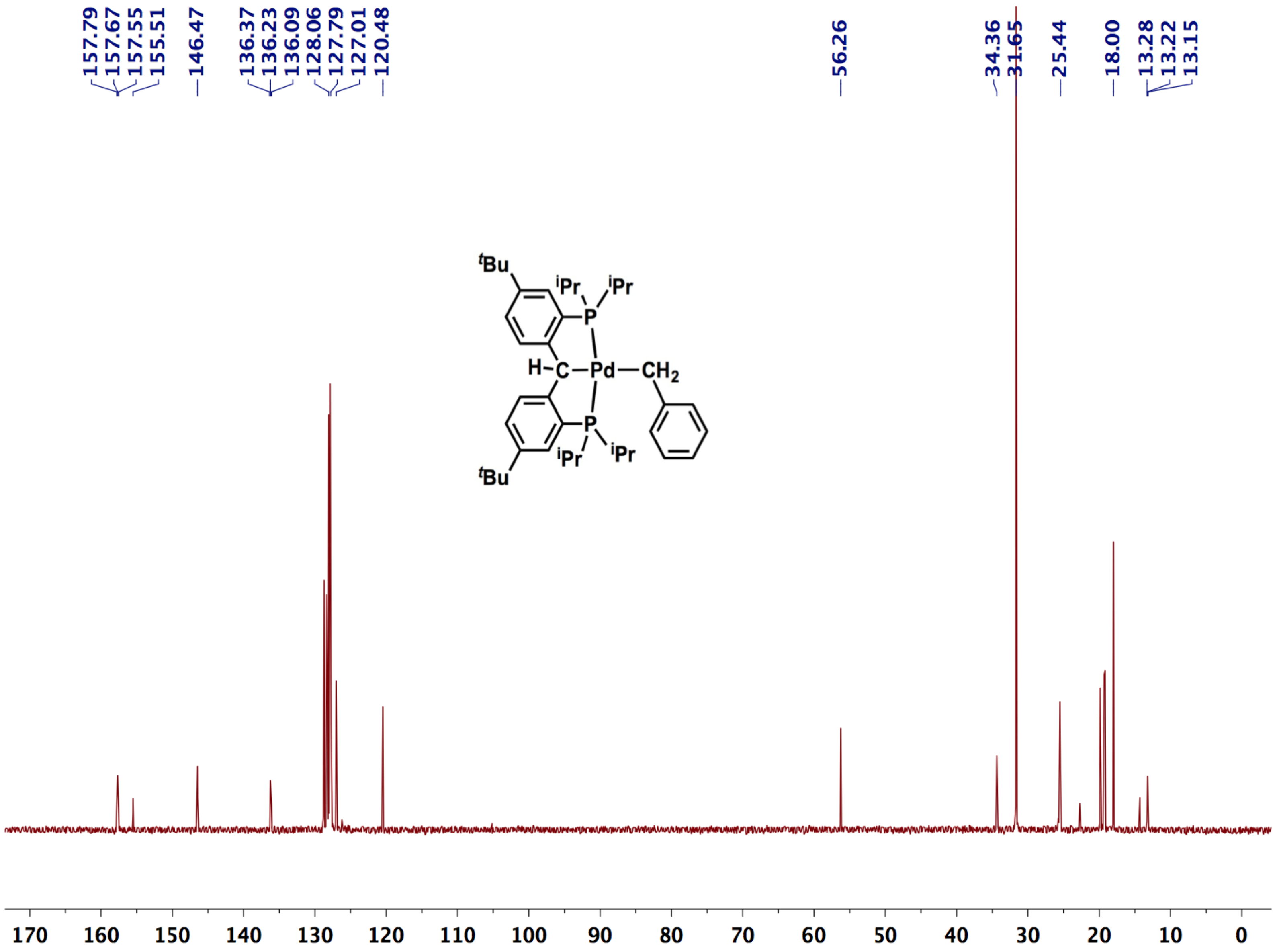

Figure S43. $^{13}C\{^1H\}$ NMR spectrum for $[\{PC(sp^3)HP\}^{tBu}PdCH_2Ph]$ (**8**).

5 Crystallographic tables

5.1 Crystal data for [{PC•(sp^2)P}^{tBu}PdNH^{p}Tol] (2)

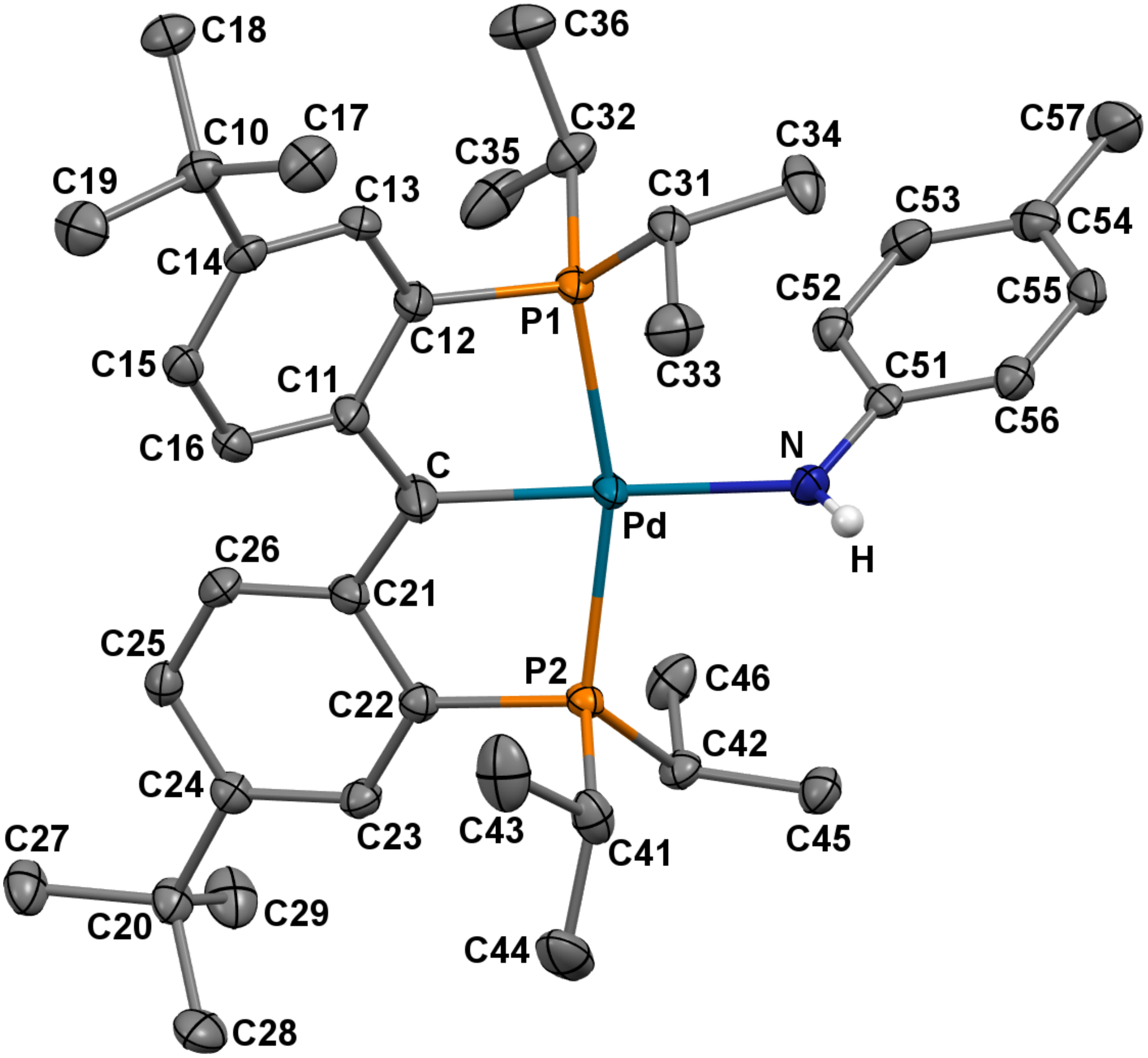

Figure S44. Thermal-ellipsoid representation of [{PC•(sp^2)P}^{tBu}PdNH^{p}Tol] (**2**) at 50% probability. Most hydrogen atoms were omitted for clarity.

Table S21. Crystal data and structure refinement for [{PC$^\bullet$(sp^2)P}^{tBu}PdNH^{p}Tol] (**2**).

Identification code:	pc43	
Empirical formula:	$C_{40}H_{60}NP_2Pd$	
Formula weight:	723.23	
Temperature:	120(2) K	
Wavelength:	0.71073 Å	
Crystal system:	Triclinic	
Space group:	$P\bar{1}$	
Unit cell dimensions:	a = 9.9475(7) Å	α = 98.9950(19)°
	b = 13.7704(10) Å	β = 100.8508(18)°
	c = 14.6585(10) Å	γ = 97.3666(18)°
Volume:	1921.9(2) $Å^3$	
Z:	2	
Density (calculated):	1.250 g·cm^{-3}	
Absorption coefficient (μ):	0.593 mm^{-1}	
F(000):	766	
Crystal size:	0.09 × 0.09 × 0.06 mm^3	
θ range for data collection:	1.89 to 25.00°	
Index ranges:	−11 ≤ h ≤ 11, −16 ≤ k ≤ 16, −17 ≤ l ≤ 17	
Reflections collected:	47002	
Independent reflections:	6761 [R_{int} = 0.0350]	
Completeness to θ = 25.00°:	100.0 %	
Absorption correction:	Semi-empirical from equivalents	
Max. and min. transmission:	0.7457 and 0.6875	
Refinement method:	Full-matrix least-squares on F^2	
Data / restraints / parameters:	6761 / 0 / 416	
Goodness-of-fit on F^2:	1.039	
Final R indices [I>2σ(I)]:	R_1 = 0.0253, wR_2 = 0.0606	
R indices (all data):	R_1 = 0.0302, wR_2 = 0.0624	
Largest diff. peak and hole:	0.818 and −0.527 e$^-$·Å^{-3}	

Table S22. Atomic coordinates and equivalent isotropic displacement parameters (Å^2) for [{PC$^•$(sp^2)P}^{tBu}PdNH^{p}Tol] (**2**). U(eq) is defined as one third of the trace of the orthogonalized U_{ij} tensor

atom	x	y	z	U(eq)
Pd	0.80704(2)	0.83723(1)	0.65165(1)	0.018(1)
P(1)	0.94622(5)	0.73444(4)	0.59103(4)	0.019(1)
P(2)	0.72522(5)	0.95588(4)	0.74369(4)	0.016(1)
C(11)	1.1011(2)	0.84841(15)	0.75360(15)	0.022(1)
C(10)	1.3847(2)	0.63478(15)	0.80883(15)	0.022(1)
C(12)	1.0976(2)	0.76051(15)	0.68804(15)	0.021(1)
C(13)	1.1935(2)	0.69736(15)	0.70509(14)	0.020(1)
C(14)	1.2931(2)	0.71367(15)	0.78937(14)	0.019(1)
C(15)	1.2961(2)	0.80055(15)	0.85418(14)	0.020(1)
C(16)	1.2045(2)	0.86679(15)	0.83653(15)	0.022(1)
C(17)	1.2916(2)	0.53418(17)	0.79930(18)	0.032(1)
C(18)	1.4784(2)	0.62452(17)	0.73701(16)	0.027(1)
C(19)	1.4750(2)	0.65969(18)	0.90832(16)	0.032(1)
C(20)	1.0087(2)	1.31771(15)	0.91878(15)	0.023(1)
C(21)	1.0034(2)	1.00972(15)	0.78150(14)	0.021(1)
C(22)	0.8807(2)	1.04791(15)	0.79158(14)	0.018(1)
C(23)	0.8849(2)	1.14537(15)	0.83526(14)	0.020(1)
C(25)	1.1306(2)	1.17412(15)	0.85831(14)	0.021(1)
C(24)	1.0097(2)	1.21086(14)	0.87106(14)	0.019(1)
C	0.9900(2)	0.90616(17)	0.73555(17)	0.033(1)
C(26)	1.1282(2)	1.07709(16)	0.81488(15)	0.023(1)
C(27)	1.1552(2)	1.37241(16)	0.96651(17)	0.031(1)
C(28)	0.9231(2)	1.31690(17)	0.99557(17)	0.033(1)
C(29)	0.9448(3)	1.37390(16)	0.84366(18)	0.034(1)
C(33)	0.8449(3)	0.57378(18)	0.66251(18)	0.034(1)
C(32)	1.0043(2)	0.75898(16)	0.48335(16)	0.025(1)
C(31)	0.8932(2)	0.59885(15)	0.57522(15)	0.021(1)
C(35)	1.0509(3)	0.87130(18)	0.4935(2)	0.041(1)
C(34)	0.7813(2)	0.55835(16)	0.48587(17)	0.032(1)
C(36)	1.1169(3)	0.70061(19)	0.45876(18)	0.035(1)
C(42)	0.5909(2)	1.02208(15)	0.69125(15)	0.020(1)
C(43)	0.7850(3)	0.8700(2)	0.90187(18)	0.042(1)
C(44)	0.6275(3)	0.99892(19)	0.91265(17)	0.039(1)
C(45)	0.4494(2)	0.95419(16)	0.66134(16)	0.025(1)
C(46)	0.6345(2)	1.06190(17)	0.60822(16)	0.029(1)
C(51)	0.58263(19)	0.74656(14)	0.47172(14)	0.017(1)
C(52)	0.6592(2)	0.79960(15)	0.41853(15)	0.021(1)
C(53)	0.6262(2)	0.78118(17)	0.32081(15)	0.026(1)
C(54)	0.5144(2)	0.71004(17)	0.26939(15)	0.026(1)

Continued on next page

Table S22. – continued from previous page

atom	x	y	x	U(eq)
C(55)	0.4375(2)	0.65787(16)	0.32107(15)	0.025(1)
C(56)	0.4698(2)	0.67466(15)	0.41869(15)	0.021(1)
C(57)	0.4784(3)	0.6920(2)	0.16280(16)	0.038(1)
N	0.61756(18)	0.76538(14)	0.56737(12)	0.020(1)
C(41)	0.6706(2)	0.91642(16)	0.84770(15)	0.026(1)
H(13)	1.1915	0.6411	0.6579	0.024
H(15)	1.3628	0.8146	0.9121	0.024
H(16)	1.2121	0.9259	0.8816	0.026
H(17A)	1.2355	0.5146	0.7348	0.047
H(17B)	1.2303	0.5403	0.8443	0.047
H(17C)	1.3495	0.4835	0.8127	0.047
H	0.567(2)	0.7349(17)	0.5891(16)	0.018(6)
H(18A)	1.4210	0.6084	0.6728	0.041
H(18B)	1.5326	0.5712	0.7477	0.041
H(18C)	1.5414	0.6875	0.7446	0.041
H(19A)	1.5321	0.6076	0.9176	0.049
H(19B)	1.4157	0.6635	0.9547	0.049
H(19C)	1.5353	0.7239	0.9166	0.049
H(23)	0.8003	1.1683	0.8410	0.024
H(25)	1.2173	1.2171	0.8802	0.025
H(26)	1.2130	1.0554	0.8074	0.028
H(27A)	1.2116	1.3765	0.9188	0.046
H(27B)	1.1976	1.3359	1.0136	0.046
H(27C)	1.1500	1.4398	0.9976	0.046
H(28A)	0.8267	1.2869	0.9665	0.050
H(28B)	0.9259	1.3854	1.0276	0.050
H(28C)	0.9618	1.2779	1.0416	0.050
H(29A)	1.0012	1.3753	0.7957	0.050
H(29B)	0.9419	1.4422	0.8735	0.050
H(29C)	0.8503	1.3401	0.8138	0.050
H(33A)	0.7621	0.6035	0.6691	0.051
H(33B)	0.9188	0.6004	0.7186	0.051
H(33C)	0.8228	0.5013	0.6560	0.051
H(32)	0.9221	0.7388	0.4295	0.031
H(31)	0.9763	0.5665	0.5691	0.026
H(35A)	1.0668	0.8863	0.4329	0.061
H(35B)	1.1370	0.8923	0.5416	0.061
H(35C)	0.9786	0.9071	0.5126	0.061
H(34A)	0.8154	0.5753	0.4309	0.047
H(34B)	0.6980	0.5880	0.4909	0.047
H(34C)	0.7588	0.4858	0.4785	0.047
H(36A)	1.0838	0.6291	0.4517	0.053

Continued on next page

Table S22. – continued from previous page

atom	x	y	x	U(eq)
H(36B)	1.2003	0.7203	0.5094	0.053
H(36C)	1.1391	0.7149	0.3994	0.053
H(42)	0.5840	1.0799	0.7398	0.024
H(43A)	0.8081	0.8156	0.8590	0.063
H(43B)	0.7527	0.8439	0.9538	0.063
H(43C)	0.8676	0.9209	0.9275	0.063
H(44A)	0.5496	1.0238	0.8772	0.058
H(44B)	0.7059	1.0535	0.9368	0.058
H(44C)	0.5993	0.9725	0.9657	0.058
H(45A)	0.3794	0.9912	0.6336	0.037
H(45B)	0.4236	0.9315	0.7168	0.037
H(45C)	0.4547	0.8964	0.6145	0.037
H(46A)	0.6435	1.0061	0.5605	0.043
H(46B)	0.7239	1.1064	0.6303	0.043
H(46C)	0.5643	1.0988	0.5804	0.043
H(52)	0.7357	0.8495	0.4507	0.025
H(53)	0.6815	0.8182	0.2878	0.031
H(55)	0.3602	0.6090	0.2883	0.030
H(56)	0.4144	0.6367	0.4510	0.025
H(57A)	0.5596	0.6763	0.1381	0.057
H(57B)	0.4505	0.7519	0.1417	0.057
H(57C)	0.4018	0.6360	0.1393	0.057
H(41)	0.5878	0.8629	0.8236	0.031

Table S23. Anisotropic displacement parameters (Å^2) for [{PC$^•$(sp^2)P}^{tBu}PdNH^{p}Tol] (**2**). The anisotropic displacement factor exponent takes the form: $-2\pi^2[h^2a^{*2}U_{11} + ... + 2hka^*b^*U_{12}]$.

atom	U_{11}	U_{22}	U_{33}	U_{23}	U_{13}	U_{12}
Pd	0.0154(1)	0.0164(1)	0.0200(1)	−0.0039(1)	0.0002(1)	0.0049(1)
P(1)	0.0168(3)	0.0149(3)	0.0223(3)	−0.0028(2)	0.0015(2)	0.0047(2)
P(2)	0.0177(3)	0.0139(2)	0.0164(3)	0.0002(2)	0.0025(2)	0.0039(2)
C(11)	0.0170(10)	0.0171(10)	0.0277(11)	−0.0027(9)	0.0032(9)	0.0022(8)
C(10)	0.0221(11)	0.0226(11)	0.0229(11)	0.0066(9)	0.0054(9)	0.0068(9)
C(12)	0.0175(10)	0.0194(10)	0.0238(11)	−0.0019(9)	0.0015(8)	0.0042(8)
C(13)	0.0196(10)	0.0182(10)	0.0210(11)	−0.0030(8)	0.0051(8)	0.0049(8)
C(14)	0.0170(10)	0.0200(10)	0.0212(11)	0.0059(8)	0.0073(8)	0.0038(8)
C(15)	0.0195(10)	0.0235(11)	0.0170(10)	0.0030(8)	0.0027(8)	0.0020(8)
C(16)	0.0214(11)	0.0193(10)	0.0226(11)	−0.0036(9)	0.0063(9)	0.0015(8)
C(17)	0.0335(13)	0.0264(12)	0.0391(14)	0.0141(10)	0.0103(11)	0.0072(10)
C(18)	0.0261(11)	0.0272(12)	0.0322(12)	0.0068(10)	0.0099(10)	0.0117(9)
C(19)	0.0337(13)	0.0362(13)	0.0283(12)	0.0085(10)	0.0013(10)	0.0140(11)
C(20)	0.0212(11)	0.0146(10)	0.0310(12)	0.0017(9)	0.0035(9)	0.0018(8)
C(21)	0.0198(10)	0.0225(11)	0.0191(10)	−0.0016(8)	0.0010(8)	0.0072(8)
C(22)	0.0181(10)	0.0176(10)	0.0181(10)	0.0027(8)	0.0034(8)	0.0033(8)
C(23)	0.0174(10)	0.0182(10)	0.0244(11)	0.0027(8)	0.0061(8)	0.0048(8)
C(25)	0.0169(10)	0.0216(11)	0.0218(11)	0.0031(9)	0.0021(8)	0.0000(8)
C(24)	0.0212(10)	0.0157(10)	0.0198(10)	0.0043(8)	0.0044(8)	0.0031(8)
C	0.0216(11)	0.0292(12)	0.0391(14)	−0.0116(10)	−0.0033(10)	0.0092(10)
C(26)	0.0167(10)	0.0271(12)	0.0260(11)	0.0004(9)	0.0044(9)	0.0075(9)
C(27)	0.0266(12)	0.0173(11)	0.0420(14)	−0.0008(10)	0.0006(10)	0.0010(9)
C(28)	0.0354(13)	0.0216(12)	0.0397(14)	−0.0075(10)	0.0119(11)	0.0039(10)
C(29)	0.0357(13)	0.0173(11)	0.0440(15)	0.0043(10)	−0.0015(11)	0.0071(10)
C(33)	0.0371(13)	0.0284(13)	0.0395(14)	0.0067(11)	0.0145(11)	0.0045(10)
C(32)	0.0225(11)	0.0271(12)	0.0300(12)	0.0086(10)	0.0080(9)	0.0082(9)
C(31)	0.0206(10)	0.0155(10)	0.0255(11)	−0.0018(8)	0.0024(9)	0.0049(8)
C(35)	0.0291(13)	0.0318(14)	0.0707(19)	0.0199(13)	0.0220(13)	0.0081(11)
C(34)	0.0320(13)	0.0169(11)	0.0374(14)	−0.0034(10)	−0.0067(10)	0.0037(9)
C(36)	0.0381(14)	0.0413(14)	0.0345(14)	0.0115(11)	0.0168(11)	0.0195(11)
C(42)	0.0170(10)	0.0194(10)	0.0239(11)	0.0030(8)	0.0045(8)	0.0065(8)
C(43)	0.0436(15)	0.0446(15)	0.0337(14)	0.0215(12)	−0.0058(12)	−0.0021(12)
C(44)	0.0459(15)	0.0431(15)	0.0240(12)	−0.0024(11)	0.0160(11)	−0.0067(12)
C(45)	0.0181(10)	0.0270(12)	0.0295(12)	0.0056(9)	0.0034(9)	0.0058(9)
C(46)	0.0226(11)	0.0343(13)	0.0345(13)	0.0177(10)	0.0060(10)	0.0093(10)
C(51)	0.0154(10)	0.0181(10)	0.0185(10)	0.0019(8)	0.0021(8)	0.0084(8)
C(52)	0.0160(10)	0.0222(11)	0.0246(11)	0.0045(9)	0.0019(8)	0.0048(8)
C(53)	0.0231(11)	0.0344(13)	0.0250(12)	0.0125(10)	0.0092(9)	0.0110(9)
C(54)	0.0251(11)	0.0366(13)	0.0194(11)	0.0041(9)	0.0029(9)	0.0158(10)
C(55)	0.0188(10)	0.0287(12)	0.0237(11)	−0.0021(9)	−0.0022(9)	0.0065(9)

Continued on next page

Table S23. – continued from previous page

**atom**	$\mathbf{U_{11}}$	$\mathbf{U_{22}}$	$\mathbf{U_{33}}$	$\mathbf{U_{23}}$	$\mathbf{U_{13}}$	$\mathbf{U_{12}}$
C(56)	0.0179(10)	0.0215(11)	0.0246(11)	0.0048(9)	0.0040(9)	0.0042(8)
C(57)	0.0341(13)	0.0604(17)	0.0210(12)	0.0068(12)	0.0027(10)	0.0164(12)
N	0.0172(9)	0.0232(9)	0.0184(9)	0.0029(8)	0.0048(8)	−0.0001(7)
C(41)	0.0293(12)	0.0243(11)	0.0207(11)	0.0056(9)	0.0020(9)	−0.0042(9)

Table S24. Distances [Å] for [{PC•(sp²)P}^{tBu}PdNH^{p}Tol] (**2**).

atom – atom	distance	atom – atom	distance
Pd – C	2.019(2)	Pd – N	2.0787(18)
Pd – P(2)	2.2841(5)	Pd – P(1)	2.2983(5)
P(1) – C(12)	1.821(2)	P(1) – C(31)	1.840(2)
P(1) – C(32)	1.844(2)	P(2) – C(22)	1.817(2)
P(2) – C(42)	1.831(2)	P(2) – C(41)	1.847(2)
C(11) – C(16)	1.402(3)	C(11) – C(12)	1.415(3)
C(11) – C	1.451(3)	C(10) – C(19)	1.524(3)
C(10) – C(14)	1.532(3)	C(10) – C(18)	1.534(3)
C(10) – C(17)	1.536(3)	C(12) – C(13)	1.386(3)
C(13) – C(14)	1.398(3)	C(13) – H(13)	0.9500
C(14) – C(15)	1.400(3)	C(15) – C(16)	1.388(3)
C(15) – H(15)	0.9500	C(16) – H(16)	0.9500
C(17) – H(17A)	0.9800	C(17) – H(17B)	0.9800
C(17) – H(17C)	0.9800	C(18) – H(18A)	0.9800
C(18) – H(18B)	0.9800	C(18) – H(18C)	0.9800
C(19) – H(19A)	0.9800	C(19) – H(19B)	0.9800
C(19) – H(19C)	0.9800	C(20) – C(24)	1.529(3)
C(20) – C(29)	1.531(3)	C(20) – C(27)	1.533(3)
C(20) – C(28)	1.535(3)	C(21) – C(26)	1.405(3)
C(21) – C(22)	1.413(3)	C(21) – C	1.457(3)
C(22) – C(23)	1.385(3)	C(23) – C(24)	1.396(3)
C(23) – H(23)	0.9500	C(25) – C(26)	1.382(3)
C(25) – C(24)	1.396(3)	C(25) – H(25)	0.9500
C(26) – H(26)	0.9500	C(27) – H(27A)	0.9800
C(27) – H(27B)	0.9800	C(27) – H(27C)	0.9800
C(28) – H(28A)	0.9800	C(28) – H(28B)	0.9800
C(28) – H(28C)	0.9800	C(29) – H(29A)	0.9800
C(29) – H(29B)	0.9800	C(29) – H(29C)	0.9800
C(33) – C(31)	1.521(3)	C(33) – H(33A)	0.9800
C(33) – H(33B)	0.9800	C(33) – H(33C)	0.9800
C(32) – C(36)	1.524(3)	C(32) – C(35)	1.532(3)
C(32) – H(32)	1.0000	C(31) – C(34)	1.526(3)
C(31) – H(31)	1.0000	C(35) – H(35A)	0.9800
C(35) – H(35B)	0.9800	C(35) – H(35C)	0.9800
C(34) – H(34A)	0.9800	C(34) – H(34B)	0.9800
C(34) – H(34C)	0.9800	C(36) – H(36A)	0.9800
C(36) – H(36B)	0.9800	C(36) – H(36C)	0.9800
C(42) – C(46)	1.522(3)	C(42) – C(45)	1.533(3)
C(42) – H(42)	1.0000	C(43) – C(41)	1.529(3)
C(43) – H(43A)	0.9800	C(43) – H(43B)	0.9800
C(43) – H(43C)	0.9800	C(44) – C(41)	1.524(3)

Continued on next page

Table S24. – continued from previous page

**atom – atom**	**distance**	**atom – atom**	**distance**
C(44) – H(44A)	0.9800	C(44) – H(44B)	0.9800
C(44) – H(44C)	0.9800	C(45) – H(45A)	0.9800
C(45) – H(45B)	0.9800	C(45) – H(45C)	0.9800
C(46) – H(46A)	0.9800	C(46) – H(46B)	0.9800
C(46) – H(46C)	0.9800	C(51) – N	1.354(3)
C(51) – C(52)	1.407(3)	C(51) – C(56)	1.411(3)
C(52) – C(53)	1.383(3)	C(52) – H(52)	0.9500
C(53) – C(54)	1.392(3)	C(53) – H(53)	0.9500
C(54) – C(55)	1.386(3)	C(54) – C(57)	1.509(3)
C(55) – C(56)	1.382(3)	C(55) – H(55)	0.9500
C(56) – H(56)	0.9500	C(57) – H(57A)	0.9800
C(57) – H(57B)	0.9800	C(57) – H(57C)	0.9800
N – H	0.76(2)	C(41) – H(41)	1.0000

Table S25. Angles [°] for [{PC$^{\bullet}$(sp^2)P}^{tBu}PdNH^{p}Tol] (**2**).

atom – atom – atom	angle	atom – atom – atom	angle
C – Pd – N	178.97(9)	C – Pd – P(2)	81.80(6)
N – Pd – P(2)	97.79(5)	C – Pd – P(1)	82.15(6)
N – Pd – P(1)	98.20(5)	P(2) – Pd – P(1)	163.689(19)
C(12) – P(1) – C(31)	103.37(9)	C(12) – P(1) – C(32)	108.03(10)
C(31) – P(1) – C(32)	106.95(10)	C(12) – P(1) – Pd	100.58(7)
C(31) – P(1) – Pd	118.47(7)	C(32) – P(1) – Pd	117.77(7)
C(22) – P(2) – C(42)	106.81(9)	C(22) – P(2) – C(41)	105.16(10)
C(42) – P(2) – C(41)	106.28(10)	C(22) – P(2) – Pd	101.61(7)
C(42) – P(2) – Pd	120.79(7)	C(41) – P(2) – Pd	114.74(7)
C(16) – C(11) – C(12)	116.58(18)	C(16) – C(11) – C	124.86(19)
C(12) – C(11) – C	118.18(18)	C(19) – C(10) – C(14)	112.17(17)
C(19) – C(10) – C(18)	108.99(18)	C(14) – C(10) – C(18)	110.09(17)
C(19) – C(10) – C(17)	107.65(18)	C(14) – C(10) – C(17)	108.99(17)
C(18) – C(10) – C(17)	108.87(18)	C(13) – C(12) – C(11)	121.13(19)
C(13) – C(12) – P(1)	125.28(15)	C(11) – C(12) – P(1)	113.18(15)
C(12) – C(13) – C(14)	122.21(18)	C(12) – C(13) – H(13)	118.9
C(14) – C(13) – H(13)	118.9	C(13) – C(14) – C(15)	116.42(18)
C(13) – C(14) – C(10)	119.61(18)	C(15) – C(14) – C(10)	123.85(18)
C(16) – C(15) – C(14)	122.09(19)	C(16) – C(15) – H(15)	119.0
C(14) – C(15) – H(15)	119.0	C(15) – C(16) – C(11)	121.46(18)
C(15) – C(16) – H(16)	119.3	C(11) – C(16) – H(16)	119.3
C(10) – C(17) – H(17A)	109.5	C(10) – C(17) – H(17B)	109.5
H(17A) – C(17) – H(17B)	109.5	C(10) – C(17) – H(17C)	109.5
H(17A) – C(17) – H(17C)	109.5	H(17B) – C(17) – H(17C)	109.5
C(10) – C(18) – H(18A)	109.5	C(10) – C(18) – H(18B)	109.5
H(18A) – C(18) – H(18B)	109.5	C(10) – C(18) – H(18C)	109.5
H(18A) – C(18) – H(18C)	109.5	H(18B) – C(18) – H(18C)	109.5
C(10) – C(19) – H(19A)	109.5	C(10) – C(19) – H(19B)	109.5
H(19A) – C(19) – H(19B)	109.5	C(10) – C(19) – H(19C)	109.5
H(19A) – C(19) – H(19C)	109.5	H(19B) – C(19) – H(19C)	109.5
C(24) – C(20) – C(29)	108.57(17)	C(24) – C(20) – C(27)	111.92(17)
C(29) – C(20) – C(27)	109.06(18)	C(24) – C(20) – C(28)	109.84(17)
C(29) – C(20) – C(28)	109.99(19)	C(27) – C(20) – C(28)	107.45(18)
C(26) – C(21) – C(22)	116.22(18)	C(26) – C(21) – C	125.83(19)
C(22) – C(21) – C	117.93(19)	C(23) – C(22) – C(21)	121.35(18)
C(23) – C(22) – P(2)	125.91(15)	C(21) – C(22) – P(2)	112.74(15)
C(22) – C(23) – C(24)	122.05(18)	C(22) – C(23) – H(23)	119.0
C(24) – C(23) – H(23)	119.0	C(26) – C(25) – C(24)	122.06(19)
C(26) – C(25) – H(25)	119.0	C(24) – C(25) – H(25)	119.0
C(23) – C(24) – C(25)	116.60(18)	C(23) – C(24) – C(20)	119.99(17)
C(25) – C(24) – C(20)	123.39(18)	C(11) – C – C(21)	121.98(19)

Continued on next page

Table S25. – continued from previous page

atom – atom – atom	angle	atom – atom – atom	angle
C(11) – C – Pd	118.57(15)	C(21) – C – Pd	119.30(15)
C(25) – C(26) – C(21)	121.67(19)	C(25) – C(26) – H(26)	119.2
C(21) – C(26) – H(26)	119.2	C(20) – C(27) – H(27A)	109.5
C(20) – C(27) – H(27B)	109.5	H(27A) – C(27) – H(27B)	109.5
C(20) – C(27) – H(27C)	109.5	H(27A) – C(27) – H(27C)	109.5
H(27B) – C(27) – H(27C)	109.5	C(20) – C(28) – H(28A)	109.5
C(20) – C(28) – H(28B)	109.5	H(28A) – C(28) – H(28B)	109.5
C(20) – C(28) – H(28C)	109.5	H(28A) – C(28) – H(28C)	109.5
H(28B) – C(28) – H(28C)	109.5	C(20) – C(29) – H(29A)	109.5
C(20) – C(29) – H(29B)	109.5	H(29A) – C(29) – H(29B)	109.5
C(20) – C(29) – H(29C)	109.5	H(29A) – C(29) – H(29C)	109.5
H(29B) – C(29) – H(29C)	109.5	C(31) – C(33) – H(33A)	109.5
C(31) – C(33) – H(33B)	109.5	H(33A) – C(33) – H(33B)	109.5
C(31) – C(33) – H(33C)	109.5	H(33A) – C(33) – H(33C)	109.5
H(33B) – C(33) – H(33C)	109.5	C(36) – C(32) – C(35)	111.24(19)
C(36) – C(32) – P(1)	114.12(16)	C(35) – C(32) – P(1)	108.91(16)
C(36) – C(32) – H(32)	107.4	C(35) – C(32) – H(32)	107.4
P(1) – C(32) – H(32)	107.4	C(33) – C(31) – C(34)	111.27(19)
C(33) – C(31) – P(1)	109.42(14)	C(34) – C(31) – P(1)	111.89(15)
C(33) – C(31) – H(31)	108.0	C(34) – C(31) – H(31)	108.0
P(1) – C(31) – H(31)	108.0	C(32) – C(35) – H(35A)	109.5
C(32) – C(35) – H(35B)	109.5	H(35A) – C(35) – H(35B)	109.5
C(32) – C(35) – H(35C)	109.5	H(35A) – C(35) – H(35C)	109.5
H(35B) – C(35) – H(35C)	109.5	C(31) – C(34) – H(34A)	109.5
C(31) – C(34) – H(34B)	109.5	H(34A) – C(34) – H(34B)	109.5
C(31) – C(34) – H(34C)	109.5	H(34A) – C(34) – H(34C)	109.5
H(34B) – C(34) – H(34C)	109.5	C(32) – C(36) – H(36A)	109.5
C(32) – C(36) – H(36B)	109.5	H(36A) – C(36) – H(36B)	109.5
C(32) – C(36) – H(36C)	109.5	H(36A) – C(36) – H(36C)	109.5
H(36B) – C(36) – H(36C)	109.5	C(46) – C(42) – C(45)	111.79(18)
C(46) – C(42) – P(2)	108.71(14)	C(45) – C(42) – P(2)	111.01(14)
C(46) – C(42) – H(42)	108.4	C(45) – C(42) – H(42)	108.4
P(2) – C(42) – H(42)	108.4	C(41) – C(43) – H(43A)	109.5
C(41) – C(43) – H(43B)	109.5	H(43A) – C(43) – H(43B)	109.5
C(41) – C(43) – H(43C)	109.5	H(43A) – C(43) – H(43C)	109.5
H(43B) – C(43) – H(43C)	109.5	C(41) – C(44) – H(44A)	109.5
C(41) – C(44) – H(44B)	109.5	H(44A) – C(44) – H(44B)	109.5
C(41) – C(44) – H(44C)	109.5	H(44A) – C(44) – H(44C)	109.5
H(44B) – C(44) – H(44C)	109.5	C(42) – C(45) – H(45A)	109.5
C(42) – C(45) – H(45B)	109.5	H(45A) – C(45) – H(45B)	109.5
C(42) – C(45) – H(45C)	109.5	H(45A) – C(45) – H(45C)	109.5
H(45B) – C(45) – H(45C)	109.5	C(42) – C(46) – H(46A)	109.5

Continued on next page

Table S25. – continued from previous page

atom – atom – atom	angle	atom – atom – atom	angle
C(42) – C(46) – H(46B)	109.5	H(46A) – C(46) – H(46B)	109.5
C(42) – C(46) – H(46C)	109.5	H(46A) – C(46) – H(46C)	109.5
H(46B) – C(46) – H(46C)	109.5	N – C(51) – C(52)	120.79(19)
N – C(51) – C(56)	123.67(19)	C(52) – C(51) – C(56)	115.54(18)
C(53) – C(52) – C(51)	121.8(2)	C(53) – C(52) – H(52)	119.1
C(51) – C(52) – H(52)	119.1	C(52) – C(53) – C(54)	122.0(2)
C(52) – C(53) – H(53)	119.0	C(54) – C(53) – H(53)	119.0
C(55) – C(54) – C(53)	116.66(19)	C(55) – C(54) – C(57)	122.0(2)
C(53) – C(54) – C(57)	121.3(2)	C(56) – C(55) – C(54)	122.1(2)
C(56) – C(55) – H(55)	118.9	C(54) – C(55) – H(55)	118.9
C(55) – C(56) – C(51)	121.8(2)	C(55) – C(56) – H(56)	119.1
C(51) – C(56) – H(56)	119.1	C(54) – C(57) – H(57A)	109.5
C(54) – C(57) – H(57B)	109.5	H(57A) – C(57) – H(57B)	109.5
C(54) – C(57) – H(57C)	109.5	H(57A) – C(57) – H(57C)	109.5
H(57B) – C(57) – H(57C)	109.5	C(51) – N – Pd	126.94(15)
C(51) – N – H	112.3(18)	Pd – N – H	119.0(17)
C(44) – C(41) – C(43)	111.3(2)	C(44) – C(41) – P(2)	114.06(16)
C(43) – C(41) – P(2)	109.61(16)	C(44) – C(41) – H(41)	107.2
C(43) – C(41) – H(41)	107.2	P(2) – C(41) – H(41)	107.2

5.2 Crystal data for [{PC•(sp^2)P}^{tBu}PdNPh$_2$] (3)

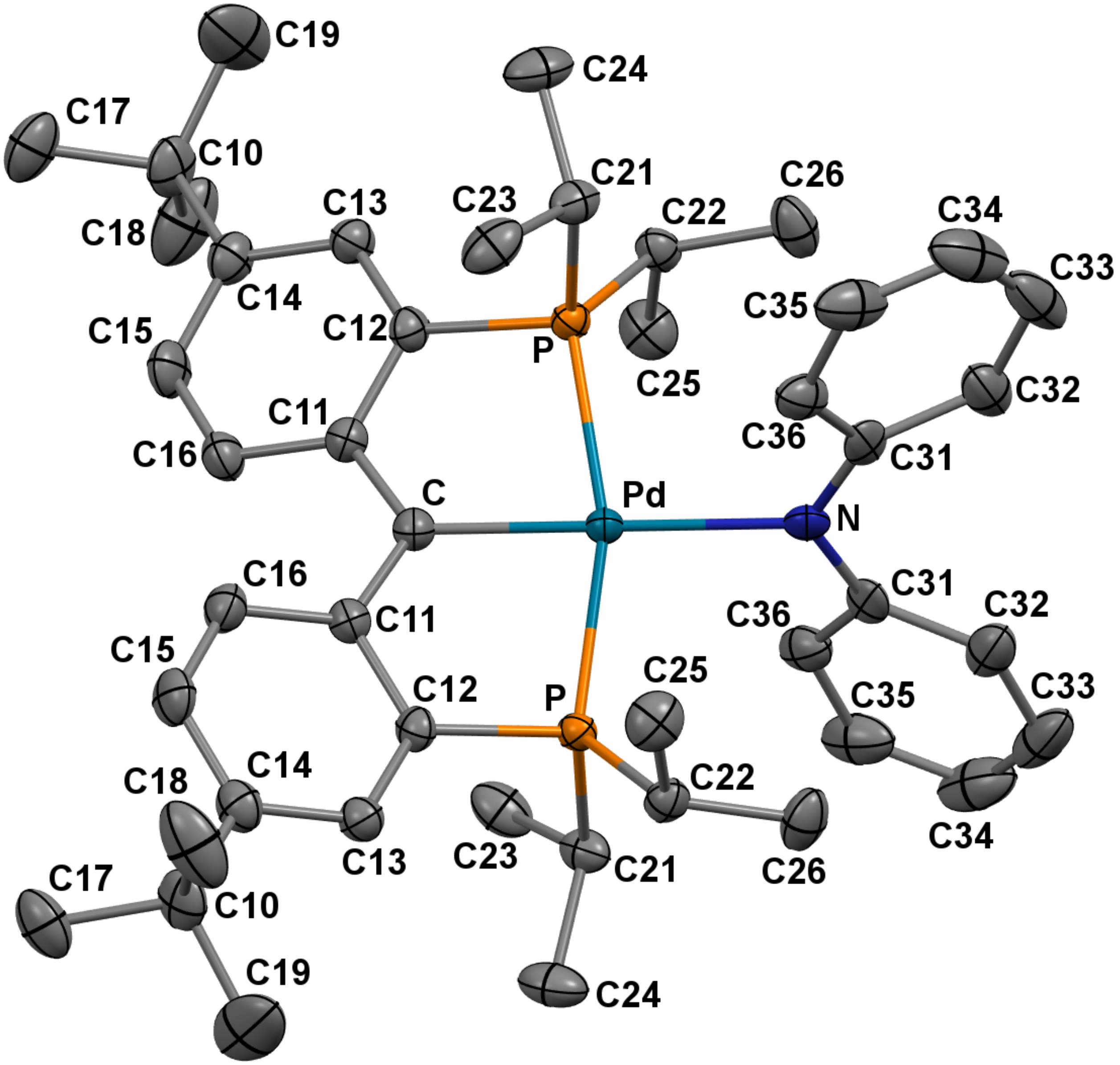

Figure S45. Thermal-ellipsoid representation of [{PC•(sp^2)P}^{tBu}PdNPh$_2$] (**3**) at 50% probability. Hydrogen atoms were omitted for clarity.

Table S26. Crystal data and structure refinement for [{PC$^{\bullet}$(sp^2)P}^{tBu}PdNPh$_2$] (**3**).

Identification code:	pc40b	
Empirical formula:	$C_{45}H_{62}NP_2Pd$	
Formula weight:	785.30	
Temperature:	120(2) K	
Wavelength:	0.71073 Å	
Crystal system:	Monoclinic	
Space group:	$C2/c$	
Unit cell dimensions:	a = 20.2994(14) Å	α = 90°
	b = 17.5268(14) Å	β = 123.775(3)°
	c = 15.9561(16) Å	γ = 90°
Volume:	4718.8(7) Å^3	
Z:	4	
Density (calculated):	1.105 g·cm^{-3}	
Absorption coefficient (μ):	0.488 mm^{-1}	
F(000):	1660	
Crystal size:	0.12 × 0.10 × 0.08 mm^3	
θ range for data collection:	1.68 to 25.00°	
Index ranges:	$-24 \le h \le 24$, $-20 \le k \le 20$, $-18 \le l \le 18$	
Reflections collected:	50505	
Independent reflections:	4154 [R_{int} = 0.0474]	
Completeness to θ = 25.00°:	100.0 %	
Absorption correction:	Semi-empirical from equivalents	
Max. and min. transmission:	0.7454 and 0.6915	
Refinement method:	Full-matrix least-squares on F^2	
Data / restraints / parameters:	4154 / 0 / 230	
Goodness-of-fit on F^2:	1.059	
Final R indices [I>2σ(I)]:	R_1 = 0.0246, wR_2 = 0.0587	
R indices (all data):	R_1 = 0.0300, wR_2 = 0.0604	
Largest diff. peak and hole:	0.481 and −0.418 e$^-$·Å^{-3}	

Table S27. Atomic coordinates and equivalent isotropic displacement parameters ($Å^2$) for [{PC•(sp^2)P}^{tBu}PdNPh_2] (**3**). U(eq) is defined as one third of the trace of the orthogonalized U_{ij} tensor

**atom**	**x**	**y**	**z**	**U(eq)**
P	0.63131(3)	0.45974(2)	0.37636(3)	0.019(1)
N	0.5000	0.31863(12)	0.2500	0.025(1)
C	0.5000	0.55672(14)	0.2500	0.022(1)
Pd	0.5000	0.44123(1)	0.2500	0.018(1)
C(11)	0.57372(10)	0.59663(10)	0.28824(13)	0.022(1)
C(10)	0.80519(12)	0.70037(11)	0.39744(16)	0.035(1)
C(12)	0.64563(11)	0.55754(10)	0.35315(13)	0.022(1)
C(13)	0.71865(11)	0.59130(11)	0.39028(14)	0.025(1)
C(14)	0.72503(11)	0.66519(11)	0.36329(14)	0.029(1)
C(15)	0.65382(12)	0.70351(11)	0.29699(15)	0.032(1)
C(16)	0.58089(12)	0.67163(11)	0.26115(14)	0.028(1)
C(17)	0.80785(14)	0.78470(12)	0.42291(18)	0.044(1)
C(18)	0.81714(16)	0.69292(15)	0.3115(2)	0.060(1)
C(19)	0.87322(14)	0.66097(17)	0.4919(2)	0.073(1)
C(21)	0.65540(11)	0.45620(11)	0.50644(13)	0.026(1)
C(22)	0.71085(11)	0.40597(10)	0.38065(14)	0.024(1)
C(23)	0.59758(13)	0.50631(13)	0.51392(16)	0.037(1)
C(24)	0.74111(12)	0.47677(14)	0.58793(15)	0.040(1)
C(25)	0.70521(13)	0.41267(13)	0.28225(16)	0.037(1)
C(26)	0.71066(12)	0.32295(11)	0.40863(17)	0.036(1)
C(31)	0.48248(10)	0.28236(11)	0.16480(15)	0.028(1)
C(32)	0.44830(11)	0.20830(12)	0.13526(17)	0.039(1)
C(33)	0.42575(13)	0.17757(15)	0.0427(2)	0.054(1)
C(34)	0.43374(14)	0.21792(17)	−0.0255(2)	0.057(1)
C(35)	0.46666(13)	0.28965(15)	0.00073(16)	0.046(1)
C(36)	0.49112(11)	0.32041(12)	0.09395(14)	0.032(1)
H(13)	0.7656	0.5633	0.4353	0.030
H(15)	0.6560	0.7536	0.2759	0.039
H(16)	0.5344	0.7005	0.2173	0.034
H(17A)	0.7658	0.8121	0.3634	0.066
H(17B)	0.8594	0.8061	0.4437	0.066
H(17C)	0.8001	0.7901	0.4780	0.066
H(18A)	0.8198	0.6388	0.2981	0.090
H(18B)	0.8667	0.7182	0.3303	0.090
H(18C)	0.7726	0.7170	0.2507	0.090
H(19A)	0.8779	0.6082	0.4757	0.109
H(19B)	0.8625	0.6613	0.5447	0.109
H(19C)	0.9228	0.6882	0.5159	0.109
H(21)	0.6467	0.4025	0.5193	0.032

Continued on next page

Table S27. – continued from previous page

atom	x	y	x	U(eq)
H(22)	0.7625	0.4289	0.4348	0.029
H(23A)	0.6074	0.5600	0.5073	0.056
H(23B)	0.6050	0.4983	0.5794	0.056
H(23C)	0.5432	0.4929	0.4599	0.056
H(24A)	0.7770	0.4413	0.5847	0.060
H(24B)	0.7492	0.4732	0.6544	0.060
H(24C)	0.7521	0.5290	0.5770	0.060
H(25A)	0.6559	0.3889	0.2281	0.055
H(25B)	0.7506	0.3868	0.2885	0.055
H(25C)	0.7055	0.4667	0.2665	0.055
H(26A)	0.6621	0.2981	0.3547	0.055
H(26B)	0.7130	0.3206	0.4716	0.055
H(26C)	0.7568	0.2967	0.4176	0.055
H(32)	0.4408	0.1794	0.1796	0.047
H(33)	0.4043	0.1275	0.0258	0.065
H(34)	0.4169	0.1966	−0.0892	0.069
H(35)	0.4727	0.3183	−0.0451	0.055
H(36)	0.5149	0.3696	0.1106	0.039

Table S28. Anisotropic displacement parameters (Å^2) for [{PC$^\bullet$(sp^2)P}^{tBu}PdNPh$_2$] (**3**). The anisotropic displacement factor exponent takes the form: $-2\pi^2[h^2a^{*2}U_{11} + ...+ 2hka^*b^*U_{12}]$.

**atom**	**U$_{11}$**	**U$_{22}$**	**U$_{33}$**	**U$_{23}$**	**U$_{13}$**	**U$_{12}$**
P	0.0158(2)	0.0192(2)	0.0195(2)	0.0015(2)	0.0086(2)	−0.0017(2)
N	0.0133(10)	0.0252(12)	0.0225(11)	0.000	0.0051(9)	0.000
C	0.0230(13)	0.0201(13)	0.0215(12)	0.000	0.0112(11)	0.000
Pd	0.0154(1)	0.0165(1)	0.0184(1)	0.000	0.0078(1)	0.000
C(11)	0.0229(10)	0.0206(9)	0.0229(9)	−0.0009(7)	0.0126(8)	−0.0009(7)
C(10)	0.0325(11)	0.0284(11)	0.0495(13)	0.0020(9)	0.0266(10)	−0.0090(9)
C(12)	0.0242(10)	0.0199(9)	0.0228(9)	0.0019(7)	0.0138(8)	−0.0023(8)
C(13)	0.0230(10)	0.0254(10)	0.0264(9)	0.0019(8)	0.0130(8)	−0.0018(8)
C(14)	0.0305(11)	0.0266(10)	0.0323(10)	−0.0003(8)	0.0196(9)	−0.0055(8)
C(15)	0.0376(12)	0.0226(10)	0.0390(11)	0.0063(8)	0.0224(10)	−0.0046(9)
C(16)	0.0306(11)	0.0222(10)	0.0305(10)	0.0052(8)	0.0161(9)	0.0031(8)
C(17)	0.0465(14)	0.0378(13)	0.0588(14)	−0.0127(11)	0.0357(12)	−0.0189(11)
C(18)	0.0634(17)	0.0557(16)	0.093(2)	−0.0278(15)	0.0634(17)	−0.0282(14)
C(19)	0.0291(13)	0.0628(18)	0.096(2)	0.0280(16)	0.0159(14)	−0.0176(12)
C(21)	0.0255(10)	0.0317(11)	0.0210(9)	0.0003(8)	0.0124(8)	−0.0068(8)
C(22)	0.0189(9)	0.0240(10)	0.0285(10)	0.0040(8)	0.0123(8)	0.0010(8)
C(23)	0.0391(12)	0.0459(13)	0.0336(11)	−0.0131(10)	0.0245(10)	−0.0111(10)
C(24)	0.0312(12)	0.0587(14)	0.0223(10)	−0.0023(10)	0.0105(9)	−0.0106(11)
C(25)	0.0375(12)	0.0406(12)	0.0403(12)	0.0022(10)	0.0269(10)	0.0051(10)
C(26)	0.0302(11)	0.0285(11)	0.0555(13)	0.0106(10)	0.0267(11)	0.0081(9)
C(31)	0.0149(9)	0.0222(10)	0.0386(11)	−0.0020(8)	0.0100(8)	0.0091(7)
C(32)	0.0222(10)	0.0309(11)	0.0532(13)	−0.0117(10)	0.0145(10)	0.0016(9)
C(33)	0.0248(12)	0.0448(14)	0.0731(18)	−0.0340(13)	0.0154(12)	−0.0022(10)
C(34)	0.0331(13)	0.0748(19)	0.0468(14)	−0.0314(14)	0.0118(12)	0.0114(13)
C(35)	0.0331(12)	0.0638(16)	0.0341(12)	−0.0087(11)	0.0144(10)	0.0180(11)
C(36)	0.0244(10)	0.0346(11)	0.0301(10)	−0.0048(9)	0.0106(9)	0.0096(8)

Table S29. Distances [Å] for [{PC$^{\bullet}$(sp^2)P}^{tBu}PdNPh$_2$] (**3**).

**atom – atom**	**distance**	**atom – atom**	**distance**
P – C(12)	1.8110(17)	P – C(22)	1.8376(18)
P – C(21)	1.8500(18)	P – Pd	2.3021(5)
N – C(31)	1.357(2)	N – C(31)#1	1.357(2)
N – Pd	2.149(2)	C – C(11)	1.444(2)
C – C(11)#1	1.444(2)	C – Pd	2.024(2)
Pd – P#1	2.3021(5)	C(11) – C(12)	1.412(3)
C(11) – C(16)	1.416(3)	C(10) – C(17)	1.526(3)
C(10) – C(18)	1.526(3)	C(10) – C(14)	1.529(3)
C(10) – C(19)	1.530(3)	C(12) – C(13)	1.386(3)
C(13) – C(14)	1.393(3)	C(13) – H(13)	0.9500
C(14) – C(15)	1.400(3)	C(15) – C(16)	1.375(3)
C(15) – H(15)	0.9500	C(16) – H(16)	0.9500
C(17) – H(17A)	0.9800	C(17) – H(17B)	0.9800
C(17) – H(17C)	0.9800	C(18) – H(18A)	0.9800
C(18) – H(18B)	0.9800	C(18) – H(18C)	0.9800
C(19) – H(19A)	0.9800	C(19) – H(19B)	0.9800
C(19) – H(19C)	0.9800	C(21) – C(23)	1.523(3)
C(21) – C(24)	1.527(3)	C(21) – H(21)	1.0000
C(22) – C(25)	1.514(3)	C(22) – C(26)	1.523(3)
C(22) – H(22)	1.0000	C(23) – H(23A)	0.9800
C(23) – H(23B)	0.9800	C(23) – H(23C)	0.9800
C(24) – H(24A)	0.9800	C(24) – H(24B)	0.9800
C(24) – H(24C)	0.9800	C(25) – H(25A)	0.9800
C(25) – H(25B)	0.9800	C(25) – H(25C)	0.9800
C(26) – H(26A)	0.9800	C(26) – H(26B)	0.9800
C(26) – H(26C)	0.9800	C(31) – C(36)	1.405(3)
C(31) – C(32)	1.423(3)	C(32) – C(33)	1.389(3)
C(32) – H(32)	0.9500	C(33) – C(34)	1.381(4)
C(33) – H(33)	0.9500	C(34) – C(35)	1.375(4)
C(34) – H(34)	0.9500	C(35) – C(36)	1.389(3)
C(35) – H(35)	0.9500	C(36) – H(36)	0.9500

Symmetry transformations used to generate equivalent atoms: #1 $-x + 1, y, -z + \frac{1}{2}$

Table S30. Angles [°] for [{PC•(sp^2)P}^{tBu}PdNPh$_2$] (**3**).

atom – atom – atom	angle	atom – atom – atom	angle
C(12) – P – C(22)	104.80(8)	C(12) – P – C(21)	106.78(8)
C(22) – P – C(21)	105.20(9)	C(12) – P – Pd	101.17(6)
C(22) – P – Pd	121.73(6)	C(21) – P – Pd	115.66(6)
C(31) – N – C(31)#1	124.1(2)	C(31) – N – Pd	117.94(12)
C(31)#1 – N – Pd	117.94(12)	C(11) – C – C(11)#1	122.1(2)
C(11) – C – Pd	118.97(11)	C(11)#1 – C – Pd	118.97(11)
C – Pd – N	180.0	C – Pd – P	81.900(12)
N – Pd – P	98.100(12)	C – Pd – P#1	81.900(12)
N – Pd – P#1	98.100(12)	P – Pd – P#1	163.80(2)
C(12) – C(11) – C(16)	115.76(16)	C(12) – C(11) – C	118.75(16)
C(16) – C(11) – C	125.36(17)	C(17) – C(10) – C(18)	108.82(18)
C(17) – C(10) – C(14)	111.02(17)	C(18) – C(10) – C(14)	108.33(18)
C(17) – C(10) – C(19)	107.2(2)	C(18) – C(10) – C(19)	109.9(2)
C(14) – C(10) – C(19)	111.57(17)	C(13) – C(12) – C(11)	122.03(16)
C(13) – C(12) – P	124.87(14)	C(11) – C(12) – P	113.04(13)
C(12) – C(13) – C(14)	121.72(17)	C(12) – C(13) – H(13)	119.1
C(14) – C(13) – H(13)	119.1	C(13) – C(14) – C(15)	116.43(17)
C(13) – C(14) – C(10)	121.87(17)	C(15) – C(14) – C(10)	121.53(17)
C(16) – C(15) – C(14)	122.70(18)	C(16) – C(15) – H(15)	118.7
C(14) – C(15) – H(15)	118.7	C(15) – C(16) – C(11)	121.33(18)
C(15) – C(16) – H(16)	119.3	C(11) – C(16) – H(16)	119.3
C(10) – C(17) – H(17A)	109.5	C(10) – C(17) – H(17B)	109.5
H(17A) – C(17) – H(17B)	109.5	C(10) – C(17) – H(17C)	109.5
H(17A) – C(17) – H(17C)	109.5	H(17B) – C(17) – H(17C)	109.5
C(10) – C(18) – H(18A)	109.5	C(10) – C(18) – H(18B)	109.5
H(18A) – C(18) – H(18B)	109.5	C(10) – C(18) – H(18C)	109.5
H(18A) – C(18) – H(18C)	109.5	H(18B) – C(18) – H(18C)	109.5
C(10) – C(19) – H(19A)	109.5	C(10) – C(19) – H(19B)	109.5
H(19A) – C(19) – H(19B)	109.5	C(10) – C(19) – H(19C)	109.5
H(19A) – C(19) – H(19C)	109.5	H(19B) – C(19) – H(19C)	109.5
C(23) – C(21) – C(24)	111.10(16)	C(23) – C(21) – P	109.52(13)
C(24) – C(21) – P	114.38(13)	C(23) – C(21) – H(21)	107.2
C(24) – C(21) – H(21)	107.2	P – C(21) – H(21)	107.2
C(25) – C(22) – C(26)	111.53(17)	C(25) – C(22) – P	111.30(13)
C(26) – C(22) – P	110.84(13)	C(25) – C(22) – H(22)	107.7
C(26) – C(22) – H(22)	107.7	P – C(22) – H(22)	107.7
C(21) – C(23) – H(23A)	109.5	C(21) – C(23) – H(23B)	109.5
H(23A) – C(23) – H(23B)	109.5	C(21) – C(23) – H(23C)	109.5
H(23A) – C(23) – H(23C)	109.5	H(23B) – C(23) – H(23C)	109.5
C(21) – C(24) – H(24A)	109.5	C(21) – C(24) – H(24B)	109.5

Symmetry transformations used to generate equivalent atoms: #1 $-x + 1, y, -z + \frac{1}{2}$

Continued on next page

Table S30. – continued from previous page

atom – atom – atom	angle	atom – atom – atom	angle
H(24A) – C(24) – H(24B)	109.5	C(21) – C(24) – H(24C)	109.5
H(24A) – C(24) – H(24C)	109.5	H(24B) – C(24) – H(24C)	109.5
C(22) – C(25) – H(25A)	109.5	C(22) – C(25) – H(25B)	109.5
H(25A) – C(25) – H(25B)	109.5	C(22) – C(25) – H(25C)	109.5
H(25A) – C(25) – H(25C)	109.5	H(25B) – C(25) – H(25C)	109.5
C(22) – C(26) – H(26A)	109.5	C(22) – C(26) – H(26B)	109.5
H(26A) – C(26) – H(26B)	109.5	C(22) – C(26) – H(26C)	109.5
H(26A) – C(26) – H(26C)	109.5	H(26B) – C(26) – H(26C)	109.5
N – C(31) – C(36)	120.22(18)	N – C(31) – C(32)	124.5(2)
C(36) – C(31) – C(32)	115.00(19)	C(33) – C(32) – C(31)	121.2(2)
C(33) – C(32) – H(32)	119.4	C(31) – C(32) – H(32)	119.4
C(34) – C(33) – C(32)	121.7(2)	C(34) – C(33) – H(33)	119.1
C(32) – C(33) – H(33)	119.1	C(35) – C(34) – C(33)	118.6(2)
C(35) – C(34) – H(34)	120.7	C(33) – C(34) – H(34)	120.7
C(34) – C(35) – C(36)	120.3(3)	C(34) – C(35) – H(35)	119.8
C(36) – C(35) – H(35)	119.8	C(35) – C(36) – C(31)	123.2(2)
C(35) – C(36) – H(36)	118.4	C(31) – C(36) – H(36)	118.4

Symmetry transformations used to generate equivalent atoms: #1 $-x+1, y, -z+\frac{1}{2}$

5.3 Crystal data for [{PC(sp^2)P}^{tBu}PdNPh$_2$]$^-$[KOEt$_2$]$^+$ (5)

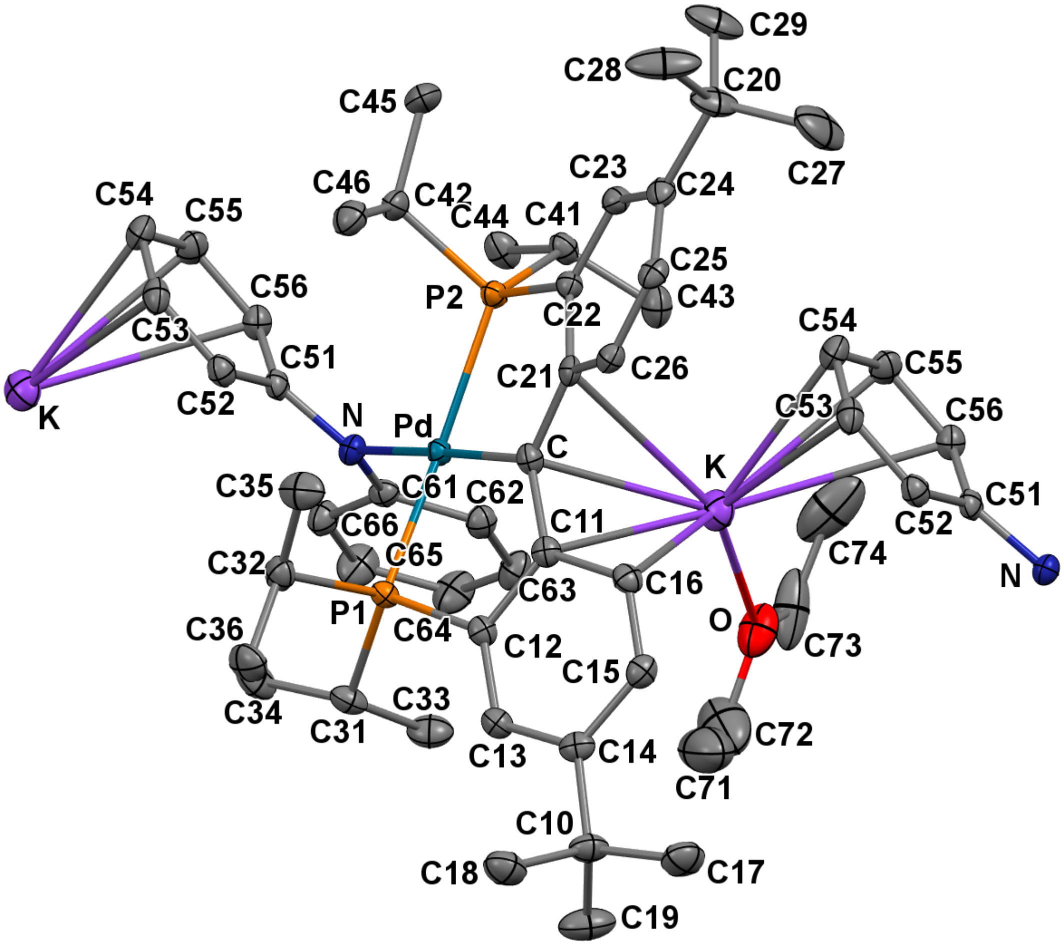

Figure S46. Thermal-ellipsoid representation of [{PC(sp^2)P}^{tBu}PdNPh$_2$]$^-$[KOEt$_2$]$^+$ (**5**) at 50% probability. Hydrogen atoms were omitted for clarity.

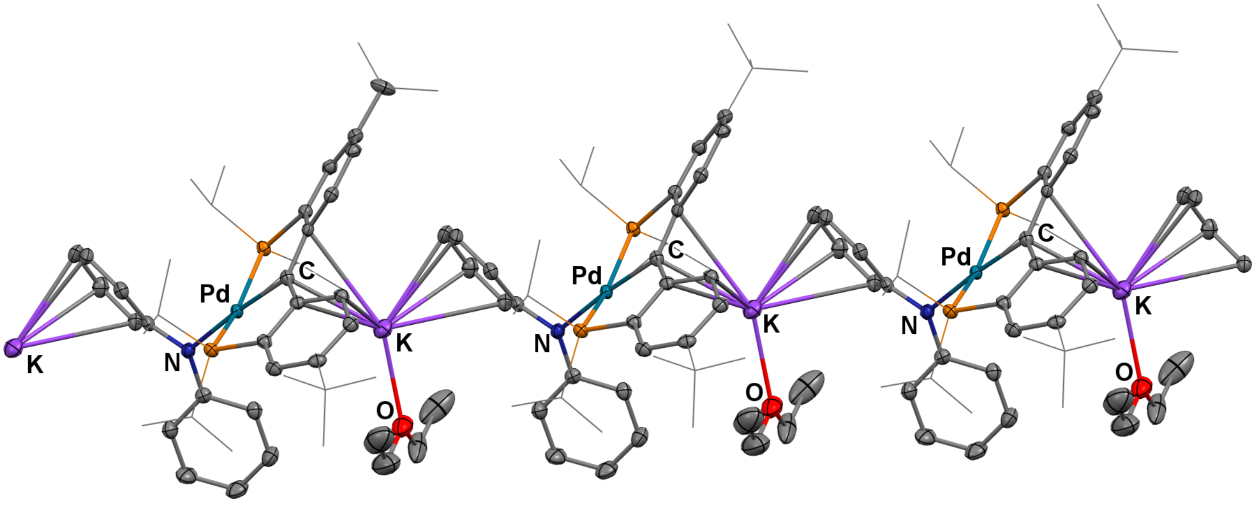

Figure S47. Polymeric structure of [{PC(sp^2)P}^{tBu}PdNPh$_2$]$^-$[KOEt$_2$]$^+$ (**5**).

Table S31. Crystal data and structure refinement for $[\{PC(sp^2)P\}^{tBu}PdNPh_2]^-[KOEt_2]^+$ (**5**).

Identification code:	pc47	
Empirical formula:	$C_{49}H_{72}KNOP_2Pd$	
Formula weight:	898.52	
Temperature:	120(2) K	
Wavelength:	0.71073 Å	
Crystal system:	Monoclinic	
Space group:	$P2_1/n$	
Unit cell dimensions:	a = 10.2409(9) Å	α = 90°
	b = 18.1690(15) Å	β = 90.553(3)°
	c = 25.743(2) Å	γ = 90°
Volume:	4789.7(7) $Å^3$	
Z:	4	
Density (calculated):	1.246 $g\cdot cm^{-3}$	
Absorption coefficient (μ):	0.575 mm^{-1}	
F(000):	1904	
Crystal size:	0.12 × 0.11 × 0.07 mm^3	
θ range for data collection:	1.94 to 25.00°	
Index ranges:	$-12 \le h \le 12$, $-15 \le k \le 21$, $-30 \le l \le 30$	
Reflections collected:	75438	
Independent reflections:	8436 [R_{int} = 0.0384]	
Completeness to θ = 25.00°:	99.9 %	
Absorption correction:	Semi-empirical from equivalents	
Max. and min. transmission:	0.7457 and 0.6996	
Refinement method:	Full-matrix least-squares on F^2	
Data / restraints / parameters:	8436 / 0 / 512	
Goodness-of-fit on F^2:	1.049	
Final R indices [I>2σ(I)]:	R_1 = 0.0252, wR_2 = 0.0563	
R indices (all data):	R_1 = 0.0318, wR_2 = 0.0582	
Largest diff. peak and hole:	0.420 and −0.279 $e^-\cdot Å^{-3}$	

Table S32. Atomic coordinates and equivalent isotropic displacement parameters (Å^2) for [{PC(sp^2)P}^{tBu}PdNPh$_2$]$^-$[KOEt$_2$]$^+$ (**5**). U(eq) is defined as one third of the trace of the orthogonalized U_{ij} tensor

atom	x	y	z	U(eq)
Pd	0.49711(1)	0.09320(1)	0.20563(1)	0.013(1)
C(22)	0.66834(18)	0.09791(11)	0.31091(7)	0.016(1)
P(1)	0.48476(5)	0.17596(3)	0.13905(2)	0.015(1)
C(23)	0.72261(19)	0.09162(11)	0.36063(7)	0.018(1)
P(2)	0.53023(5)	0.04634(3)	0.28798(2)	0.015(1)
C(24)	0.81603(19)	0.14046(12)	0.37919(8)	0.019(1)
C(26)	0.79972(18)	0.20395(11)	0.29617(8)	0.017(1)
C(21)	0.71084(18)	0.15156(11)	0.27465(8)	0.015(1)
C	0.66299(19)	0.15148(11)	0.22225(8)	0.017(1)
C(20)	0.8791(2)	0.13562(14)	0.43302(8)	0.030(1)
C(28)	0.8456(3)	0.20500(15)	0.46354(10)	0.054(1)
C(27)	1.0275(3)	0.12999(19)	0.42759(11)	0.053(1)
C(29)	0.8303(3)	0.06954(15)	0.46354(9)	0.041(1)
C(12)	0.64850(19)	0.21001(11)	0.13508(8)	0.017(1)
C(11)	0.72145(19)	0.19388(11)	0.18149(8)	0.016(1)
C(10)	0.8855(2)	0.31327(12)	0.04687(8)	0.023(1)
C(15)	0.9054(2)	0.25212(11)	0.13664(8)	0.019(1)
C(14)	0.8319(2)	0.26995(11)	0.09265(8)	0.019(1)
C(13)	0.70181(19)	0.24704(11)	0.09343(8)	0.018(1)
C(17)	1.0306(2)	0.32939(14)	0.05312(9)	0.032(1)
C(18)	0.8139(2)	0.38720(13)	0.04304(9)	0.031(1)
C(19)	0.8650(3)	0.27129(15)	−0.00369(9)	0.039(1)
N	0.33184(15)	0.02650(9)	0.18497(6)	0.016(1)
C(31)	0.4302(2)	0.15321(12)	0.07285(8)	0.022(1)
C(33)	0.5107(2)	0.09055(13)	0.05058(8)	0.031(1)
C(34)	0.2846(2)	0.13484(14)	0.07147(9)	0.032(1)
C(42)	0.39437(19)	0.06894(12)	0.33238(8)	0.020(1)
C(46)	0.3827(2)	0.15188(12)	0.33794(9)	0.026(1)
C(51)	0.22261(18)	0.03394(11)	0.21513(8)	0.017(1)
C(52)	0.18579(19)	0.10431(11)	0.23196(8)	0.019(1)
C(53)	0.09146(19)	0.11463(12)	0.26896(8)	0.022(1)
C(54)	0.0262(2)	0.05511(12)	0.29029(8)	0.024(1)
C(55)	0.0575(2)	−0.01465(12)	0.27271(8)	0.023(1)
C(56)	0.15123(19)	−0.02551(12)	0.23540(8)	0.019(1)
C(61)	0.34516(19)	−0.03273(11)	0.15206(8)	0.017(1)
C(62)	0.4703(2)	−0.05907(11)	0.14013(8)	0.020(1)
C(63)	0.4879(2)	−0.11539(12)	0.10518(9)	0.026(1)
C(64)	0.3834(2)	−0.14855(13)	0.08055(9)	0.033(1)
C(65)	0.2596(2)	−0.12290(13)	0.09134(9)	0.032(1)

Continued on next page

Table S32. – continued from previous page

atom	x	y	x	U(eq)
C(66)	0.2407(2)	−0.06625(12)	0.12570(8)	0.023(1)
C(71)	0.8918(4)	0.0677(2)	0.06195(12)	0.068(1)
C(72)	0.8650(4)	−0.0136(2)	0.05975(13)	0.073(1)
C(73)	0.8817(3)	−0.12236(17)	0.10856(15)	0.062(1)
C(74)	0.9095(4)	−0.15203(17)	0.16145(15)	0.071(1)
C(45)	0.4011(2)	0.03314(14)	0.38637(8)	0.030(1)
C(41)	0.56477(19)	−0.05181(11)	0.29988(8)	0.019(1)
C(43)	0.6881(2)	−0.07526(12)	0.27211(9)	0.027(1)
C(44)	0.4482(2)	−0.09905(12)	0.28349(9)	0.025(1)
C(32)	0.3831(2)	0.25641(11)	0.15708(8)	0.022(1)
C(35)	0.4233(3)	0.28431(13)	0.21019(9)	0.032(1)
C(36)	0.3853(2)	0.31911(12)	0.11770(9)	0.031(1)
C(16)	0.85414(19)	0.21697(11)	0.17915(8)	0.018(1)
O	0.88447(16)	−0.04438(10)	0.10966(7)	0.041(1)
C(25)	0.84866(19)	0.19826(11)	0.34570(8)	0.018(1)
K	0.87015(5)	0.04914(3)	0.19096(2)	0.029(1)
H(23)	0.6947	0.0526	0.3825	0.021
H(26)	0.8260	0.2444	0.2753	0.020
H(28A)	0.8777	0.2483	0.4449	0.080
H(28B)	0.8870	0.2027	0.4980	0.080
H(28C)	0.7507	0.2086	0.4673	0.080
H(27A)	1.0495	0.0844	0.4093	0.080
H(27B)	1.0684	0.1296	0.4622	0.080
H(27C)	1.0597	0.1723	0.4079	0.080
H(29A)	0.7357	0.0735	0.4681	0.062
H(29B)	0.8735	0.0683	0.4977	0.062
H(29C)	0.8503	0.0243	0.4445	0.062
H(15)	0.9953	0.2649	0.1373	0.023
H(13)	0.6477	0.2573	0.0641	0.022
H(17A)	1.0791	0.2829	0.0549	0.049
H(17B)	1.0455	0.3575	0.0851	0.049
H(17C)	1.0607	0.3581	0.0233	0.049
H(18A)	0.8468	0.4150	0.0133	0.047
H(18B)	0.8291	0.4155	0.0750	0.047
H(18C)	0.7200	0.3784	0.0385	0.047
H(19A)	0.8962	0.3011	−0.0328	0.058
H(19B)	0.7719	0.2608	−0.0086	0.058
H(19C)	0.9139	0.2249	−0.0023	0.058
H(31)	0.4441	0.1976	0.0506	0.026
H(33A)	0.4818	0.0805	0.0149	0.046
H(33B)	0.4988	0.0463	0.0718	0.046
H(33C)	0.6031	0.1044	0.0507	0.046

Continued on next page

Table S32. – continued from previous page

atom	x	y	x	U(eq)
H(34A)	0.2581	0.1224	0.0359	0.047
H(34B)	0.2345	0.1775	0.0833	0.047
H(34C)	0.2678	0.0928	0.0943	0.047
H(42)	0.3120	0.0513	0.3153	0.024
H(46A)	0.3781	0.1745	0.3034	0.040
H(46B)	0.4591	0.1709	0.3568	0.040
H(46C)	0.3033	0.1638	0.3572	0.040
H(52)	0.2273	0.1461	0.2174	0.023
H(53)	0.0708	0.1631	0.2800	0.026
H(54)	−0.0380	0.0620	0.3162	0.029
H(55)	0.0133	−0.0560	0.2867	0.028
H(56)	0.1679	−0.0739	0.2232	0.023
H(62)	0.5444	−0.0375	0.1566	0.024
H(63)	0.5739	−0.1317	0.0979	0.032
H(64)	0.3961	−0.1879	0.0568	0.039
H(65)	0.1862	−0.1449	0.0747	0.038
H(66)	0.1544	−0.0493	0.1318	0.027
H(71A)	0.8811	0.0889	0.0272	0.102
H(71B)	0.9813	0.0760	0.0744	0.102
H(71C)	0.8303	0.0912	0.0857	0.102
H(72A)	0.7739	−0.0223	0.0480	0.087
H(72B)	0.9243	−0.0373	0.0346	0.087
H(73A)	0.9479	−0.1408	0.0840	0.074
H(73B)	0.7948	−0.1394	0.0965	0.074
H(74A)	0.9061	−0.2059	0.1605	0.106
H(74B)	0.8441	−0.1335	0.1857	0.106
H(74C)	0.9966	−0.1361	0.1729	0.106
H(45A)	0.4033	−0.0205	0.3826	0.045
H(45B)	0.3241	0.0474	0.4063	0.045
H(45C)	0.4802	0.0497	0.4046	0.045
H(41)	0.5794	−0.0587	0.3380	0.023
H(43A)	0.6752	−0.0701	0.2345	0.040
H(43B)	0.7077	−0.1267	0.2805	0.040
H(43C)	0.7611	−0.0441	0.2834	0.040
H(44A)	0.4287	−0.0908	0.2466	0.037
H(44B)	0.3720	−0.0855	0.3042	0.037
H(44C)	0.4689	−0.1511	0.2892	0.037
H(32)	0.2908	0.2389	0.1595	0.027
H(35A)	0.5136	0.3020	0.2091	0.048
H(35B)	0.4167	0.2443	0.2355	0.048
H(35C)	0.3656	0.3248	0.2203	0.048
H(36A)	0.3502	0.3017	0.0844	0.046

Continued on next page

Table S32. – continued from previous page

**atom**	**x**	**y**	**x**	**U(eq)**
H(36B)	0.4754	0.3359	0.1131	0.046
H(36C)	0.3319	0.3600	0.1303	0.046
H(16)	0.9097	0.2077	0.2082	0.022
H(25)	0.9072	0.2351	0.3579	0.021

Table S33. Anisotropic displacement parameters (Å^2) for [{PC(sp^2)P}^{tBu}PdNPh$_2$]$^-$[KOEt$_2$]$^+$ (**5**). The anisotropic displacement factor exponent takes the form: $-2\pi^2[h^2a^{*2}U_{11} + ... + 2hka^*b^*U_{12}]$.

atom	U_{11}	U_{22}	U_{33}	U_{23}	U_{13}	U_{12}
Pd	0.0121(1)	0.0128(1)	0.0138(1)	0.0005(1)	−0.0009(1)	−0.0008(1)
C(22)	0.0144(9)	0.0161(10)	0.0161(10)	−0.0011(8)	0.0004(8)	0.0005(8)
P(1)	0.0155(3)	0.0147(3)	0.0147(3)	0.0010(2)	−0.0020(2)	−0.0007(2)
C(23)	0.0182(10)	0.0190(10)	0.0156(10)	0.0003(9)	0.0024(8)	0.0011(9)
P(2)	0.0144(2)	0.0160(3)	0.0154(3)	0.0021(2)	−0.0008(2)	−0.0010(2)
C(24)	0.0171(10)	0.0239(11)	0.0162(10)	−0.0038(9)	0.0003(8)	0.0021(9)
C(26)	0.0152(10)	0.0151(10)	0.0196(10)	−0.0007(8)	0.0032(8)	0.0004(8)
C(21)	0.0115(9)	0.0155(10)	0.0183(10)	−0.0012(8)	0.0011(8)	0.0034(8)
C	0.0145(10)	0.0166(10)	0.0198(10)	0.0004(8)	−0.0021(8)	0.0004(8)
C(20)	0.0390(14)	0.0356(14)	0.0155(11)	0.0007(10)	−0.0080(10)	−0.0128(11)
C(28)	0.100(3)	0.0421(17)	0.0187(13)	−0.0078(12)	0.0069(14)	−0.0323(17)
C(27)	0.0405(16)	0.078(2)	0.0412(16)	0.0167(16)	−0.0246(13)	−0.0161(15)
C(29)	0.0603(18)	0.0410(15)	0.0224(12)	0.0075(11)	−0.0183(12)	−0.0176(13)
C(12)	0.0167(10)	0.0152(10)	0.0177(10)	−0.0014(8)	−0.0021(8)	−0.0011(8)
C(11)	0.0172(10)	0.0132(10)	0.0176(10)	−0.0023(8)	−0.0006(8)	−0.0004(8)
C(10)	0.0270(12)	0.0240(12)	0.0187(11)	0.0026(9)	0.0025(9)	−0.0069(9)
C(15)	0.0176(10)	0.0184(11)	0.0215(11)	−0.0031(9)	0.0010(8)	−0.0034(8)
C(14)	0.0242(11)	0.0144(10)	0.0173(10)	−0.0023(8)	0.0019(8)	−0.0025(9)
C(13)	0.0202(10)	0.0165(10)	0.0173(10)	0.0005(8)	−0.0026(8)	−0.0003(8)
C(17)	0.0325(13)	0.0351(14)	0.0301(13)	0.0084(11)	0.0056(10)	−0.0077(11)
C(18)	0.0360(13)	0.0299(13)	0.0272(12)	0.0075(10)	−0.0005(10)	−0.0034(11)
C(19)	0.0556(17)	0.0399(15)	0.0205(12)	−0.0030(11)	0.0066(11)	−0.0163(13)
N	0.0138(8)	0.0169(9)	0.0189(9)	−0.0014(7)	0.0010(7)	−0.0004(7)
C(31)	0.0261(11)	0.0217(11)	0.0168(10)	0.0033(9)	−0.0055(9)	−0.0048(9)
C(33)	0.0418(14)	0.0296(13)	0.0200(11)	−0.0046(10)	0.0006(10)	−0.0037(11)
C(34)	0.0290(13)	0.0350(14)	0.0306(13)	0.0034(11)	−0.0129(10)	−0.0071(11)
C(42)	0.0146(10)	0.0257(11)	0.0195(11)	0.0025(9)	0.0015(8)	−0.0007(9)
C(46)	0.0257(12)	0.0286(13)	0.0250(12)	−0.0034(10)	0.0050(9)	0.0027(10)
C(51)	0.0118(9)	0.0199(11)	0.0180(10)	−0.0008(8)	−0.0032(8)	0.0005(8)
C(52)	0.0159(10)	0.0196(11)	0.0214(11)	0.0013(9)	−0.0026(8)	−0.0008(8)
C(53)	0.0165(10)	0.0229(11)	0.0249(11)	−0.0057(9)	−0.0019(9)	0.0047(9)
C(54)	0.0173(11)	0.0292(12)	0.0250(12)	−0.0013(10)	0.0047(9)	0.0041(9)
C(55)	0.0187(11)	0.0236(12)	0.0276(12)	0.0051(10)	0.0037(9)	−0.0021(9)
C(56)	0.0165(10)	0.0175(11)	0.0241(11)	−0.0005(9)	−0.0005(8)	0.0006(8)
C(61)	0.0185(10)	0.0151(10)	0.0167(10)	0.0015(8)	0.0011(8)	−0.0007(8)
C(62)	0.0172(10)	0.0187(11)	0.0232(11)	0.0009(9)	0.0000(8)	−0.0023(8)
C(63)	0.0233(11)	0.0231(12)	0.0329(13)	−0.0035(10)	0.0059(10)	0.0033(9)
C(64)	0.0362(14)	0.0274(13)	0.0343(14)	−0.0140(11)	0.0051(11)	−0.0007(11)
C(65)	0.0288(13)	0.0342(13)	0.0318(13)	−0.0138(11)	−0.0018(10)	−0.0088(11)
C(66)	0.0172(10)	0.0273(12)	0.0241(11)	−0.0029(10)	0.0005(9)	−0.0021(9)

Continued on next page

Table S33. – continued from previous page

atom	U_{11}	U_{22}	U_{33}	U_{23}	U_{13}	U_{12}
C(71)	0.083(3)	0.078(3)	0.0435(18)	0.0083(17)	0.0097(17)	0.029(2)
C(72)	0.083(3)	0.083(3)	0.051(2)	−0.0286(19)	−0.0119(18)	0.036(2)
C(73)	0.0251(14)	0.0452(18)	0.115(3)	−0.045(2)	−0.0109(16)	0.0021(13)
C(74)	0.078(2)	0.0309(17)	0.104(3)	−0.0127(18)	0.050(2)	−0.0190(16)
C(45)	0.0251(12)	0.0430(15)	0.0223(12)	0.0073(11)	0.0073(9)	0.0025(11)
C(41)	0.0186(10)	0.0176(11)	0.0216(11)	0.0048(9)	−0.0028(8)	−0.0010(9)
C(43)	0.0207(11)	0.0226(12)	0.0374(13)	0.0002(10)	−0.0009(10)	0.0030(9)
C(44)	0.0242(11)	0.0191(11)	0.0311(12)	0.0056(10)	−0.0057(9)	−0.0044(9)
C(32)	0.0217(11)	0.0187(11)	0.0260(11)	0.0025(9)	0.0013(9)	0.0040(9)
C(35)	0.0506(15)	0.0187(12)	0.0278(13)	−0.0027(10)	0.0049(11)	0.0076(11)
C(36)	0.0391(14)	0.0229(12)	0.0303(13)	0.0064(10)	0.0001(11)	0.0105(10)
C(16)	0.0186(10)	0.0192(11)	0.0163(10)	−0.0018(9)	−0.0029(8)	−0.0009(8)
O	0.0311(9)	0.0398(11)	0.0517(12)	−0.0208(9)	0.0016(8)	−0.0006(8)
C(25)	0.0164(10)	0.0184(11)	0.0183(10)	−0.0055(9)	0.0018(8)	−0.0019(8)
K	0.0322(3)	0.0233(3)	0.0317(3)	−0.0040(2)	−0.0002(2)	0.0052(2)

Table S34. Distances [Å] for $[\{PC(sp^2)P\}^{tBu}PdNPh_2]^-[KOEt_2]^+$ (**5**).

**atom – atom**	**distance**	**atom – atom**	**distance**
Pd – C	2.043(2)	Pd – N	2.1445(16)
Pd – P(1)	2.2827(5)	Pd – P(2)	2.3064(5)
C(22) – C(23)	1.395(3)	C(22) – C(21)	1.421(3)
C(22) – P(2)	1.792(2)	P(1) – C(12)	1.791(2)
P(1) – C(31)	1.835(2)	P(1) – C(32)	1.856(2)
C(23) – C(24)	1.386(3)	C(23) – H(23)	0.9500
P(2) – C(41)	1.843(2)	P(2) – C(42)	1.855(2)
C(24) – C(25)	1.401(3)	C(24) – C(20)	1.526(3)
C(26) – C(25)	1.369(3)	C(26) – C(21)	1.425(3)
C(26) – H(26)	0.9500	C(21) – C	1.431(3)
C(21) – K	3.292(2)	C – C(11)	1.437(3)
C – K	2.939(2)	C(20) – C(29)	1.522(3)
C(20) – C(28)	1.526(4)	C(20) – C(27)	1.532(4)
C(28) – H(28A)	0.9800	C(28) – H(28B)	0.9800
C(28) – H(28C)	0.9800	C(27) – H(27A)	0.9800
C(27) – H(27B)	0.9800	C(27) – H(27C)	0.9800
C(29) – H(29A)	0.9800	C(29) – H(29B)	0.9800
C(29) – H(29C)	0.9800	C(12) – C(13)	1.382(3)
C(12) – C(11)	1.433(3)	C(11) – C(16)	1.424(3)
C(11) – K	3.047(2)	C(10) – C(19)	1.521(3)
C(10) – C(17)	1.522(3)	C(10) – C(14)	1.524(3)
C(10) – C(18)	1.533(3)	C(15) – C(16)	1.375(3)
C(15) – C(14)	1.392(3)	C(15) – H(15)	0.9500
C(14) – C(13)	1.396(3)	C(13) – H(13)	0.9500
C(17) – H(17A)	0.9800	C(17) – H(17B)	0.9800
C(17) – H(17C)	0.9800	C(18) – H(18A)	0.9800
C(18) – H(18B)	0.9800	C(18) – H(18C)	0.9800
C(19) – H(19A)	0.9800	C(19) – H(19B)	0.9800
C(19) – H(19C)	0.9800	N – C(51)	1.375(3)
N – C(61)	1.377(3)	C(31) – C(33)	1.521(3)
C(31) – C(34)	1.528(3)	C(31) – H(31)	1.0000
C(33) – H(33A)	0.9800	C(33) – H(33B)	0.9800
C(33) – H(33C)	0.9800	C(34) – H(34A)	0.9800
C(34) – H(34B)	0.9800	C(34) – H(34C)	0.9800
C(42) – C(46)	1.519(3)	C(42) – C(45)	1.536(3)
C(42) – H(42)	1.0000	C(46) – H(46A)	0.9800
C(46) – H(46B)	0.9800	C(46) – H(46C)	0.9800
C(51) – C(52)	1.403(3)	C(51) – C(56)	1.407(3)
C(52) – C(53)	1.376(3)	C(52) – H(52)	0.9500
C(53) – C(54)	1.387(3)	C(53) – H(53)	0.9500

Symmetry transformations used to generate equivalent atoms: #1 $x + 1, y, z$

Continued on next page

Table S34. – continued from previous page

atom – atom	distance	atom – atom	distance
C(54) – C(55)	1.384(3)	C(54) – H(54)	0.9500
C(55) – C(56)	1.379(3)	C(55) – H(55)	0.9500
C(56) – H(56)	0.9500	C(61) – C(66)	1.401(3)
C(61) – C(62)	1.405(3)	C(62) – C(63)	1.376(3)
C(62) – H(62)	0.9500	C(63) – C(64)	1.377(3)
C(63) – H(63)	0.9500	C(64) – C(65)	1.382(3)
C(64) – H(64)	0.9500	C(65) – C(66)	1.372(3)
C(65) – H(65)	0.9500	C(66) – H(66)	0.9500
C(71) – C(72)	1.504(5)	C(71) – H(71A)	0.9800
C(71) – H(71B)	0.9800	C(71) – H(71C)	0.9800
C(72) – O	1.414(4)	C(72) – H(72A)	0.9900
C(72) – H(72B)	0.9900	C(73) – O	1.417(3)
C(73) – C(74)	1.489(5)	C(73) – H(73A)	0.9900
C(73) – H(73B)	0.9900	C(74) – H(74A)	0.9800
C(74) – H(74B)	0.9800	C(74) – H(74C)	0.9800
C(45) – H(45A)	0.9800	C(45) – H(45B)	0.9800
C(45) – H(45C)	0.9800	C(41) – C(43)	1.519(3)
C(41) – C(44)	1.527(3)	C(41) – H(41)	1.0000
C(43) – H(43A)	0.9800	C(43) – H(43B)	0.9800
C(43) – H(43C)	0.9800	C(44) – H(44A)	0.9800
C(44) – H(44B)	0.9800	C(44) – H(44C)	0.9800
C(32) – C(35)	1.512(3)	C(32) – C(36)	1.525(3)
C(32) – H(32)	1.0000	C(35) – H(35A)	0.9800
C(35) – H(35B)	0.9800	C(35) – H(35C)	0.9800
C(36) – H(36A)	0.9800	C(36) – H(36B)	0.9800
C(36) – H(36C)	0.9800	C(16) – K	3.069(2)
C(16) – H(16)	0.9500	O – K	2.7010(17)
C(25) – H(25)	0.9500	K – C(54)#1	3.004(2)
K – C(55)#1	3.062(2)	K – C(53)#1	3.240(2)
K – C(56)#1	3.372(2)	K – C(52)#1	3.536(2)
K – C(51)#1	3.667(2)		

Symmetry transformations used to generate equivalent atoms: #1 $x + 1, y, z$

Table S35. Angles [°] for $[\{PC(sp^2)P\}^{tBu}PdNPh_2]^-[KOEt_2]^+$ (**5**).

atom – atom – atom	angle	atom – atom – atom	angle
C – Pd – N	175.74(7)	C – Pd – P(1)	81.71(6)
N – Pd – P(1)	98.52(5)	C – Pd – P(2)	83.36(6)
N – Pd – P(2)	97.36(5)	P(1) – Pd – P(2)	160.036(19)
C(23) – C(22) – C(21)	122.49(18)	C(23) – C(22) – P(2)	124.44(15)
C(21) – C(22) – P(2)	112.84(14)	C(12) – P(1) – C(31)	107.54(9)
C(12) – P(1) – C(32)	105.66(10)	C(31) – P(1) – C(32)	104.07(10)
C(12) – P(1) – Pd	103.02(7)	C(31) – P(1) – Pd	124.27(7)
C(32) – P(1) – Pd	110.99(7)	C(24) – C(23) – C(22)	122.08(19)
C(24) – C(23) – H(23)	119.0	C(22) – C(23) – H(23)	119.0
C(22) – P(2) – C(41)	107.55(9)	C(22) – P(2) – C(42)	105.99(9)
C(41) – P(2) – C(42)	104.81(9)	C(22) – P(2) – Pd	102.61(7)
C(41) – P(2) – Pd	122.44(7)	C(42) – P(2) – Pd	112.34(7)
C(23) – C(24) – C(25)	115.82(18)	C(23) – C(24) – C(20)	124.11(19)
C(25) – C(24) – C(20)	120.04(18)	C(25) – C(26) – C(21)	122.55(19)
C(25) – C(26) – H(26)	118.7	C(21) – C(26) – H(26)	118.7
C(22) – C(21) – C(26)	113.66(17)	C(22) – C(21) – C	120.96(18)
C(26) – C(21) – C	125.36(18)	C(22) – C(21) – K	101.48(12)
C(26) – C(21) – K	108.25(12)	C – C(21) – K	63.23(10)
C(21) – C – C(11)	123.14(18)	C(21) – C – Pd	118.31(14)
C(11) – C – Pd	118.45(14)	C(21) – C – K	91.00(11)
C(11) – C – K	80.34(11)	Pd – C – K	102.45(8)
C(29) – C(20) – C(24)	112.10(18)	C(29) – C(20) – C(28)	108.0(2)
C(24) – C(20) – C(28)	109.0(2)	C(29) – C(20) – C(27)	109.0(2)
C(24) – C(20) – C(27)	109.44(19)	C(28) – C(20) – C(27)	109.3(2)
C(13) – C(12) – C(11)	122.64(18)	C(13) – C(12) – P(1)	126.11(15)
C(11) – C(12) – P(1)	111.25(14)	C(16) – C(11) – C(12)	113.23(18)
C(16) – C(11) – C	126.38(18)	C(12) – C(11) – C	120.06(17)
C(16) – C(11) – K	77.37(11)	C(12) – C(11) – K	119.95(13)
C – C(11) – K	71.96(11)	C(19) – C(10) – C(17)	108.25(19)
C(19) – C(10) – C(14)	110.81(18)	C(17) – C(10) – C(14)	112.09(18)
C(19) – C(10) – C(18)	108.81(19)	C(17) – C(10) – C(18)	107.72(18)
C(14) – C(10) – C(18)	109.06(18)	C(16) – C(15) – C(14)	123.25(19)
C(16) – C(15) – H(15)	118.4	C(14) – C(15) – H(15)	118.4
C(15) – C(14) – C(13)	115.29(18)	C(15) – C(14) – C(10)	123.63(18)
C(13) – C(14) – C(10)	121.06(18)	C(12) – C(13) – C(14)	122.70(19)
C(12) – C(13) – H(13)	118.6	C(14) – C(13) – H(13)	118.6
C(51) – N – C(61)	120.68(17)	C(51) – N – Pd	116.68(13)
C(61) – N – Pd	120.77(13)	C(33) – C(31) – C(34)	111.08(19)
C(33) – C(31) – P(1)	110.96(15)	C(34) – C(31) – P(1)	111.03(15)
C(33) – C(31) – H(31)	107.9	C(34) – C(31) – H(31)	107.9

Symmetry transformations used to generate equivalent atoms: #1 $x + 1, y, z$

Continued on next page

Table S35. – continued from previous page

atom – atom – atom	angle	atom – atom – atom	angle
P(1) – C(31) – H(31)	107.9	C(46) – C(42) – C(45)	109.75(18)
C(46) – C(42) – P(2)	109.76(14)	C(45) – C(42) – P(2)	115.89(15)
C(46) – C(42) – H(42)	107.0	C(45) – C(42) – H(42)	107.0
P(2) – C(42) – H(42)	107.0	N – C(51) – C(52)	119.13(18)
N – C(51) – C(56)	124.23(18)	C(52) – C(51) – C(56)	116.28(18)
C(53) – C(52) – C(51)	122.1(2)	C(53) – C(52) – H(52)	119.0
C(51) – C(52) – H(52)	119.0	C(52) – C(53) – C(54)	120.8(2)
C(52) – C(53) – H(53)	119.6	C(54) – C(53) – H(53)	119.6
C(55) – C(54) – C(53)	118.09(19)	C(55) – C(54) – H(54)	121.0
C(53) – C(54) – H(54)	121.0	C(56) – C(55) – C(54)	121.6(2)
C(56) – C(55) – H(55)	119.2	C(54) – C(55) – H(55)	119.2
C(55) – C(56) – C(51)	121.1(2)	C(55) – C(56) – H(56)	119.5
C(51) – C(56) – H(56)	119.5	N – C(61) – C(66)	123.91(18)
N – C(61) – C(62)	119.78(18)	C(66) – C(61) – C(62)	116.12(19)
C(63) – C(62) – C(61)	121.45(19)	C(63) – C(62) – H(62)	119.3
C(61) – C(62) – H(62)	119.3	C(62) – C(63) – C(64)	121.4(2)
C(62) – C(63) – H(63)	119.3	C(64) – C(63) – H(63)	119.3
C(63) – C(64) – C(65)	118.0(2)	C(63) – C(64) – H(64)	121.0
C(65) – C(64) – H(64)	121.0	C(66) – C(65) – C(64)	121.2(2)
C(66) – C(65) – H(65)	119.4	C(64) – C(65) – H(65)	119.4
C(65) – C(66) – C(61)	121.8(2)	C(65) – C(66) – H(66)	119.1
C(61) – C(66) – H(66)	119.1	O – C(72) – C(71)	109.3(3)
O – C(72) – H(72A)	109.8	C(71) – C(72) – H(72A)	109.8
O – C(72) – H(72B)	109.8	C(71) – C(72) – H(72B)	109.8
H(72A) – C(72) – H(72B)	108.3	O – C(73) – C(74)	109.9(2)
O – C(73) – H(73A)	109.7	C(74) – C(73) – H(73A)	109.7
O – C(73) – H(73B)	109.7	C(74) – C(73) – H(73B)	109.7
H(73A) – C(73) – H(73B)	108.2	C(43) – C(41) – C(44)	111.36(18)
C(43) – C(41) – P(2)	110.63(14)	C(44) – C(41) – P(2)	110.46(14)
C(43) – C(41) – H(41)	108.1	C(44) – C(41) – H(41)	108.1
P(2) – C(41) – H(41)	108.1	C(35) – C(32) – C(36)	110.17(18)
C(35) – C(32) – P(1)	109.98(15)	C(36) – C(32) – P(1)	114.20(15)
C(35) – C(32) – H(32)	107.4	C(36) – C(32) – H(32)	107.4
P(1) – C(32) – H(32)	107.4	C(15) – C(16) – C(11)	122.81(19)
C(15) – C(16) – K	121.31(14)	C(11) – C(16) – K	75.71(11)
C(15) – C(16) – H(16)	118.6	C(11) – C(16) – H(16)	118.6
K – C(16) – H(16)	73.4	C(72) – O – C(73)	112.0(2)
C(72) – O – K	116.56(17)	C(73) – O – K	130.0(2)
C(26) – C(25) – C(24)	122.83(19)	C(26) – C(25) – H(25)	118.6
C(24) – C(25) – H(25)	118.6	O – K – C	130.92(6)

Symmetry transformations used to generate equivalent atoms: #1 $x + 1, y, z$

Continued on next page

Table S35. – continued from previous page

**atom – atom – atom**	**angle**	**atom – atom – atom**	**angle**
O – K – C(54)#1	130.53(6)	C – K – C(54)#1	97.09(6)
O – K – C(11)	120.78(6)	C – K – C(11)	27.70(5)
C(54)#1 – K – C(11)	107.36(6)	O – K – C(55)#1	104.84(6)
C – K – C(55)#1	120.02(6)	C(54)#1 – K – C(55)#1	26.36(6)
C(11) – K – C(55)#1	133.71(6)	O – K – C(16)	123.48(6)
C – K – C(16)	50.25(5)	C(54)#1 – K – C(16)	94.35(6)
C(11) – K – C(16)	26.93(5)	C(55)#1 – K – C(16)	118.48(6)
O – K – C(53)#1	131.95(5)	C – K – C(53)#1	95.75(5)
C(54)#1 – K – C(53)#1	25.31(6)	C(11) – K – C(53)#1	94.51(5)
C(55)#1 – K – C(53)#1	44.20(6)	C(16) – K – C(53)#1	74.50(5)
O – K – C(21)	153.36(5)	C – K – C(21)	25.76(5)
C(54)#1 – K – C(21)	71.67(5)	C(11) – K – C(21)	46.69(5)
C(55)#1 – K – C(21)	94.32(5)	C(16) – K – C(21)	58.45(5)
C(53)#1 – K – C(21)	74.63(5)	O – K – C(56)#1	87.55(5)
C – K – C(56)#1	141.03(6)	C(54)#1 – K – C(56)#1	43.97(5)
C(11) – K – C(56)#1	142.99(5)	C(55)#1 – K – C(56)#1	24.13(5)
C(16) – K – C(56)#1	118.55(5)	C(53)#1 – K – C(56)#1	49.28(5)
C(21) – K – C(56)#1	115.61(5)	O – K – C(52)#1	110.69(5)
C – K – C(52)#1	113.52(5)	C(54)#1 – K – C(52)#1	42.12(5)
C(11) – K – C(52)#1	103.53(5)	C(55)#1 – K – C(52)#1	48.49(5)
C(16) – K – C(52)#1	78.21(5)	C(53)#1 – K – C(52)#1	22.90(5)
C(21) – K – C(52)#1	95.84(5)	C(56)#1 – K – C(52)#1	40.34(5)
O – K – C(51)#1	91.34(5)	C – K – C(51)#1	135.48(5)
C(54)#1 – K – C(51)#1	48.97(5)	C(11) – K – C(51)#1	124.67(5)
C(55)#1 – K – C(51)#1	41.04(5)	C(16) – K – C(51)#1	98.23(5)
C(53)#1 – K – C(51)#1	40.62(5)	C(21) – K – C(51)#1	115.14(5)
C(56)#1 – K – C(51)#1	22.56(5)	C(52)#1 – K – C(51)#1	22.36(5)

Symmetry transformations used to generate equivalent atoms: #1 $x + 1, y, z$

5.4 Crystal data for [{PC(sp^2)P}^{tBu}PdCH$_2$Ph]$^-$K$^+$ (6)

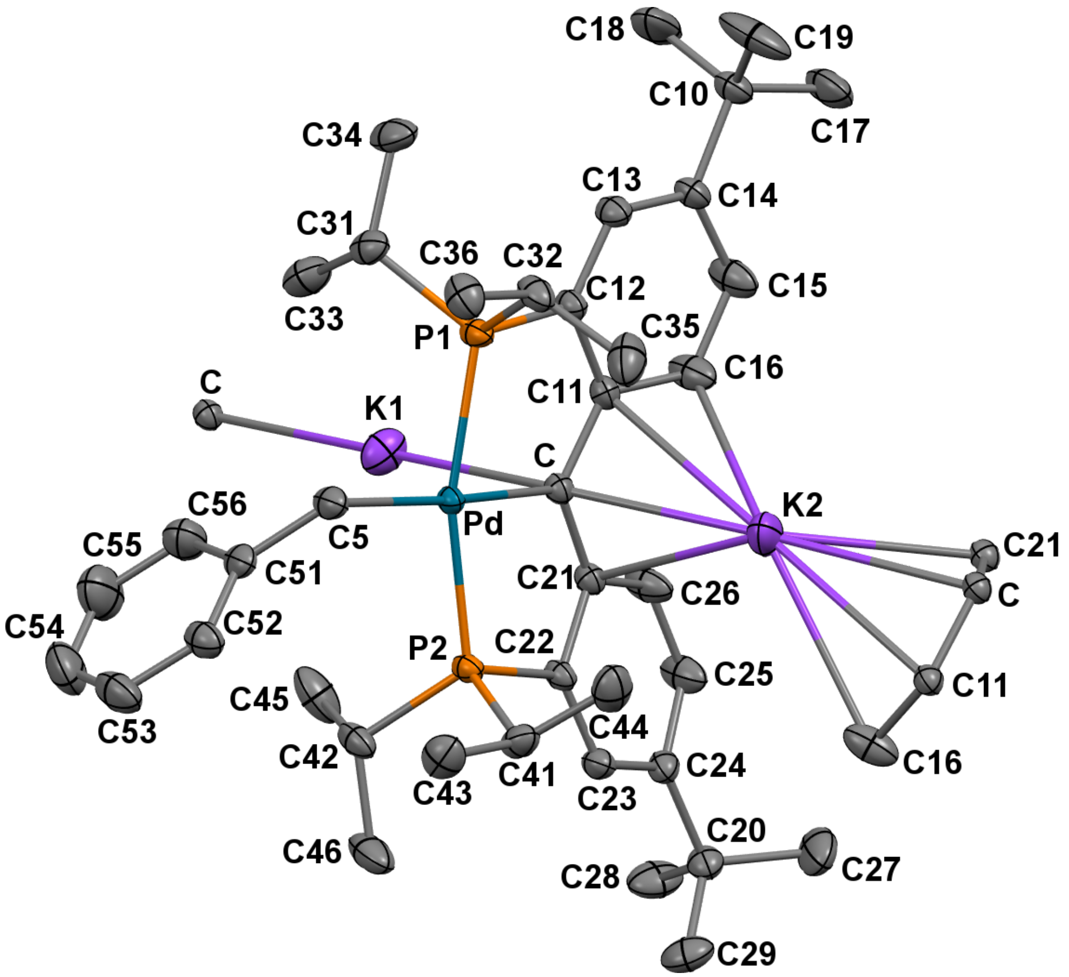

Figure S48. Thermal-ellipsoid representation of [{PC(sp^2)P}^{tBu}PdCH$_2$Ph]$^-$K$^+$ (**6**) at 50% probability. Hydrogen atoms were omitted for clarity.

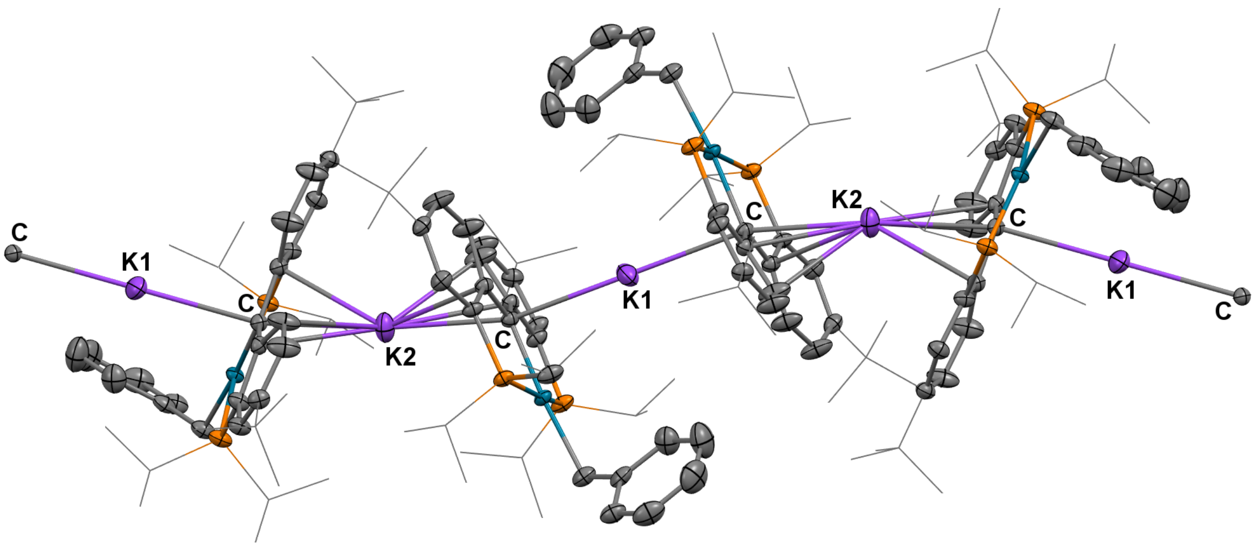

Figure S49. Polymeric structure of [{PC(sp^2)P}^{tBu}PdCH$_2$Ph]$^-$K$^+$ (**6**).

Table S36. Crystal data and structure refinement for $[\{PC(sp^2)P\}^{tBu}PdCH_2Ph]^-K^+$ (**6**).

Identification code:	pc44	
Empirical formula:	$C_{40}H_{59}KP_2Pd$	
Formula weight:	747.31	
Temperature:	120(2) K	
Wavelength:	0.71073 Å	
Crystal system:	Monoclinic	
Space group:	$C2/c$	
Unit cell dimensions:	a = 26.989(3) Å	α = 90°
	b = 12.0603(10) Å	β = 96.879(4)°
	c = 23.568(2) Å	γ = 90°
Volume:	7616.1(12) $Å^3$	
Z:	8	
Density (calculated):	1.303 $g·cm^{-3}$	
Absorption coefficient (μ):	0.707 mm^{-1}	
F(000):	3152	
Crystal size:	0.13 × 0.12 × 0.10 mm^3	
θ range for data collection:	1.74 to 25.00°	
Index ranges:	$-32 \leq h \leq 32$, $-14 \leq k \leq 14$, $-28 \leq l \leq 28$	
Reflections collected:	90845	
Independent reflections:	6712 [R_{int} = 0.0540]	
Completeness to θ = 25.00°:	100.0 %	
Absorption correction:	Semi-empirical from equivalents	
Max. and min. transmission:	0.7457 and 0.6757	
Refinement method:	Full-matrix least-squares on F^2	
Data / restraints / parameters:	6712 / 0 / 458	
Goodness-of-fit on F^2:	1.066	
Final R indices [I>2σ(I)]:	R_1 = 0.0267, wR_2 = 0.0661	
R indices (all data):	R_1 = 0.0349, wR_2 = 0.0692	
Largest diff. peak and hole:	0.917 and −0.478 $e^-·Å^{-3}$	

Table S37. Atomic coordinates and equivalent isotropic displacement parameters ($Å^2$) for $[\{PC(sp^2)P\}^{tBu}PdCH_2Ph]^-K^+$ (**6**). U(eq) is defined as one third of the trace of the orthogonalized U_{ij} tensor

atom	x	y	z	U(eq)
Pd	0.04254(1)	0.78119(1)	0.09393(1)	0.016(1)
P(1)	−0.03530(2)	0.70860(5)	0.07545(3)	0.022(1)
K(2)	0.0000	0.90423(8)	0.2500	0.038(1)
P(2)	0.11202(2)	0.87644(5)	0.13175(2)	0.019(1)
C	0.00435(8)	0.91767(18)	0.12240(9)	0.018(1)
C(10)	−0.21394(8)	0.8679(2)	0.11379(11)	0.026(1)
C(11)	−0.04919(8)	0.91110(18)	0.12137(9)	0.019(1)
C(12)	−0.07573(8)	0.81553(19)	0.09731(10)	0.022(1)
C(13)	−0.12711(9)	0.80309(19)	0.09665(10)	0.024(1)
C(15)	−0.13161(9)	0.9687(2)	0.14729(13)	0.038(1)
C(14)	−0.15714(8)	0.88093(19)	0.11867(10)	0.022(1)
C(16)	−0.08056(10)	0.9837(2)	0.14885(13)	0.036(1)
C(17)	−0.23774(10)	0.9537(3)	0.14899(14)	0.047(1)
C(5)	0.07820(9)	0.6421(2)	0.05736(11)	0.025(1)
C(18)	−0.23563(10)	0.8813(3)	0.05164(13)	0.056(1)
C(19)	−0.22805(11)	0.7531(3)	0.13385(19)	0.060(1)
C(20)	0.12494(9)	1.27972(19)	0.23522(10)	0.025(1)
C(21)	0.03330(8)	1.01061(18)	0.14598(9)	0.019(1)
C(22)	0.08662(8)	1.00268(18)	0.15874(9)	0.019(1)
C(24)	0.09491(9)	1.18521(19)	0.20441(10)	0.022(1)
C(23)	0.11499(8)	1.08563(19)	0.18808(10)	0.023(1)
C(27)	0.09845(12)	1.3215(3)	0.28479(13)	0.049(1)
C(26)	0.01486(9)	1.1181(2)	0.15825(12)	0.035(1)
C(25)	0.04403(9)	1.1987(2)	0.18638(13)	0.036(1)
C(28)	0.12824(11)	1.3756(2)	0.19344(12)	0.040(1)
C(29)	0.17765(10)	1.2444(2)	0.25852(12)	0.038(1)
C(31)	−0.05742(9)	0.6628(2)	0.00203(10)	0.027(1)
C(32)	−0.04818(9)	0.5887(2)	0.12049(11)	0.028(1)
C(33)	−0.04488(12)	0.7479(3)	−0.04066(13)	0.038(1)
C(34)	−0.11257(9)	0.6305(2)	−0.01002(13)	0.039(1)
C(36)	−0.02327(10)	0.4819(2)	0.10375(12)	0.033(1)
C(35)	−0.03142(12)	0.6165(2)	0.18297(12)	0.043(1)
C(43)	0.18009(10)	0.7135(2)	0.17619(12)	0.035(1)
C(44)	0.11497(12)	0.7734(3)	0.23639(12)	0.038(1)
C(45)	0.13226(13)	0.9873(3)	0.03519(16)	0.045(1)
C(46)	0.20533(10)	0.9760(3)	0.11179(14)	0.047(1)
C(51)	0.11661(9)	0.6687(2)	0.01925(10)	0.026(1)
C(52)	0.16562(9)	0.6305(2)	0.02957(11)	0.029(1)
C(53)	0.20072(10)	0.6551(2)	−0.00706(13)	0.042(1)

Continued on next page

Table S37. – continued from previous page

atom	x	y	x	U(eq)
C(54)	0.18797(12)	0.7191(3)	−0.05510(14)	0.052(1)
C(55)	0.14040(13)	0.7586(3)	−0.06582(13)	0.056(1)
C(56)	0.10535(10)	0.7327(3)	−0.02987(12)	0.040(1)
C(42)	0.15828(9)	0.9180(2)	0.08351(11)	0.027(1)
C(41)	0.14946(9)	0.8110(2)	0.19381(10)	0.027(1)
K(1)	0.0000	1.0000	0.0000	0.036(1)
H(13)	−0.1423	0.7377	0.0802	0.029
H(15)	−0.1500	1.0207	0.1666	0.045
H(16)	−0.0649(10)	1.044(2)	0.1715(12)	0.041(8)
H(17A)	−0.2299	1.0282	0.1360	0.071
H(17B)	−0.2247	0.9453	0.1894	0.071
H(17C)	−0.2740	0.9432	0.1444	0.071
H(5B)	0.0909(9)	0.593(2)	0.0890(11)	0.029(7)
H(5A)	0.0512(10)	0.604(2)	0.0338(11)	0.031(7)
H(18A)	−0.2282	0.9559	0.0385	0.085
H(18B)	−0.2719	0.8708	0.0481	0.085
H(18C)	−0.2208	0.8260	0.0283	0.085
H(19A)	−0.2145	0.6964	0.1102	0.090
H(19B)	−0.2645	0.7465	0.1304	0.090
H(19C)	−0.2142	0.7428	0.1739	0.090
H(23)	0.1498	1.0736	0.1974	0.027
H(27A)	0.0650	1.3476	0.2702	0.074
H(27B)	0.1176	1.3826	0.3040	0.074
H(27C)	0.0958	1.2610	0.3120	0.074
H(26)	−0.0192	1.1344	0.1463	0.041
H(25)	0.0289	1.2673	0.1940	0.043
H(28A)	0.1451	1.3504	0.1612	0.060
H(28B)	0.1472	1.4367	0.2130	0.060
H(28C)	0.0945	1.4012	0.1794	0.060
H(29A)	0.1957	1.2214	0.2268	0.057
H(29B)	0.1760	1.1821	0.2850	0.057
H(29C)	0.1951	1.3067	0.2787	0.057
H(31)	−0.0378	0.5948	−0.0050	0.033
H(32)	−0.0851	0.5762	0.1161	0.034
H(33C)	−0.0545(12)	0.725(3)	−0.0776(15)	0.051(9)
H(33B)	−0.0635(11)	0.816(3)	−0.0355(12)	0.040(8)
H(33A)	−0.0112(13)	0.765(2)	−0.0361(13)	0.045(9)
H(34A)	−0.1186	0.5943	−0.0474	0.059
H(34B)	−0.1333	0.6972	−0.0100	0.059
H(34C)	−0.1211	0.5794	0.0197	0.059
H(36A)	−0.0361	0.4621	0.0644	0.050
H(36B)	−0.0306	0.4220	0.1296	0.050

Continued on next page

Table S37. – continued from previous page

**atom**	**x**	**y**	**x**	**U(eq)**
H(36C)	0.0129	0.4931	0.1065	0.050
H(35A)	0.0050	0.6247	0.1888	0.065
H(35B)	−0.0414	0.5566	0.2073	0.065
H(35C)	−0.0471	0.6860	0.1929	0.065
H(43A)	0.1576	0.6558	0.1588	0.052
H(43B)	0.2004	0.6834	0.2099	0.052
H(43C)	0.2020	0.7387	0.1484	0.052
H(44C)	0.0881(12)	0.724(2)	0.2165(13)	0.045(9)
H(44B)	0.1332(12)	0.732(3)	0.2656(14)	0.046(9)
H(44A)	0.0999(11)	0.836(3)	0.2517(13)	0.046(9)
H(45A)	0.1230(13)	1.055(3)	0.0480(14)	0.062(11)
H(45B)	0.1031(13)	0.952(3)	0.0171(13)	0.053(10)
H(45C)	0.1525(13)	1.003(3)	0.0087(15)	0.058(10)
H(46A)	0.2295	0.9829	0.0841	0.070
H(46B)	0.2200	0.9323	0.1446	0.070
H(46C)	0.1966	1.0499	0.1247	0.070
H(52)	0.1752	0.5865	0.0625	0.035
H(53)	0.2338	0.6276	0.0010	0.050
H(54)	0.2119	0.7355	−0.0803	0.062
H(55)	0.1314	0.8042	−0.0983	0.067
H(56)	0.0723	0.7596	−0.0389	0.047
H(42)	0.1695	0.8483	0.0659	0.033
H(41)	0.1729	0.8678	0.2127	0.033

Table S38. Anisotropic displacement parameters (Å^2) for [{PC(sp^2)P}^{tBu}PdCH$_2$Ph]$^-$K$^+$ (**6**). The anisotropic displacement factor exponent takes the form: $-2\pi^2[h^2a^{*2}U_{11} + ... + 2hka^*b^*U_{12}]$.

**atom**	**U$_{11}$**	**U$_{22}$**	**U$_{33}$**	**U$_{23}$**	**U$_{13}$**	**U$_{12}$**
Pd	0.0137(1)	0.0157(1)	0.0190(1)	−0.0036(1)	0.0036(1)	0.0000(1)
P(1)	0.0155(3)	0.0194(3)	0.0310(3)	−0.0090(3)	0.0045(2)	−0.0016(2)
K(2)	0.0336(4)	0.0565(6)	0.0238(4)	0.000	0.0061(3)	0.000
P(2)	0.0139(3)	0.0179(3)	0.0254(3)	−0.0047(2)	0.0037(2)	0.0002(2)
C	0.0173(11)	0.0174(11)	0.0189(11)	−0.0006(9)	0.0044(9)	0.0006(9)
C(10)	0.0169(12)	0.0277(13)	0.0357(14)	−0.0009(11)	0.0065(10)	−0.0007(10)
C(11)	0.0185(11)	0.0188(11)	0.0191(11)	0.0005(9)	0.0050(9)	0.0014(9)
C(12)	0.0174(11)	0.0211(12)	0.0272(12)	−0.0060(10)	0.0050(9)	0.0002(9)
C(13)	0.0204(12)	0.0210(12)	0.0312(13)	−0.0064(10)	0.0044(10)	−0.0029(9)
C(15)	0.0236(14)	0.0258(14)	0.0655(19)	−0.0106(13)	0.0131(13)	0.0022(11)
C(14)	0.0157(11)	0.0228(12)	0.0282(12)	0.0008(10)	0.0052(9)	0.0009(9)
C(16)	0.0237(13)	0.0232(13)	0.0620(19)	−0.0139(13)	0.0098(12)	−0.0023(11)
C(17)	0.0216(14)	0.0537(19)	0.070(2)	−0.0204(17)	0.0172(14)	−0.0020(13)
C(5)	0.0210(13)	0.0230(13)	0.0319(14)	−0.0093(11)	0.0049(10)	−0.0012(10)
C(18)	0.0182(14)	0.105(3)	0.0455(18)	0.0010(18)	0.0001(12)	−0.0047(16)
C(19)	0.0230(15)	0.0386(18)	0.123(3)	0.0149(19)	0.0243(18)	−0.0034(13)
C(20)	0.0267(13)	0.0201(12)	0.0286(13)	−0.0046(10)	0.0059(10)	−0.0054(10)
C(21)	0.0183(11)	0.0182(11)	0.0208(11)	−0.0003(9)	0.0051(9)	0.0009(9)
C(22)	0.0158(11)	0.0162(11)	0.0262(12)	−0.0029(9)	0.0065(9)	0.0003(9)
C(24)	0.0233(12)	0.0202(12)	0.0234(12)	−0.0046(10)	0.0070(9)	−0.0039(10)
C(23)	0.0175(11)	0.0240(13)	0.0267(12)	−0.0045(10)	0.0036(9)	−0.0018(10)
C(27)	0.056(2)	0.0487(18)	0.0465(18)	−0.0275(15)	0.0192(15)	−0.0160(16)
C(26)	0.0187(13)	0.0254(14)	0.0600(18)	−0.0109(13)	0.0067(12)	0.0021(11)
C(25)	0.0235(13)	0.0235(13)	0.0607(18)	−0.0157(12)	0.0105(12)	0.0003(10)
C(28)	0.0414(16)	0.0306(15)	0.0465(17)	0.0058(13)	−0.0042(13)	−0.0160(13)
C(29)	0.0374(16)	0.0282(14)	0.0444(16)	−0.0070(12)	−0.0081(13)	−0.0064(12)
C(31)	0.0238(13)	0.0236(13)	0.0339(14)	−0.0118(11)	−0.0003(10)	0.0025(10)
C(32)	0.0238(13)	0.0228(13)	0.0397(14)	−0.0065(11)	0.0126(11)	−0.0054(10)
C(33)	0.0349(17)	0.0372(17)	0.0378(17)	−0.0046(13)	−0.0090(13)	0.0026(13)
C(34)	0.0265(14)	0.0388(16)	0.0508(17)	−0.0228(13)	−0.0029(12)	−0.0031(12)
C(36)	0.0369(15)	0.0241(13)	0.0404(15)	−0.0004(11)	0.0125(12)	0.0012(11)
C(35)	0.061(2)	0.0353(16)	0.0359(16)	−0.0049(13)	0.0200(14)	−0.0103(14)
C(43)	0.0328(15)	0.0339(15)	0.0356(14)	−0.0017(12)	−0.0016(11)	0.0129(12)
C(44)	0.0385(17)	0.0485(18)	0.0257(14)	0.0031(14)	0.0007(12)	0.0101(15)
C(45)	0.0422(19)	0.0424(19)	0.057(2)	0.0168(16)	0.0276(17)	0.0066(15)
C(46)	0.0322(16)	0.0421(17)	0.070(2)	−0.0212(15)	0.0255(14)	−0.0156(13)
C(51)	0.0244(13)	0.0240(13)	0.0291(13)	−0.0123(11)	0.0057(10)	−0.0018(10)
C(52)	0.0235(13)	0.0259(13)	0.0400(15)	−0.0074(11)	0.0079(11)	0.0007(10)
C(53)	0.0264(14)	0.0397(17)	0.063(2)	−0.0113(15)	0.0160(13)	0.0000(12)
C(54)	0.0479(19)	0.064(2)	0.0481(19)	−0.0024(17)	0.0271(15)	−0.0057(16)

Continued on next page

Table S38. – continued from previous page

atom	U_{11}	U_{22}	U_{33}	U_{23}	U_{13}	U_{12}
C(55)	0.053(2)	0.079(2)	0.0356(17)	0.0111(16)	0.0106(15)	−0.0027(18)
C(56)	0.0292(15)	0.0523(18)	0.0366(15)	0.0005(14)	0.0026(12)	0.0014(13)
C(42)	0.0207(12)	0.0222(13)	0.0418(15)	−0.0058(11)	0.0140(11)	−0.0011(10)
C(41)	0.0217(12)	0.0299(13)	0.0289(13)	−0.0076(11)	−0.0037(10)	0.0035(10)
K(1)	0.0468(5)	0.0304(4)	0.0299(4)	0.0062(3)	−0.0023(4)	−0.0007(4)

Table S39. Distances [Å] for [{PC(sp^2)P}^{tBu}PdCH$_2$Ph]$^-$K$^+$ (**6**).

**atom – atom**	**distance**	**atom – atom**	**distance**
Pd – C	2.095(2)	Pd – C(5)	2.163(2)
Pd – P(1)	2.2695(6)	Pd – P(2)	2.2868(6)
Pd – K(1)	3.5460(3)	P(1) – C(12)	1.804(2)
P(1) – C(31)	1.846(2)	P(1) – C(32)	1.852(3)
K(2) – C(21)#1	2.998(2)	K(2) – C(21)	2.998(2)
K(2) – C#1	3.028(2)	K(2) – C	3.028(2)
K(2) – C(11)#1	3.162(2)	K(2) – C(11)	3.162(2)
K(2) – C(16)#1	3.176(3)	K(2) – C(16)	3.176(3)
K(2) – C(26)#1	3.420(3)	K(2) – C(26)	3.420(3)
K(2) – C(44)#1	3.530(3)	K(2) – C(44)	3.530(3)
K(2) – H(16)	2.93(3)	K(2) – H(44A)	2.82(3)
P(2) – C(22)	1.816(2)	P(2) – C(41)	1.852(2)
P(2) – C(42)	1.855(2)	C – C(21)	1.439(3)
C – C(11)	1.444(3)	C – K(1)	3.040(2)
C(10) – C(17)	1.517(4)	C(10) – C(18)	1.520(4)
C(10) – C(19)	1.525(4)	C(10) – C(14)	1.532(3)
C(11) – C(16)	1.426(3)	C(11) – C(12)	1.437(3)
C(11) – K(1)	3.465(2)	C(12) – C(13)	1.393(3)
C(13) – C(14)	1.381(3)	C(13) – H(13)	0.9500
C(15) – C(16)	1.386(4)	C(15) – C(14)	1.393(3)
C(15) – H(15)	0.9500	C(16) – H(16)	0.97(3)
C(17) – H(17A)	0.9800	C(17) – H(17B)	0.9800
C(17) – H(17C)	0.9800	C(5) – C(51)	1.486(3)
C(5) – H(5B)	0.98(3)	C(5) – H(5A)	0.98(3)
C(18) – H(18A)	0.9800	C(18) – H(18B)	0.9800
C(18) – H(18C)	0.9800	C(19) – H(19A)	0.9800
C(19) – H(19B)	0.9800	C(19) – H(19C)	0.9800
C(20) – C(29)	1.523(4)	C(20) – C(27)	1.526(4)
C(20) – C(28)	1.528(4)	C(20) – C(24)	1.531(3)
C(21) – C(26)	1.430(3)	C(21) – C(22)	1.438(3)
C(21) – K(1)	3.453(2)	C(22) – C(23)	1.392(3)
C(24) – C(23)	1.391(3)	C(24) – C(25)	1.397(3)
C(23) – H(23)	0.9500	C(27) – H(27A)	0.9800
C(27) – H(27B)	0.9800	C(27) – H(27C)	0.9800
C(26) – C(25)	1.371(4)	C(26) – H(26)	0.9500
C(25) – H(25)	0.9500	C(28) – H(28A)	0.9800
C(28) – H(28B)	0.9800	C(28) – H(28C)	0.9800
C(29) – H(29A)	0.9800	C(29) – H(29B)	0.9800
C(29) – H(29C)	0.9800	C(31) – C(33)	1.504(4)
C(31) – C(34)	1.532(3)	C(31) – H(31)	1.0000

Symmetry transformations used to generate equivalent atoms: #1 $-x, y, -z + \frac{1}{2}$; #2 $-x, -y + 2, -z$

Continued on next page

Table S39. – continued from previous page

**atom – atom**	**distance**	**atom – atom**	**distance**
C(32) – C(35)	1.525(4)	C(32) – C(36)	1.526(3)
C(32) – H(32)	1.0000	C(33) – K(1)	3.369(3)
C(33) – H(33C)	0.92(3)	C(33) – H(33B)	0.98(3)
C(33) – H(33A)	0.93(3)	C(34) – H(34A)	0.9800
C(34) – H(34B)	0.9800	C(34) – H(34C)	0.9800
C(36) – H(36A)	0.9800	C(36) – H(36B)	0.9800
C(36) – H(36C)	0.9800	C(35) – H(35A)	0.9800
C(35) – H(35B)	0.9800	C(35) – H(35C)	0.9800
C(43) – C(41)	1.523(3)	C(43) – H(43A)	0.9800
C(43) – H(43B)	0.9800	C(43) – H(43C)	0.9800
C(44) – C(41)	1.518(4)	C(44) – H(44C)	1.01(3)
C(44) – H(44B)	0.94(3)	C(44) – H(44A)	0.95(3)
C(45) – C(42)	1.516(4)	C(45) – H(45A)	0.91(4)
C(45) – H(45B)	0.95(3)	C(45) – H(45C)	0.90(4)
C(46) – C(42)	1.531(4)	C(46) – H(46A)	0.9800
C(46) – H(46B)	0.9800	C(46) – H(46C)	0.9800
C(51) – C(52)	1.394(3)	C(51) – C(56)	1.395(4)
C(52) – C(53)	1.388(4)	C(52) – H(52)	0.9500
C(53) – C(54)	1.380(4)	C(53) – H(53)	0.9500
C(54) – C(55)	1.364(5)	C(54) – H(54)	0.9500
C(55) – C(56)	1.379(4)	C(55) – H(55)	0.9500
C(56) – H(56)	0.9500	C(42) – H(42)	1.0000
C(41) – H(41)	1.0000	K(1) – C#2	3.040(2)
K(1) – C(33)#2	3.369(3)	K(1) – C(21)#2	3.453(2)
K(1) – C(11)#2	3.465(2)	K(1) – Pd#2	3.5460(3)
K(1) – H(33B)	2.87(3)	K(1) – H(33A)	2.96(3)
K(1) – H(45B)	2.82(3)		

Symmetry transformations used to generate equivalent atoms: #1 $-x, y, -z + \frac{1}{2}$; #2 $-x, -y + 2, -z$

Table S40. Angles [°] for [{PC(sp^2)P}^{tBu}PdCH$_2$Ph]$^-$K$^+$ (**6**).

atom – atom – atom	angle	atom – atom – atom	angle
C – Pd – C(5)	175.02(9)	C – Pd – P(1)	83.38(6)
C(5) – Pd – P(1)	94.03(7)	C – Pd – P(2)	83.83(6)
C(5) – Pd – P(2)	99.30(7)	P(1) – Pd – P(2)	164.91(2)
C – Pd – K(1)	58.68(6)	C(5) – Pd – K(1)	117.03(7)
P(1) – Pd – K(1)	86.801(17)	P(2) – Pd – K(1)	93.357(17)
C(12) – P(1) – C(31)	110.05(11)	C(12) – P(1) – C(32)	103.49(11)
C(31) – P(1) – C(32)	103.99(11)	C(12) – P(1) – Pd	104.31(8)
C(31) – P(1) – Pd	118.90(8)	C(32) – P(1) – Pd	115.11(8)
C(21)#1 – K(2) – C(21)	129.34(9)	C(21)#1 – K(2) – C#1	27.63(6)
C(21) – K(2) – C#1	147.16(7)	C(21)#1 – K(2) – C	147.16(7)
C(21) – K(2) – C	27.63(6)	C#1 – K(2) – C	173.86(9)
C(21)#1 – K(2) – C(11)#1	48.50(6)	C(21) – K(2) – C(11)#1	129.81(6)
C#1 – K(2) – C(11)#1	26.88(6)	C – K(2) – C(11)#1	152.77(6)
C(21)#1 – K(2) – C(11)	129.81(6)	C(21) – K(2) – C(11)	48.50(6)
C#1 – K(2) – C(11)	152.77(6)	C – K(2) – C(11)	26.88(6)
C(11)#1 – K(2) – C(11)	177.00(9)	C(21)#1 – K(2) – C(16)#1	60.16(6)
C(21) – K(2) – C(16)#1	103.84(7)	C#1 – K(2) – C(16)#1	48.92(6)
C – K(2) – C(16)#1	128.67(7)	C(11)#1 – K(2) – C(16)#1	26.01(6)
C(11) – K(2) – C(16)#1	151.99(7)	C(21)#1 – K(2) – C(16)	103.84(7)
C(21) – K(2) – C(16)	60.16(6)	C#1 – K(2) – C(16)	128.67(7)
C – K(2) – C(16)	48.92(6)	C(11)#1 – K(2) – C(16)	151.99(7)
C(11) – K(2) – C(16)	26.01(6)	C(16)#1 – K(2) – C(16)	144.87(10)
C(21)#1 – K(2) – C(26)#1	24.64(6)	C(21) – K(2) – C(26)#1	105.28(7)
C#1 – K(2) – C(26)#1	46.39(6)	C – K(2) – C(26)#1	127.52(7)
C(11)#1 – K(2) – C(26)#1	56.05(6)	C(11) – K(2) – C(26)#1	121.26(6)
C(16)#1 – K(2) – C(26)#1	54.51(7)	C(16) – K(2) – C(26)#1	97.20(7)
C(21)#1 – K(2) – C(26)	105.28(7)	C(21) – K(2) – C(26)	24.64(6)
C#1 – K(2) – C(26)	127.52(7)	C – K(2) – C(26)	46.39(6)
C(11)#1 – K(2) – C(26)	121.26(6)	C(11) – K(2) – C(26)	56.05(6)
C(16)#1 – K(2) – C(26)	97.20(7)	C(16) – K(2) – C(26)	54.51(7)
C(26)#1 – K(2) – C(26)	82.10(9)	C(21)#1 – K(2) – C(44)#1	76.36(6)
C(21) – K(2) – C(44)#1	128.20(6)	C#1 – K(2) – C(44)#1	78.15(6)
C – K(2) – C(44)#1	104.67(6)	C(11)#1 – K(2) – C(44)#1	101.38(6)
C(11) – K(2) – C(44)#1	79.98(6)	C(16)#1 – K(2) – C(44)#1	126.65(7)
C(16) – K(2) – C(44)#1	70.91(7)	C(26)#1 – K(2) – C(44)#1	96.24(7)
C(26) – K(2) – C(44)#1	124.44(7)	C(21)#1 – K(2) – C(44)	128.20(6)
C(21) – K(2) – C(44)	76.36(6)	C#1 – K(2) – C(44)	104.67(6)
C – K(2) – C(44)	78.15(6)	C(11)#1 – K(2) – C(44)	79.98(6)
C(11) – K(2) – C(44)	101.38(6)	C(16)#1 – K(2) – C(44)	70.91(7)
C(16) – K(2) – C(44)	126.65(7)	C(26)#1 – K(2) – C(44)	124.44(7)

Symmetry transformations used to generate equivalent atoms: #1 $-x, y, -z + \frac{1}{2}$; #2 $-x, -y + 2, -z$

Continued on next page

Table S40. – continued from previous page

atom – atom – atom	angle	atom – atom – atom	angle
C(26) – K(2) – C(44)	96.24(7)	C(44)#1 – K(2) – C(44)	126.90(12)
C(21)#1 – K(2) – H(16)	93.1(6)	C(21) – K(2) – H(16)	56.8(5)
C#1 – K(2) – H(16)	120.3(6)	C – K(2) – H(16)	55.5(6)
C(11)#1 – K(2) – H(16)	137.8(6)	C(11) – K(2) – H(16)	39.5(6)
C(16)#1 – K(2) – H(16)	127.2(6)	C(16) – K(2) – H(16)	17.7(6)
C(26)#1 – K(2) – H(16)	81.8(6)	C(26) – K(2) – H(16)	43.3(6)
C(44)#1 – K(2) – H(16)	81.3(6)	C(44) – K(2) – H(16)	131.8(6)
C(21)#1 – K(2) – H(44A)	119.6(6)	C(21) – K(2) – H(44A)	75.9(6)
C#1 – K(2) – H(44A)	98.7(6)	C – K(2) – H(44A)	83.1(6)
C(11)#1 – K(2) – H(44A)	72.7(6)	C(11) – K(2) – H(44A)	108.3(6)
C(16)#1 – K(2) – H(44A)	60.6(6)	C(16) – K(2) – H(44A)	131.9(6)
C(26)#1 – K(2) – H(44A)	113.3(6)	C(26) – K(2) – H(44A)	92.6(6)
C(44)#1 – K(2) – H(44A)	136.0(6)	C(44) – K(2) – H(44A)	11.4(6)
H(16) – K(2) – H(44A)	132.7(8)	C(22) – P(2) – C(41)	105.89(11)
C(22) – P(2) – C(42)	107.37(11)	C(41) – P(2) – C(42)	104.79(11)
C(22) – P(2) – Pd	103.38(7)	C(41) – P(2) – Pd	115.99(8)
C(42) – P(2) – Pd	118.51(8)	C(21) – C – C(11)	123.02(19)
C(21) – C – Pd	118.06(15)	C(11) – C – Pd	118.76(15)
C(21) – C – K(2)	75.05(12)	C(11) – C – K(2)	81.73(12)
Pd – C – K(2)	110.80(8)	C(21) – C – K(1)	93.99(13)
C(11) – C – K(1)	94.42(13)	Pd – C – K(1)	85.25(7)
K(2) – C – K(1)	163.40(8)	C(17) – C(10) – C(18)	108.3(2)
C(17) – C(10) – C(19)	108.2(2)	C(18) – C(10) – C(19)	108.3(3)
C(17) – C(10) – C(14)	112.0(2)	C(18) – C(10) – C(14)	109.1(2)
C(19) – C(10) – C(14)	110.8(2)	C(16) – C(11) – C(12)	112.1(2)
C(16) – C(11) – C	127.3(2)	C(12) – C(11) – C	120.06(19)
C(16) – C(11) – K(2)	77.56(15)	C(12) – C(11) – K(2)	119.31(15)
C – C(11) – K(2)	71.39(12)	C(16) – C(11) – K(1)	119.46(16)
C(12) – C(11) – K(1)	98.31(14)	C – C(11) – K(1)	61.02(11)
K(2) – C(11) – K(1)	129.89(7)	C(13) – C(12) – C(11)	122.6(2)
C(13) – C(12) – P(1)	123.72(18)	C(11) – C(12) – P(1)	113.33(16)
C(14) – C(13) – C(12)	123.4(2)	C(14) – C(13) – H(13)	118.3
C(12) – C(13) – H(13)	118.3	C(16) – C(15) – C(14)	123.0(2)
C(16) – C(15) – H(15)	118.5	C(14) – C(15) – H(15)	118.5
C(13) – C(14) – C(15)	114.8(2)	C(13) – C(14) – C(10)	121.9(2)
C(15) – C(14) – C(10)	123.2(2)	C(15) – C(16) – C(11)	123.4(2)
C(15) – C(16) – K(2)	124.7(2)	C(11) – C(16) – K(2)	76.43(14)
C(15) – C(16) – H(16)	118.5(17)	C(11) – C(16) – H(16)	118.0(17)
K(2) – C(16) – H(16)	66.5(17)	C(10) – C(17) – H(17A)	109.5
C(10) – C(17) – H(17B)	109.5	H(17A) – C(17) – H(17B)	109.5

Symmetry transformations used to generate equivalent atoms: #1 $-x, y, -z+\frac{1}{2}$; #2 $-x, -y+2, -z$

Continued on next page

Table S40. – continued from previous page

atom – atom – atom	angle	atom – atom – atom	angle
C(10) – C(17) – H(17C)	109.5	H(17A) – C(17) – H(17C)	109.5
H(17B) – C(17) – H(17C)	109.5	C(51) – C(5) – Pd	116.71(17)
C(51) – C(5) – H(5B)	112.6(15)	Pd – C(5) – H(5B)	107.3(15)
C(51) – C(5) – H(5A)	106.6(15)	Pd – C(5) – H(5A)	104.8(15)
H(5B) – C(5) – H(5A)	108(2)	C(10) – C(18) – H(18A)	109.5
C(10) – C(18) – H(18B)	109.5	H(18A) – C(18) – H(18B)	109.5
C(10) – C(18) – H(18C)	109.5	H(18A) – C(18) – H(18C)	109.5
H(18B) – C(18) – H(18C)	109.5	C(10) – C(19) – H(19A)	109.5
C(10) – C(19) – H(19B)	109.5	H(19A) – C(19) – H(19B)	109.5
C(10) – C(19) – H(19C)	109.5	H(19A) – C(19) – H(19C)	109.5
H(19B) – C(19) – H(19C)	109.5	C(29) – C(20) – C(27)	108.6(2)
C(29) – C(20) – C(28)	108.7(2)	C(27) – C(20) – C(28)	108.2(2)
C(29) – C(20) – C(24)	112.4(2)	C(27) – C(20) – C(24)	109.8(2)
C(28) – C(20) – C(24)	109.0(2)	C(26) – C(21) – C(22)	112.4(2)
C(26) – C(21) – C	126.7(2)	C(22) – C(21) – C	120.83(19)
C(26) – C(21) – K(2)	94.42(15)	C(22) – C(21) – K(2)	101.02(14)
C – C(21) – K(2)	77.32(12)	C(26) – C(21) – K(1)	100.35(15)
C(22) – C(21) – K(1)	109.91(13)	C – C(21) – K(1)	61.44(11)
K(2) – C(21) – K(1)	136.98(7)	C(23) – C(22) – C(21)	122.2(2)
C(23) – C(22) – P(2)	124.63(17)	C(21) – C(22) – P(2)	113.13(16)
C(23) – C(24) – C(25)	114.7(2)	C(23) – C(24) – C(20)	124.9(2)
C(25) – C(24) – C(20)	120.2(2)	C(24) – C(23) – C(22)	123.3(2)
C(24) – C(23) – H(23)	118.4	C(22) – C(23) – H(23)	118.4
C(20) – C(27) – H(27A)	109.5	C(20) – C(27) – H(27B)	109.5
H(27A) – C(27) – H(27B)	109.5	C(20) – C(27) – H(27C)	109.5
H(27A) – C(27) – H(27C)	109.5	H(27B) – C(27) – H(27C)	109.5
C(25) – C(26) – C(21)	123.2(2)	C(25) – C(26) – K(2)	109.5(2)
C(21) – C(26) – K(2)	60.94(13)	C(25) – C(26) – H(26)	118.4
C(21) – C(26) – H(26)	118.4	K(2) – C(26) – H(26)	99.2
C(26) – C(25) – C(24)	123.4(2)	C(26) – C(25) – H(25)	118.3
C(24) – C(25) – H(25)	118.3	C(20) – C(28) – H(28A)	109.5
C(20) – C(28) – H(28B)	109.5	H(28A) – C(28) – H(28B)	109.5
C(20) – C(28) – H(28C)	109.5	H(28A) – C(28) – H(28C)	109.5
H(28B) – C(28) – H(28C)	109.5	C(20) – C(29) – H(29A)	109.5
C(20) – C(29) – H(29B)	109.5	H(29A) – C(29) – H(29B)	109.5
C(20) – C(29) – H(29C)	109.5	H(29A) – C(29) – H(29C)	109.5
H(29B) – C(29) – H(29C)	109.5	C(33) – C(31) – C(34)	109.9(2)
C(33) – C(31) – P(1)	110.45(18)	C(34) – C(31) – P(1)	116.59(18)
C(33) – C(31) – H(31)	106.4	C(34) – C(31) – H(31)	106.4
P(1) – C(31) – H(31)	106.4	C(35) – C(32) – C(36)	110.3(2)

Symmetry transformations used to generate equivalent atoms: #1 $-x, y, -z + \frac{1}{2}$; #2 $-x, -y + 2, -z$

Continued on next page

Table S40. – continued from previous page

atom – atom – atom	angle	atom – atom – atom	angle
C(35) – C(32) – P(1)	109.10(17)	C(36) – C(32) – P(1)	113.08(17)
C(35) – C(32) – H(32)	108.1	C(36) – C(32) – H(32)	108.1
P(1) – C(32) – H(32)	108.1	C(31) – C(33) – K(1)	121.85(18)
C(31) – C(33) – H(33C)	111(2)	K(1) – C(33) – H(33C)	126(2)
C(31) – C(33) – H(33B)	109.2(17)	K(1) – C(33) – H(33B)	51.6(17)
H(33C) – C(33) – H(33B)	106(3)	C(31) – C(33) – H(33A)	111.8(19)
K(1) – C(33) – H(33A)	56.5(19)	H(33C) – C(33) – H(33A)	110(3)
H(33B) – C(33) – H(33A)	108(3)	C(31) – C(34) – H(34A)	109.5
C(31) – C(34) – H(34B)	109.5	H(34A) – C(34) – H(34B)	109.5
C(31) – C(34) – H(34C)	109.5	H(34A) – C(34) – H(34C)	109.5
H(34B) – C(34) – H(34C)	109.5	C(32) – C(36) – H(36A)	109.5
C(32) – C(36) – H(36B)	109.5	H(36A) – C(36) – H(36B)	109.5
C(32) – C(36) – H(36C)	109.5	H(36A) – C(36) – H(36C)	109.5
H(36B) – C(36) – H(36C)	109.5	C(32) – C(35) – H(35A)	109.5
C(32) – C(35) – H(35B)	109.5	H(35A) – C(35) – H(35B)	109.5
C(32) – C(35) – H(35C)	109.5	H(35A) – C(35) – H(35C)	109.5
H(35B) – C(35) – H(35C)	109.5	C(41) – C(43) – H(43A)	109.5
C(41) – C(43) – H(43B)	109.5	H(43A) – C(43) – H(43B)	109.5
C(41) – C(43) – H(43C)	109.5	H(43A) – C(43) – H(43C)	109.5
H(43B) – C(43) – H(43C)	109.5	C(41) – C(44) – K(2)	122.83(18)
C(41) – C(44) – H(44C)	109.4(17)	K(2) – C(44) – H(44C)	73.6(17)
C(41) – C(44) – H(44B)	109.6(19)	K(2) – C(44) – H(44B)	124.0(19)
H(44C) – C(44) – H(44B)	108(2)	C(41) – C(44) – H(44A)	109.8(19)
K(2) – C(44) – H(44A)	35.8(18)	H(44C) – C(44) – H(44A)	109(2)
H(44B) – C(44) – H(44A)	111(3)	C(42) – C(45) – H(45A)	111(2)
C(42) – C(45) – H(45B)	112(2)	H(45A) – C(45) – H(45B)	108(3)
C(42) – C(45) – H(45C)	112(2)	H(45A) – C(45) – H(45C)	104(3)
H(45B) – C(45) – H(45C)	109(3)	C(42) – C(46) – H(46A)	109.5
C(42) – C(46) – H(46B)	109.5	H(46A) – C(46) – H(46B)	109.5
C(42) – C(46) – H(46C)	109.5	H(46A) – C(46) – H(46C)	109.5
H(46B) – C(46) – H(46C)	109.5	C(52) – C(51) – C(56)	115.8(2)
C(52) – C(51) – C(5)	122.6(2)	C(56) – C(51) – C(5)	121.5(2)
C(53) – C(52) – C(51)	121.7(3)	C(53) – C(52) – H(52)	119.2
C(51) – C(52) – H(52)	119.2	C(54) – C(53) – C(52)	120.5(3)
C(54) – C(53) – H(53)	119.7	C(52) – C(53) – H(53)	119.7
C(55) – C(54) – C(53)	118.9(3)	C(55) – C(54) – H(54)	120.5
C(53) – C(54) – H(54)	120.5	C(54) – C(55) – C(56)	120.5(3)
C(54) – C(55) – H(55)	119.8	C(56) – C(55) – H(55)	119.8
C(55) – C(56) – C(51)	122.5(3)	C(55) – C(56) – H(56)	118.7
C(51) – C(56) – H(56)	118.7	C(45) – C(42) – C(46)	111.1(2)

Symmetry transformations used to generate equivalent atoms: #1 $-x, y, -z+\frac{1}{2}$; #2 $-x, -y+2, -z$

Continued on next page

Table S40. – continued from previous page

atom – atom – atom	angle	atom – atom – atom	angle
C(45) – C(42) – P(2)	108.90(18)	C(46) – C(42) – P(2)	116.14(19)
C(45) – C(42) – H(42)	106.7	C(46) – C(42) – H(42)	106.7
P(2) – C(42) – H(42)	106.7	C(44) – C(41) – C(43)	110.2(2)
C(44) – C(41) – P(2)	109.25(18)	C(43) – C(41) – P(2)	112.09(17)
C(44) – C(41) – H(41)	108.4	C(43) – C(41) – H(41)	108.4
P(2) – C(41) – H(41)	108.4	C#2 – K(1) – C	180.0
C#2 – K(1) – C(33)#2	86.95(7)	C – K(1) – C(33)#2	93.05(7)
C#2 – K(1) – C(33)	93.05(7)	C – K(1) – C(33)	86.95(7)
C(33)#2 – K(1) – C(33)	180.0	C#2 – K(1) – C(21)#2	24.58(5)
C – K(1) – C(21)#2	155.42(5)	C(33)#2 – K(1) – C(21)#2	111.02(6)
C(33) – K(1) – C(21)#2	68.98(6)	C#2 – K(1) – C(21)	155.42(5)
C – K(1) – C(21)	24.58(5)	C(33)#2 – K(1) – C(21)	68.98(6)
C(33) – K(1) – C(21)	111.02(6)	C(21)#2 – K(1) – C(21)	180.00(7)
C#2 – K(1) – C(11)	155.44(5)	C – K(1) – C(11)	24.56(5)
C(33)#2 – K(1) – C(11)	101.81(7)	C(33) – K(1) – C(11)	78.19(7)
C(21)#2 – K(1) – C(11)	137.01(5)	C(21) – K(1) – C(11)	42.99(5)
C#2 – K(1) – C(11)#2	24.56(5)	C – K(1) – C(11)#2	155.44(5)
C(33)#2 – K(1) – C(11)#2	78.19(7)	C(33) – K(1) – C(11)#2	101.81(7)
C(21)#2 – K(1) – C(11)#2	42.99(5)	C(21) – K(1) – C(11)#2	137.01(5)
C(11) – K(1) – C(11)#2	180.00(7)	C#2 – K(1) – Pd	143.93(4)
C – K(1) – Pd	36.07(4)	C(33)#2 – K(1) – Pd	114.55(5)
C(33) – K(1) – Pd	65.45(5)	C(21)#2 – K(1) – Pd	128.37(4)
C(21) – K(1) – Pd	51.63(4)	C(11) – K(1) – Pd	51.81(4)
C(11)#2 – K(1) – Pd	128.19(4)	C#2 – K(1) – Pd#2	36.07(4)
C – K(1) – Pd#2	143.93(4)	C(33)#2 – K(1) – Pd#2	65.45(5)
C(33) – K(1) – Pd#2	114.55(5)	C(21)#2 – K(1) – Pd#2	51.63(4)
C(21) – K(1) – Pd#2	128.37(4)	C(11) – K(1) – Pd#2	128.19(4)
C(11)#2 – K(1) – Pd#2	51.81(4)	Pd – K(1) – Pd#2	180.0
C#2 – K(1) – H(33B)	91.3(6)	C – K(1) – H(33B)	88.7(6)
C(33)#2 – K(1) – H(33B)	164.5(6)	C(33) – K(1) – H(33B)	15.5(6)
C(21)#2 – K(1) – H(33B)	66.8(6)	C(21) – K(1) – H(33B)	113.2(6)
C(11) – K(1) – H(33B)	74.1(6)	C(11)#2 – K(1) – H(33B)	105.9(6)
Pd – K(1) – H(33B)	75.1(6)	Pd#2 – K(1) – H(33B)	104.9(6)
C#2 – K(1) – H(33A)	92.8(6)	C – K(1) – H(33A)	87.2(6)
C(33)#2 – K(1) – H(33A)	164.9(6)	C(33) – K(1) – H(33A)	15.1(6)
C(21)#2 – K(1) – H(33A)	70.9(6)	C(21) – K(1) – H(33A)	109.1(6)
C(11) – K(1) – H(33A)	84.5(6)	C(11)#2 – K(1) – H(33A)	95.5(6)
Pd – K(1) – H(33A)	58.8(6)	Pd#2 – K(1) – H(33A)	121.2(6)
H(33B) – K(1) – H(33A)	30.5(8)	C#2 – K(1) – H(45B)	97.3(6)
C – K(1) – H(45B)	82.7(6)	C(33)#2 – K(1) – H(45B)	80.2(7)

Symmetry transformations used to generate equivalent atoms: #1 $-x, y, -z+\frac{1}{2}$; #2 $-x, -y+2, -z$

Continued on next page

Table S40. – continued from previous page

atom – atom – atom	angle	atom – atom – atom	angle
C(33) – K(1) – H(45B)	99.8(7)	C(21)#2 – K(1) – H(45B)	105.6(6)
C(21) – K(1) – H(45B)	74.4(6)	C(11) – K(1) – H(45B)	106.7(6)
C(11)#2 – K(1) – H(45B)	73.3(6)	Pd – K(1) – H(45B)	61.2(7)
Pd#2 – K(1) – H(45B)	118.8(7)	H(33B) – K(1) – H(45B)	115.3(9)
H(33A) – K(1) – H(45B)	84.8(10)		

Symmetry transformations used to generate equivalent atoms: #1 $-x, y, -z + \frac{1}{2}$; #2 $-x, -y + 2, -z$
